# Supplementary material for: Conditioned culture medium of bone marrow mesenchymal stem cells promotes phenotypic transformation of microglia by regulating mitochondrial autophagy
Source: PeerJ. 2024 Jul 4;12:e17664. doi: 10.7717/peerj.17664 (PMC11227809; doi:10.7717/peerj.17664)
Supplement: Data S1 [file peerj-12-17664-s001.zip › raw data1/1.Rt-qPCR/primer/Arg-1.docx]

Mus musculus arginase, liver (Arg1), mRNA

NCBI Reference Sequence: NM_007482.3

GenBank Graphics

>NM_007482.3:103-1074 Mus musculus arginase, liver (Arg1), mRNA

Forward primer TAGACAAGCTGGGGATTGGC

Reverse primer GCTCAGGTGAATCGGCCTT

product length 83

ATGAGCTCCAAGCCAAAGTCCTTAGAGATTATCGGAGCGCCTTTCTCAAAAGGACAGCCTCGAGGAGGGGTAGAGAAAGGCCCTGCAGCACTGAGGAAAGCTGGTCTGCTGGAAAAACTTAAAGAAACAGAGTATGACGTGAGAGACCACGGGGACCTGGCCTTTGTTGATGTCCCTAATGACAGCTCCTTTCAAATTGTGAAGAACCCACGGTCTGTGGGGAAAGCCAATGAAGAGCTGGCTGGTGTGGTGGCAGAGGTCCAGAAGAATGGAAGAGTCAGTGTGGTGCTGGGTGGAGACCACAGTCTGGCAGTTGGAAGCATCTCTGGCCACGCCAGGGTCCACCCTGACCTATGTGTCATTTGGGTGGATGCTCACACTGACATCAACACTCCCCTGACAACCAGCTCTGGGAATCTGCATGGGCAACCTGTGTCCTTTCTCCTGAAGGAACTGAAAGGAAAGTTCCCAGATGTACCAGGATTCTCCTGGGTGACTCCCTGCATATCTGCCAAAGACATCGTGTACATTGGCTTGCGAGACGTAGACCCTGGGGAACACTATATAATAAAAACTCTGGGAATTAAGTATTTCTCCATGACTGAAGTAGACAAGCTGGGGATTGGCAAGGTGATGGAAGAGACCTTCAGCTACCTGCTGGGAAGGAAGAAAAGGCCGATTCACCTGAGCTTTGATGTCGACGGGCTGGACCCAGCATTCACCCCGGCGACCGGCACCCCGGTTCTGGGAGGCCTATCTTACAGAGAAGGTCTCTACATCACAGAAGAAATTTACAAGACAGGGCTCCTTTCAGGACTAGATATCATGGAAGTGAACCCAACTCTTGGGAAGACAGCAGAGGAGGTGAAGAGTACTGTGAACACGGCAGTGGCTTTAACCTTGGCTTGCTTCGGAACTCAACGGGAGGGTAACCATAAGCCAGGGACTGACTACCTTAAACCACCTAAGTGA
