# Supplementary material for: Conditioned culture medium of bone marrow mesenchymal stem cells promotes phenotypic transformation of microglia by regulating mitochondrial autophagy
Source: PeerJ. 2024 Jul 4;12:e17664. doi: 10.7717/peerj.17664 (PMC11227809; doi:10.7717/peerj.17664)
Supplement: Data S1 [file peerj-12-17664-s001.zip › raw data1/1.Rt-qPCR/primer/CD206.docx]

Mus musculus mannose receptor, C type 1 (Mrc1), mRNA

NCBI Reference Sequence: NM_008625.2

GenBank Graphics

>NM_008625.2:100-4470 Mus musculus mannose receptor, C type 1 (Mrc1), mRNA

Forward primer ATGGATTGCCCTGAACAGCA

Reverse primer CTCGTCAGCACCCCAGTTAG

product length 91

ATGAGGCTTCTCCTGCTTCTGGCTTTTATCTCTGTCATCCCTGTCTCTGTTCAGCTATTGGACGCGAGGCAATTTTTAATCTATAATGAAGATCACAAGCGCTGCGTGGACGCTCTAAGTGCCATCTCAGTTCAGACGGCAACTTGCAACCCGGAAGCTGAATCCCAGAAATTCCGCTGGGTGTCAGATTCTCAGATCATGAGTGTTGCTTTCAAATTATGTTTGGGAGTGCCATCAAAAACTGACTGGGCTTCCGTCACCCTGTATGCCTGTGATTCGAAAAGTGAATATCAGAAATGGGAGTGTAAGAATGACACACTCTTTGGAATCAAGGGCACAGAGTTATATTTTAATTATGGCAACAGACAAGAGAAGAATATCAAGCTTTACAAAGGTTCGGGATTGTGGAGCAGATGGAAGGTCTATGGAACCACGGATGACCTGTGCTCGAGAGGATATGAAGCCATGTACTCCTTACTGGGCAATGCAAATGGAGCCGTCTGTGCATTTCCATTCAAGTTTGAAAACAAGTGGTATGCAGACTGCACCTCTGCCGGGCGCTCGGACGGATGGCTCTGGTGTGGAACCACCACTGACTACGACAAAGACAAGCTGTTTGGATTTTGTCCATTGCACTTTGAGGGAAGCGAGAGATTATGGAACAAAGATCCACTGACTGGCATTCTTTACCAGATAAACTCCAAGTCTGCTTTAACCTGGCATCAGGCAAGGGCAAGCTGCAAGCAGCAGAATGCTGACCTCCTGAGTGTCACGGAGATCCACGAGCAAATGTACCTCACAGGATTAACCAGTTCCTTGAGCTCGGGACTCTGGATTGGACTCAACAGTCTGAGTGTACGCAGTGGTTGGCAGTGGGCTGGAGGAAGCCCATTCCGGTATCTGAACTGGCTACCAGGAAGTCCATCATCAGAGCCTGGAAAGAGCTGTGTGTCACTAAACCCTGGAAAAAATGCCAAGTGGGAAAATCTGGAATGTGTTCAGAAGCTTGGCTACATTTGTAAAAAGGGAAACAATACCTTGAACCCATTTATCATTCCCTCAGCAAGCGATGTGCCTACCGGCTGCCCTAATCAGTGGTGGCCCTATGCAGGCCACTGCTACAGGATCCATAGGGAAGAGAAGAAGATCCAGAAATATGCTTTGCAAGCTTGTAGGAAGGAGGGTGGGGACCTGGCAAGTATCCACAGCATTGAGGAGTTTGACTTCATCTTCTCCCAGCTCGGATATGAGCCAAATGATGAGCTGTGGATTGGTTTAAATGACATCAAGATTCAGATGTACTTTGAGTGGAGTGATGGAACCCCAGTGACATTTACTAAATGGCTTCCTGGAGAGCCAAGCCATGAGAACAACAGACAGGAGGACTGCGTGGTTATGAAAGGCAAGGATGGATACTGGGCGGACAGAGCCTGTGAGCAACCACTAGGTTACATCTGTAAGATGGTATCACAAAGCCATGCTGTAGTACCGGAGGGTGCAGACAAAGGCTGCCGGAAAGGCTGGAAACGGCATGGGTTTTACTGCTACTTGATTGGATCCACTCTATCCACCTTCACTGATGCAAACCACACATGCACAAATGAAAAGGCTTATTTAACAACAGTTGAAGACAGATATGAACAAGCATTCCTGACTAGTTTGGTTGGATTGAGGCCTGAAAAATATTTTTGGACAGGACTCTCAGATGTTCAAAACAAAGGGACGTTTCGGTGGACTGTGGACGAGCAGGTGCAGTTTACACACTGGAATGCCGACATGCCAGGACGAAAGGCGGGATGTGTTGCCATGAAAACCGGAGTGGCAGGTGGCTTATGGGATGTTTTGAGTTGTGAAGAAAAGGCAAAATTTGTGTGCAAACATTGGGCAGAAGGAGTGACTCGCCCACCAGAGCCCACAACAACTCCTGAACCCAAATGTCCAGAAAACTGGGGTACCACCAGTAAAACCAGCATGTGTTTCAAACTGTATGCAAAAGGAAAGCATGAAAAGAAAACGTGGTTTGAATCTCGAGATTTTTGCAAAGCTATAGGTGGAGAGCTGGCGAGCATCAAGAGTAAAGATGAACAGCAAGTGATTTGGAGGCTGATTACGAGCAGTGGAAGCTACCATGAGCTGTTTTGGTTGGGACTGACCTATGGAAGTCCTTCAGAGGGGTTCACCTGGAGTGATGGTTCTCCCGTTTCCTATGAAAATTGGGCTTACGGTGAACCAAATAATTACCAAAATGTTGAATATTGTGGTGAGCTGAAAGGTGACCCTGGCATGTCCTGGAATGATATCAACTGTGAACACCTCAACAACTGGATTTGTCAGATACAAAAAGGGAAAACACTACTACCTGAGCCCACACCTGCTCCACAAGACAATCCACCAGTTACTGCAGATGGGTGGGTTATTTACAAAGACTACCAGTACTATTTTAGCAAAGAGAAGGAAACCATGGACAACGCGCGAGCATTTTGCAAGAAGAATTTTGGTGATCTTGCTACAATTAAAAGTGAAAGTGAAAAGAAGTTTCTATGGAAATATATAAACAAGAATGGTGGGCAGTCACCATATTTTATTGGCATGTTAATCAGCATGGATAAGAAATTCATTTGGATGGATGGGAGCAAAGTAGATTTTGTGGCTTGGGCTACAGGAGAACCCAACTTTGCAAATGATGATGAAAACTGTGTAACGATGTACACAAATTCAGGGTTCTGGAATGACATCAACTGTGGTTATCCAAATAACTTCATCTGCCAGAGACATAACAGCAGCATCAATGCCACTGCCATGCCTACCACACCCACGACACCAGGTGGCTGCAAGGAAGGTTGGCATTTGTACAAGAACAAGTGCTTTAAAATTTTTGGATTTGCTAATGAAGAAAAAAAAAGCTGGCAAGACGCACGGCAAGCTTGCAAAGGACTGAAAGGAAACCTGGTGTCCATAGAAAATGCACAAGAGCAAGCATTTGTTACCTATCACATGAGAGACTCCACTTTCAATGCCTGGACTGGGCTGAATGATATCAACGCAGAACACATGTTCCTGTGGACAGCTGGACAAGGAGTTCATTATACAAACTGGGGGAAAGGCTATCCTGGTGGAAGAAGAAGTAGCCTATCTTATGAAGATGCTGACTGTGTAGTTGTGATTGGTGGCAATTCACGAGAGGCAGGGACCTGGATGGATGACACCTGTGACAGTAAACAAGGCTATATATGTCAAACACAGACTGACCCTTCCCTGCCTGTTTCTCCAACCACTACTCCAAAAGATGGCTTTGTTACATATGGGAAAAGCAGCTATTCCCTTATGAAATTGAAGCTACCATGGCATGAAGCAGAGACATATTGCAAGGATCATACTTCCCTGCTTGCTAGCATTCTAGACCCCTACAGTAATGCATTTGCATGGATGAAAATGCACCCATTTAATGTACCCATATGGATTGCCCTGAACAGCAACTTGACCAATAATGAATATACTTGGACGGATAGATGGAGGGTGCGGTACACTAACTGGGGTGCTGACGAGCCGAAGCTTAAGTCAGCATGTGTTTACATGGATGTTGATGGCTACTGGAGAACATCATACTGCAATGAAAGTTTTTATTTTCTCTGCAAAAAATCAGACGAAATCCCTGCTACTGAACCTCCTCAACTGCCTGGCAAGTGTCCAGAGTCAGAACAGACTGCGTGGATTCCTTTCTATGGCCATTGCTACTATTTTGAATCTTCTTTTACGAGAAGTTGGGGTCAGGCTTCTCTGGAATGCCTTCGAATGGGTGCCTCCCTGGTTTCCATCGAGACTGCTGCTGAGTCCAGTTTTCTGTCATACCGTGTTGAACCTCTTAAAAGTAAAACCAATTTTTGGATAGGCATGTTCCGAAATGTTGAAGGGAAGTGGCTTTGGTTGAACGACAATCCTGTCTCCTTTGTCAACTGGAAAACAGGCGATCCCTCTGGTGAACGGAATGATTGTGTAGTTCTAGCTTCATCTTCGGGCCTTTGGAATAATATCCACTGTTCTTCGTACAAAGGATTTATTTGTAAAATGCCAAAAATTATTGATCCTGTAACTACACACTCATCCATTACAACCAAAGCTGACCAAAGGAAGATGGATCCTCAACCCAAGGGCTCTTCTAAAGCAGCAGGAGTGGTCACCGTGGTCCTCCTGATTGTGATAGGTGCCGGCGTTGCAGCCTATTTCTTTTATAAGAAAAGGCATGCGTTGCACATACCTCAAGAGGCCACCTTTGAAAACACTCTCTACTTCAACAGTAATCTGAGTCCAGGAACAAGTGACACGAAAGATCTCATGGGCAACATCGAGCAGAATGAGCATGCGATCATTTAG
