# Supplementary material for: Conditioned culture medium of bone marrow mesenchymal stem cells promotes phenotypic transformation of microglia by regulating mitochondrial autophagy
Source: PeerJ. 2024 Jul 4;12:e17664. doi: 10.7717/peerj.17664 (PMC11227809; doi:10.7717/peerj.17664)
Supplement: Data S1 [file peerj-12-17664-s001.zip › raw data1/1.Rt-qPCR/primer/GAPDH for mouse.docx]

Forward：GGGTCCCAGCTTAGGTTCAT

Reverse：TACGGCCAAATCCGTTCACA

Production length: 95 bp

AACCCTTAAGAGGGATGCTGCCCTTACCCCGGGGTCCCAGCTTAGGTTCATCAGGTAAACTCAGGAGAGTGTTTCCTCGTCCCGTAGACAAAATGGTGAAGGTCGGTGTGAACGGATTTGGCCGTATTGGGCGCCTGGTCACCAGGGCTGCCATTTGCAGTGGCAAAGTGGAGATTGTTGCCATCAACGACCCCTTCATTGACCTCAACTACATGGTCTACATGTTCCAGTATGACTCCACTCACGGCAAATTCAACGGCACAGTCAAGGCCGAGAATGGGAAGCTTGTCATCAACGGGAAGCCCATCACCATCTTCCAGGAGCGAGACCCCACTAACATCAAATGGGGTGAGGCCGGTGCTGAGTATGTCGTGGAGTCTACTGGTGTCTTCACCACCATGGAGAAGGCCGGGGCCCACTTGAAGGGTGGAGCCAAAAGGGTCATCATCTCCGCCCCTTCTGCCGATGCCCCCATGTTTGTGATGGGTGTGAACCACGAGAAATATGACAACTCACTCAAGATTGTCAGCAATGCATCCTGCACCACCAACTGCTTAGCCCCCCTGGCCAAGGTCATCCATGACAACTTTGGCATTGTGGAAGGGCTCATGACCACAGTCCATGCCATCACTGCCACCCAGAAGACTGTGGATGGCCCCTCTGGAAAGCTGTGGCGTGATGGCCGTGGGGCTGCCCAGAACATCATCCCTGCATCCACTGGTGCTGCCAAGGCTGTGGGCAAGGTCATCCCAGAGCTGAACGGGAAGCTCACTGGCATGGCCTTCCGTGTTCCTACCCCCAATGTGTCCGTCGTGGATCTGACGTGCCGCCTGGAGAAACCTGCCAAGTATGATGACATCAAGAAGGTGGTGAAGCAGGCATCTGAGGGCCCACTGAAGGGCATCTTGGGCTACACTGAGGACCAGGTTGTCTCCTGCGACTTCAACAGCAACTCCCACTCTTCCACCTTCGATGCCGGGGCTGGCATTGCTCTCAATGACAACTTTGTCAAGCTCATTTCCTGGTATGACAATGAATACGGCTACAGCAACAGGGTGGTGGACCTCATGGCCTACATGGCCTCCAAGGAGTAAGAAACCCTGGACCACCCACCCCAGCAAGGACACTGAGCAAGAGAGGCCCTATCCCAACTCGGCCCCCAACACTGAGCATCTCCCTCACAATTTCCATCCCAGACCCCCATAATAACAGGAGGGGCCTAGGGAGCCCTCCCTACTCTCTTGAATACCATCAATAAAGTTCGCTGCACCCACAAAAAAAAAAAAAAAAAAAAAA
