# Supplementary material for: Conditioned culture medium of bone marrow mesenchymal stem cells promotes phenotypic transformation of microglia by regulating mitochondrial autophagy
Source: PeerJ. 2024 Jul 4;12:e17664. doi: 10.7717/peerj.17664 (PMC11227809; doi:10.7717/peerj.17664)
Supplement: Data S1 [file peerj-12-17664-s001.zip › raw data1/1.Rt-qPCR/primer/iNOS.docx]

Mus musculus nitric oxide synthase 2, inducible (Nos2), transcript variant 2, mRNA

NCBI Reference Sequence: NM_001313921.1

GenBank Graphics

>NM_001313921.1:346-3441 Mus musculus nitric oxide synthase 2, inducible (Nos2), transcript variant 2, mRNA

Forward primer ACGAGACGGATAGGCAGAGA

Reverse primer GCACATGCAAGGAAGGGAAC

product length 120

ATGAACCCCAAGAGTTTGACCAGAGGACCCAGAGACAAGCCTACCCCTCTGGAGGAGCTCCTGCCTCATG

CCATTGAGTTCATCAACCAGTATTATGGCTCCTTTAAAGAGGCAAAAATAGAGGAACATCTGGCCAGGCTGGAAGCTGTAACAAAGGAAATAGAAACAACAGGAACCTACCAGCTCACTCTGGATGAGCTCATCTTTGCCACCAAGATGGCCTGGAGGAATGCCCCTCGCTGCATCGGCAGGATCCAGTGGTCCAACCTGCAGGTCTTTGACGCTCGGAACTGTAGCACAGCACAGGAAATGTTTCAGCACATCTGCAGACACATACTTTATGCCACCAACAATGGCAACATCAGGTCGGCCATCACTGTGTTCCCCCAGCGGAGTGACGGCAAACATGACTTCAGGCTCTGGAATTCACAGCTCATCCGGTACGCTGGCTACCAGATGCCCGATGGCACCATCAGAGGGGATGCTGCCACCTTGGAGTTCACCCAGTTGTGCATCGACCTAGGCTGGAAGCCCCGCTATGGCCGCTTTGATGTGCTGCCTCTGGTCTTGCAAGCTGATGGTCAAGATCCAGAGGTCTTTGAAATCCCTCCTGATCTTGTGTTGGAGGTGACCATGGAGCATCCCAAGTACGAGTGGTTCCAGGAGCTCGGGTTGAAGTGGTATGCACTGCCTGCCGTGGCCAACATGCTACTGGAGGTGGGTGGCCTCGAATTCCCAGCCTGCCCCTTCAATGGTTGGTACATGGGCACCGAGATTGGAGTTCGAGACTTCTGTGACACACAGCGCTACAACATCCTGGAGGAAGTGGGCCGAAGGATGGGCCTGGAGACCCACACACTGGCCTCCCTCTGGAAAGACCGGGCTGTCACGGAGATCAATGTGGCTGTGCTCCATAGTTTCCAGAAGCAGAATGTGACCATCATGGACCACCACACAGCCTCAGAGTCCTTCATGAAGCACATGCAGAATGAGTACCGGGCCCGTGGAGGCTGCCCGGCAGACTGGATTTGGCTGGTCCCTCCAGTGTCTGGGAGCATCACCCCTGTGTTCCACCAGGAGATGTTGAACTATGTCCTATCTCCATTCTACTACTACCAGATCGAGCCCTGGAAGACCCACATCTGGCAGAATGAGAAGCTGAGGCCCAGGAGGAGAGAGATCCGATTTAGAGTCTTGGTGAAAGTGGTGTTCTTTGCTTCCATGCTAATGCGAAAGGTCATGGCTTCACGGGTCAGAGCCACAGTCCTCTTTGCTACTGAGACAGGGAAGTCTGAAGCACTAGCCAGGGACCTGGCCACCTTGTTCAGCTACGCCTTCAACACCAAGGTTGTCTGCATGGACCAGTATAAGGCAAGCACCTTGGAAGAGGAGCAACTACTGCTGGTGGTGACAAGCACATTTGGGAATGGAGACTGTCCCAGCAATGGGCAGACTCTGAAGAAATCTCTGTTCATGCTTAGAGAACTCAACCACACCTTCAGGTATGCTGTGTTTGGCCTTGGCTCCAGCATGTACCCTCAGTTCTGCGCCTTTGCTCATGACATCGACCAGAAGCTGTCCCACCTGGGAGCCTCTCAGCTTGCCCCAACAGGAGAAGGGGACGAACTCAGTGGGCAGGAGGATGCCTTCCGCAGCTGGGCTGTACAAACCTTCCGGGCAGCCTGTGAGACCTTTGATGTCCGAAGCAAACATCACATTCAGATCCCGAAACGCTTCACTTCCAATGCAACATGGGAGCCACAGCAATATAGGCTCATCCAGAGCCCGGAGCCTTTAGACCTCAACAGAGCCCTCAGCAGCATCCATGCAAAGAACGTGTTTACCATGAGGCTGAAATCCCAGCAGAATCTGCAGAGTGAAAAGTCCAGCCGCACCACCCTCCTCGTTCAGCTCACCTTCGAGGGCAGCCGAGGGCCCAGCTACCTGCCTGGGGAACACCTGGGGATCTTCCCAGGCAACCAGACCGCCCTGGTGCAGGGAATCTTGGAGCGAGTTGTGGATTGTCCTACACCACACCAAACTGTGTGCCTGGAGGTTCTGGATGAGAGCGGCAGCTACTGGGTCAAAGACAAGAGGCTGCCCCCCTGCTCACTCAGCCAAGCCCTCACCTACTTCCTGGACATTACGACCCCTCCCACCCAGCTGCAGCTCCACAAGCTGGCTCGCTTTGCCACGGACGAGACGGATAGGCAGAGATTGGAGGCCTTGTGTCAGCCCTCAGAGTACAATGACTGGAAGTTCAGCAACAACCCCACGTTCCTGGAGGTGCTTGAAGAGTTCCCTTCCTTGCATGTGCCCGCTGCCTTCCTGCTGTCGCAGCTCCCTATCTTGAAGCCCCGCTACTACTCCATCAGCTCCTCCCAGGACCACACCCCCTCGGAGGTTCACCTCACTGTGGCCGTGGTCACCTACCGCACCCGAGATGGTCAGGGTCCCCTGCACCATGGTGTCTGCAGCACTTGGATCAGGAACCTGAAGCCCCAGGACCCAGTGCCCTGCTTTGTGCGAAGTGTCAGTGGCTTCCAGCTCCCTGAGGACCCCTCCCAGCCTTGCATCCTCATTGGGCCTGGTACGGGCATTGCTCCCTTCCGAAGTTTCTGGCAGCAGCGGCTCCATGACTCCCAGCACAAAGGGCTCAAAGGAGGCCGCATGAGCTTGGTGTTTGGGTGCCGGCACCCGGAGGAGGACCACCTCTATCAGGAAGAAATGCAGGAGATGGTCCGCAAGAGAGTGCTGTTCCAGGTGCACACAGGCTACTCCCGGCTGCCCGGCAAACCCAAGGTCTACGTTCAGGACATCCTGCAAAAGCAGCTGGCCAATGAGGTACTCAGCGTGCTCCACGGGGAGCAGGGCCACCTCTACATTTGCGGAGATGTGCGCATGGCTCGGGATGTGGCTACCACATTGAAGAAGCTGGTGGCCACCAAGCTGAACTTGAGCGAGGAGCAGGTGGAAGACTATTTCTTCCAGCTCAAGAGCCAGAAACGTTATCATGAAGATATCTTCGGTGCAGTCTTTTCCTATGGGGCAAAAAAGGGCAGCGCCTTGGAGGAGCCCAAAGCCACGAGGCTCTGA
