# Supplementary material for: Conditioned culture medium of bone marrow mesenchymal stem cells promotes phenotypic transformation of microglia by regulating mitochondrial autophagy
Source: PeerJ. 2024 Jul 4;12:e17664. doi: 10.7717/peerj.17664 (PMC11227809; doi:10.7717/peerj.17664)
Supplement: Data S1 [file peerj-12-17664-s001.zip › raw data1/1.Rt-qPCR/primer/P2RY12.docx]

Mus musculus purinergic receptor P2Y, G-protein coupled 12 (P2ry12), transcript variant 2, mRNA

NCBI Reference Sequence: NM_001357007.1

GenBank Graphics

>NM_001357007.1:380-1423 Mus musculus purinergic receptor P2Y, G-protein coupled 12 (P2ry12), transcript variant 2, mRNA

Forward primer CCCTGTGCGTCAGAGACTAC

Reverse primer CAAGCTGTTCGTGATGAGCC

product length 92

ATGGATGTGCCTGGTGTCAACACCACCTCAGCCAATACCACCTTCTCCCCTGGGACCAGCACCCTGTGCGTCAGAGACTACAAGATCACCCAGGTTCTCTTCCCATTGCTGTACACCGTCCTGTTCTTTGCTGGGCTCATCACGAACAGCTTGGCAATGAGGATTTTCTTTCAGATCCGCAGTAAATCCAACTTCATCATTTTTCTTAAGAACACGGTCATCTCTGATCTACTAATGATTCTAACTTTTCCATTTAAAATTCTTAGTGATGCTAAACTGGGAGCCGGGCCTCTGAGAACCTTGGTGTGCCAAGTTACTTCAGTCACATTTTATTTTACAATGTATATCAGTATATCGTTCCTGGGGTTGATAACCATTGACCGCTACCTGAAGACCACCAGGCCATTTAAAACGTCCAGCCCCAGCAATCTCTTGGGTGCAAAGATTCTTTCTGTTGTCATCTGGGCCTTCATGTTCTTAATTTCACTGCCTAACATGATTCTCACCAACAGGAGGCCAAAAGATAAGGACGTAACAAAATGTTCTTTCTTAAAGTCAGAGTTTGGTCTAGTTTGGCACGAAATAGTCAATTACATCTGCCAAGTCATTTTCTGGATTAATTTTTTAATTGTCATCGTTTGTTATAGCCTCATTACCAAAGAACTCTATCGGTCTTATGTCAGAACAAGGGGTTCAGCCAAAGTTCCCAAGAAAAAGGTAAACGTCAAGGTTTTCATCATCATTGCTGTATTCTTTATTTGCTTTGTTCCCTTCCACTTTGCACGGATTCCCTACACCCTGAGCCAAACTCGGGCCGTCTTTGACTGCAGTGCTGAGAACACCCTGTTCTACGTGAAGGAGAGCACCCTATGGCTGACGTCACTGAACGCCTGCCTTGATCCATTCATCTACTTTTTTCTTTGCAAGTCTTTCAGAAATTCCTTGACAAGCATGCTGAGGTGCTCAAACTCTACATCAACATCTGGGACAAACAAGAAGAAAGGACAAGAAGGTGGCGAACCAAGCGAAGAGACCCCAATGTAG
