# Supplementary material for: Conditioned culture medium of bone marrow mesenchymal stem cells promotes phenotypic transformation of microglia by regulating mitochondrial autophagy
Source: PeerJ. 2024 Jul 4;12:e17664. doi: 10.7717/peerj.17664 (PMC11227809; doi:10.7717/peerj.17664)
Supplement: Data S1 [file peerj-12-17664-s001.zip › raw data1/1.Rt-qPCR/primer/TNF-a┴.docx]

Mus musculus tumor necrosis factor (Tnf), transcript variant 2, mRNA

NCBI Reference Sequence: NM_001278601.1

GenBank Graphics

>NM_001278601.1:168-827 Mus musculus tumor necrosis factor (Tnf), transcript variant 2, mRNA

Forward primer AGGCACTCCCCCAAAAGATG

Reverse primer TGCCACAAGCAGGAATGAGA

product length 83

ATGAGCACAGAAAGCATGATCCGCGACGTGGAACTGGCAGAAGAGGCACTCCCCCAAAAGATGGGGGGCTTCCAGAACTCCAGGCGGTGCCTATGTCTCAGCCTCTTCTCATTCCTGCTTGTGGCAGGGGCCACCACGCTCTTCTGTCTACTGAACTTCGGGGTGATCGGTCCCCAAAGGGATGAGAAGTTCCCAAATGGCCTCCCTCTCATCAGTTCTATGGCCCAGACCCTCACACTCACAAACCACCAAGTGGAGGAGCAGCTGGAGTGGCTGAGCCAGCGCGCCAACGCCCTCCTGGCCAACGGCATGGATCTCAAAGACAACCAACTAGTGGTGCCAGCCGATGGGTTGTACCTTGTCTACTCCCAGGTTCTCTTCAAGGGACAAGGCTGCCCCGACTACGTGCTCCTCACCCACACCGTCAGCCGATTTGCTATCTCATACCAGGAGAAAGTCAACCTCCTCTCTGCCGTCAAGAGCCCCTGCCCCAAGGACACCCCTGAGGGGGCTGAGCTCAAACCCTGGTATGAGCCCATATACCTGGGAGGAGTCTTCCAGCTGGAGAAGGGGGACCAACTCAGCGCTGAGGTCAATCTGCCCAAGTACTTAGACTTTGCGGAGTCCGGGCAGGTCTACTTTGGAGTCATTGCTCTGTGA
