# Supplementary figures and images for: Conditioned culture medium of bone marrow mesenchymal stem cells promotes phenotypic transformation of microglia by regulating mitochondrial autophagy (part 1 of 2)
Source: PeerJ. 2024 Jul 4;12:e17664. doi: 10.7717/peerj.17664 (PMC11227809; doi:10.7717/peerj.17664)

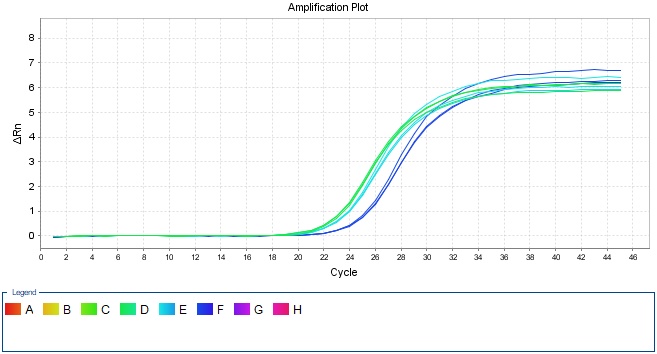

Supplement: Data S1 [file peerj-12-17664-s001.zip › raw data1/1.Rt-qPCR/curve/Arg-1/Linear.jpg]

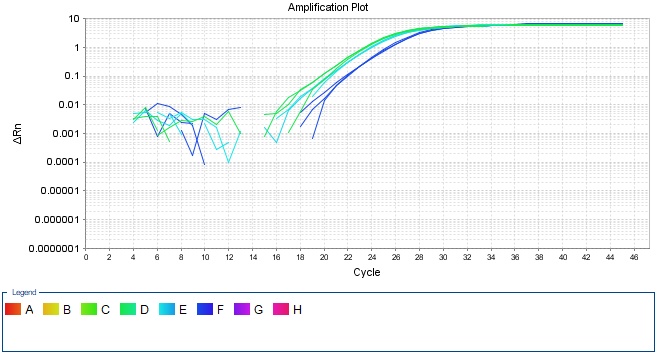

Supplement: Data S1 [file peerj-12-17664-s001.zip › raw data1/1.Rt-qPCR/curve/Arg-1/Log.jpg]

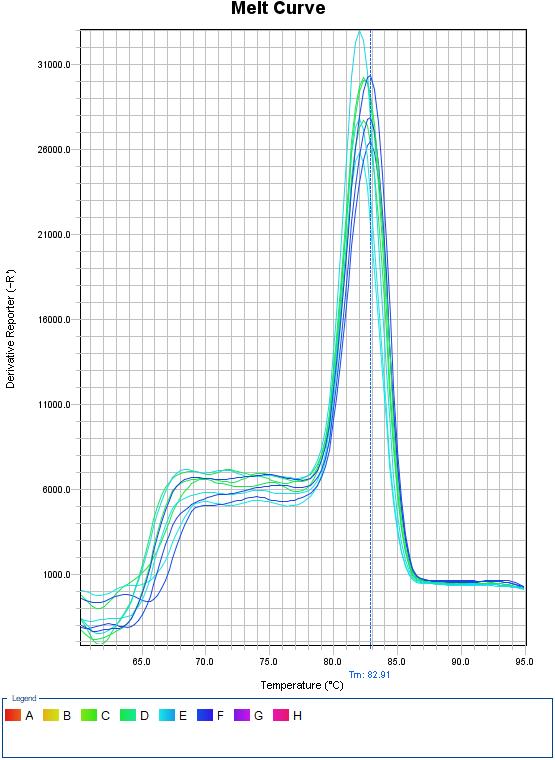

Supplement: Data S1 [file peerj-12-17664-s001.zip › raw data1/1.Rt-qPCR/curve/Arg-1/Melt Curve.jpg]

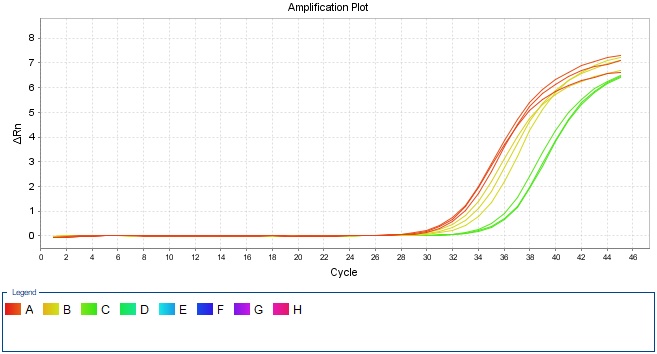

Supplement: Data S1 [file peerj-12-17664-s001.zip › raw data1/1.Rt-qPCR/curve/CD206/Linear.jpg]

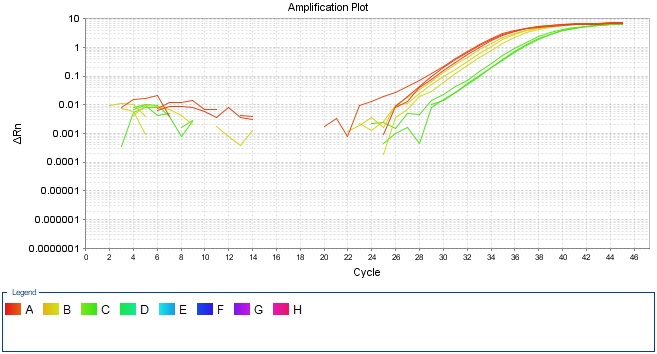

Supplement: Data S1 [file peerj-12-17664-s001.zip › raw data1/1.Rt-qPCR/curve/CD206/Log.jpg]

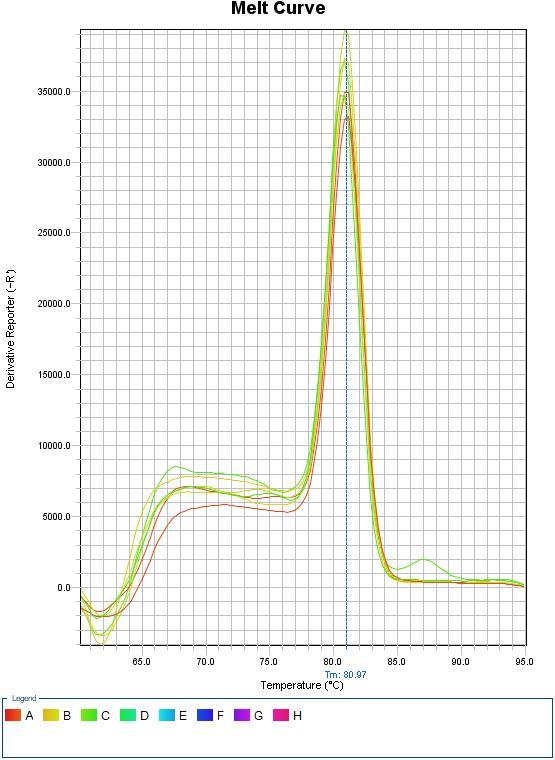

Supplement: Data S1 [file peerj-12-17664-s001.zip › raw data1/1.Rt-qPCR/curve/CD206/Melt Curve.jpg]

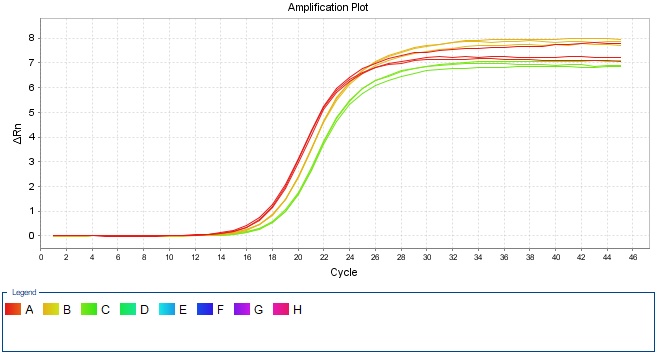

Supplement: Data S1 [file peerj-12-17664-s001.zip › raw data1/1.Rt-qPCR/curve/GAPDH/Linrear.jpg]

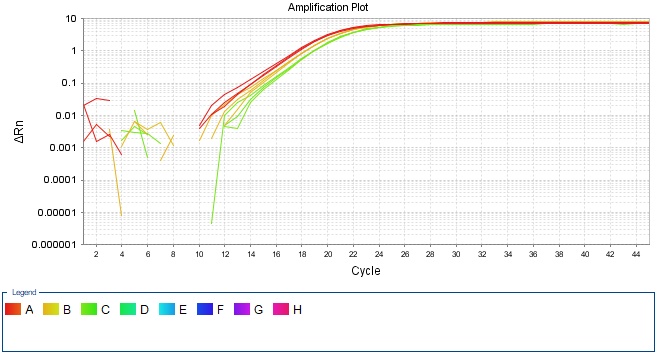

Supplement: Data S1 [file peerj-12-17664-s001.zip › raw data1/1.Rt-qPCR/curve/GAPDH/Log.jpg]

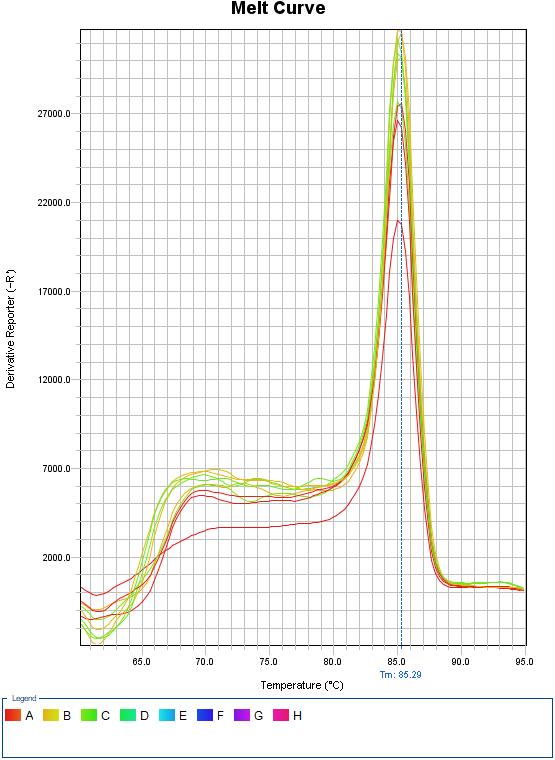

Supplement: Data S1 [file peerj-12-17664-s001.zip › raw data1/1.Rt-qPCR/curve/GAPDH/Melt Curve.jpg]

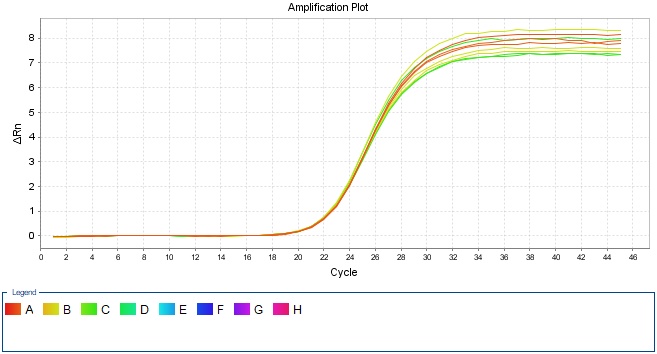

Supplement: Data S1 [file peerj-12-17664-s001.zip › raw data1/1.Rt-qPCR/curve/iNOS/Linear.jpg]

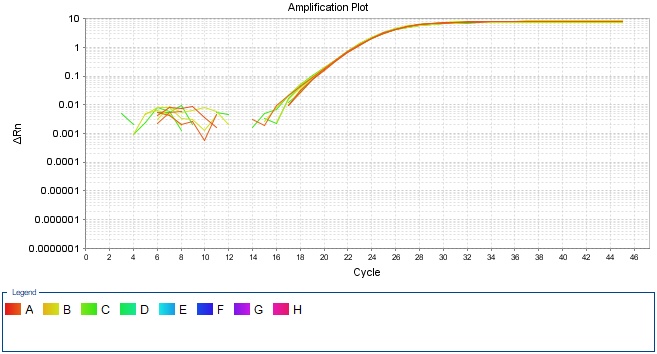

Supplement: Data S1 [file peerj-12-17664-s001.zip › raw data1/1.Rt-qPCR/curve/iNOS/Log.jpg]

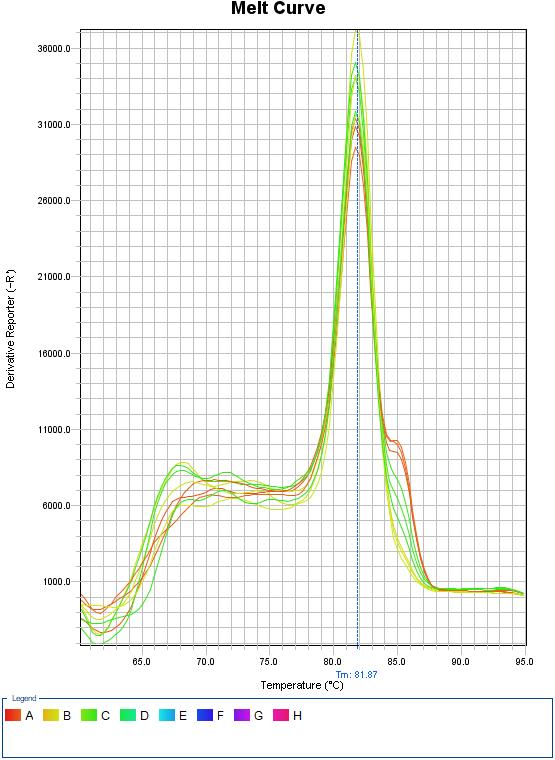

Supplement: Data S1 [file peerj-12-17664-s001.zip › raw data1/1.Rt-qPCR/curve/iNOS/Melt Curve.jpg]

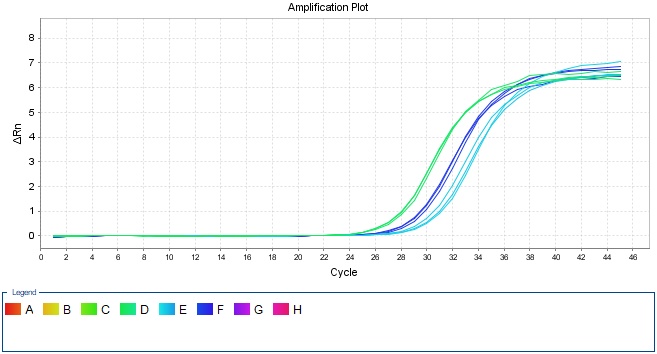

Supplement: Data S1 [file peerj-12-17664-s001.zip › raw data1/1.Rt-qPCR/curve/P2RY12/Linear.jpg]

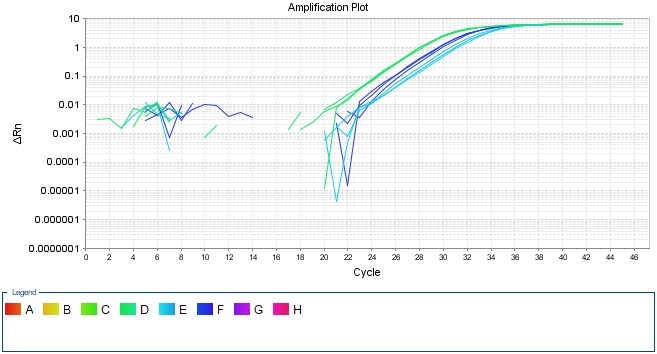

Supplement: Data S1 [file peerj-12-17664-s001.zip › raw data1/1.Rt-qPCR/curve/P2RY12/Log.jpg]

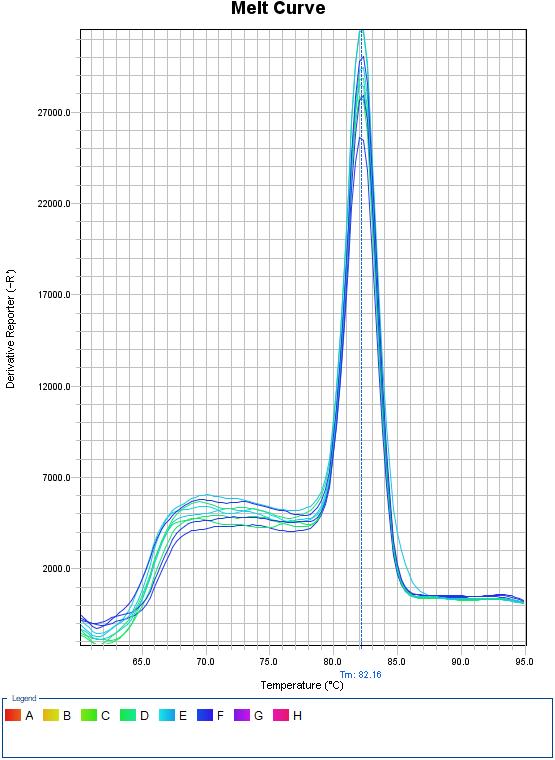

Supplement: Data S1 [file peerj-12-17664-s001.zip › raw data1/1.Rt-qPCR/curve/P2RY12/Melt Curve.jpg]

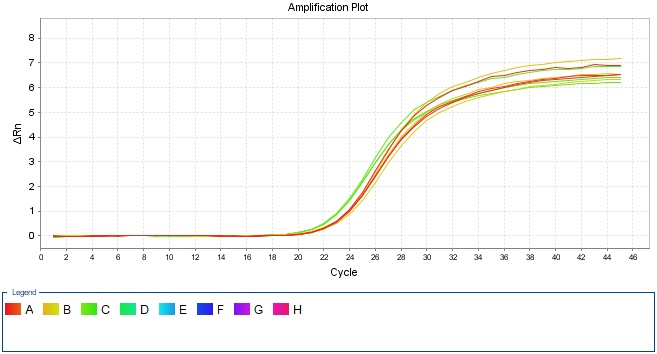

Supplement: Data S1 [file peerj-12-17664-s001.zip › raw data1/1.Rt-qPCR/curve/TNF-a┴/Linear.jpg]

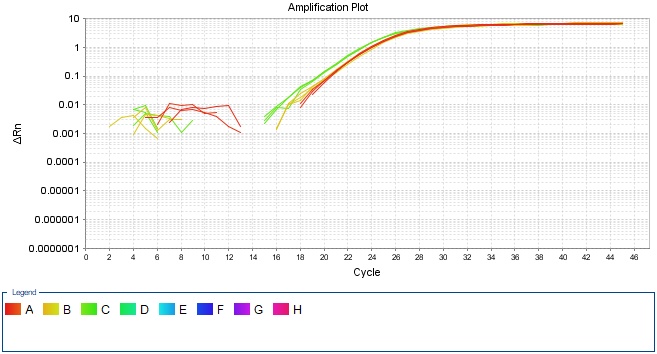

Supplement: Data S1 [file peerj-12-17664-s001.zip › raw data1/1.Rt-qPCR/curve/TNF-a┴/Log.jpg]

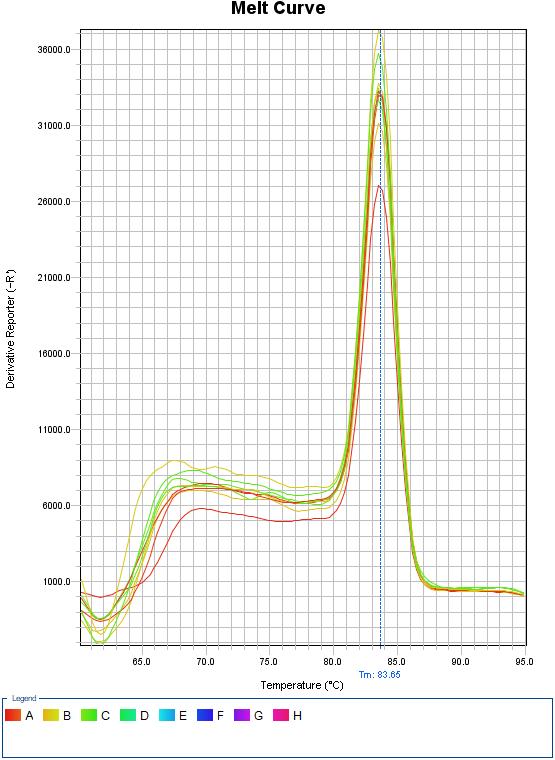

Supplement: Data S1 [file peerj-12-17664-s001.zip › raw data1/1.Rt-qPCR/curve/TNF-a┴/Melt Curve.jpg]

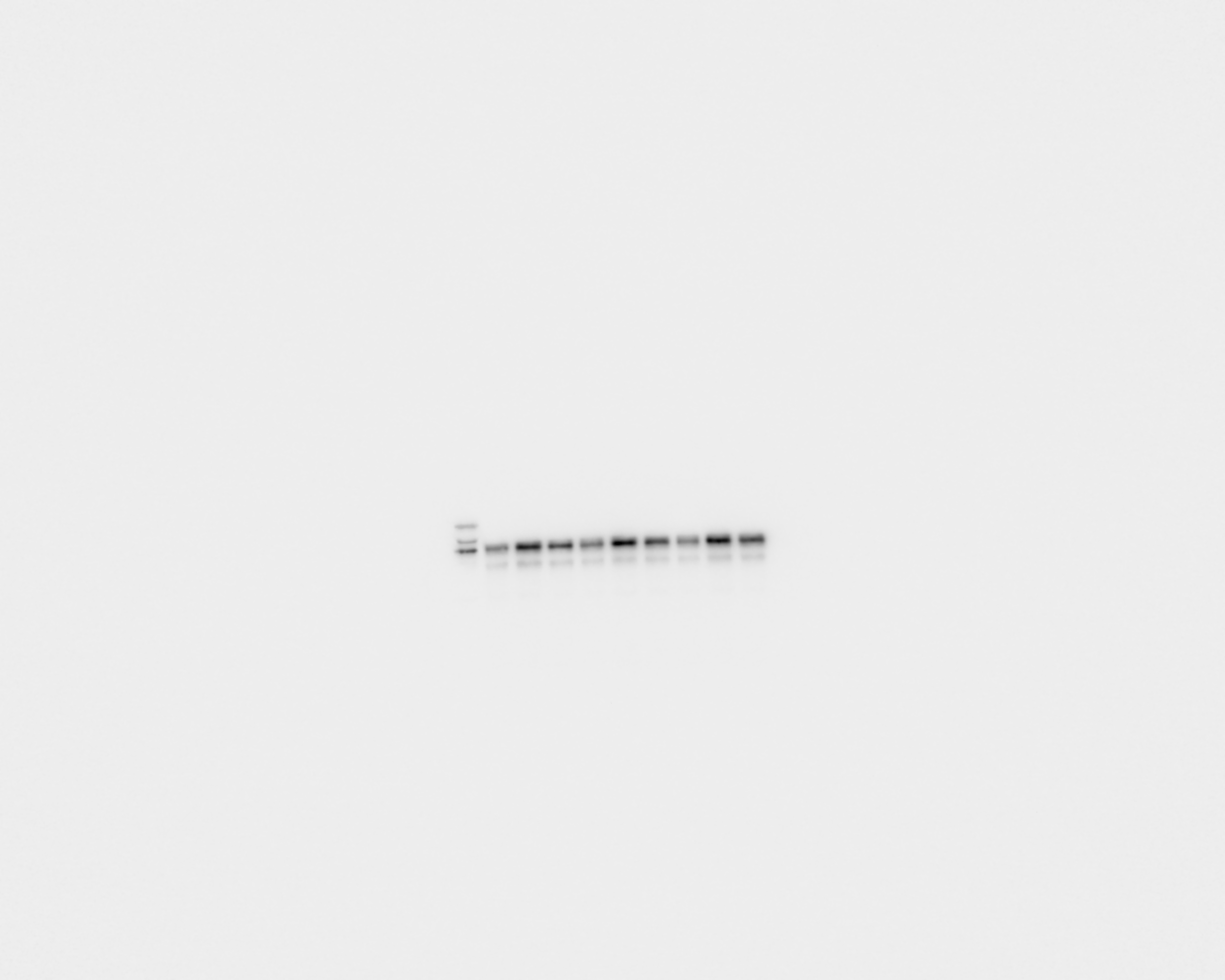

Supplement: Data S1 [file peerj-12-17664-s001.zip › raw data1/4. WB/1 AMPK.tif]

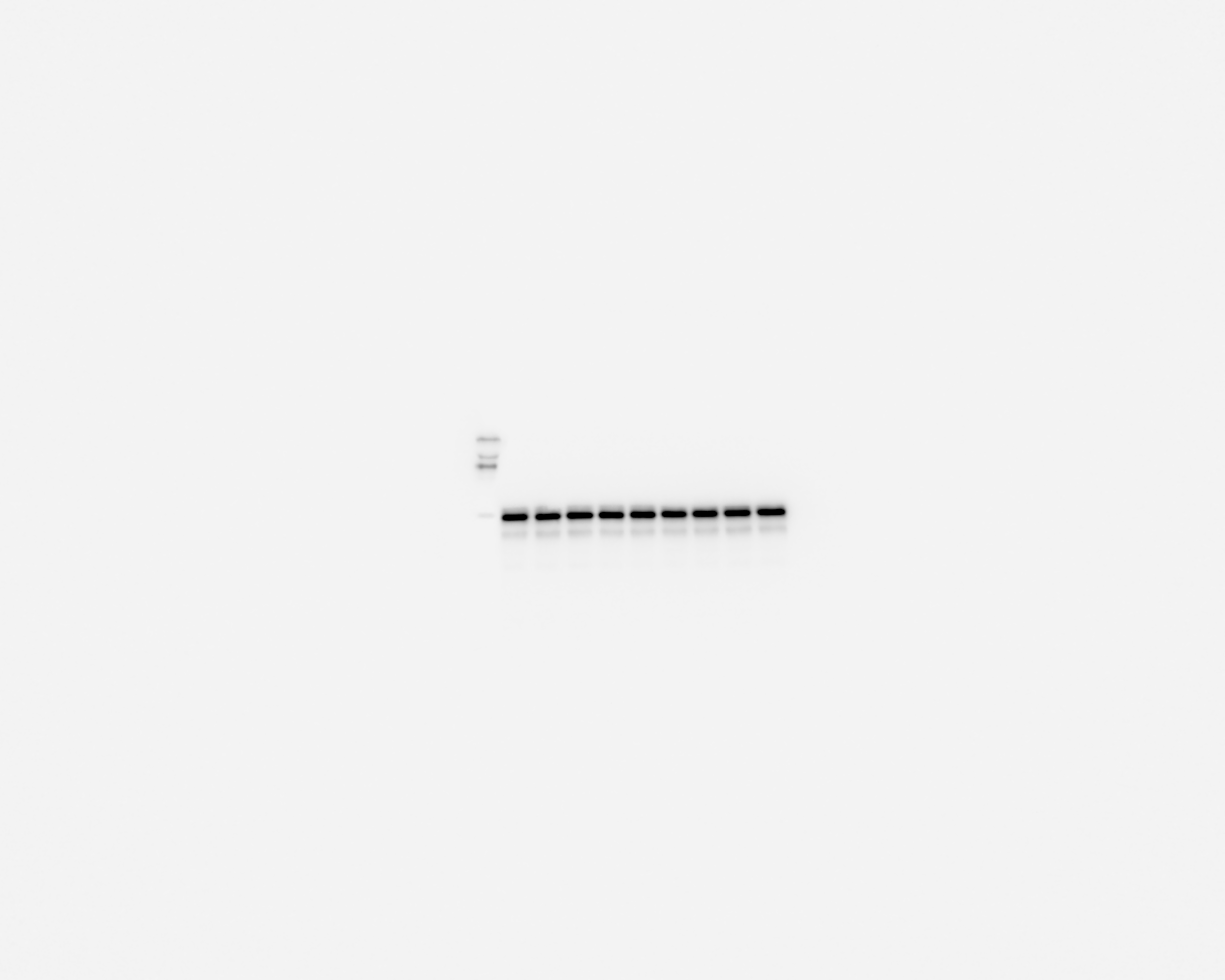

Supplement: Data S1 [file peerj-12-17664-s001.zip › raw data1/4. WB/1 GAPDH.tif]

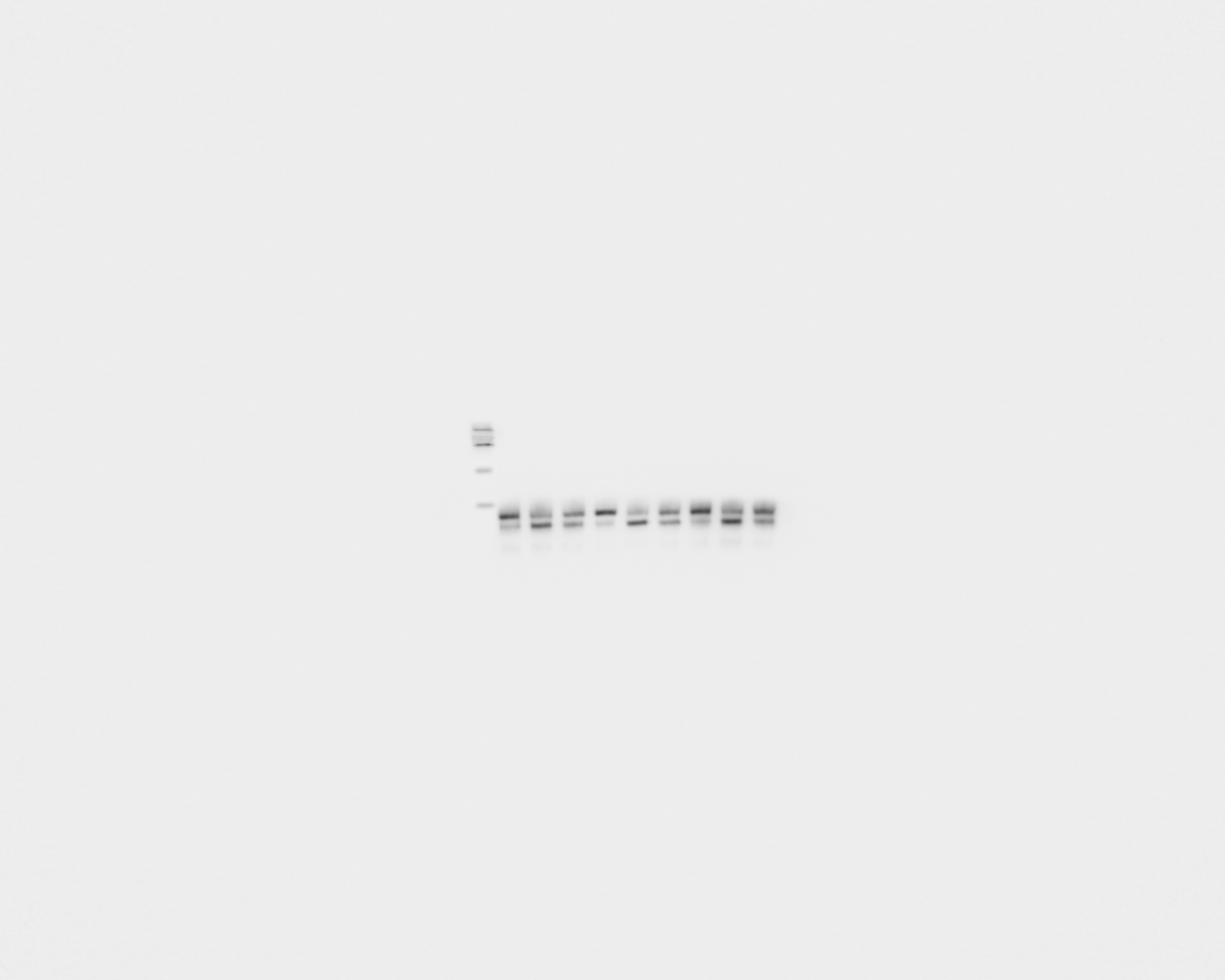

Supplement: Data S1 [file peerj-12-17664-s001.zip › raw data1/4. WB/1 LC3B.tif]

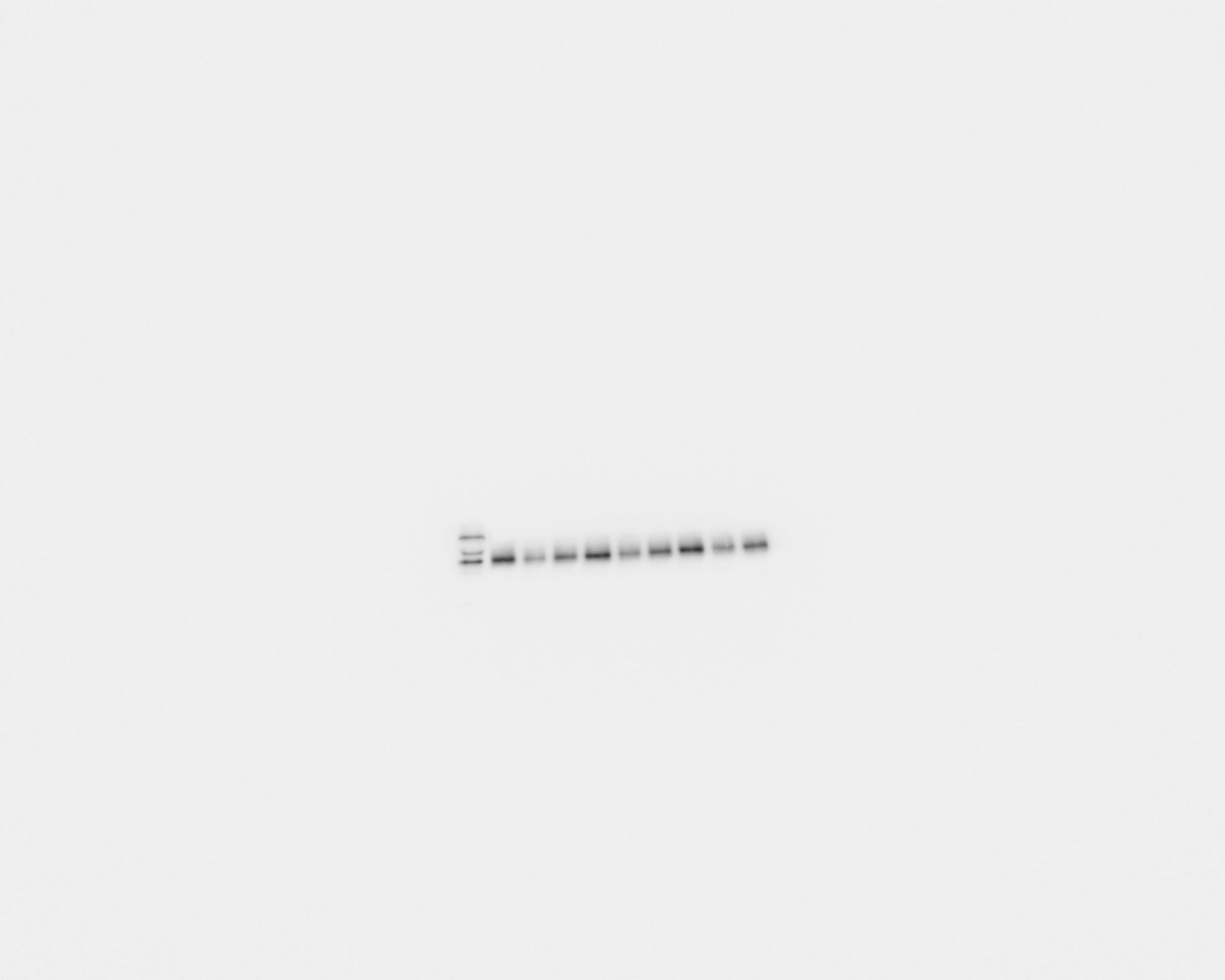

Supplement: Data S1 [file peerj-12-17664-s001.zip › raw data1/4. WB/1 P62.tif]

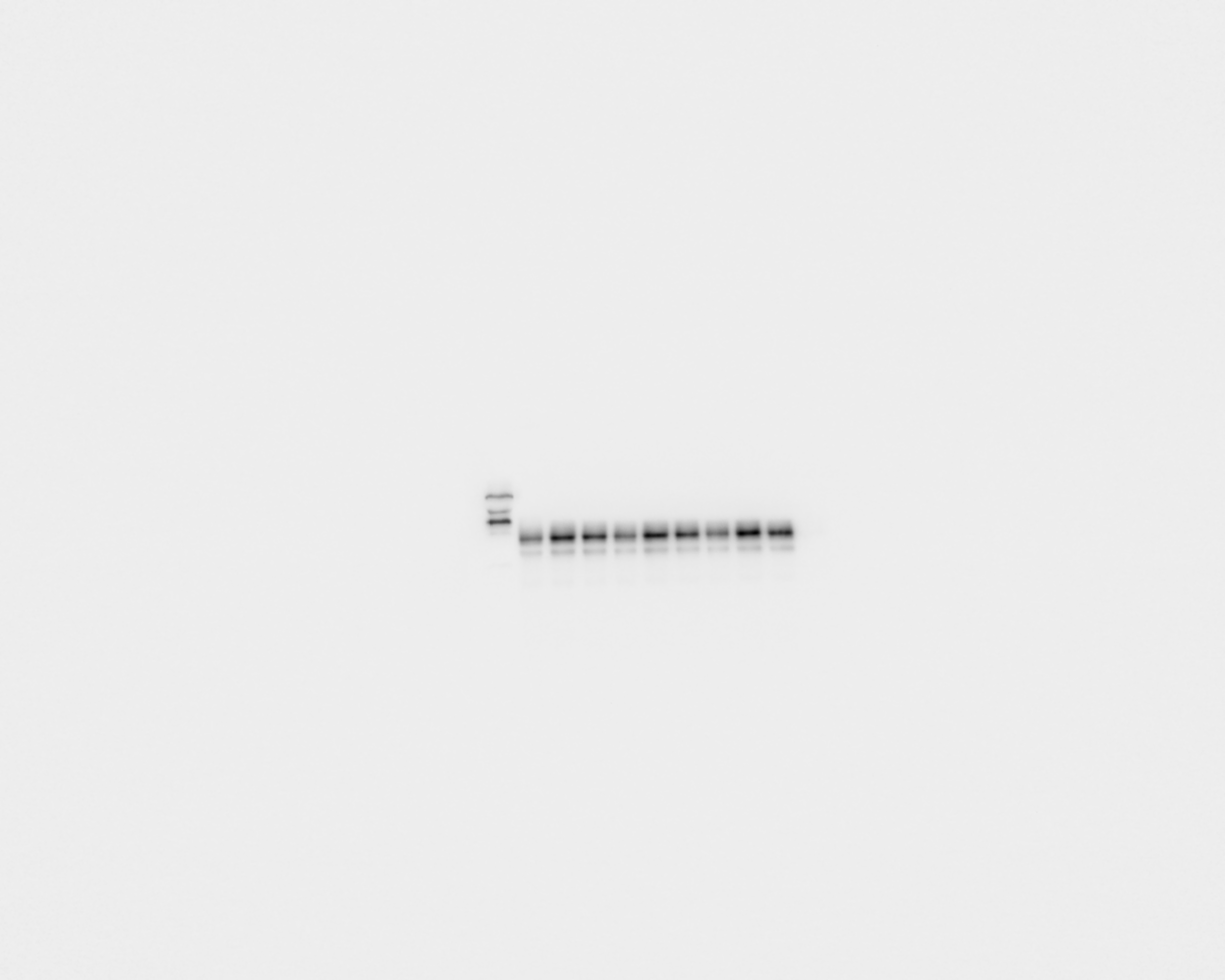

Supplement: Data S1 [file peerj-12-17664-s001.zip › raw data1/4. WB/1 Parkin.tif]

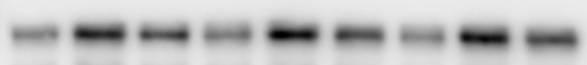

Supplement: Data S1 [file peerj-12-17664-s001.zip › raw data1/4. WB/AMPK.tif]

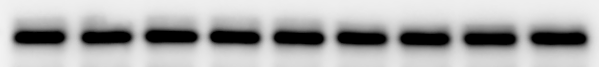

Supplement: Data S1 [file peerj-12-17664-s001.zip › raw data1/4. WB/GAPDH.tif]

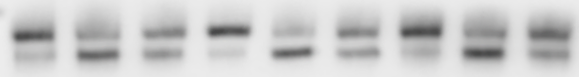

Supplement: Data S1 [file peerj-12-17664-s001.zip › raw data1/4. WB/LC3B.tif]

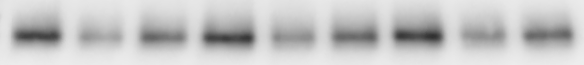

Supplement: Data S1 [file peerj-12-17664-s001.zip › raw data1/4. WB/P62.tif]

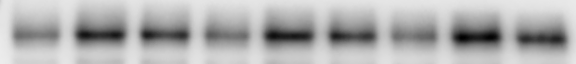

Supplement: Data S1 [file peerj-12-17664-s001.zip › raw data1/4. WB/Parkin.tif]

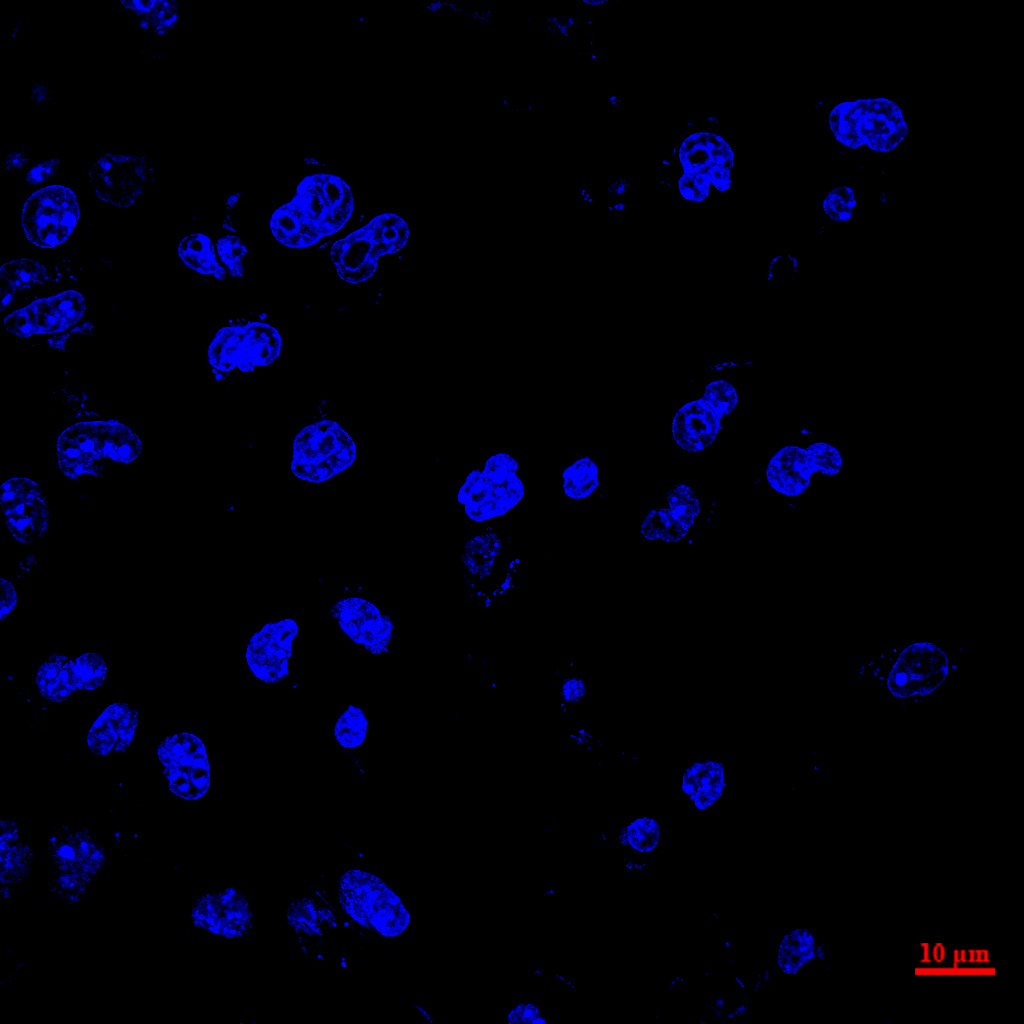

Supplement: Data S2 [file peerj-12-17664-s002.zip › raw data2/double staining+confocal/BV2_0001.tif.frames/BV2_0001_C001T001.tif]

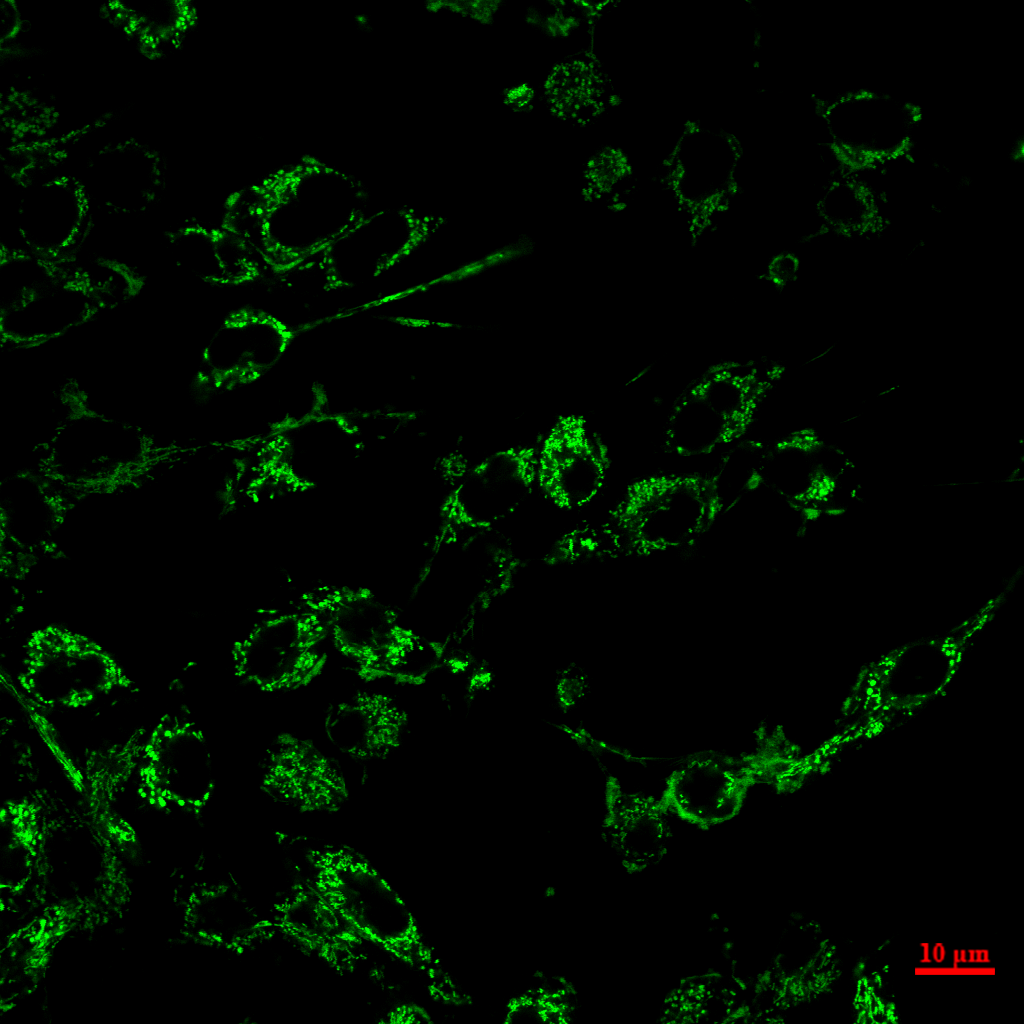

Supplement: Data S2 [file peerj-12-17664-s002.zip › raw data2/double staining+confocal/BV2_0001.tif.frames/BV2_0001_C002T001.tif]

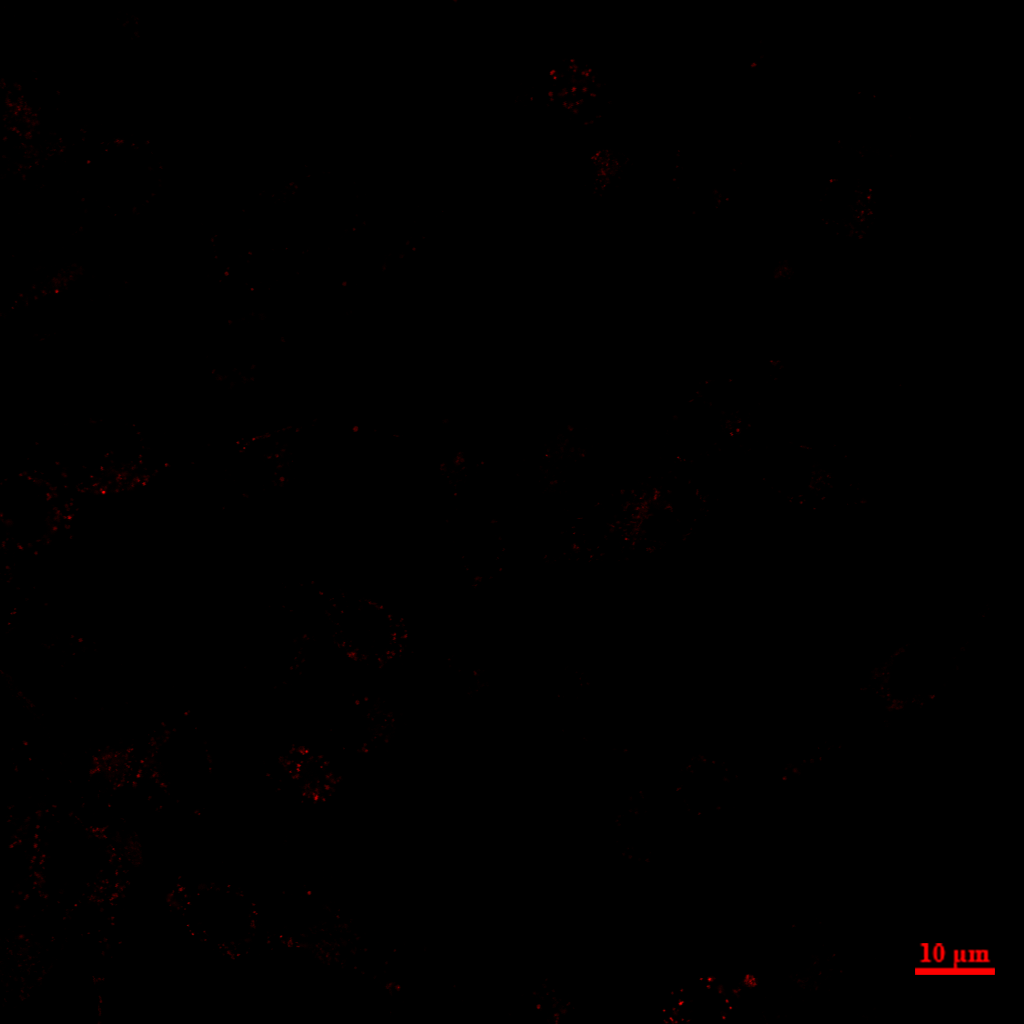

Supplement: Data S2 [file peerj-12-17664-s002.zip › raw data2/double staining+confocal/BV2_0001.tif.frames/BV2_0001_C003T001.tif]

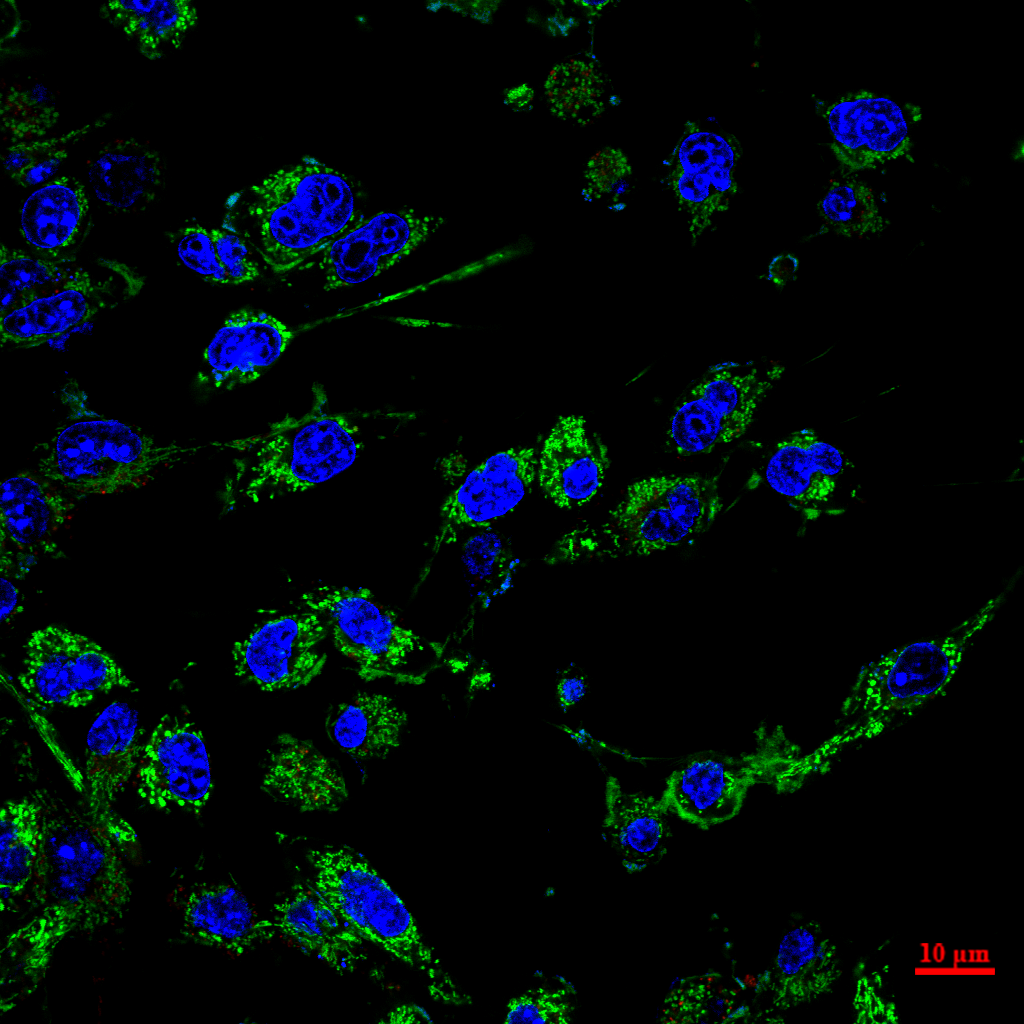

Supplement: Data S2 [file peerj-12-17664-s002.zip › raw data2/double staining+confocal/BV2_0001.tif.frames/BV2_0001_T001.tif]

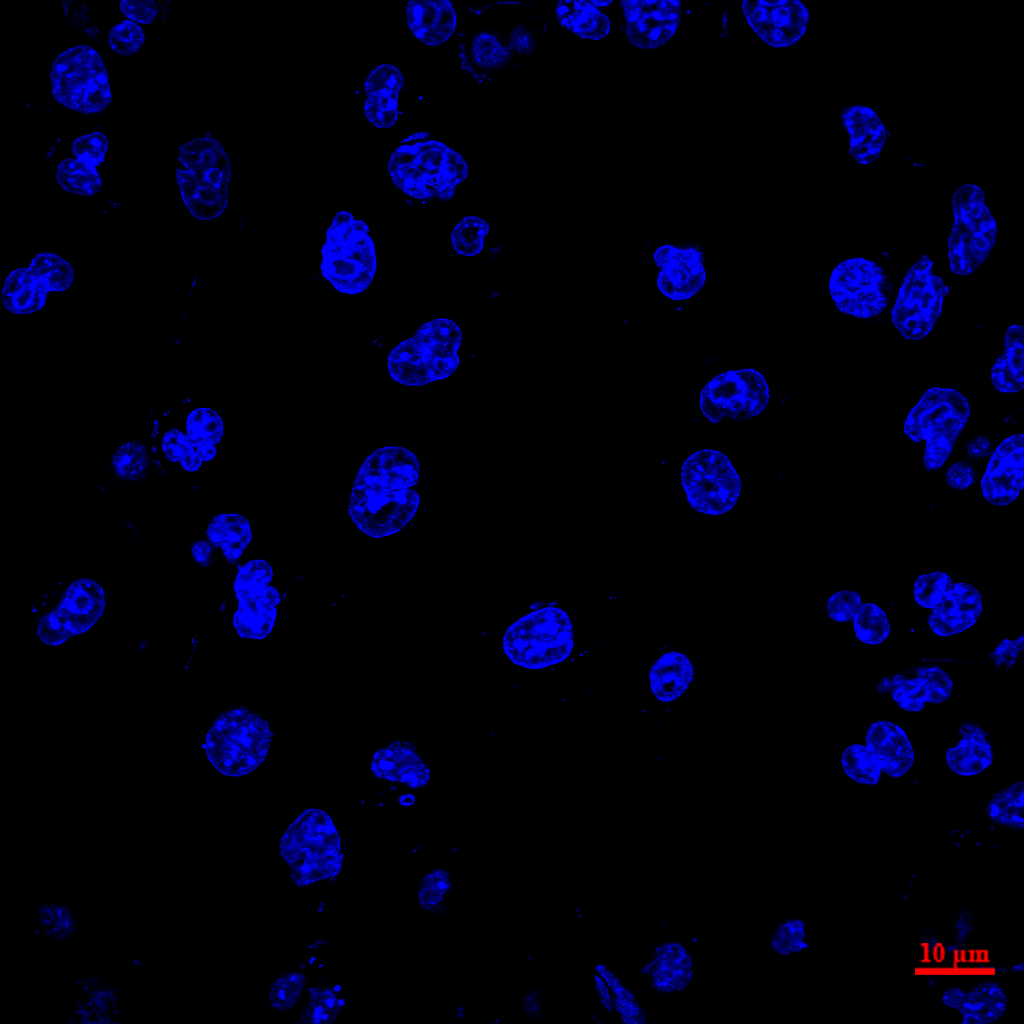

Supplement: Data S2 [file peerj-12-17664-s002.zip › raw data2/double staining+confocal/BV2_0002.tif.frames/BV2_0002_C001T001.tif]

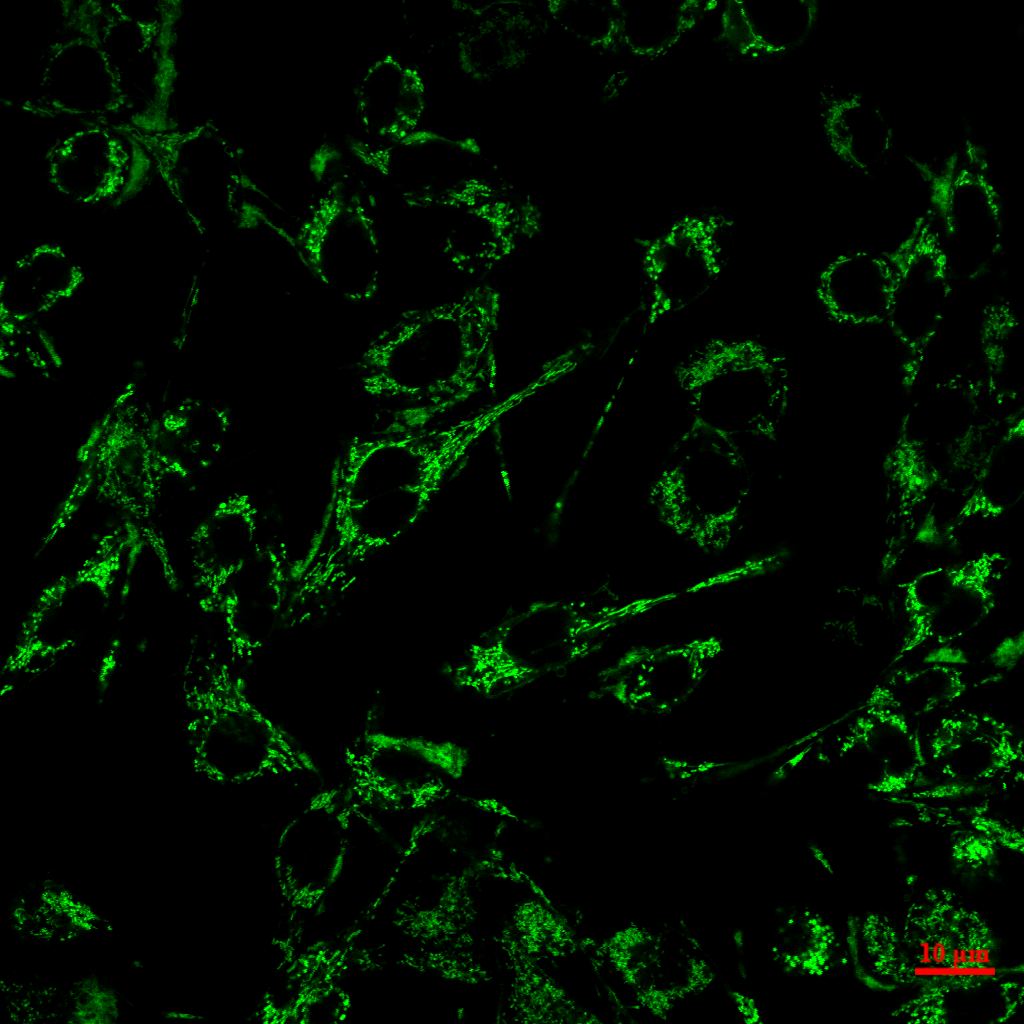

Supplement: Data S2 [file peerj-12-17664-s002.zip › raw data2/double staining+confocal/BV2_0002.tif.frames/BV2_0002_C002T001.tif]

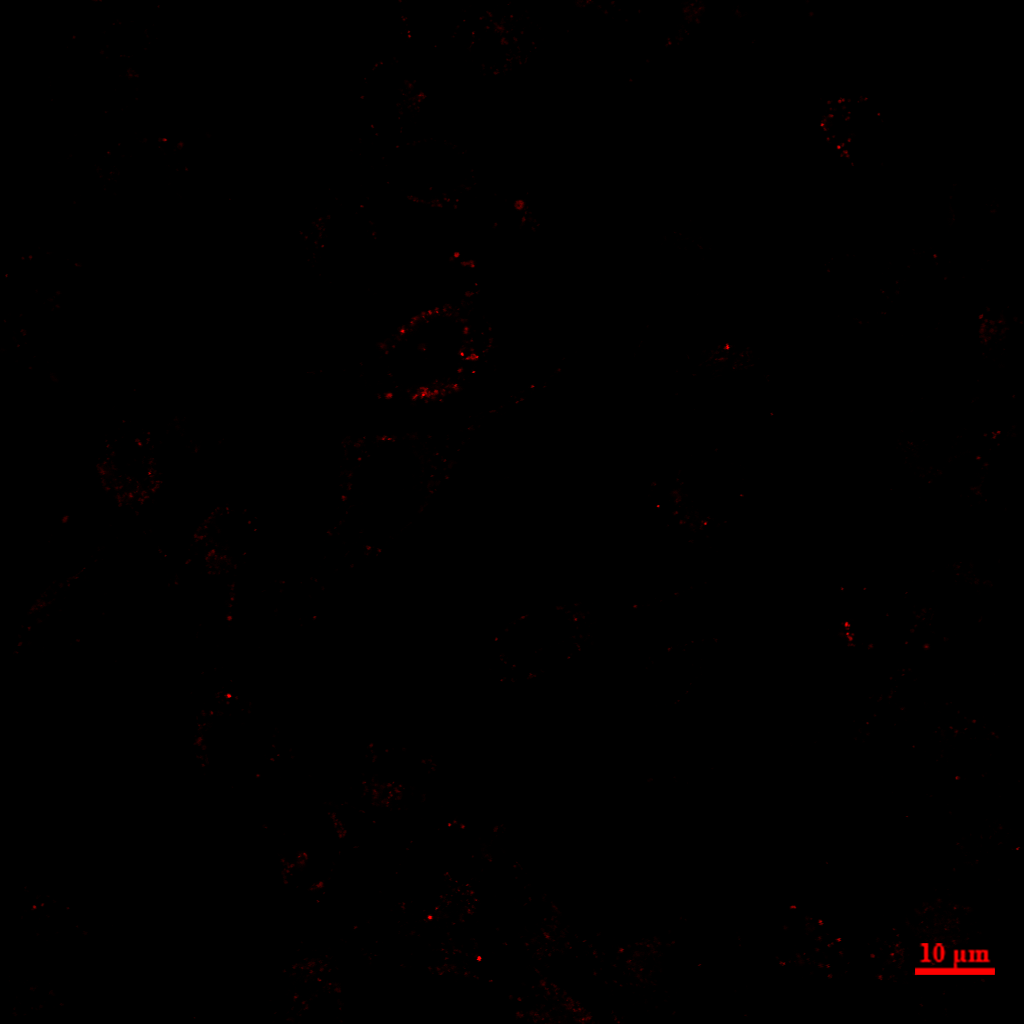

Supplement: Data S2 [file peerj-12-17664-s002.zip › raw data2/double staining+confocal/BV2_0002.tif.frames/BV2_0002_C003T001.tif]

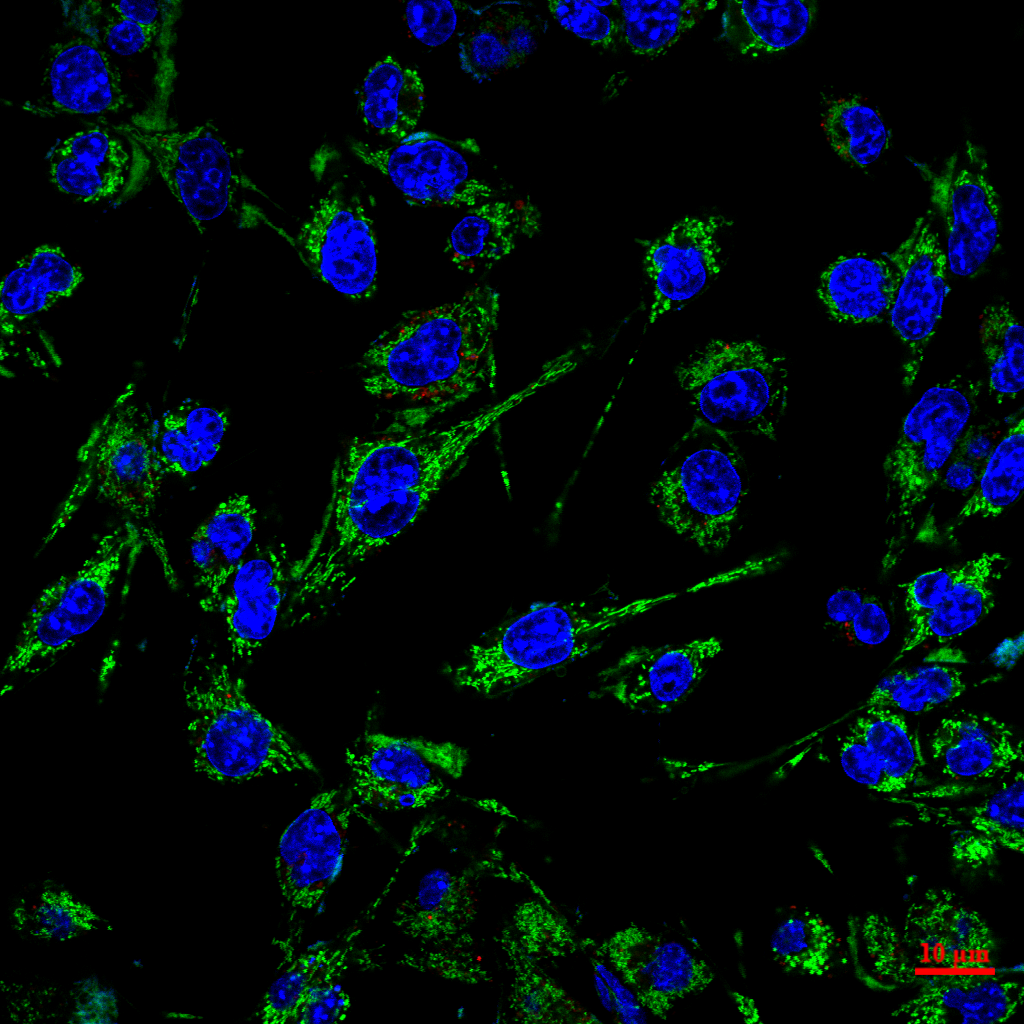

Supplement: Data S2 [file peerj-12-17664-s002.zip › raw data2/double staining+confocal/BV2_0002.tif.frames/BV2_0002_T001.tif]

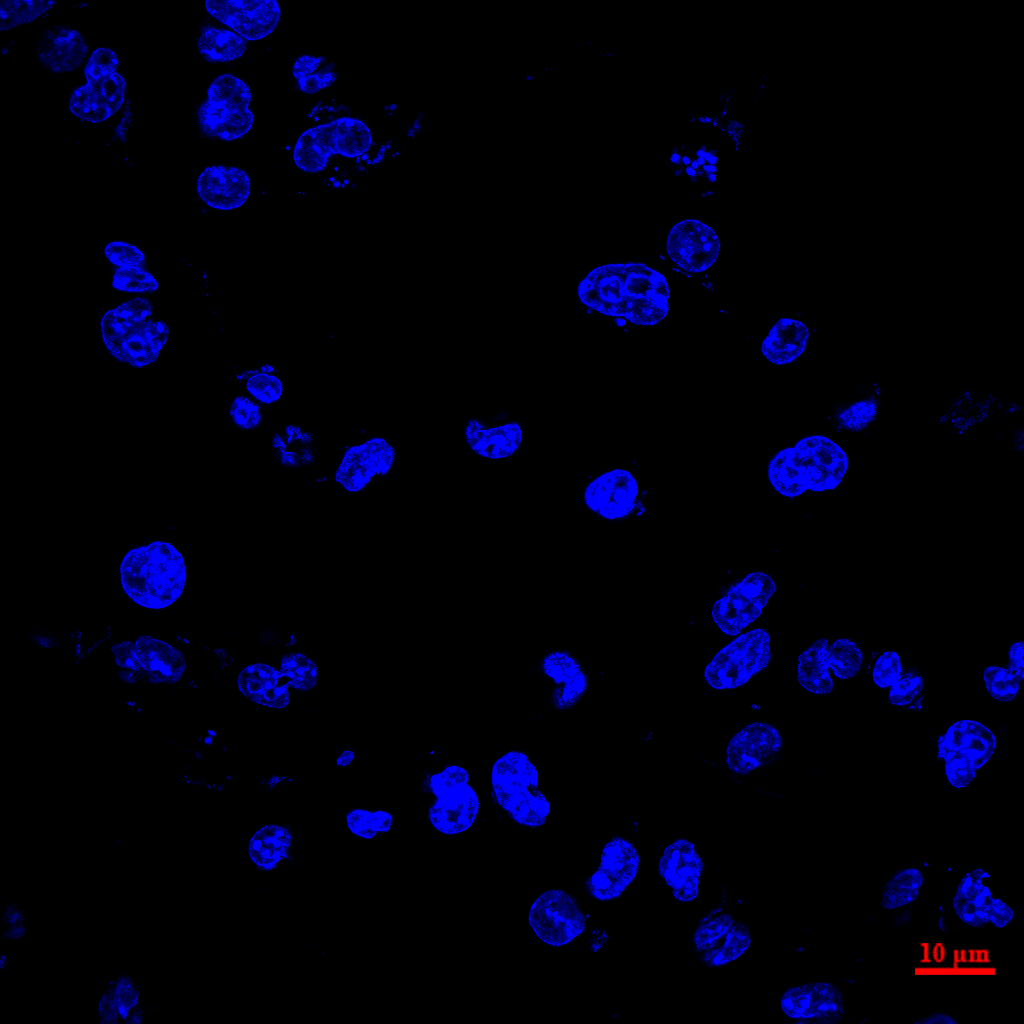

Supplement: Data S2 [file peerj-12-17664-s002.zip › raw data2/double staining+confocal/BV2_0003.tif.frames/BV2_0003_C001T001.tif]

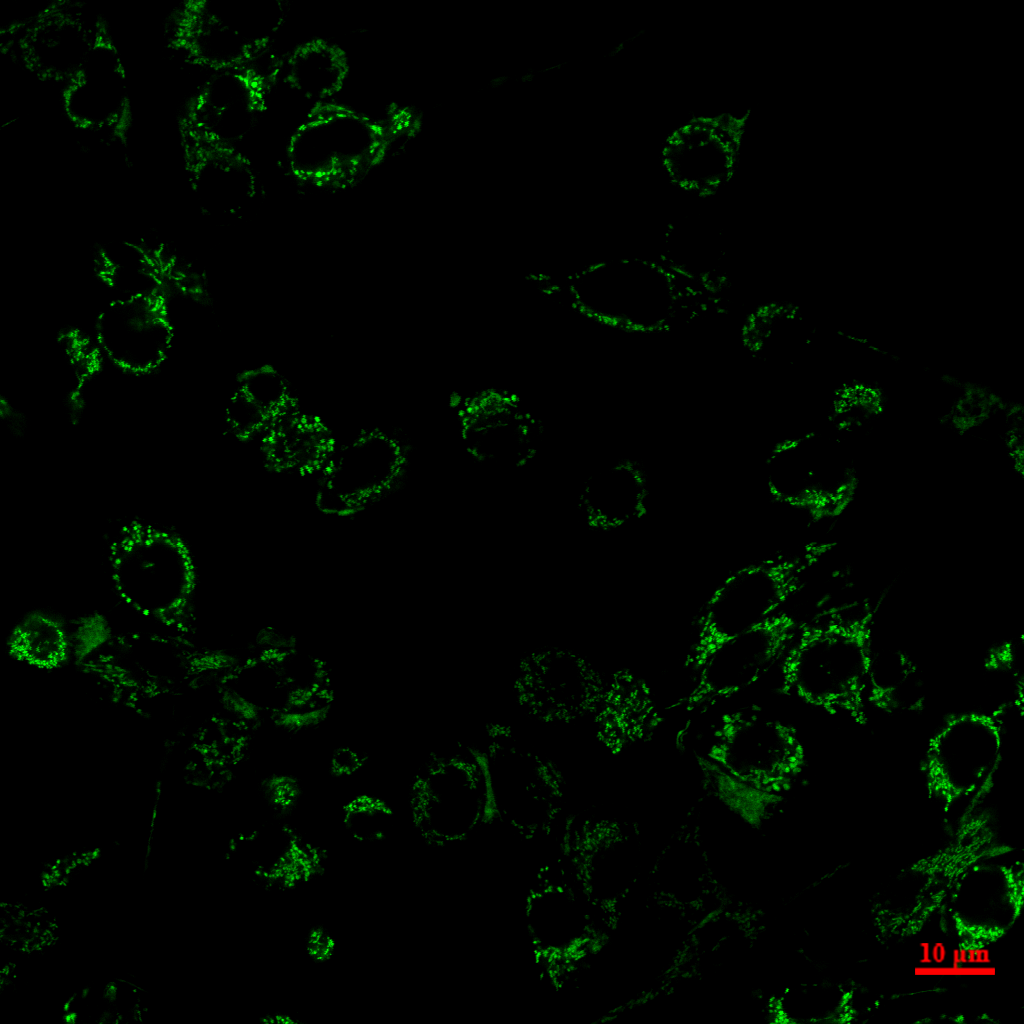

Supplement: Data S2 [file peerj-12-17664-s002.zip › raw data2/double staining+confocal/BV2_0003.tif.frames/BV2_0003_C002T001.tif]

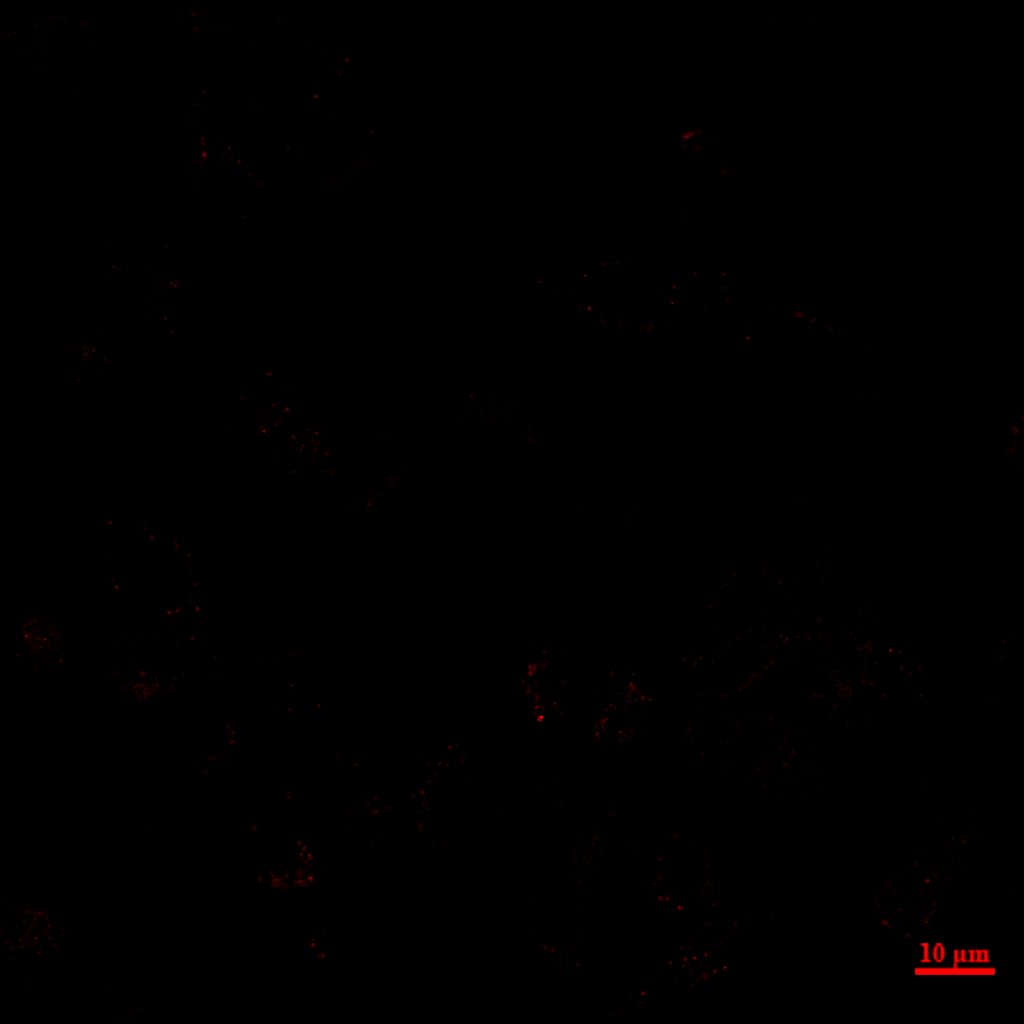

Supplement: Data S2 [file peerj-12-17664-s002.zip › raw data2/double staining+confocal/BV2_0003.tif.frames/BV2_0003_C003T001.tif]

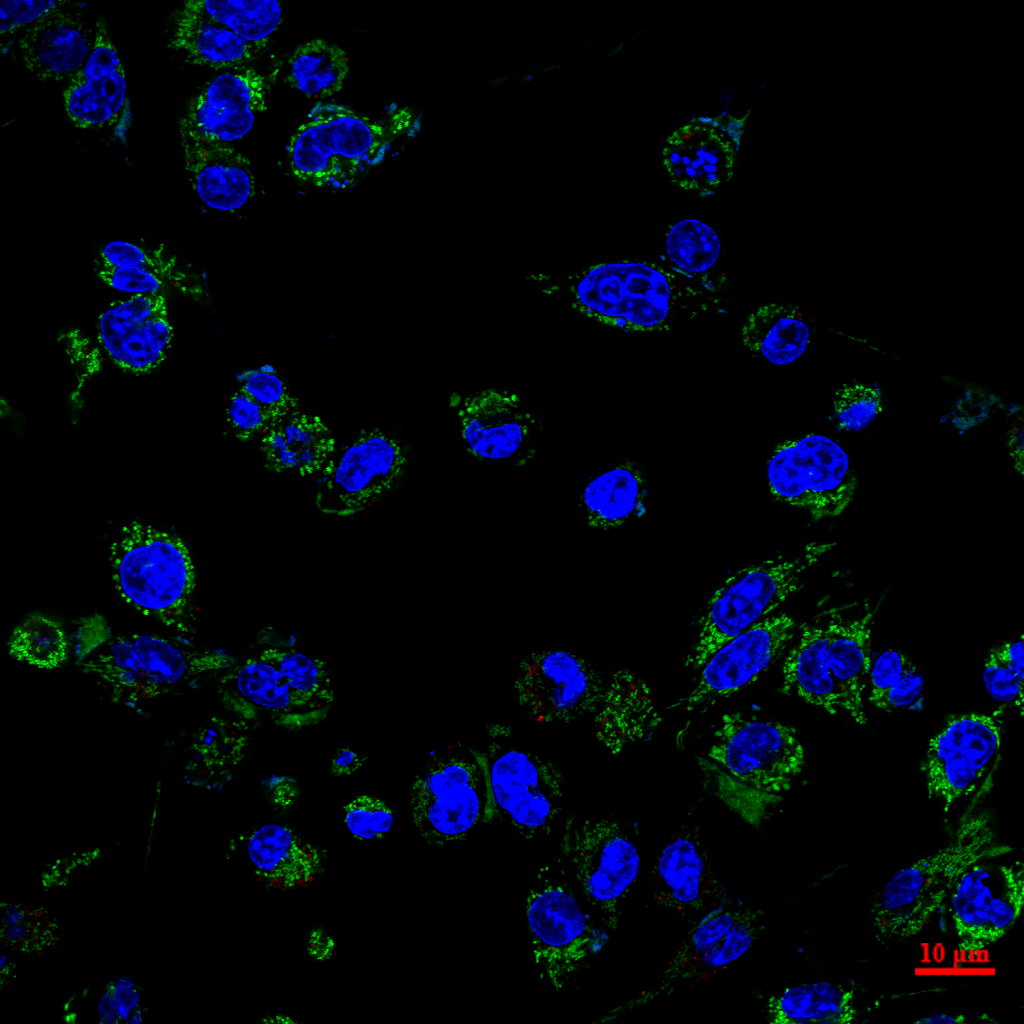

Supplement: Data S2 [file peerj-12-17664-s002.zip › raw data2/double staining+confocal/BV2_0003.tif.frames/BV2_0003_T001.tif]

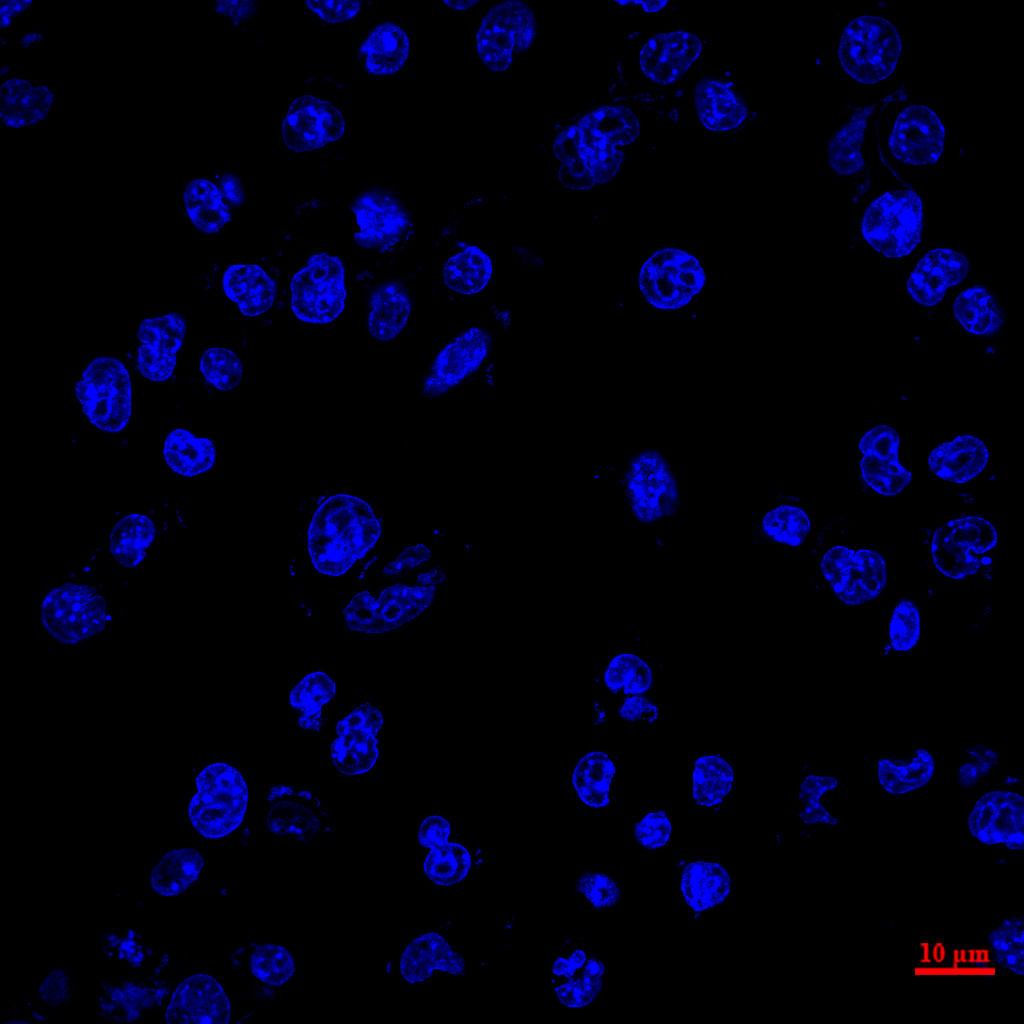

Supplement: Data S2 [file peerj-12-17664-s002.zip › raw data2/double staining+confocal/BV2+LPS_0001.tif.frames/BV2+LPS_0001_C001T001.tif]

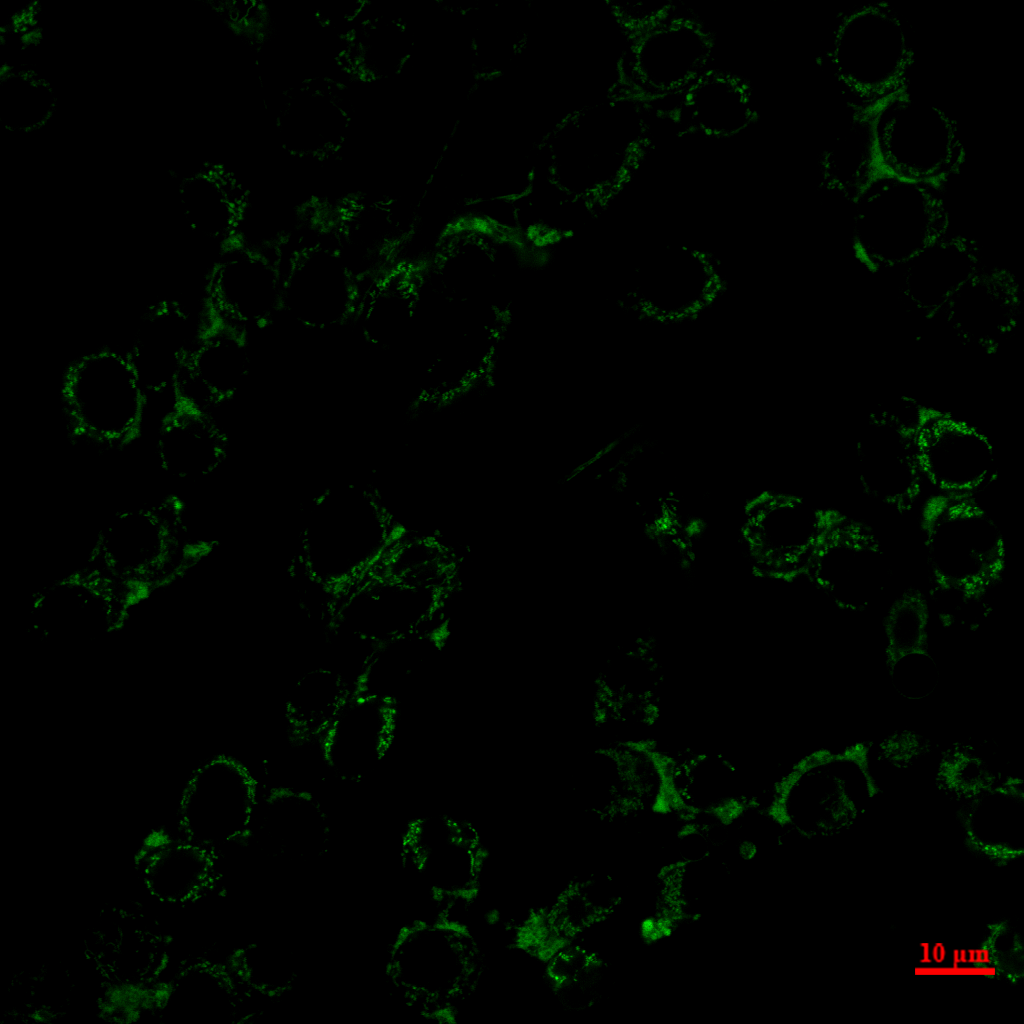

Supplement: Data S2 [file peerj-12-17664-s002.zip › raw data2/double staining+confocal/BV2+LPS_0001.tif.frames/BV2+LPS_0001_C002T001.tif]

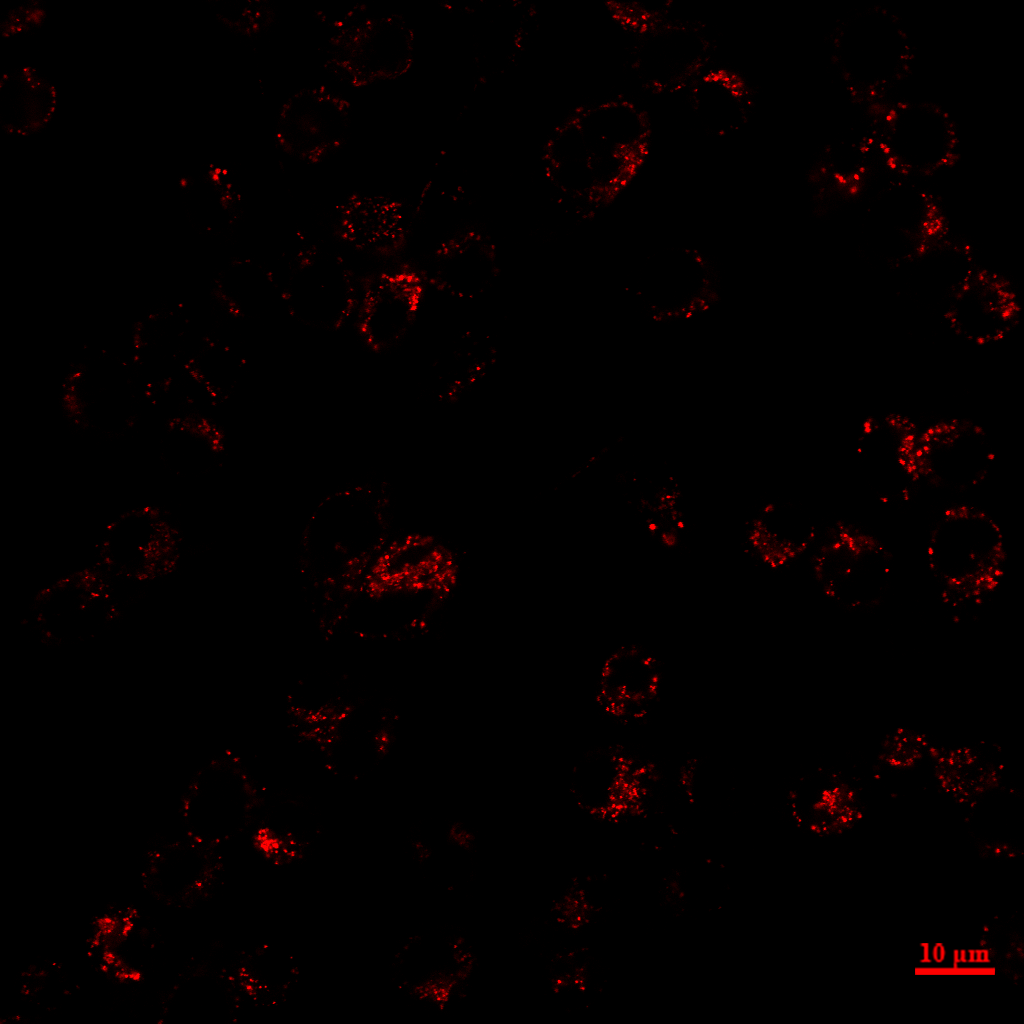

Supplement: Data S2 [file peerj-12-17664-s002.zip › raw data2/double staining+confocal/BV2+LPS_0001.tif.frames/BV2+LPS_0001_C003T001.tif]

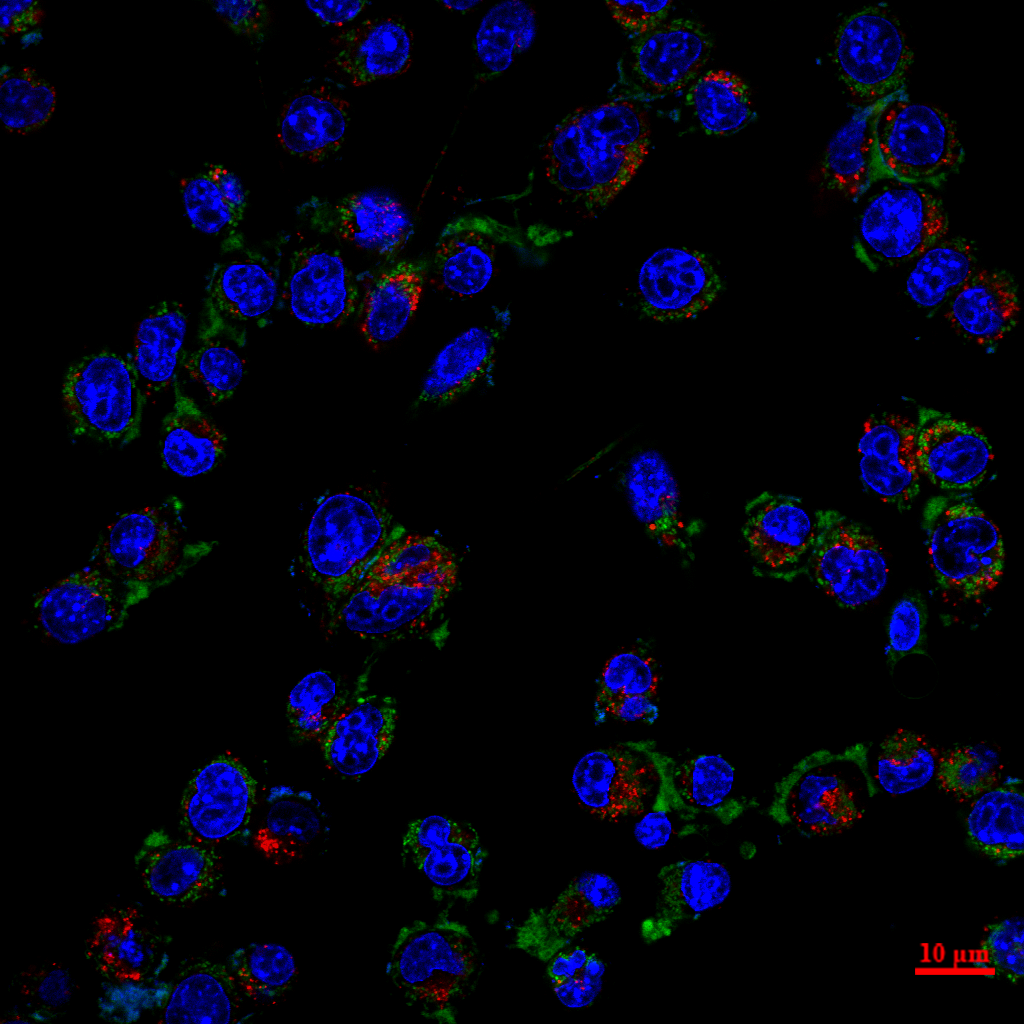

Supplement: Data S2 [file peerj-12-17664-s002.zip › raw data2/double staining+confocal/BV2+LPS_0001.tif.frames/BV2+LPS_0001_T001.tif]

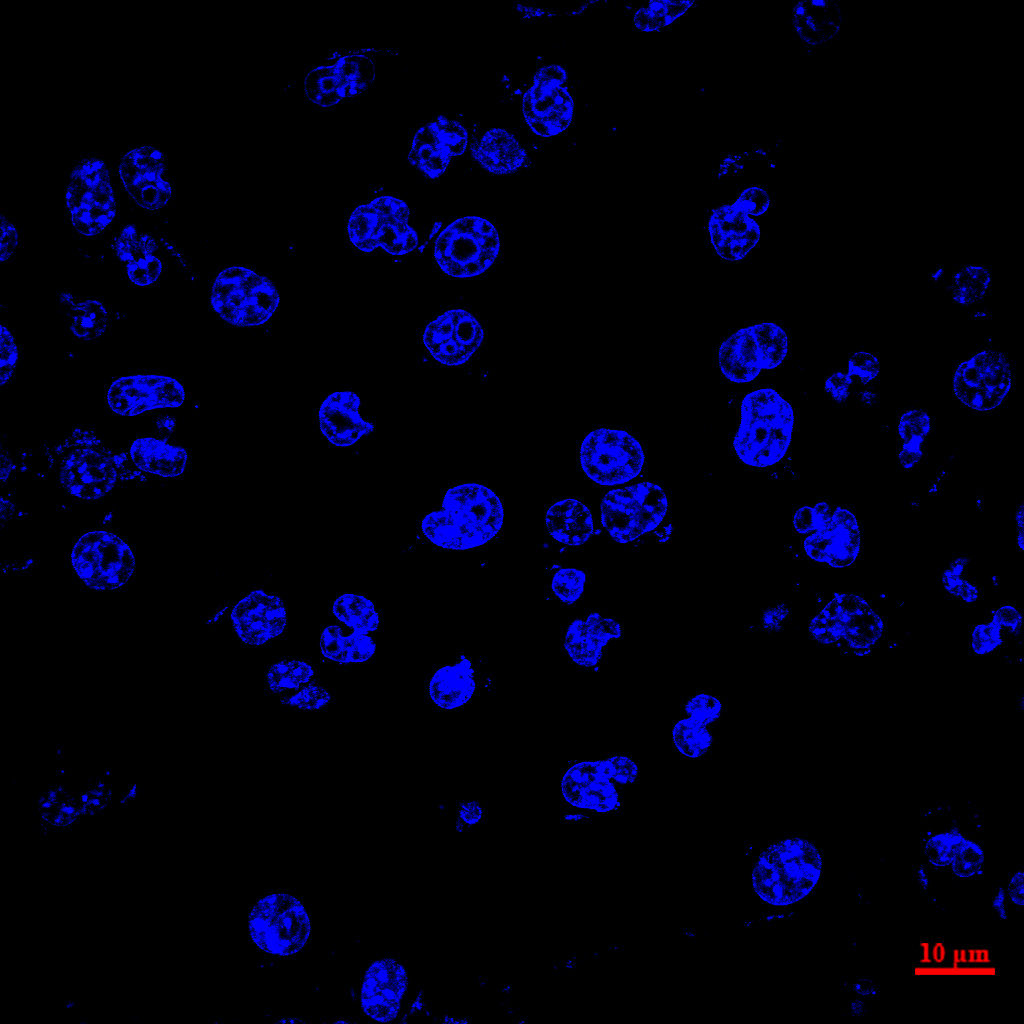

Supplement: Data S2 [file peerj-12-17664-s002.zip › raw data2/double staining+confocal/BV2+LPS_0002.tif.frames/BV2+LPS_0002_C001T001.tif]

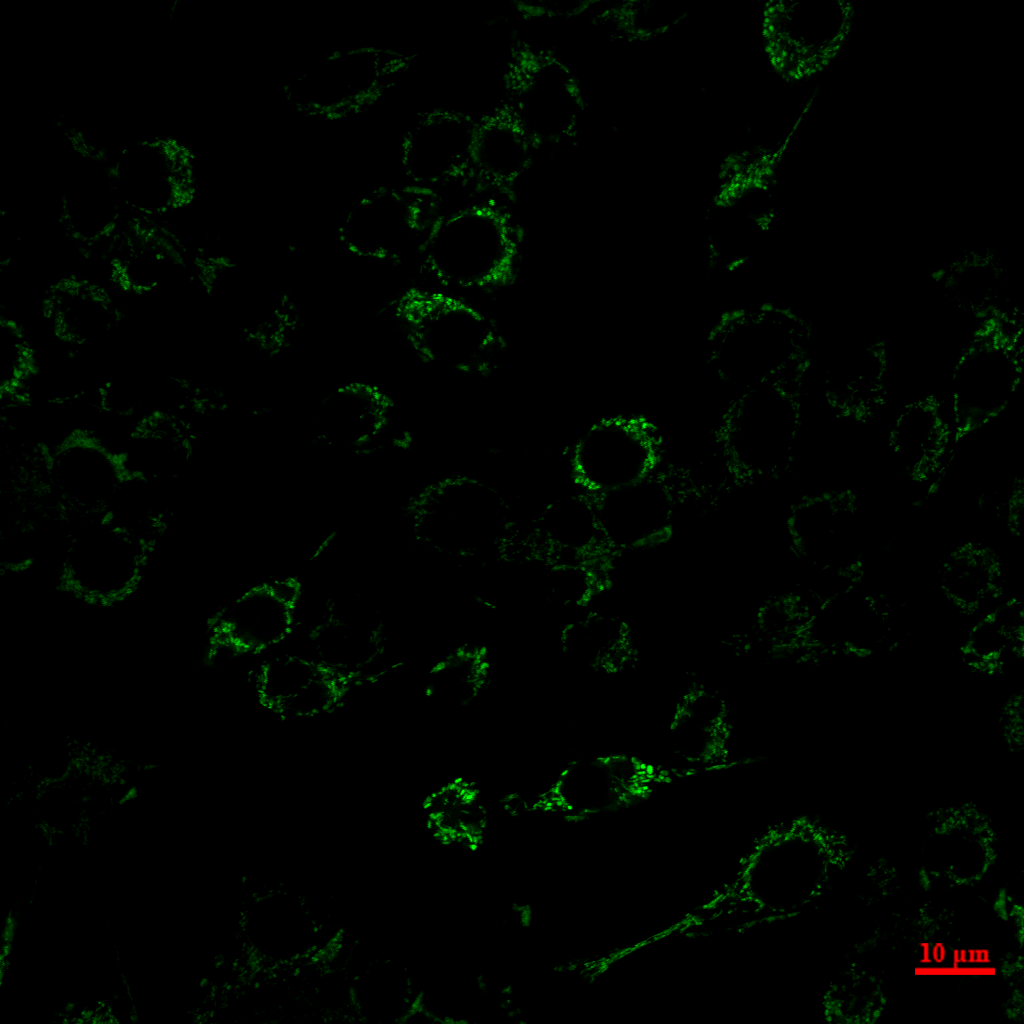

Supplement: Data S2 [file peerj-12-17664-s002.zip › raw data2/double staining+confocal/BV2+LPS_0002.tif.frames/BV2+LPS_0002_C002T001.tif]

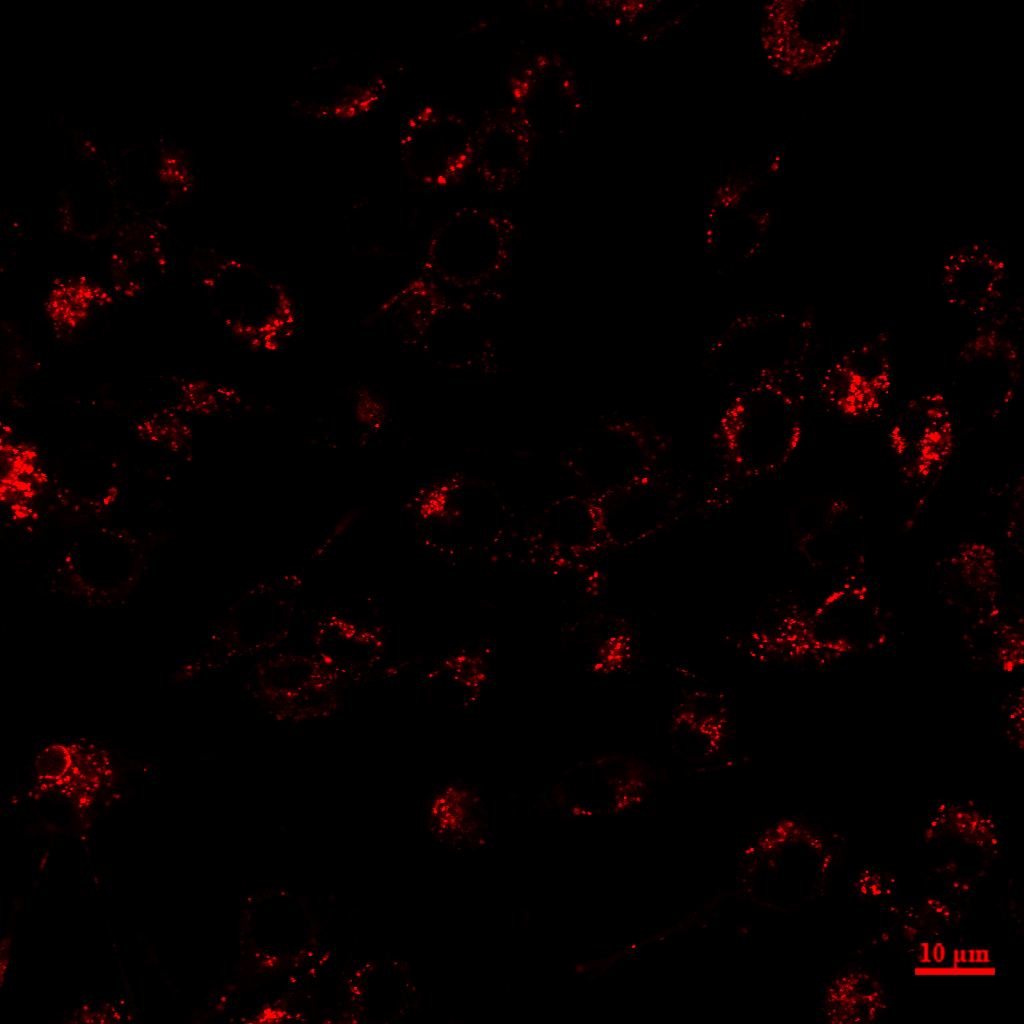

Supplement: Data S2 [file peerj-12-17664-s002.zip › raw data2/double staining+confocal/BV2+LPS_0002.tif.frames/BV2+LPS_0002_C003T001.tif]

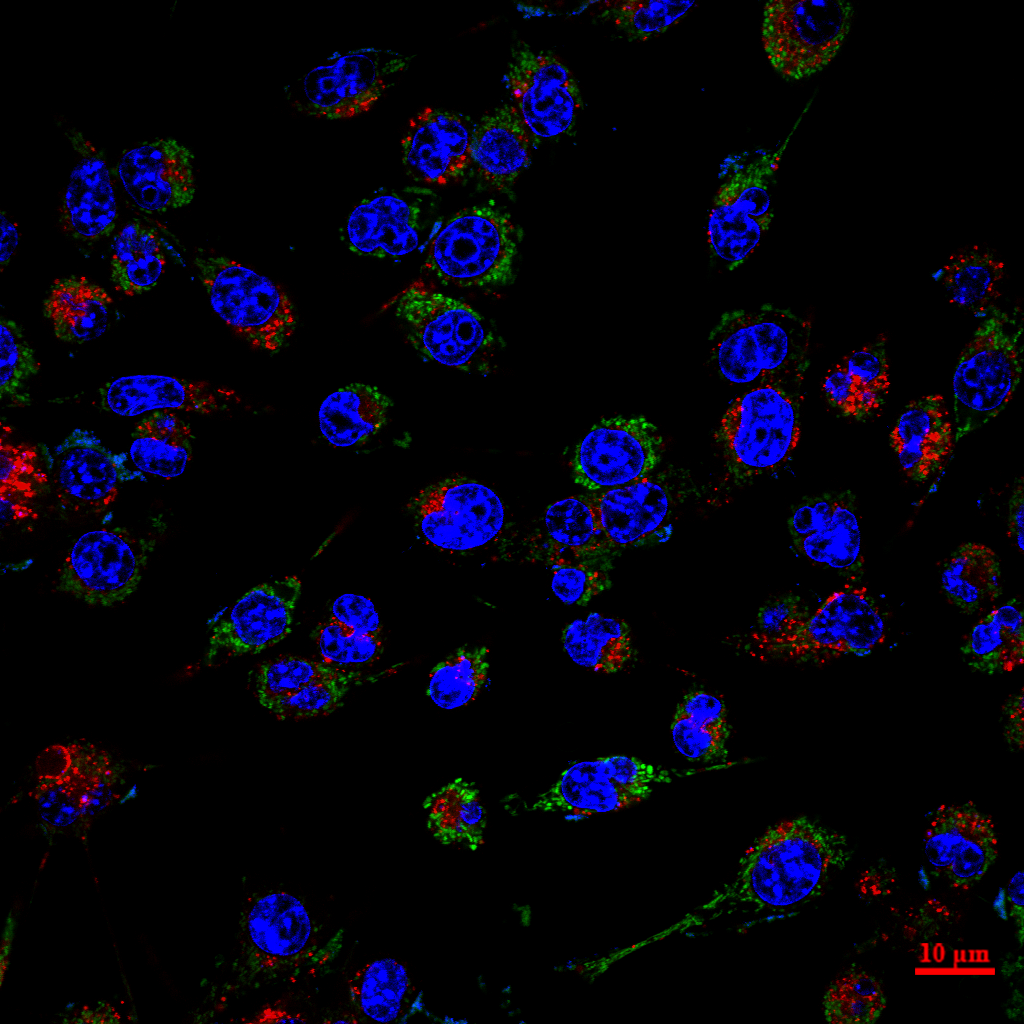

Supplement: Data S2 [file peerj-12-17664-s002.zip › raw data2/double staining+confocal/BV2+LPS_0002.tif.frames/BV2+LPS_0002_T001.tif]

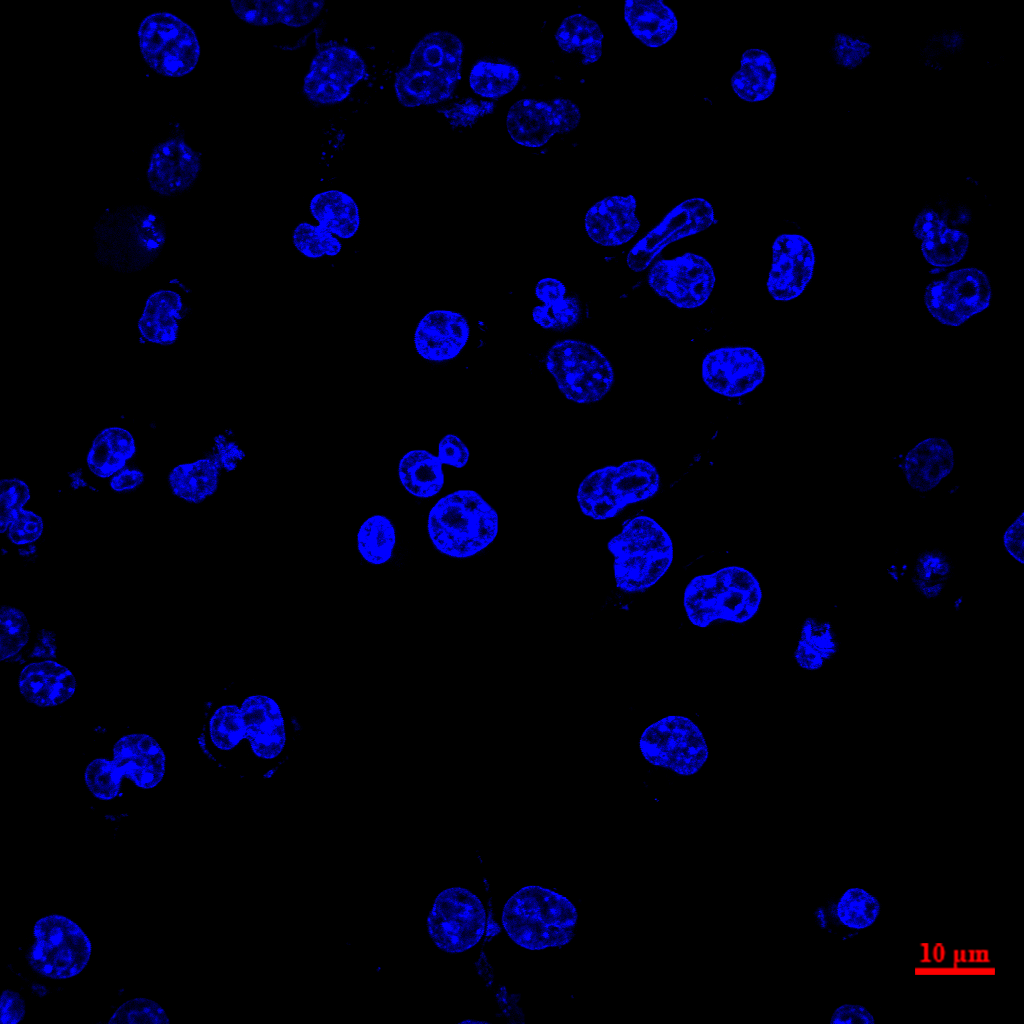

Supplement: Data S2 [file peerj-12-17664-s002.zip › raw data2/double staining+confocal/BV2+LPS_0003.tif.frames/BV2+LPS_0003_C001T001.tif]

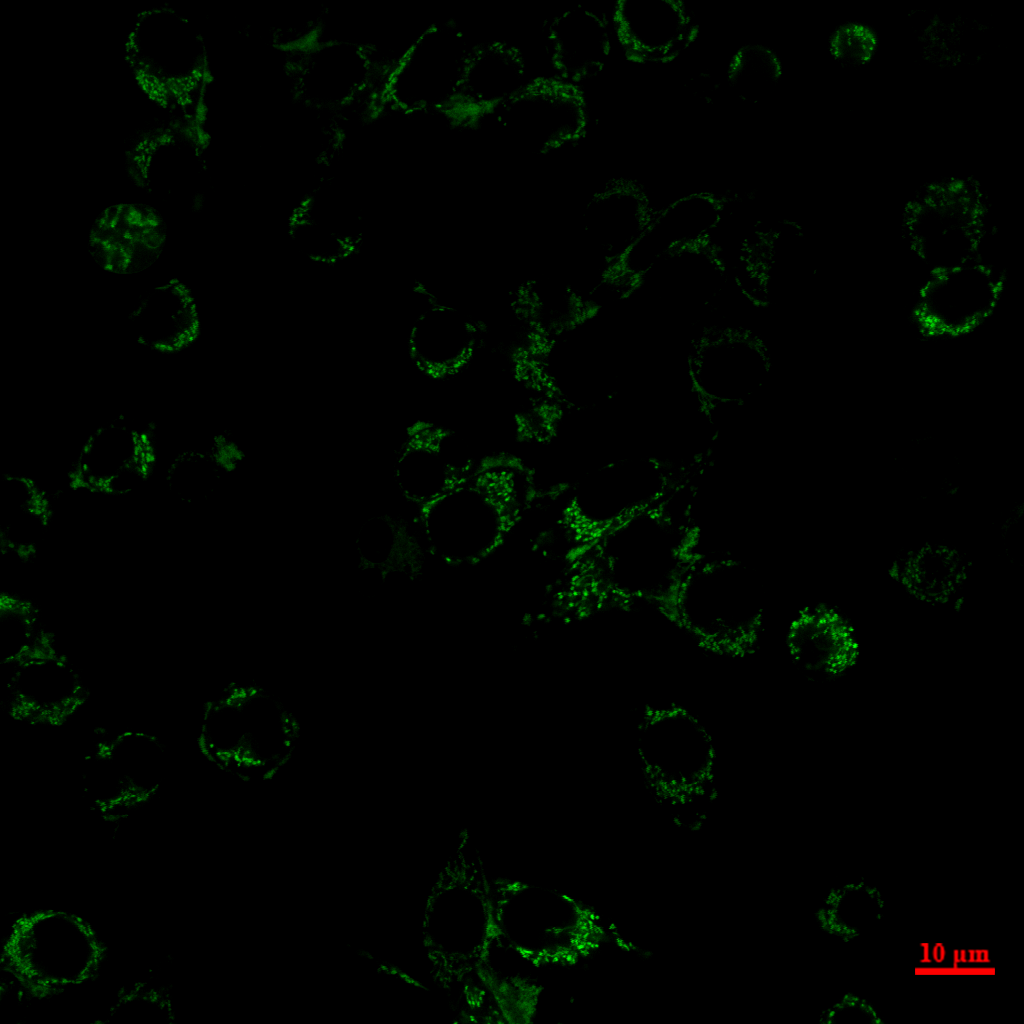

Supplement: Data S2 [file peerj-12-17664-s002.zip › raw data2/double staining+confocal/BV2+LPS_0003.tif.frames/BV2+LPS_0003_C002T001.tif]

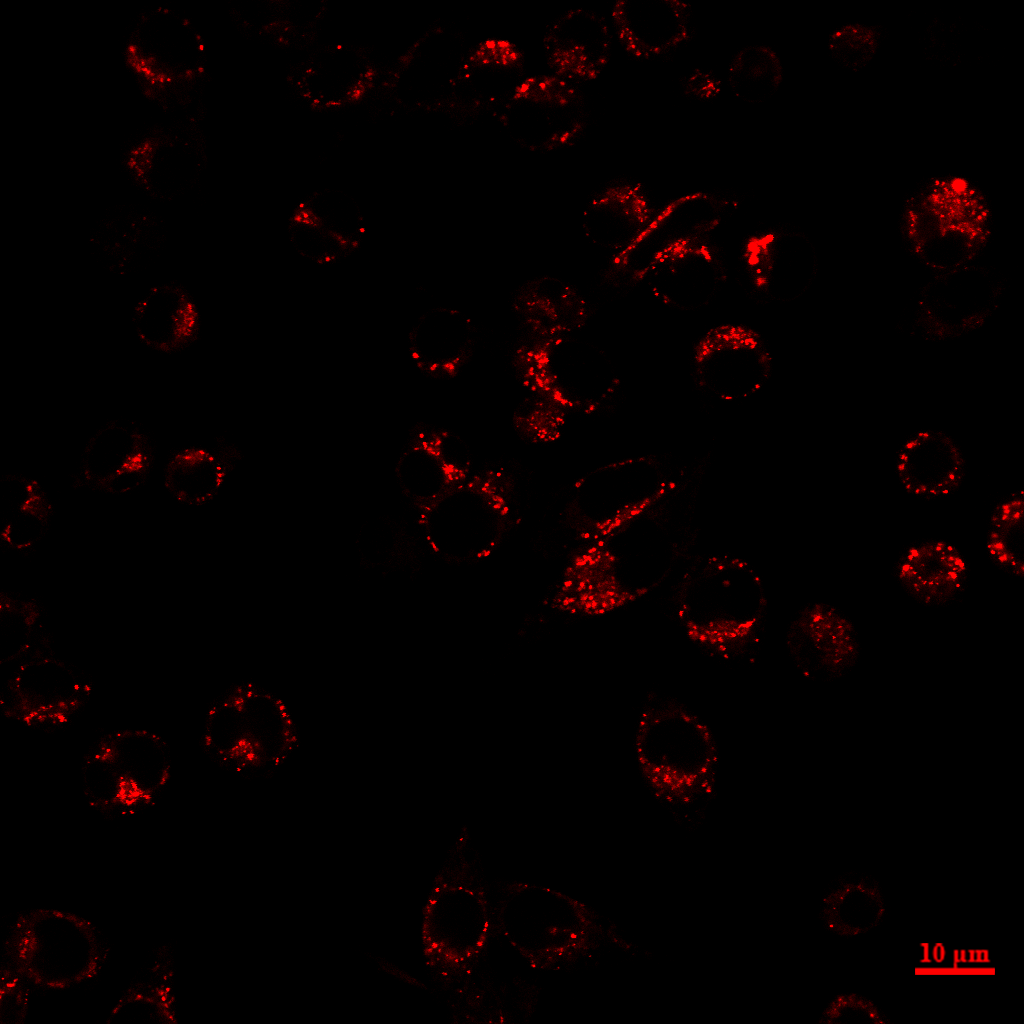

Supplement: Data S2 [file peerj-12-17664-s002.zip › raw data2/double staining+confocal/BV2+LPS_0003.tif.frames/BV2+LPS_0003_C003T001.tif]

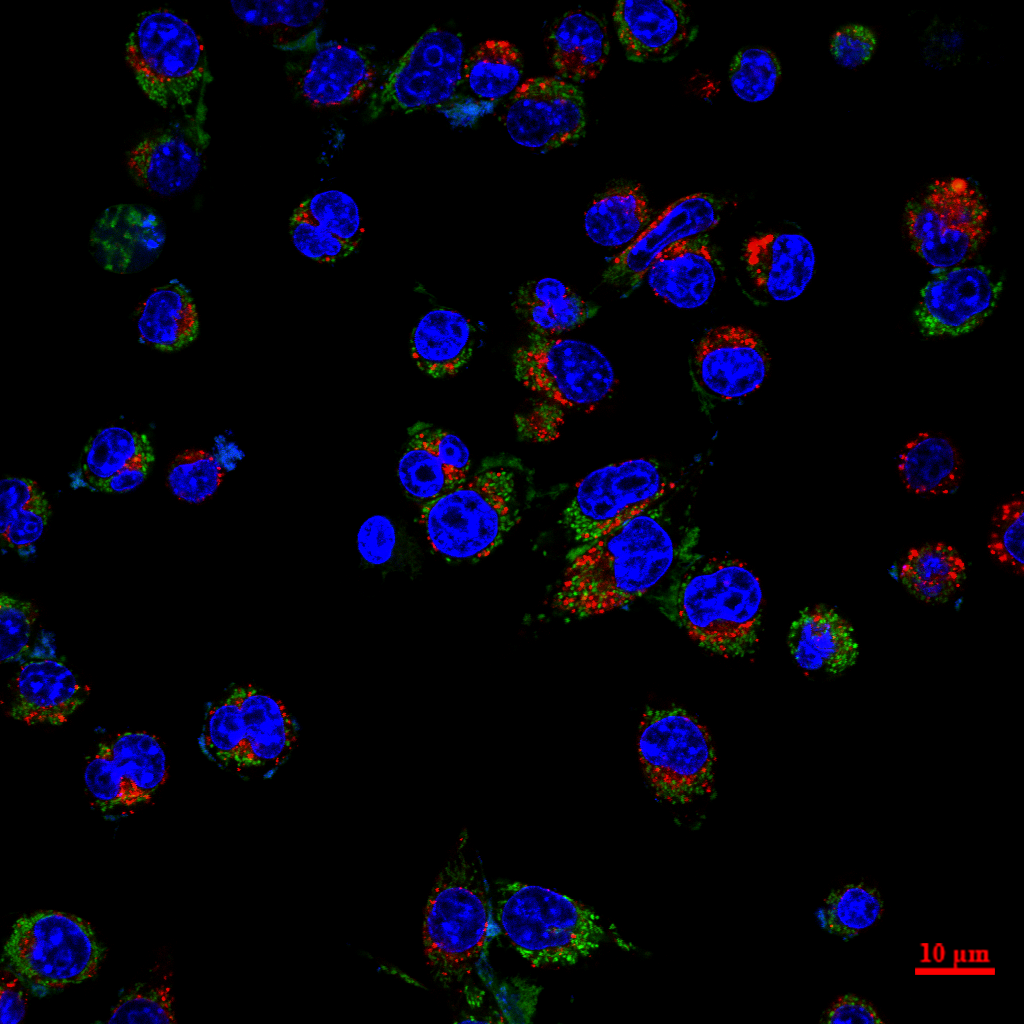

Supplement: Data S2 [file peerj-12-17664-s002.zip › raw data2/double staining+confocal/BV2+LPS_0003.tif.frames/BV2+LPS_0003_T001.tif]

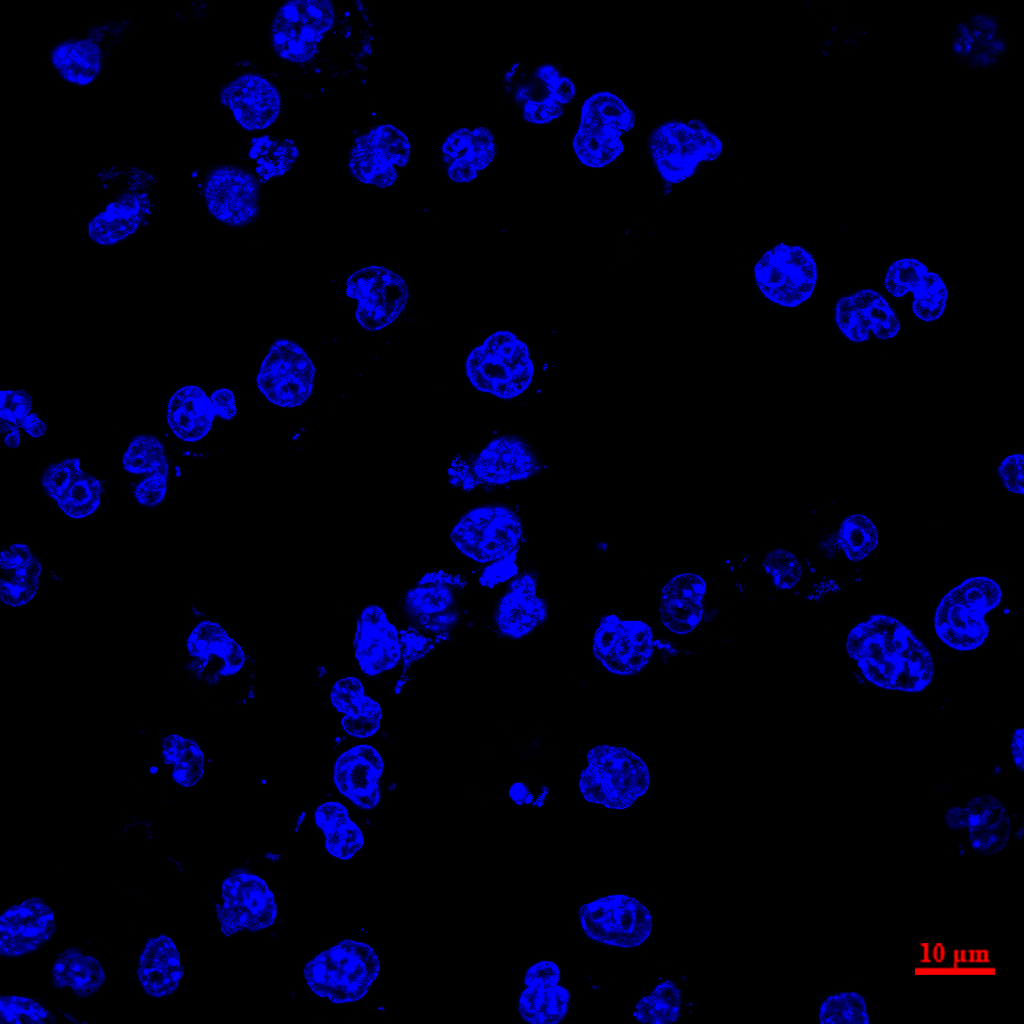

Supplement: Data S2 [file peerj-12-17664-s002.zip › raw data2/double staining+confocal/BV2+LPS+BMSC-CM_0001.tif.frames/BV2+LPS+BMSC-CM_0001_C001T001.tif]

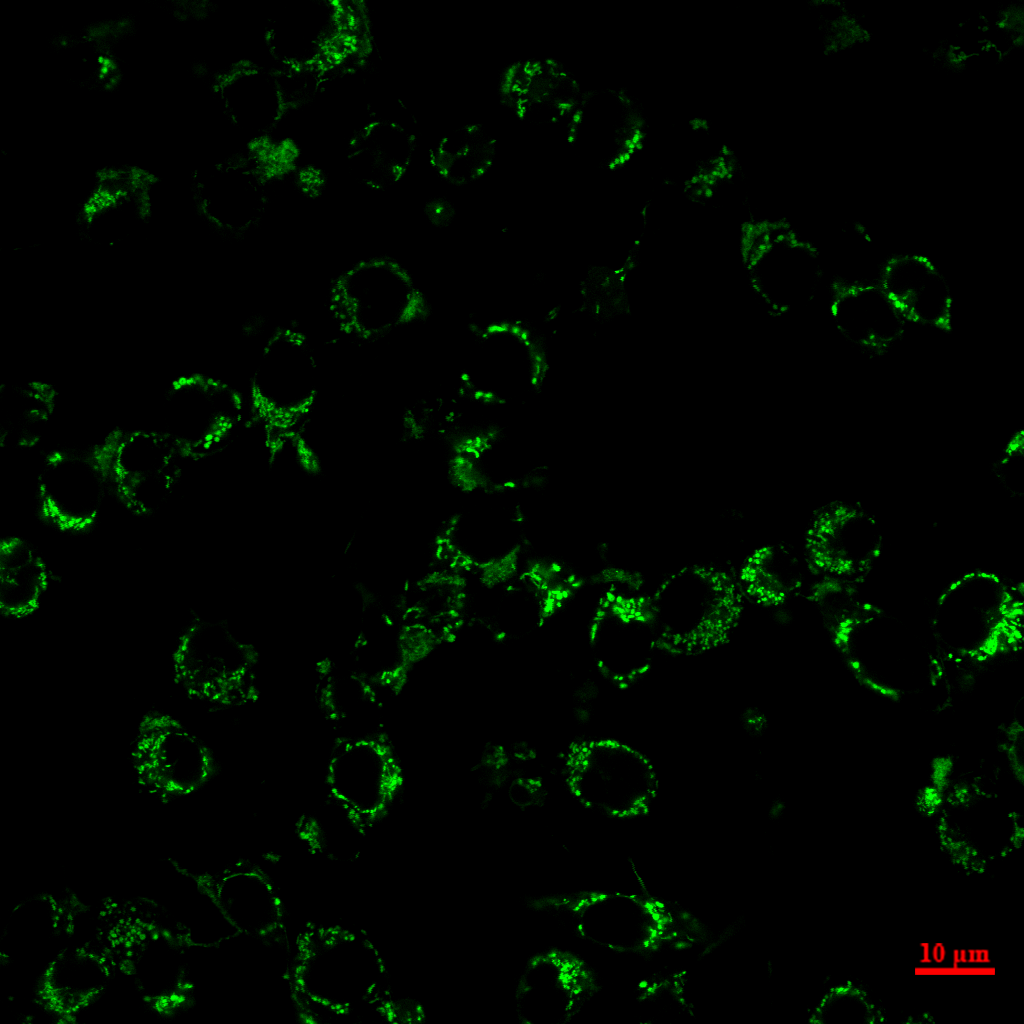

Supplement: Data S2 [file peerj-12-17664-s002.zip › raw data2/double staining+confocal/BV2+LPS+BMSC-CM_0001.tif.frames/BV2+LPS+BMSC-CM_0001_C002T001.tif]

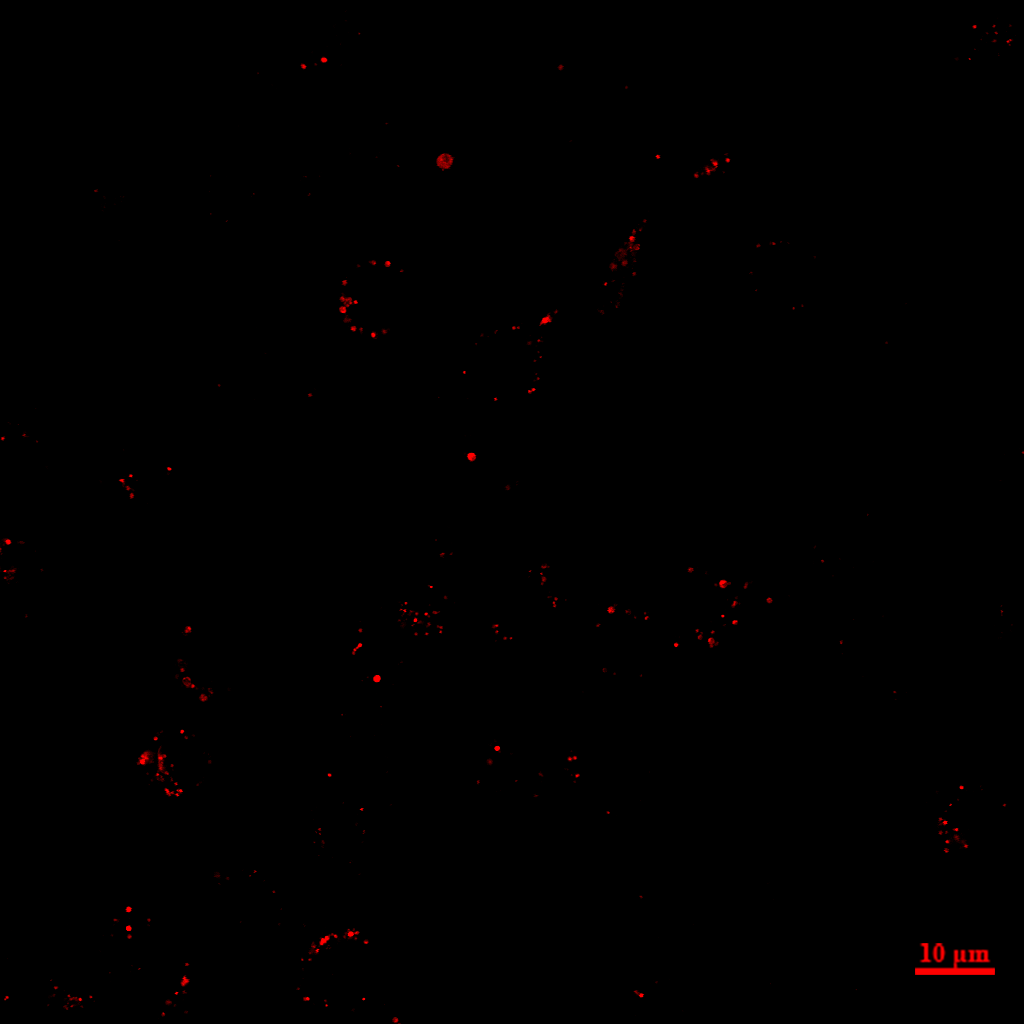

Supplement: Data S2 [file peerj-12-17664-s002.zip › raw data2/double staining+confocal/BV2+LPS+BMSC-CM_0001.tif.frames/BV2+LPS+BMSC-CM_0001_C003T001.tif]

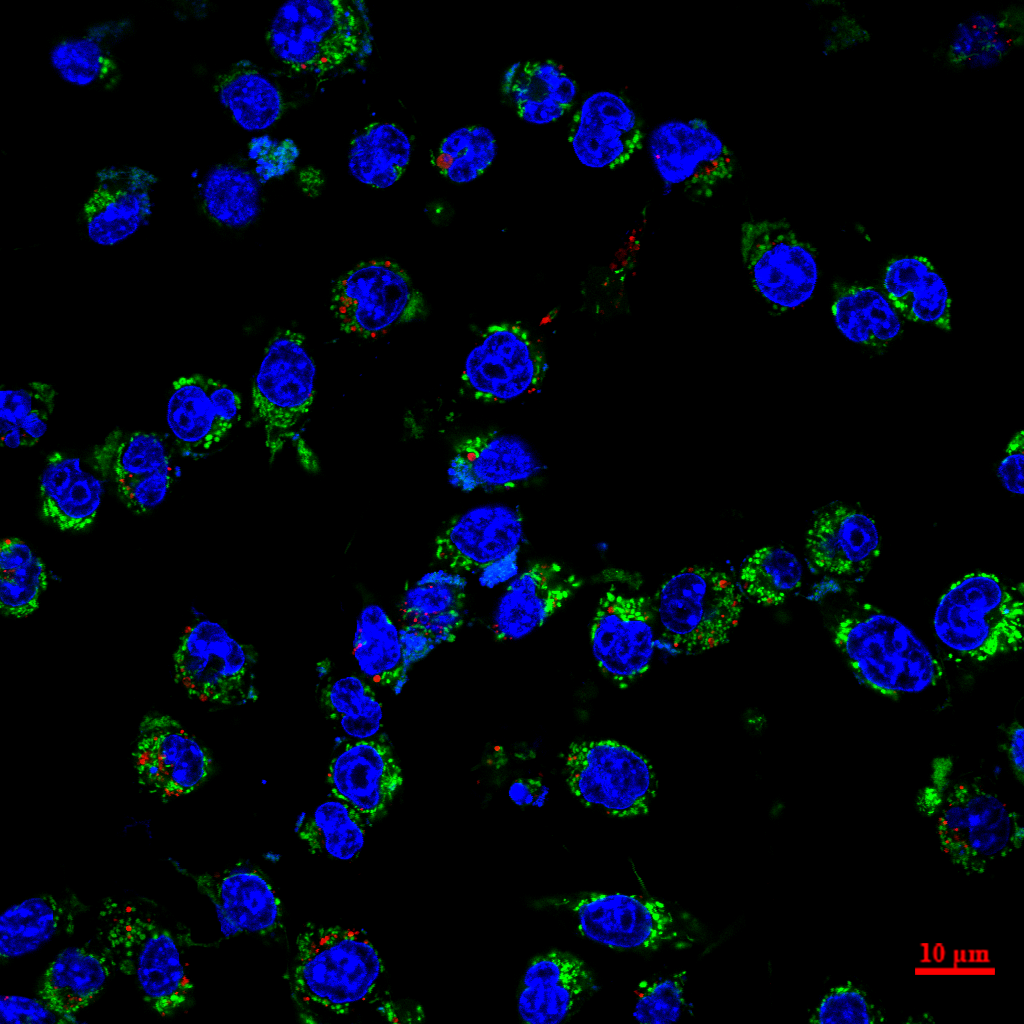

Supplement: Data S2 [file peerj-12-17664-s002.zip › raw data2/double staining+confocal/BV2+LPS+BMSC-CM_0001.tif.frames/BV2+LPS+BMSC-CM_0001_T001.tif]

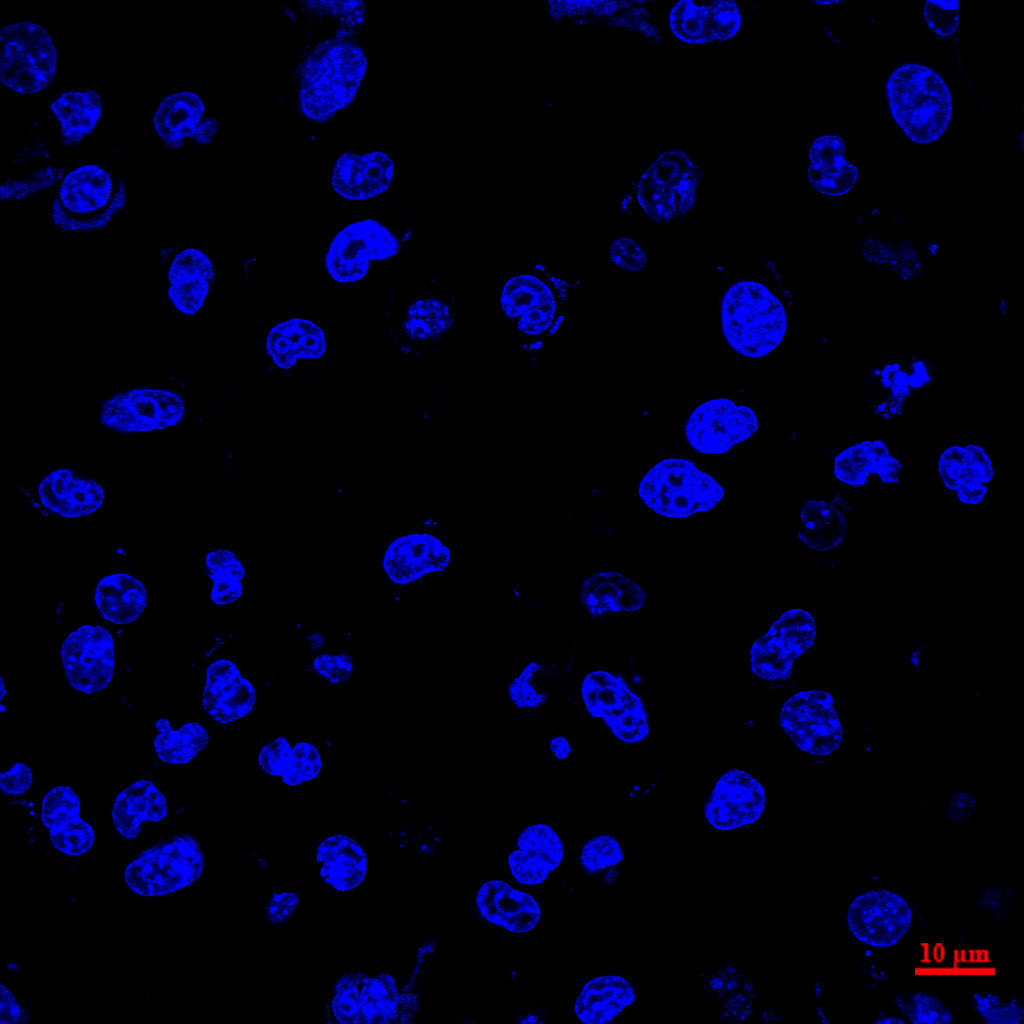

Supplement: Data S2 [file peerj-12-17664-s002.zip › raw data2/double staining+confocal/BV2+LPS+BMSC-CM_0002.tif.frames/BV2+LPS+BMSC-CM_0002_C001T001.tif]

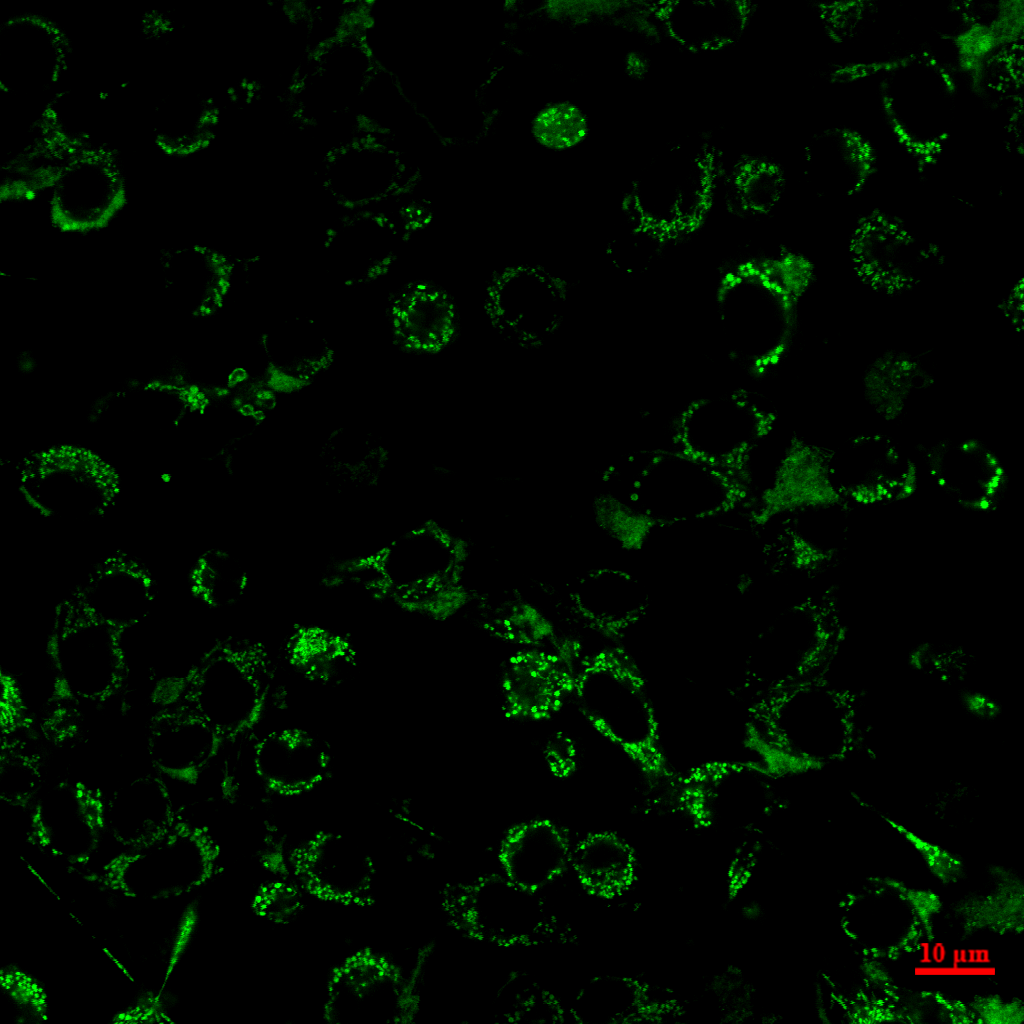

Supplement: Data S2 [file peerj-12-17664-s002.zip › raw data2/double staining+confocal/BV2+LPS+BMSC-CM_0002.tif.frames/BV2+LPS+BMSC-CM_0002_C002T001.tif]

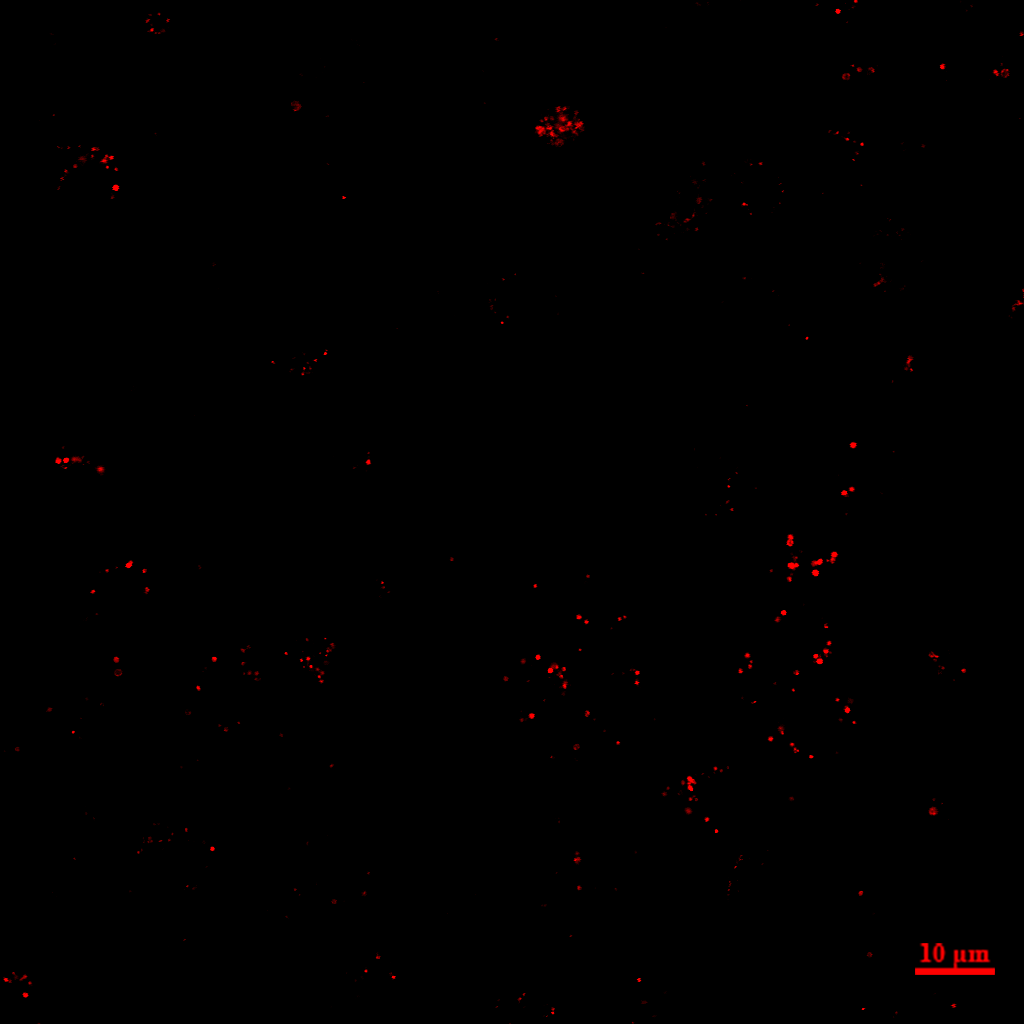

Supplement: Data S2 [file peerj-12-17664-s002.zip › raw data2/double staining+confocal/BV2+LPS+BMSC-CM_0002.tif.frames/BV2+LPS+BMSC-CM_0002_C003T001.tif]

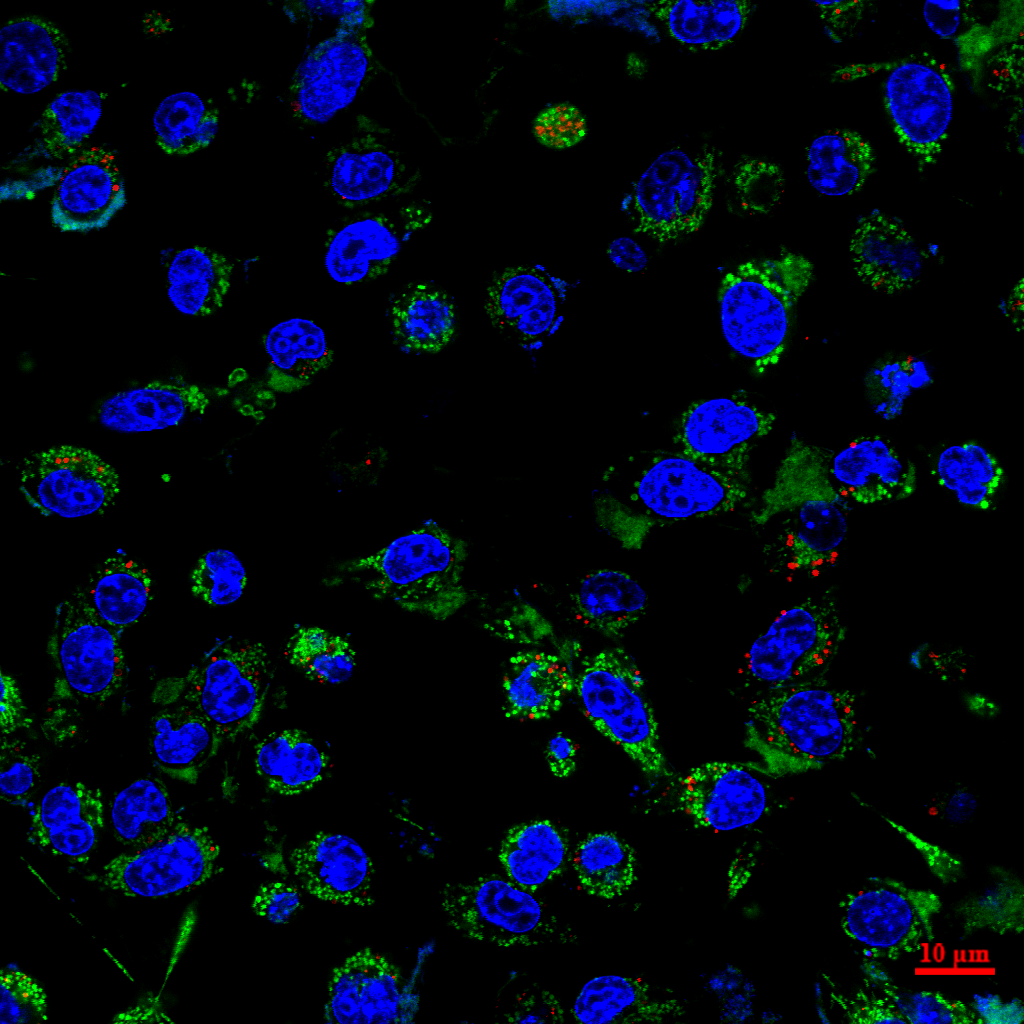

Supplement: Data S2 [file peerj-12-17664-s002.zip › raw data2/double staining+confocal/BV2+LPS+BMSC-CM_0002.tif.frames/BV2+LPS+BMSC-CM_0002_T001.tif]

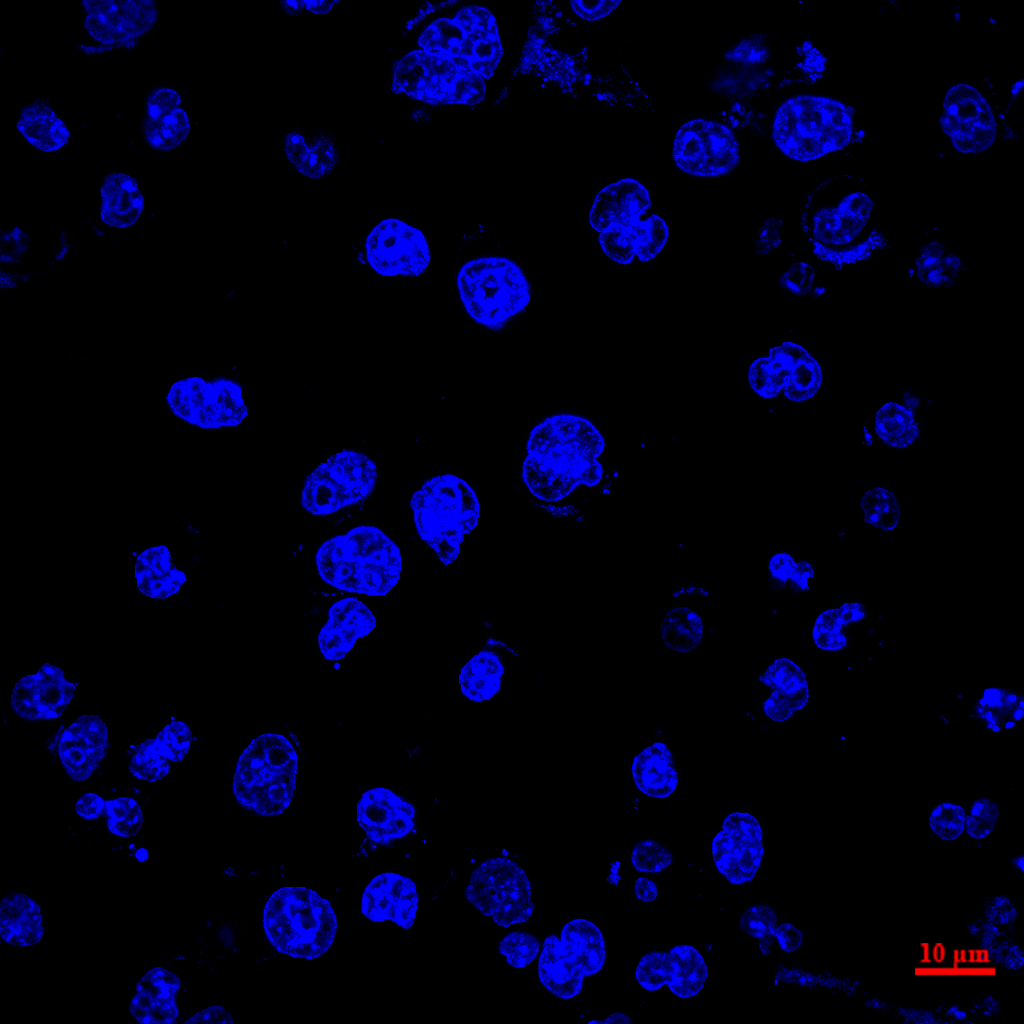

Supplement: Data S2 [file peerj-12-17664-s002.zip › raw data2/double staining+confocal/BV2+LPS+BMSC-CM_0003.tif.frames/BV2+LPS+BMSC-CM_0003_C001T001.tif]

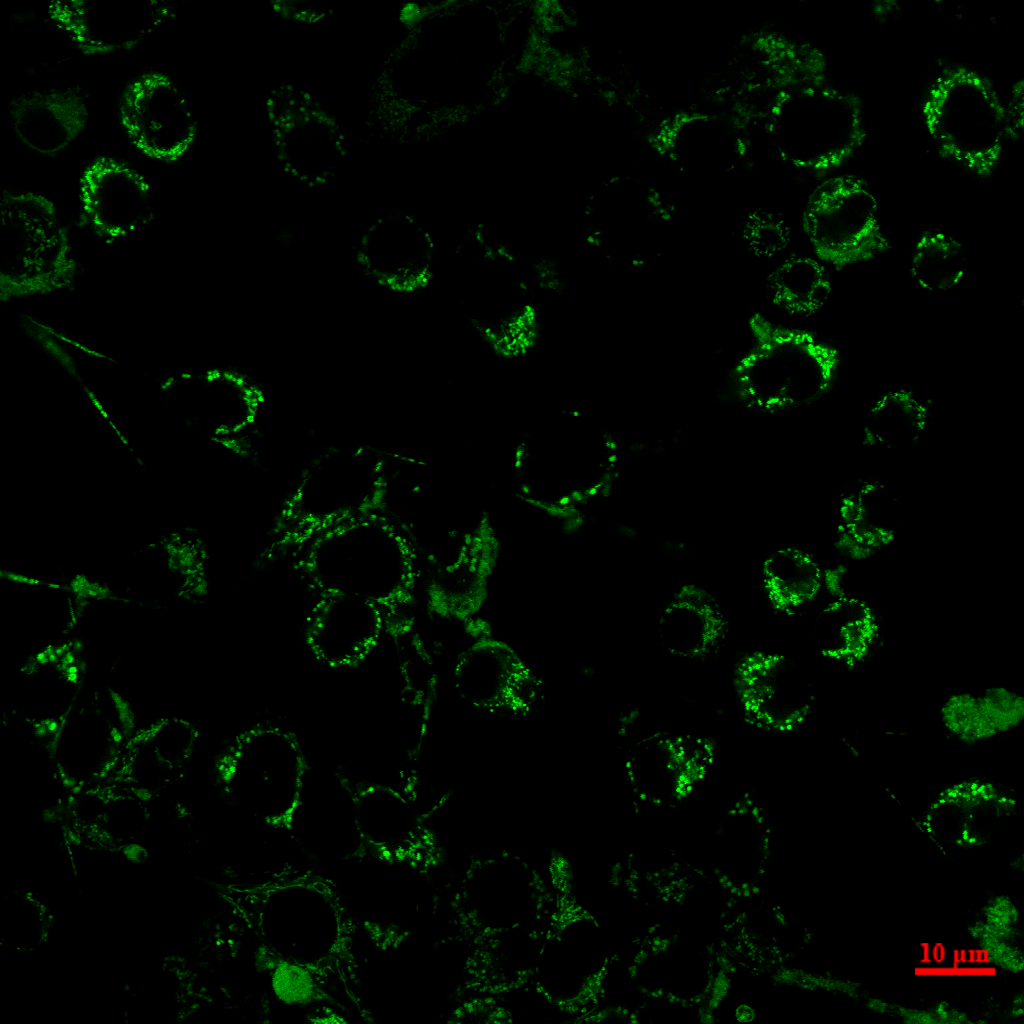

Supplement: Data S2 [file peerj-12-17664-s002.zip › raw data2/double staining+confocal/BV2+LPS+BMSC-CM_0003.tif.frames/BV2+LPS+BMSC-CM_0003_C002T001.tif]

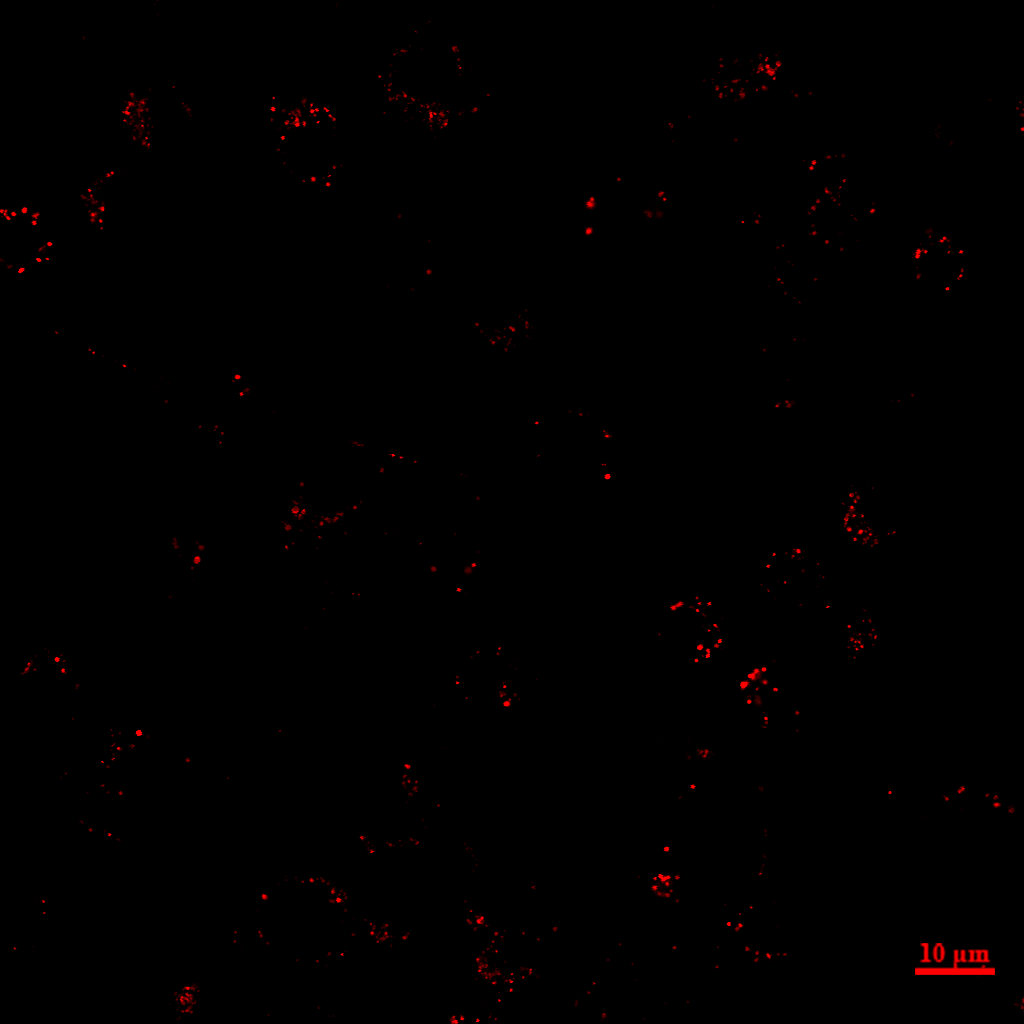

Supplement: Data S2 [file peerj-12-17664-s002.zip › raw data2/double staining+confocal/BV2+LPS+BMSC-CM_0003.tif.frames/BV2+LPS+BMSC-CM_0003_C003T001.tif]

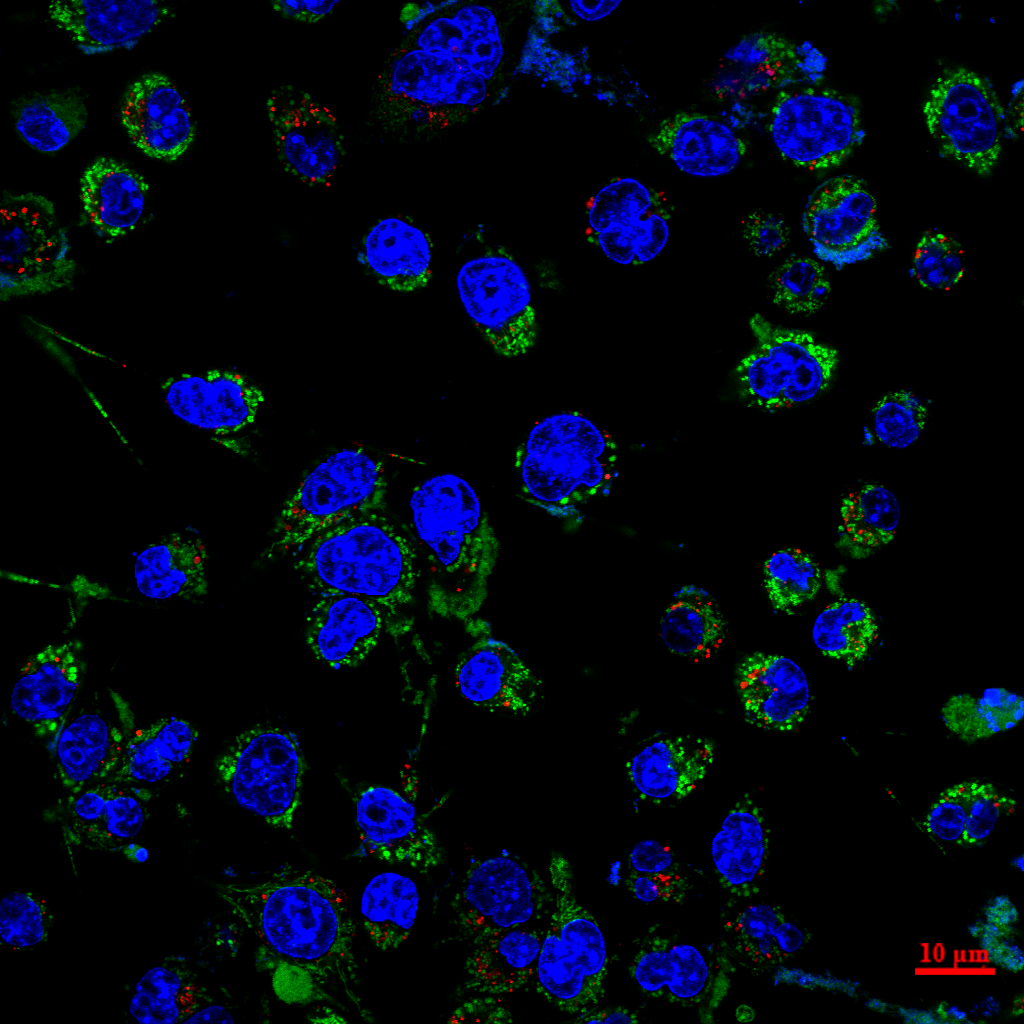

Supplement: Data S2 [file peerj-12-17664-s002.zip › raw data2/double staining+confocal/BV2+LPS+BMSC-CM_0003.tif.frames/BV2+LPS+BMSC-CM_0003_T001.tif]

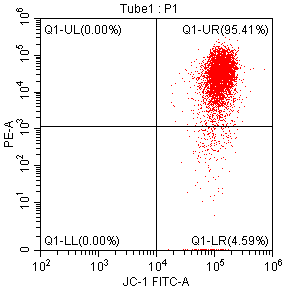

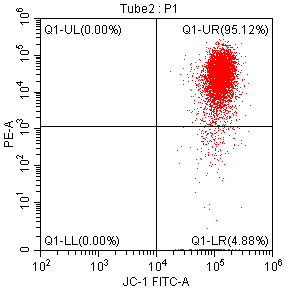

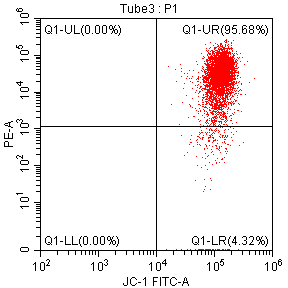

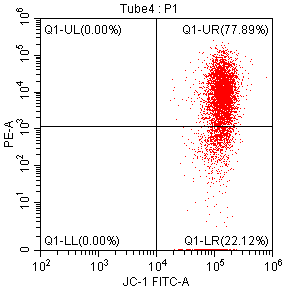

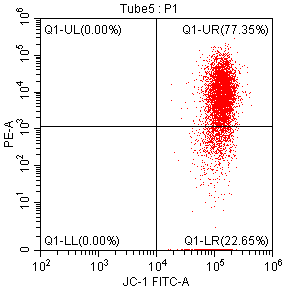

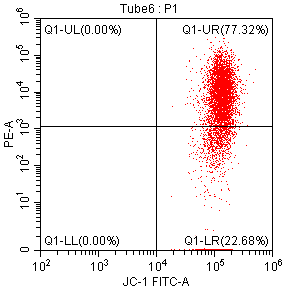

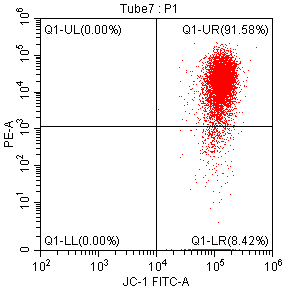

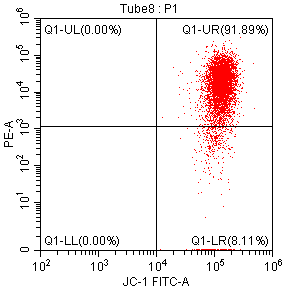

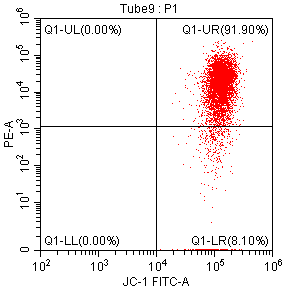

Supplement: Data S2 [file peerj-12-17664-s002.zip › raw data2/JC1/result.docx]

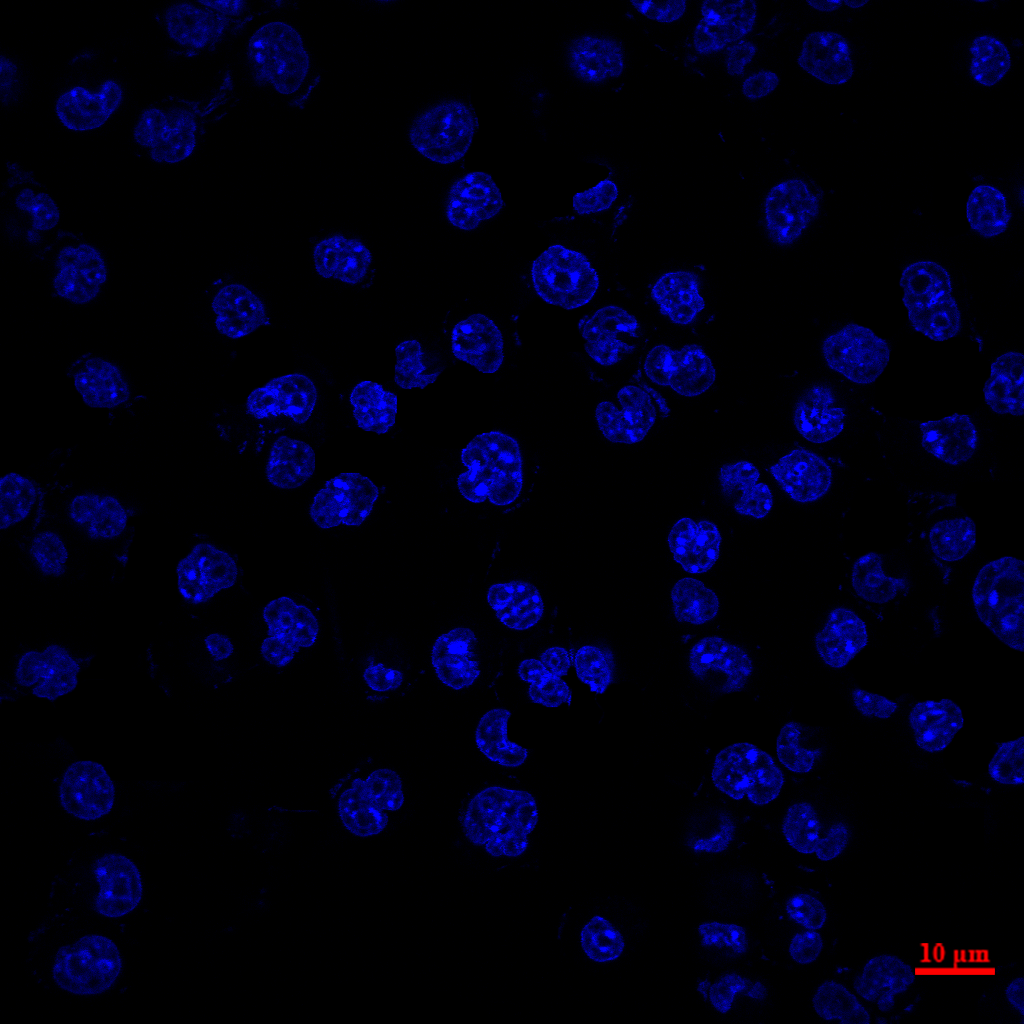

Supplement: Data S2 [file peerj-12-17664-s002.zip › raw data2/ROS+confocal/BV2_0001.tif.frames/BV2_0001_C001T001.tif]

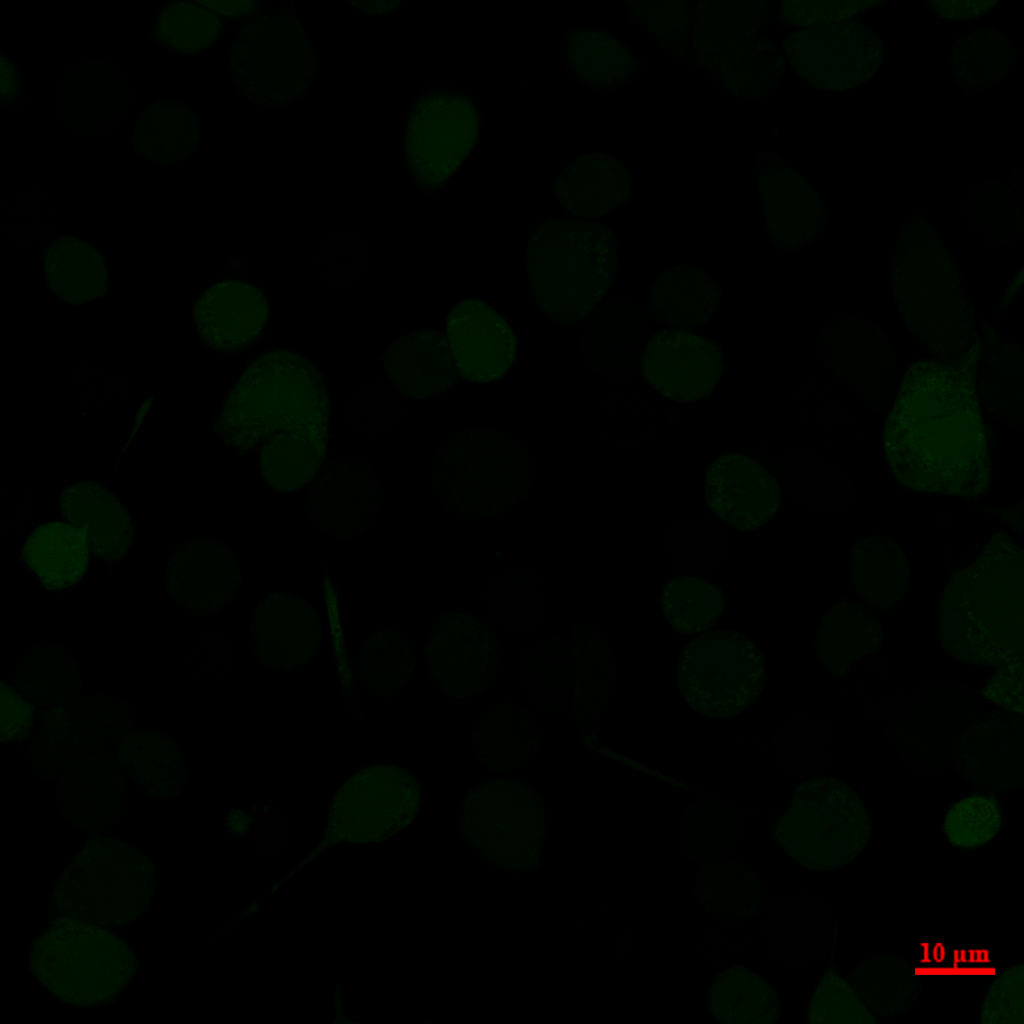

Supplement: Data S2 [file peerj-12-17664-s002.zip › raw data2/ROS+confocal/BV2_0001.tif.frames/BV2_0001_C002T001.tif]

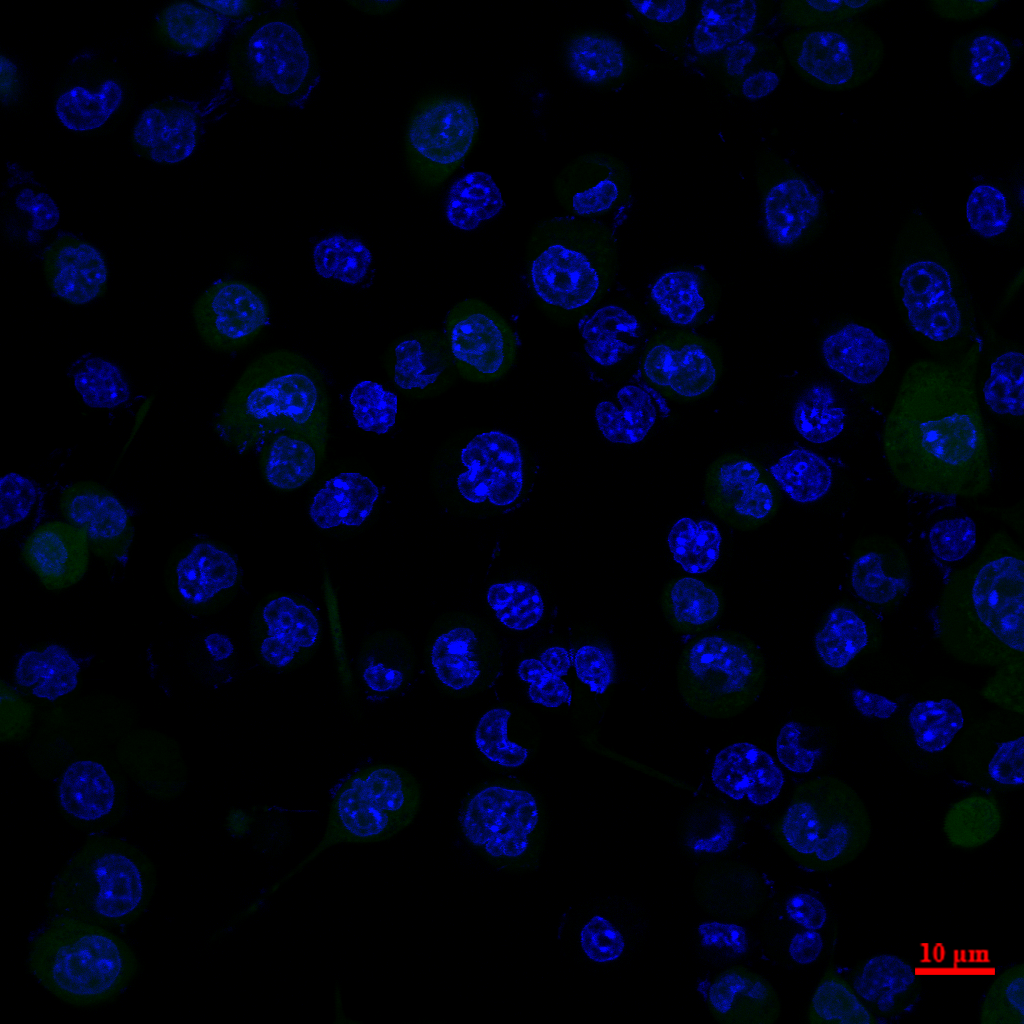

Supplement: Data S2 [file peerj-12-17664-s002.zip › raw data2/ROS+confocal/BV2_0001.tif.frames/BV2_0001_T001.tif]

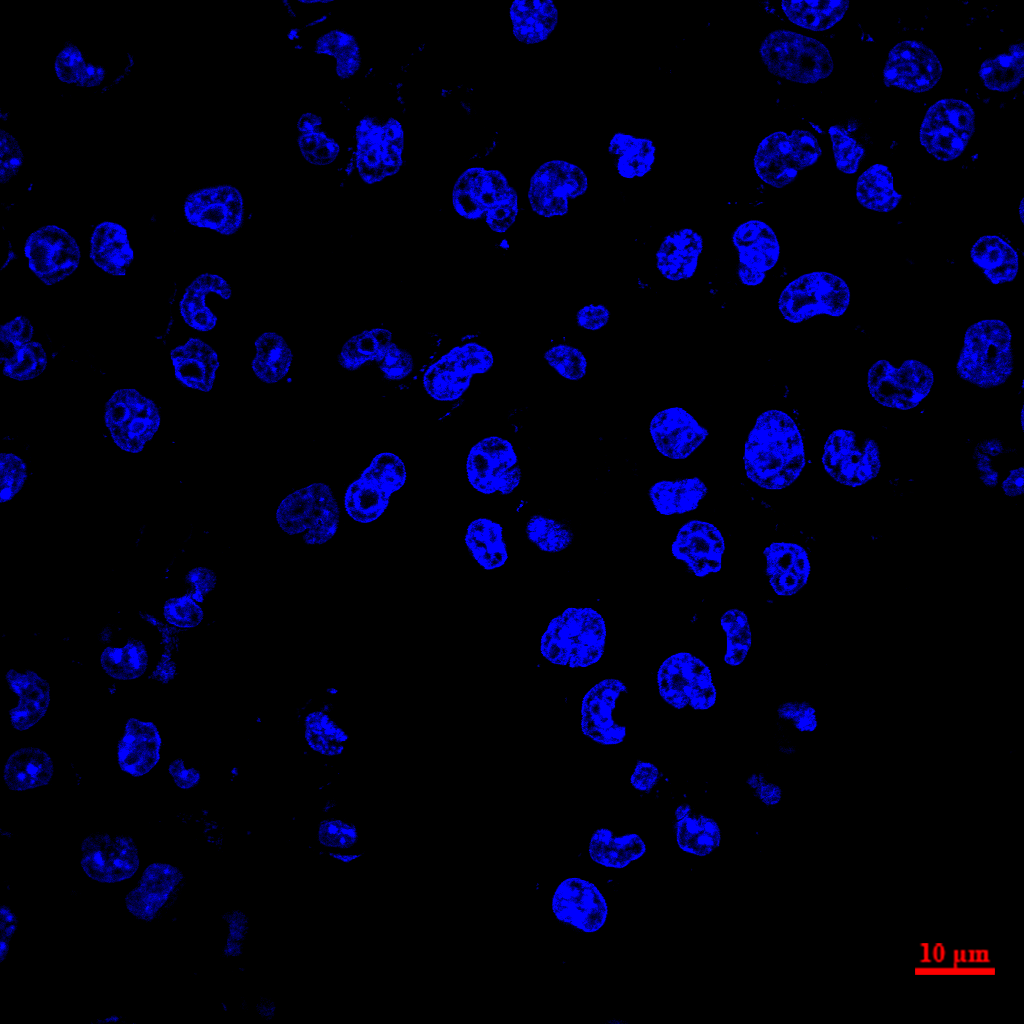

Supplement: Data S2 [file peerj-12-17664-s002.zip › raw data2/ROS+confocal/BV2_0002.tif.frames/BV2_0002_C001T001.tif]

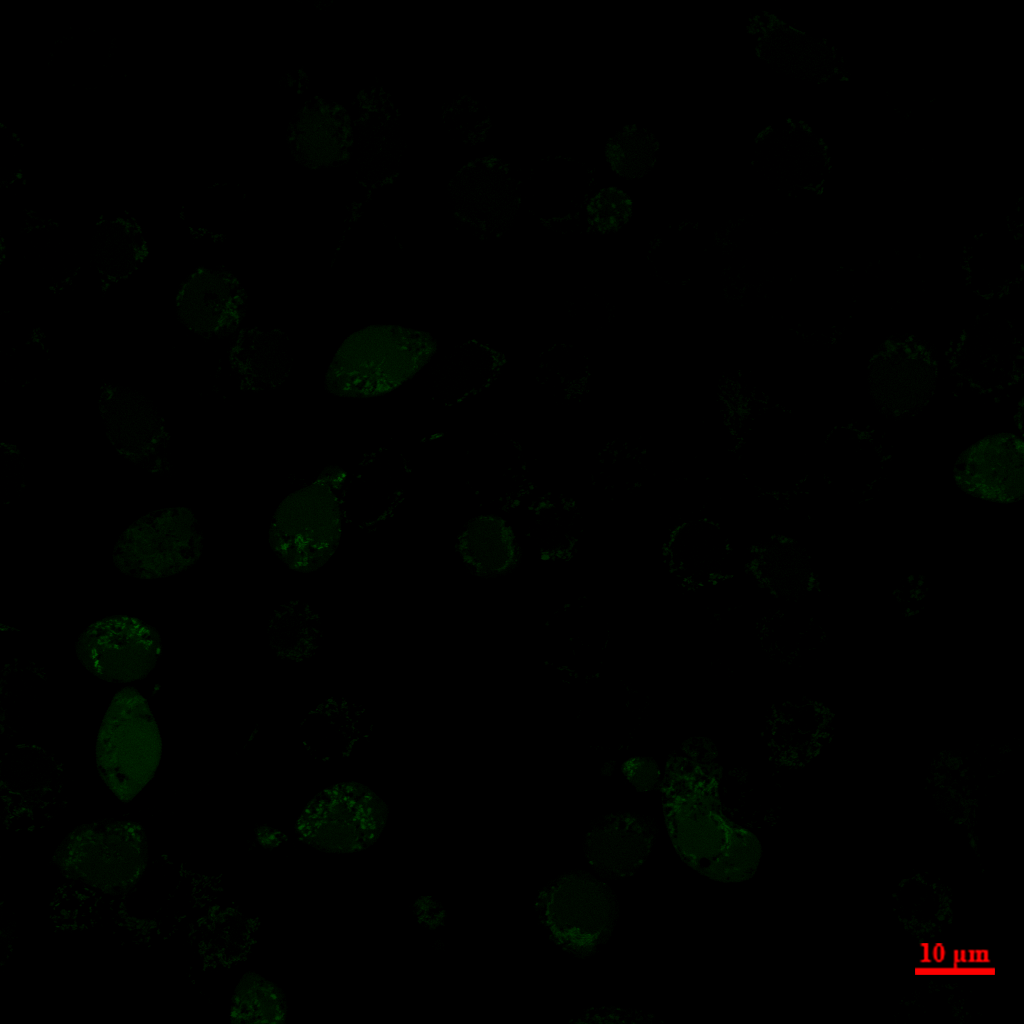

Supplement: Data S2 [file peerj-12-17664-s002.zip › raw data2/ROS+confocal/BV2_0002.tif.frames/BV2_0002_C002T001.tif]

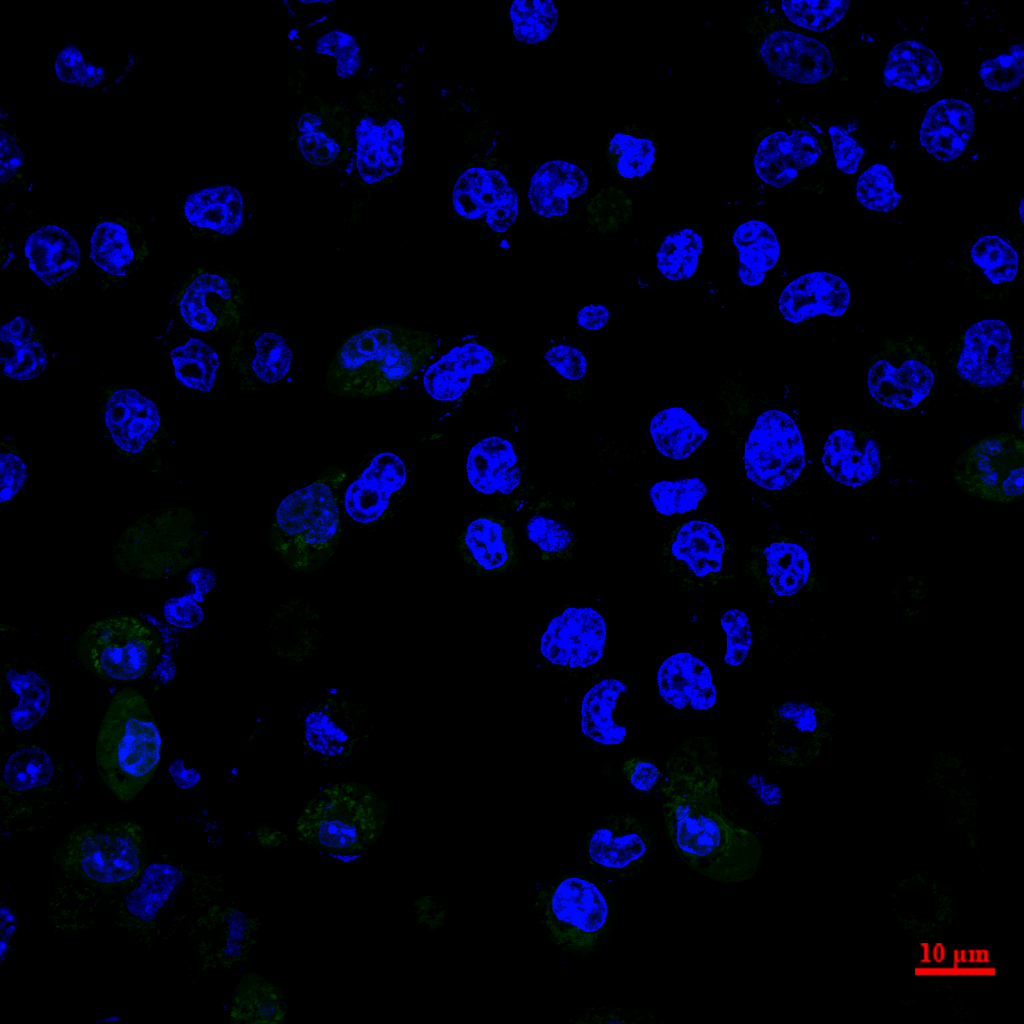

Supplement: Data S2 [file peerj-12-17664-s002.zip › raw data2/ROS+confocal/BV2_0002.tif.frames/BV2_0002_T001.tif]

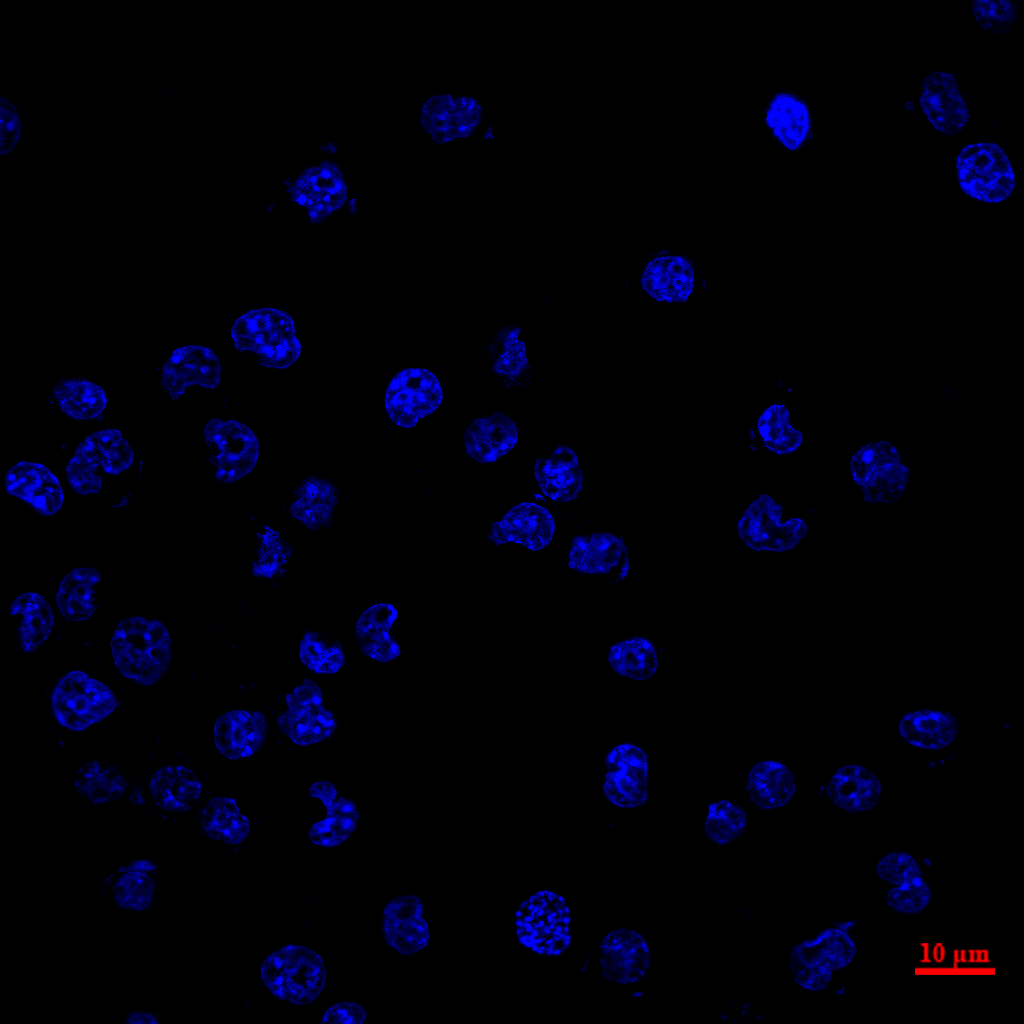

Supplement: Data S2 [file peerj-12-17664-s002.zip › raw data2/ROS+confocal/BV2_0003.tif.frames/BV2_0003_C001T001.tif]

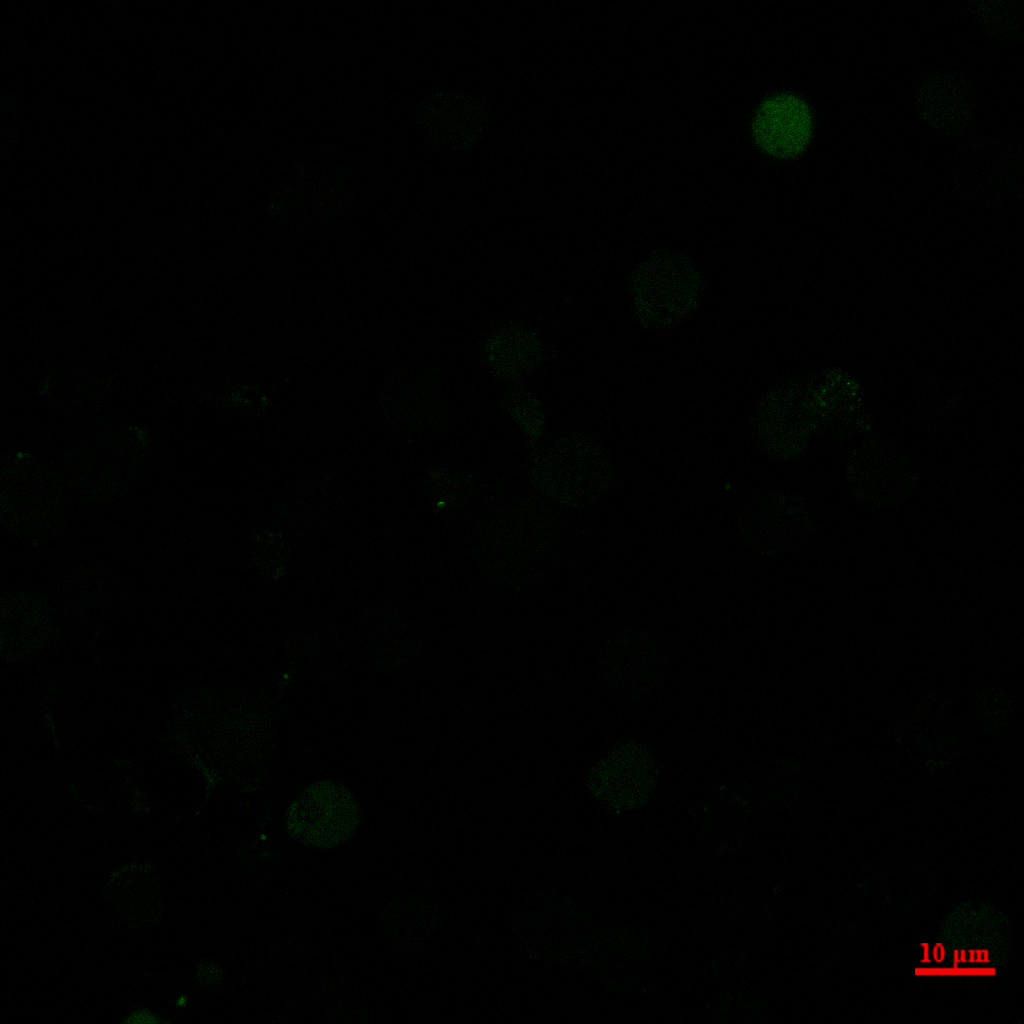

Supplement: Data S2 [file peerj-12-17664-s002.zip › raw data2/ROS+confocal/BV2_0003.tif.frames/BV2_0003_C002T001.tif]

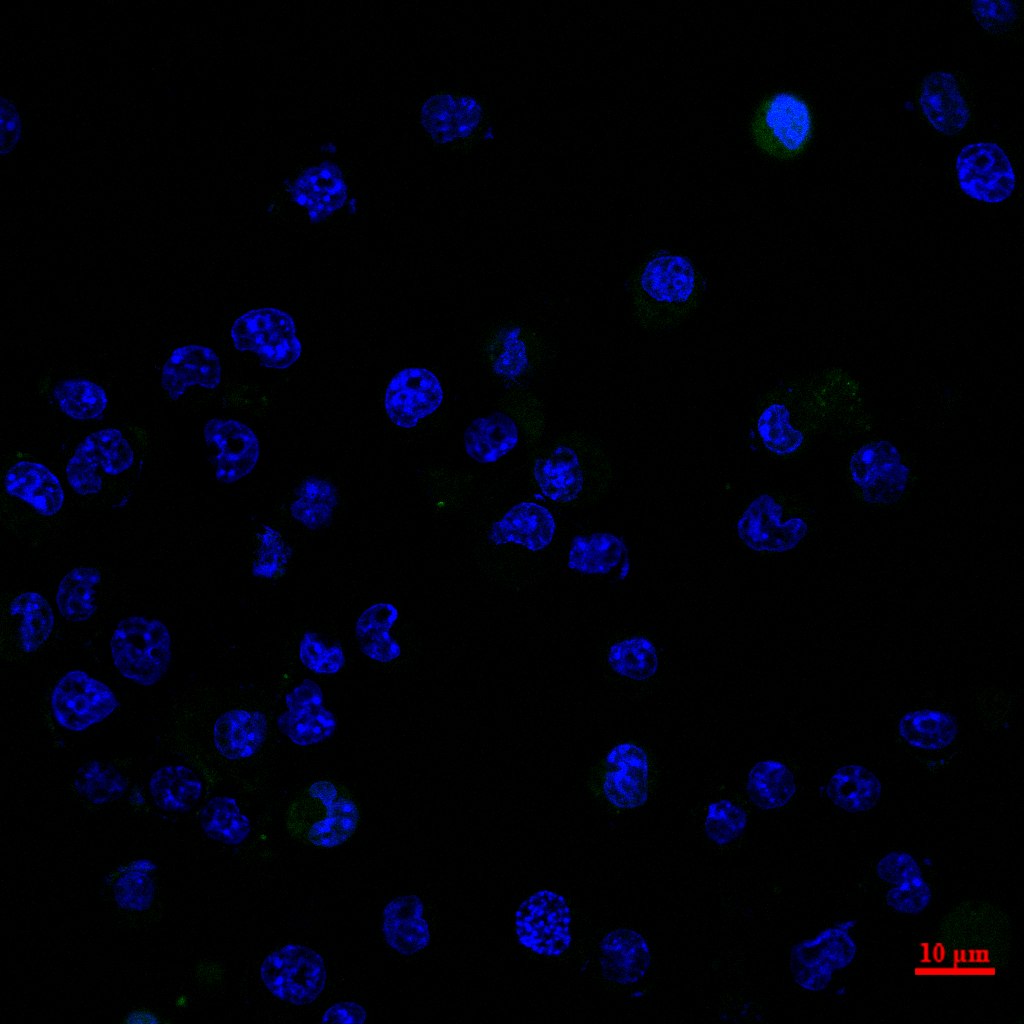

Supplement: Data S2 [file peerj-12-17664-s002.zip › raw data2/ROS+confocal/BV2_0003.tif.frames/BV2_0003_T001.tif]

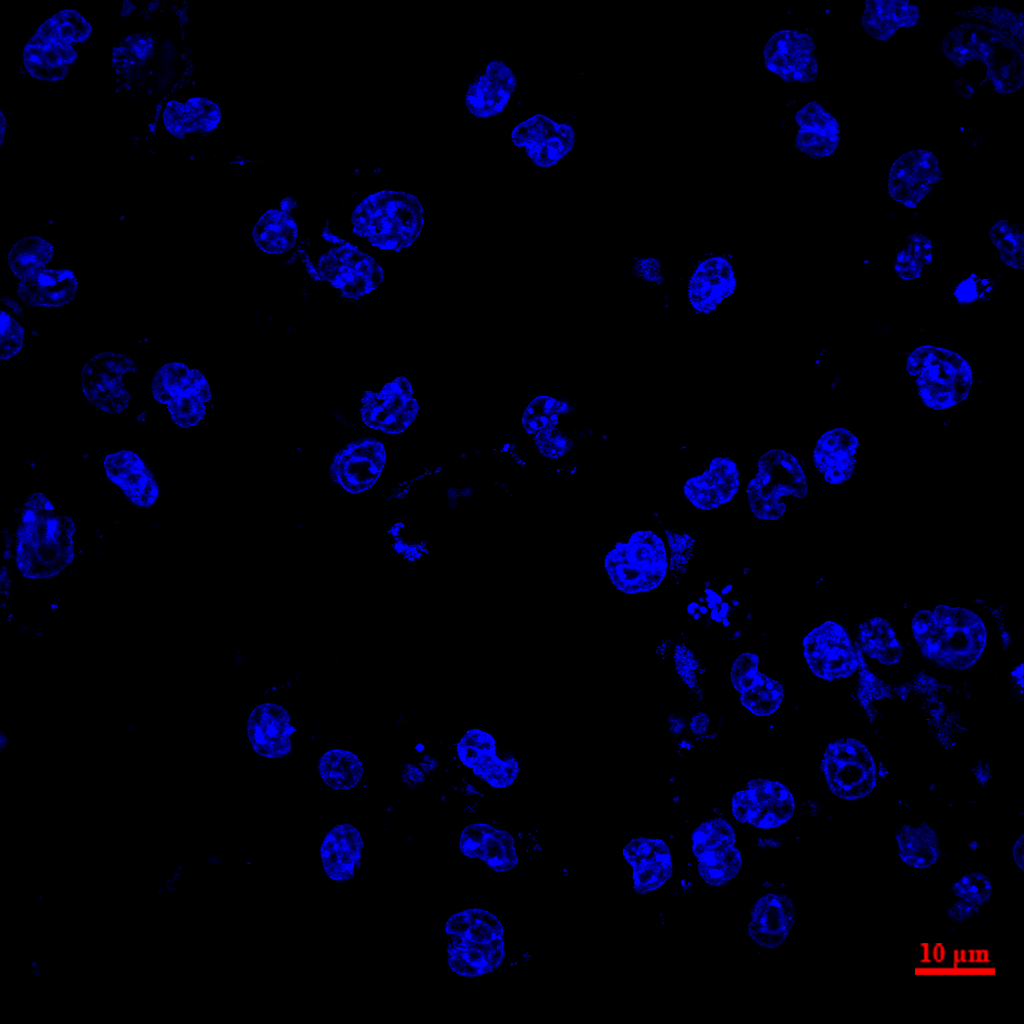

Supplement: Data S2 [file peerj-12-17664-s002.zip › raw data2/ROS+confocal/BV2+LPS_0001.tif.frames/BV2+LPS_0001_C001T001.tif]

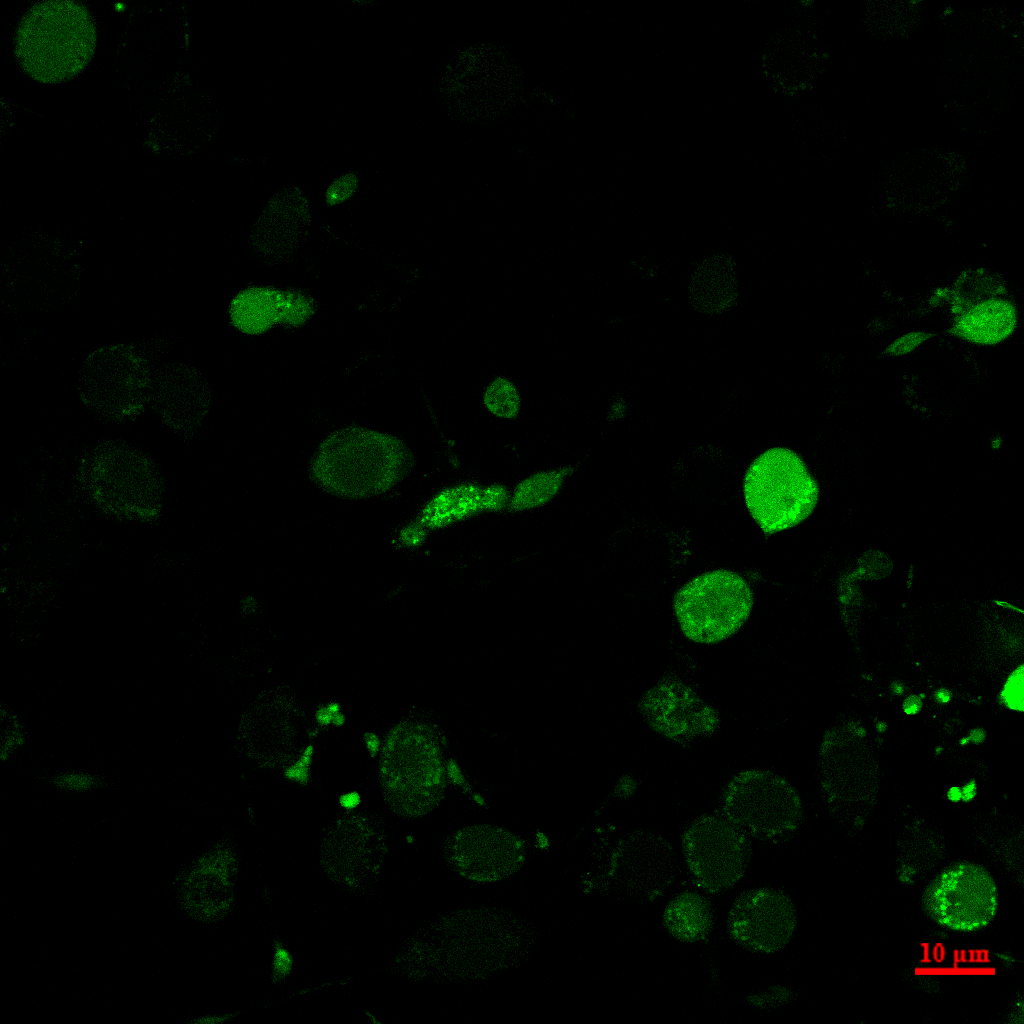

Supplement: Data S2 [file peerj-12-17664-s002.zip › raw data2/ROS+confocal/BV2+LPS_0001.tif.frames/BV2+LPS_0001_C002T001.tif]

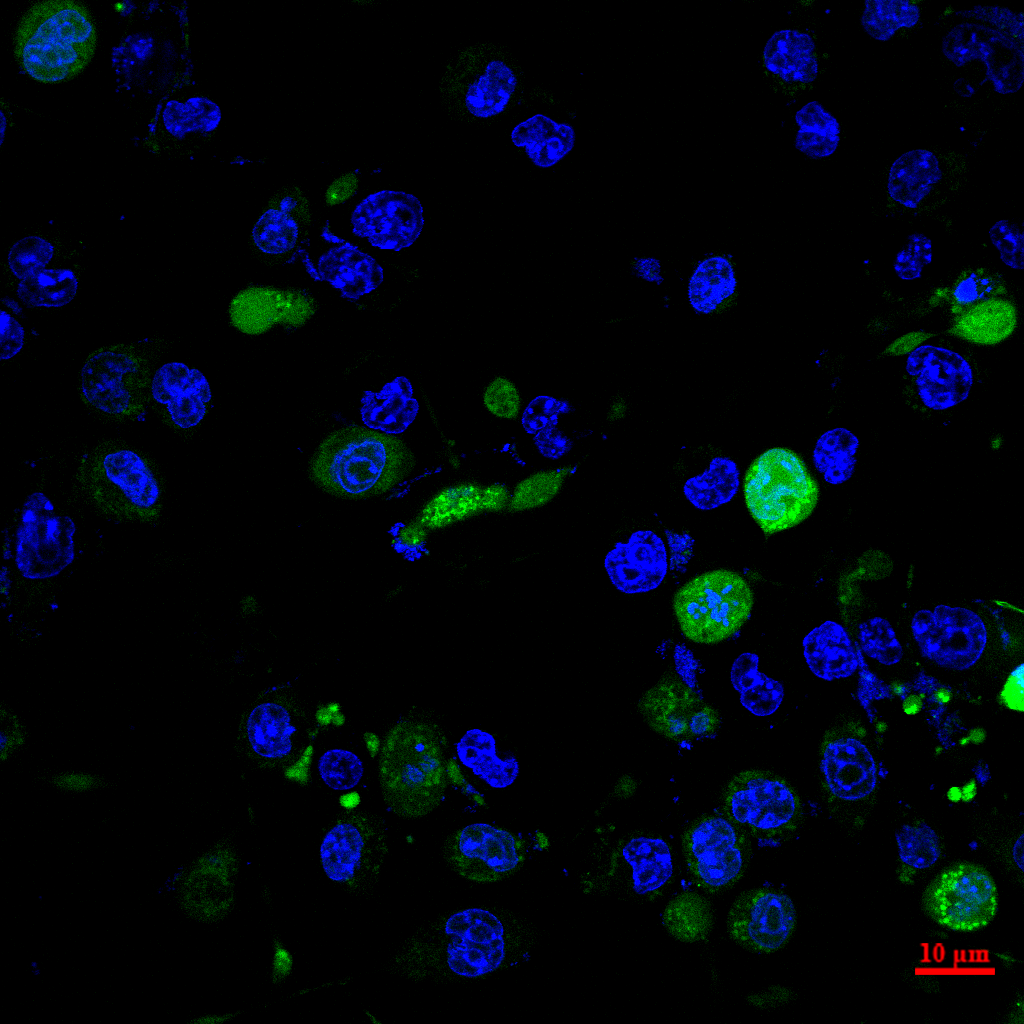

Supplement: Data S2 [file peerj-12-17664-s002.zip › raw data2/ROS+confocal/BV2+LPS_0001.tif.frames/BV2+LPS_0001_T001.tif]

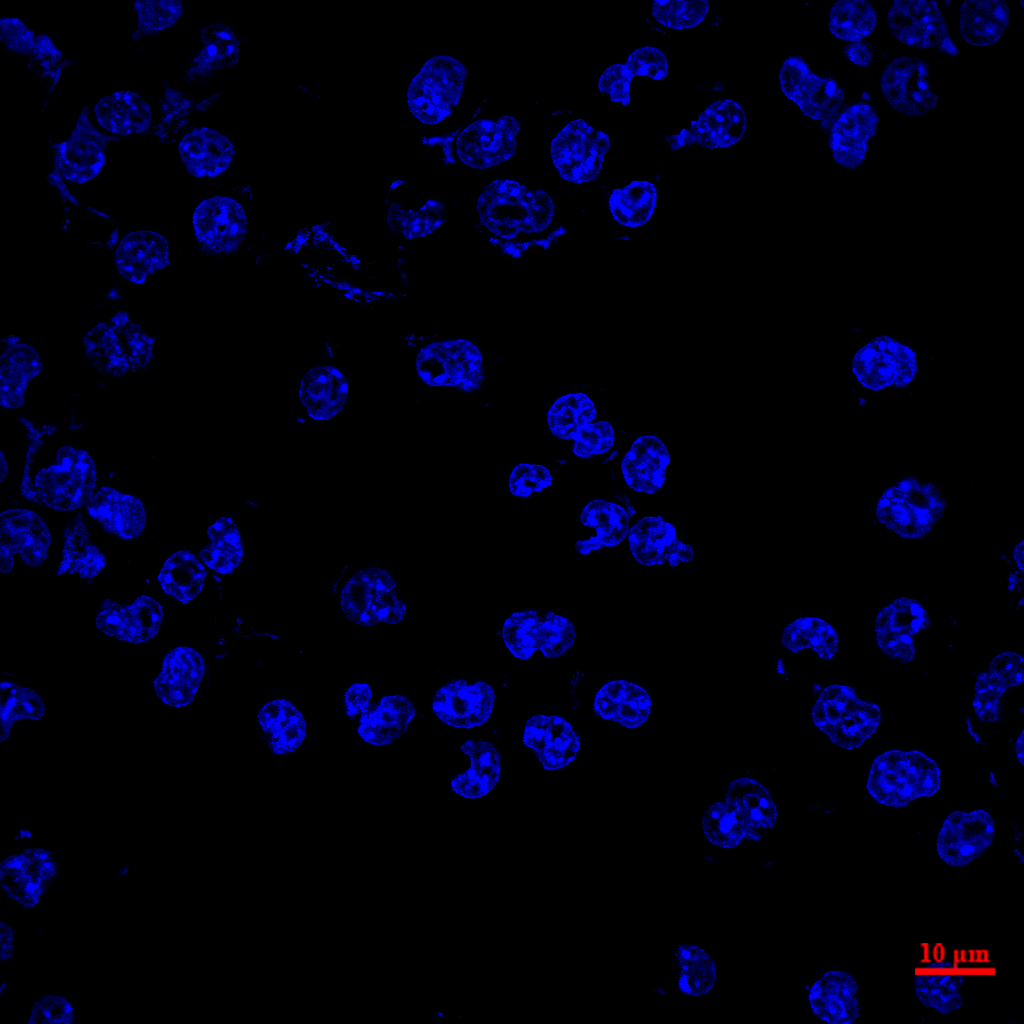

Supplement: Data S2 [file peerj-12-17664-s002.zip › raw data2/ROS+confocal/BV2+LPS_0002.tif.frames/BV2+LPS_0002_C001T001.tif]

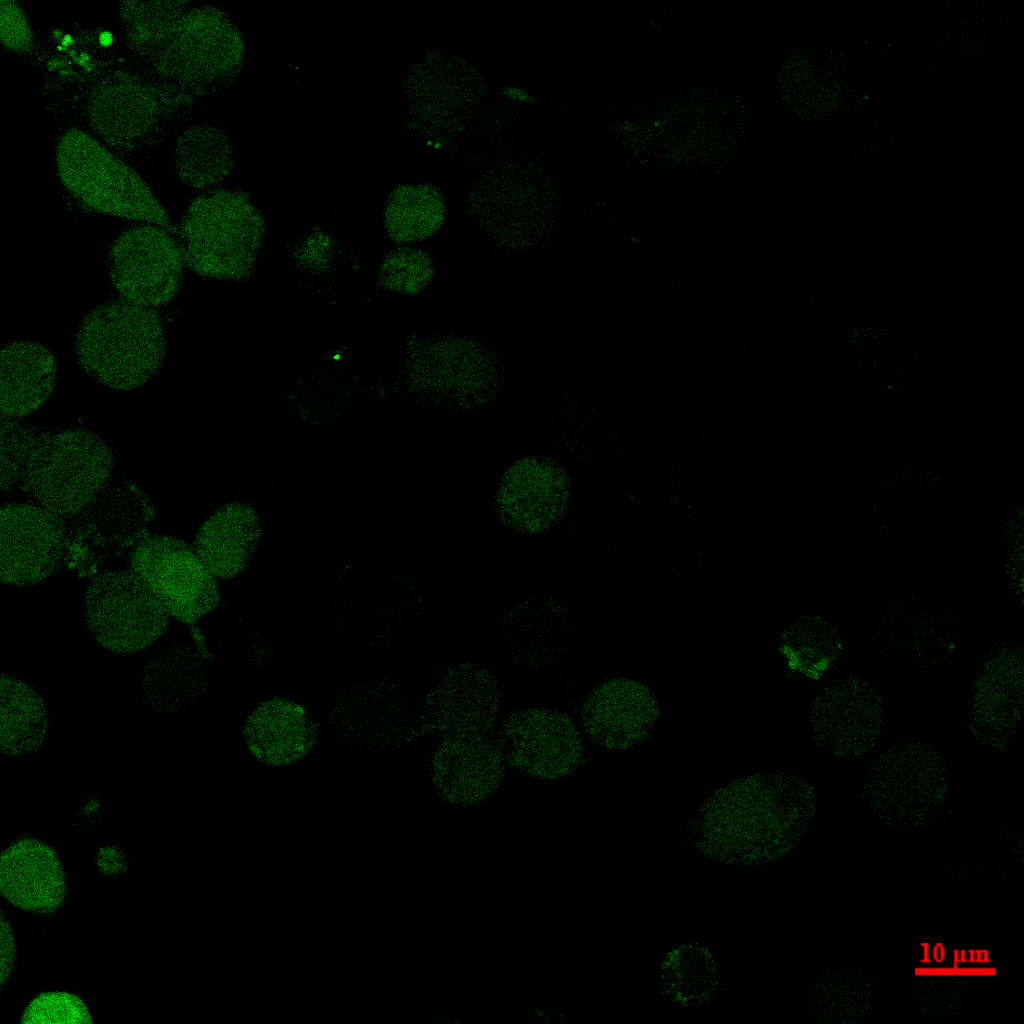

Supplement: Data S2 [file peerj-12-17664-s002.zip › raw data2/ROS+confocal/BV2+LPS_0002.tif.frames/BV2+LPS_0002_C002T001.tif]

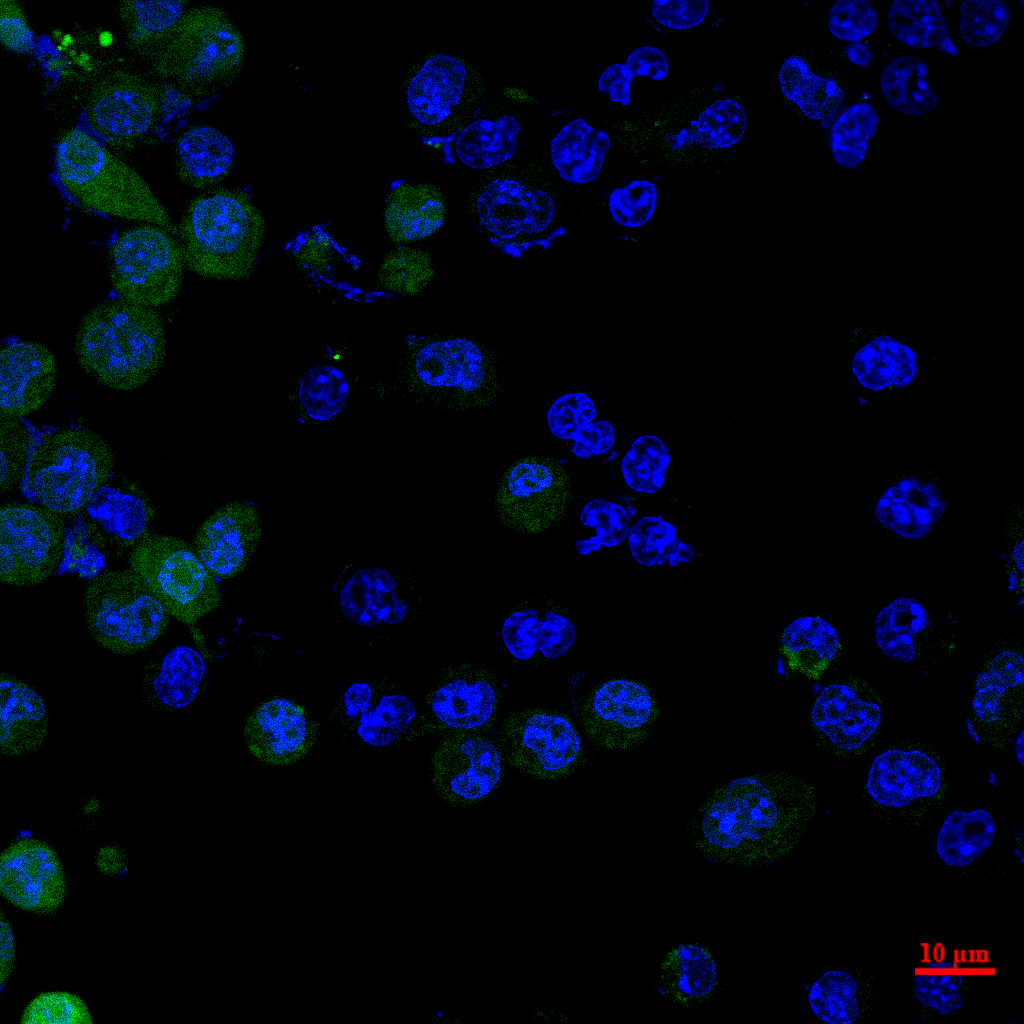

Supplement: Data S2 [file peerj-12-17664-s002.zip › raw data2/ROS+confocal/BV2+LPS_0002.tif.frames/BV2+LPS_0002_T001.tif]

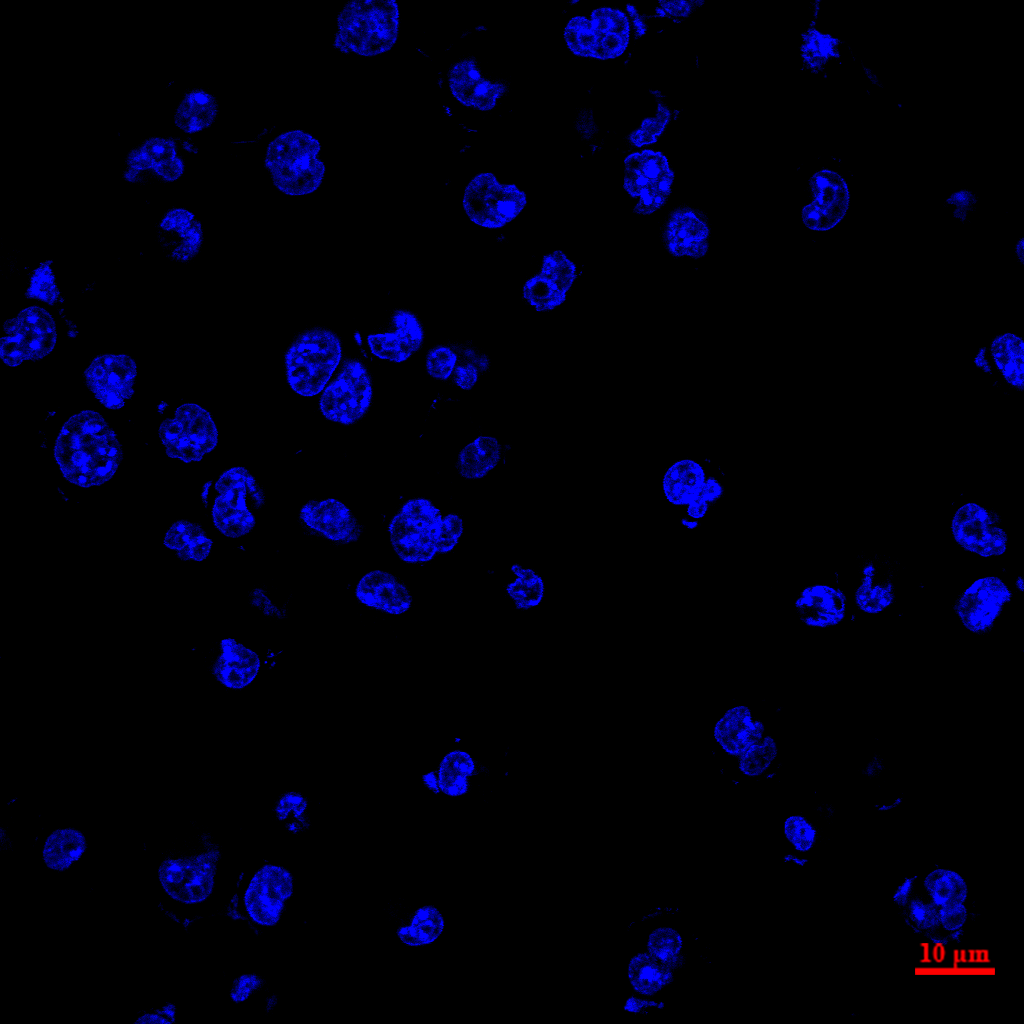

Supplement: Data S2 [file peerj-12-17664-s002.zip › raw data2/ROS+confocal/BV2+LPS_0003.tif.frames/BV2+LPS_0003_C001T001.tif]

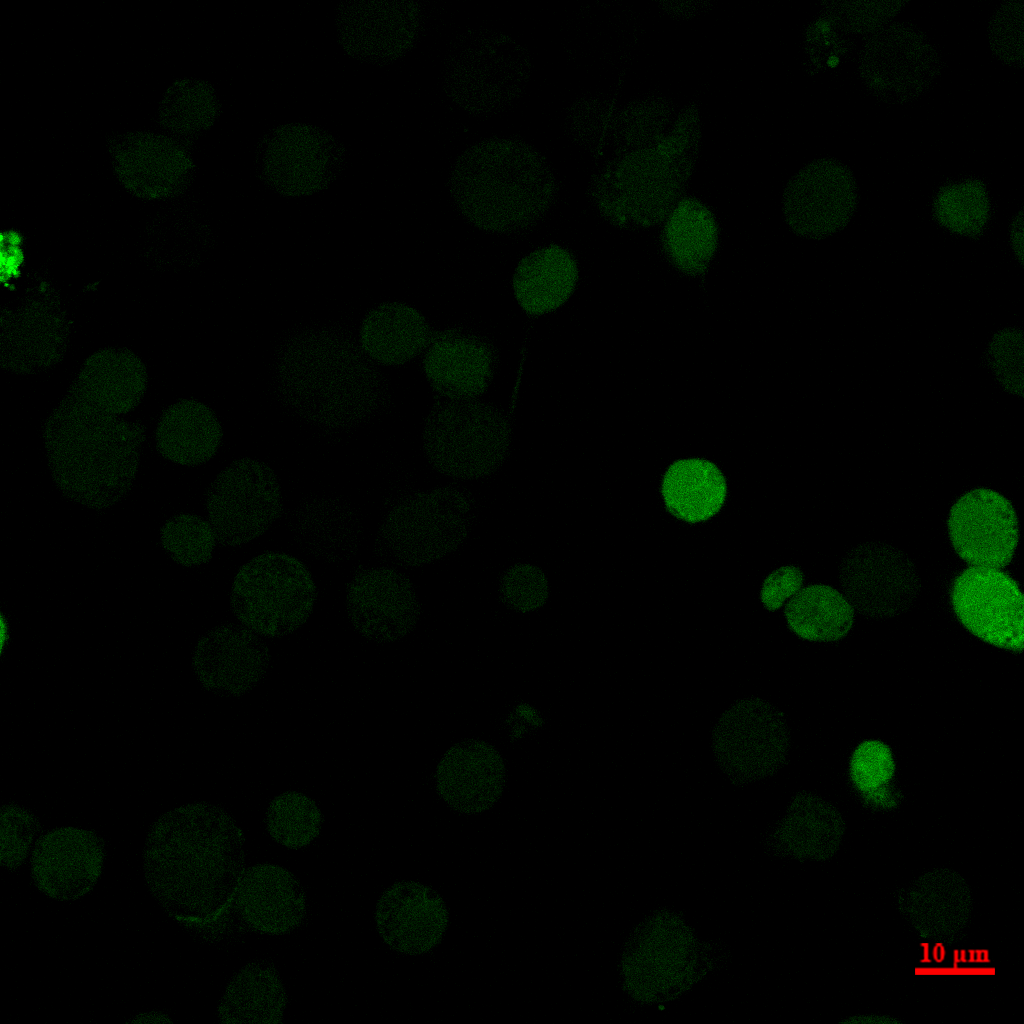

Supplement: Data S2 [file peerj-12-17664-s002.zip › raw data2/ROS+confocal/BV2+LPS_0003.tif.frames/BV2+LPS_0003_C002T001.tif]

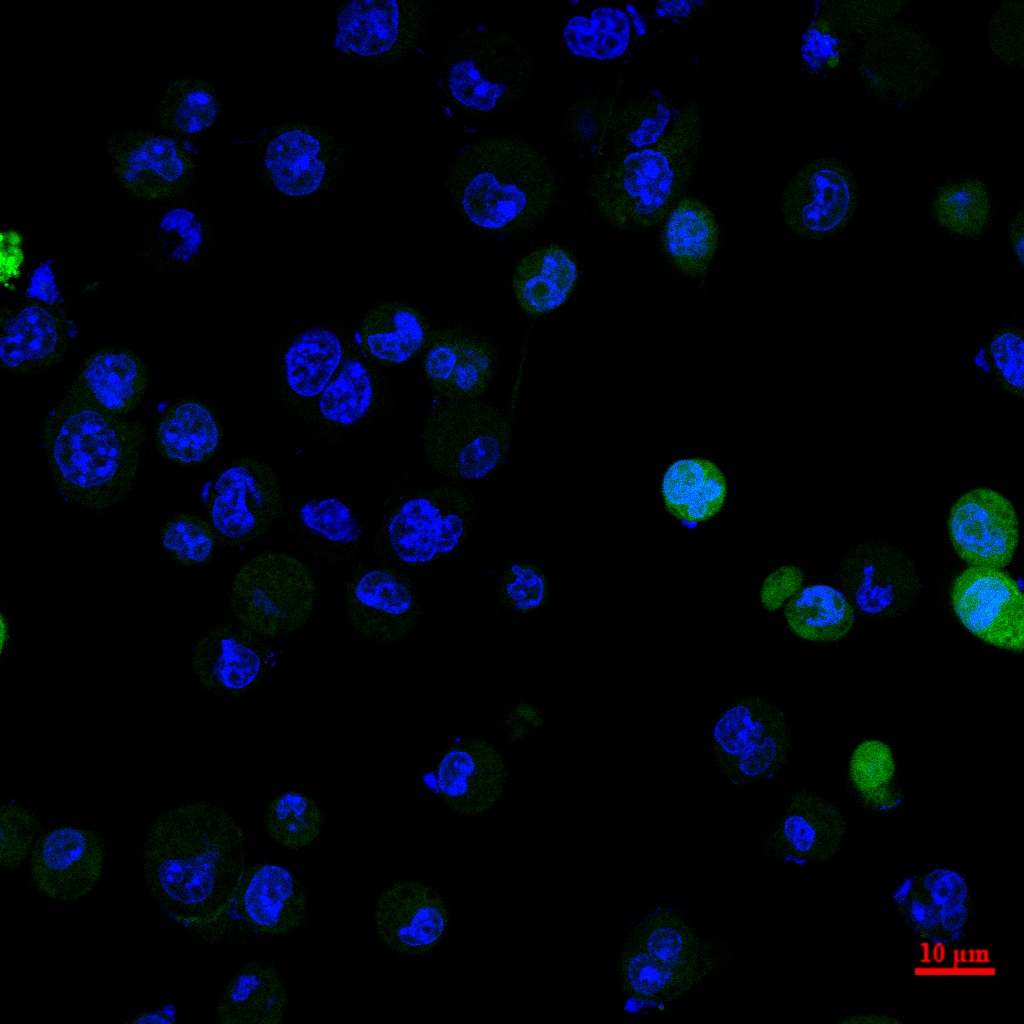

Supplement: Data S2 [file peerj-12-17664-s002.zip › raw data2/ROS+confocal/BV2+LPS_0003.tif.frames/BV2+LPS_0003_T001.tif]

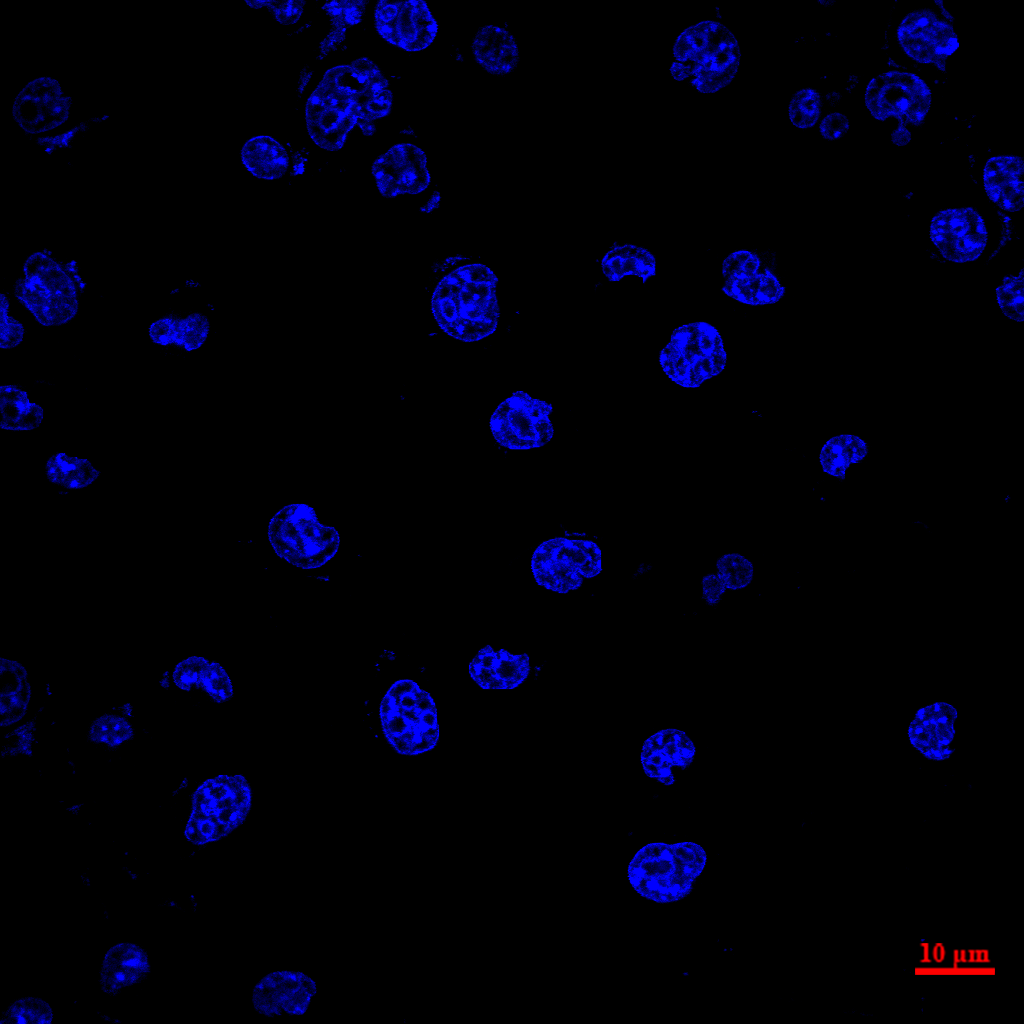

Supplement: Data S2 [file peerj-12-17664-s002.zip › raw data2/ROS+confocal/BV2+LPS+BMSC-CM_0001.tif.frames/BV2+LPS+BMSC-CM_0001_C001T001.tif]

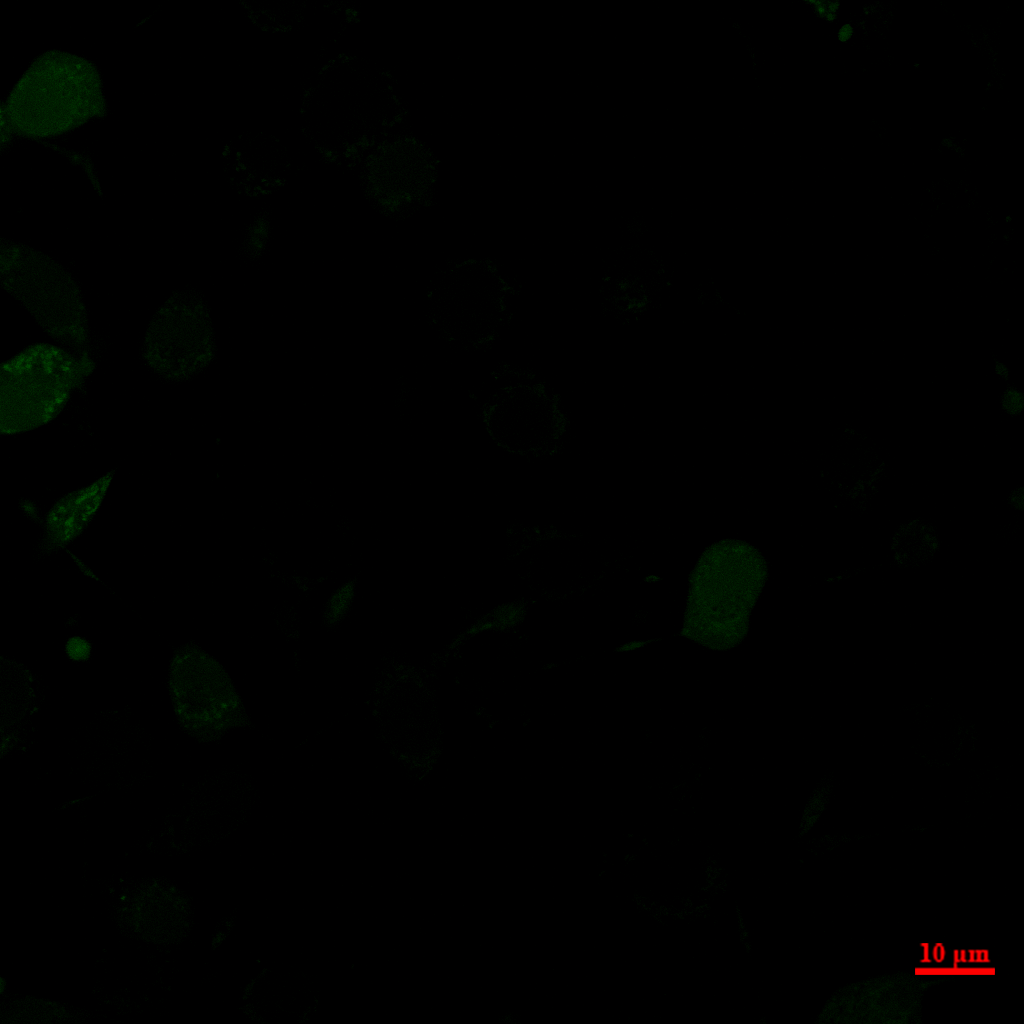

Supplement: Data S2 [file peerj-12-17664-s002.zip › raw data2/ROS+confocal/BV2+LPS+BMSC-CM_0001.tif.frames/BV2+LPS+BMSC-CM_0001_C002T001.tif]

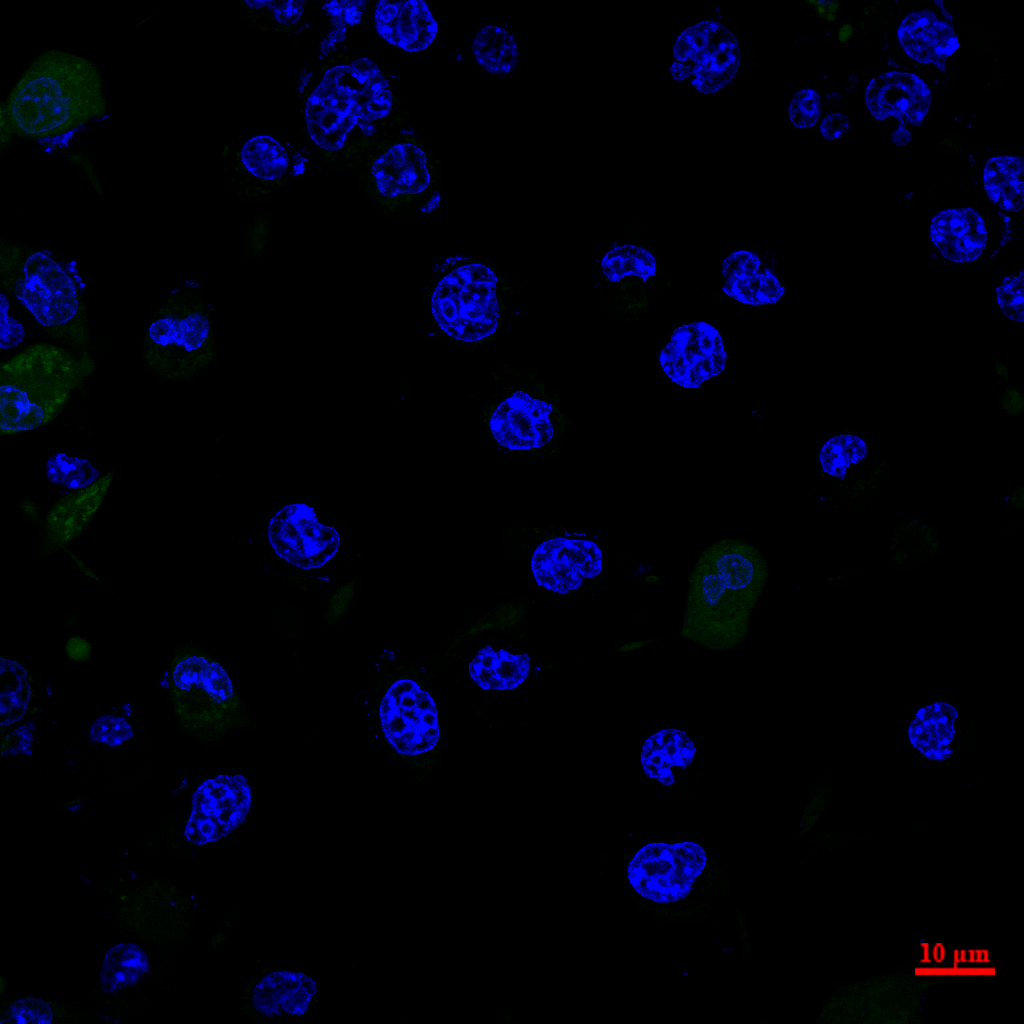

Supplement: Data S2 [file peerj-12-17664-s002.zip › raw data2/ROS+confocal/BV2+LPS+BMSC-CM_0001.tif.frames/BV2+LPS+BMSC-CM_0001_T001.tif]

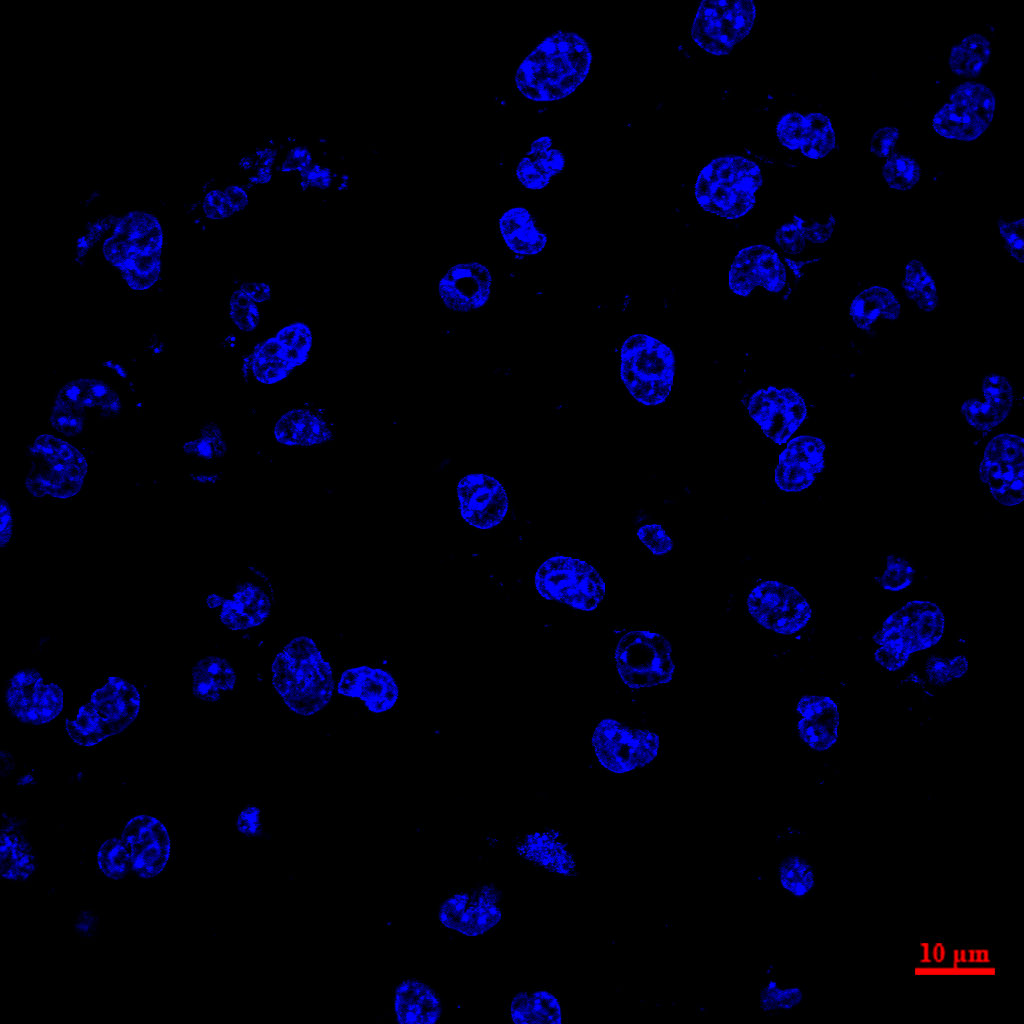

Supplement: Data S2 [file peerj-12-17664-s002.zip › raw data2/ROS+confocal/BV2+LPS+BMSC-CM_0002.tif.frames/BV2+LPS+BMSC-CM_0002_C001T001.tif]

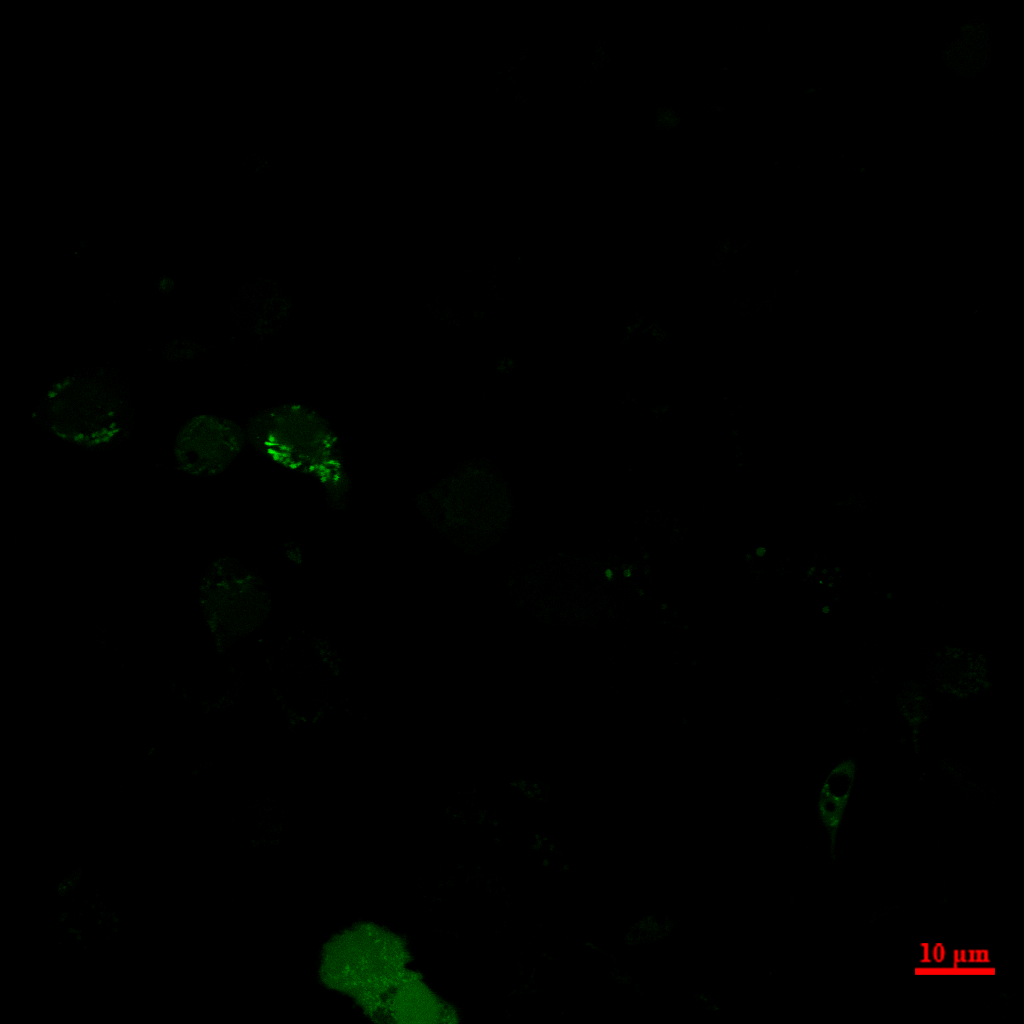

Supplement: Data S2 [file peerj-12-17664-s002.zip › raw data2/ROS+confocal/BV2+LPS+BMSC-CM_0002.tif.frames/BV2+LPS+BMSC-CM_0002_C002T001.tif]

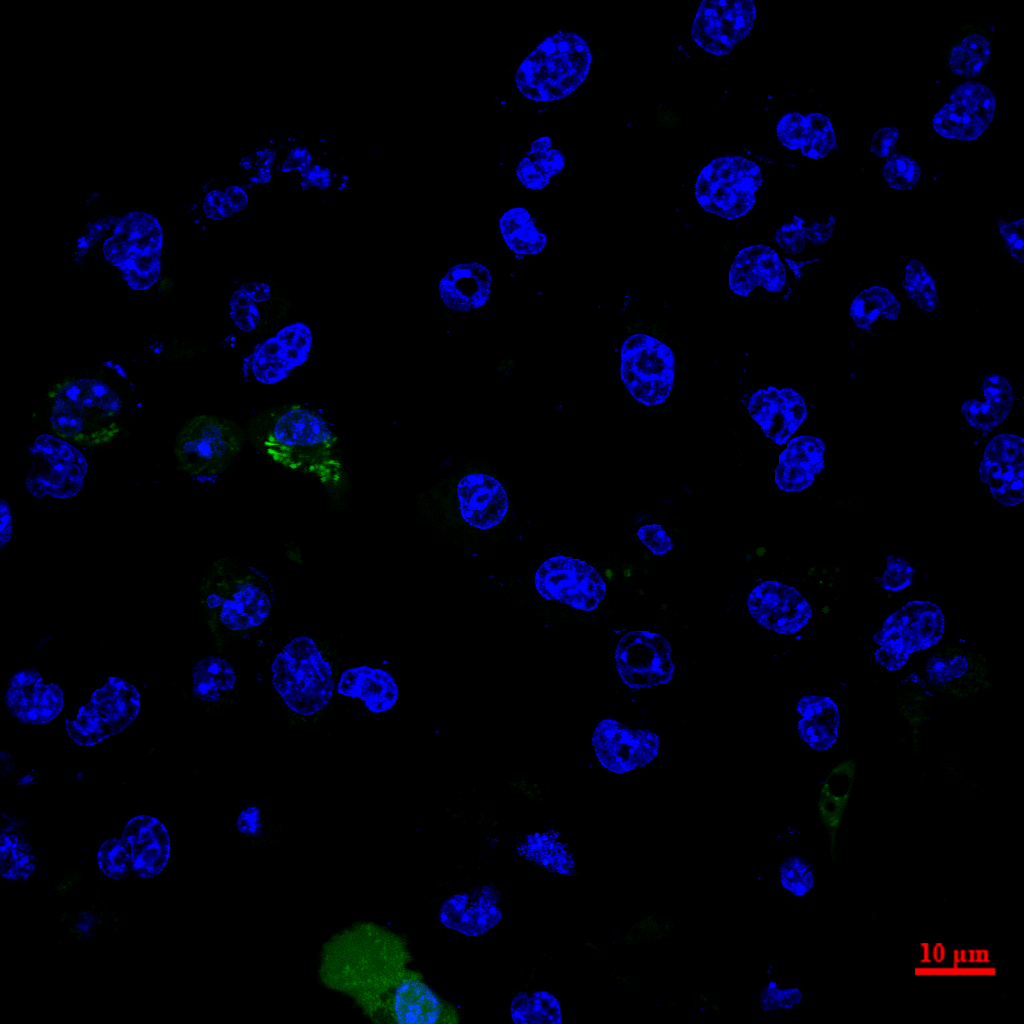

Supplement: Data S2 [file peerj-12-17664-s002.zip › raw data2/ROS+confocal/BV2+LPS+BMSC-CM_0002.tif.frames/BV2+LPS+BMSC-CM_0002_T001.tif]

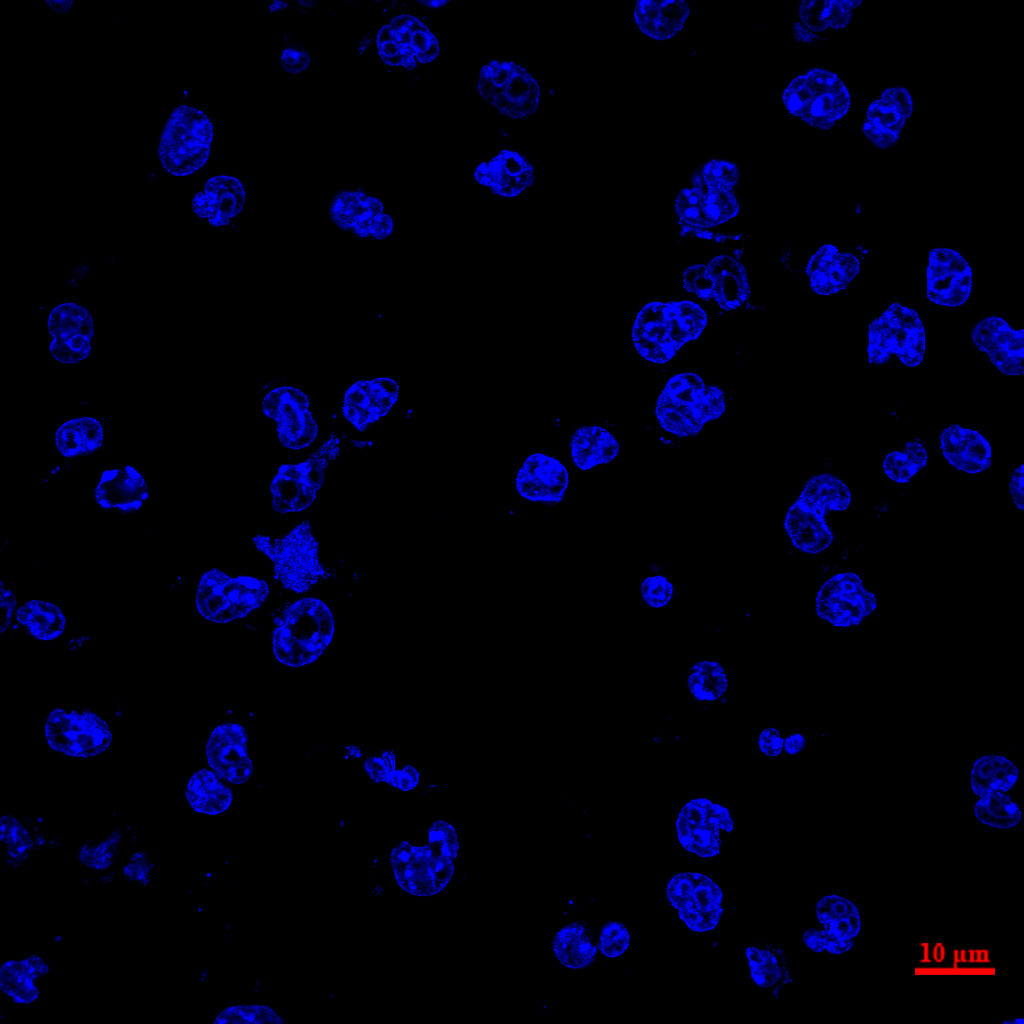

Supplement: Data S2 [file peerj-12-17664-s002.zip › raw data2/ROS+confocal/BV2+LPS+BMSC-CM_0003.tif.frames/BV2+LPS+BMSC-CM_0003_C001T001.tif]

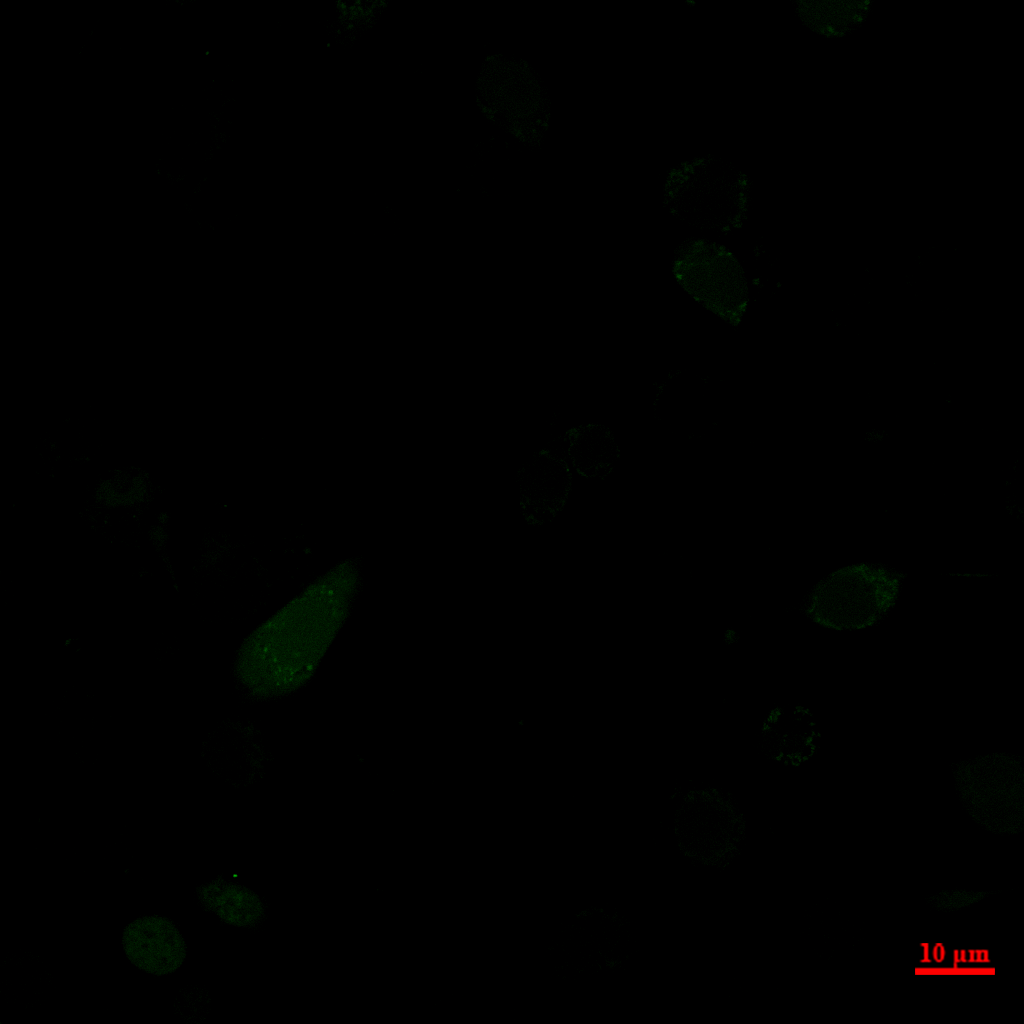

Supplement: Data S2 [file peerj-12-17664-s002.zip › raw data2/ROS+confocal/BV2+LPS+BMSC-CM_0003.tif.frames/BV2+LPS+BMSC-CM_0003_C002T001.tif]

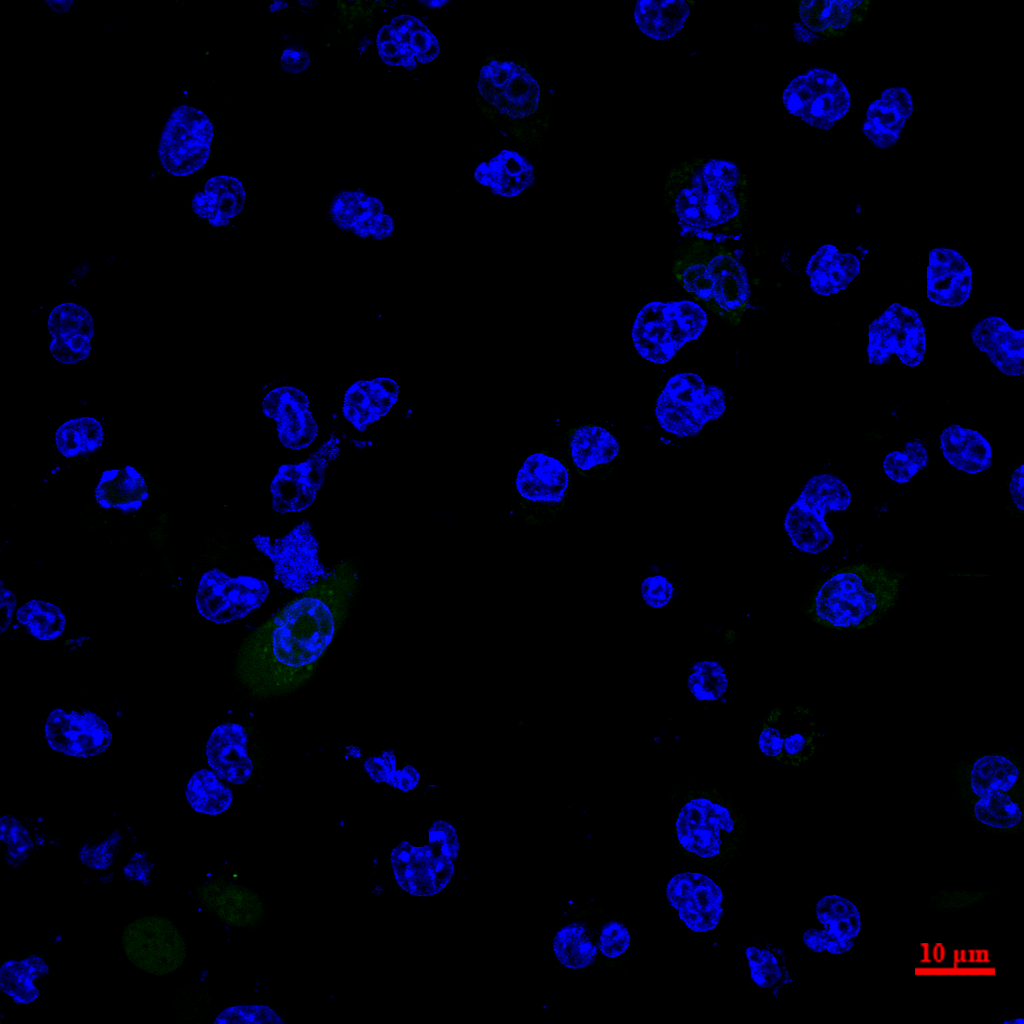

Supplement: Data S2 [file peerj-12-17664-s002.zip › raw data2/ROS+confocal/BV2+LPS+BMSC-CM_0003.tif.frames/BV2+LPS+BMSC-CM_0003_T001.tif]

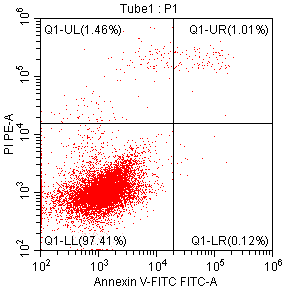

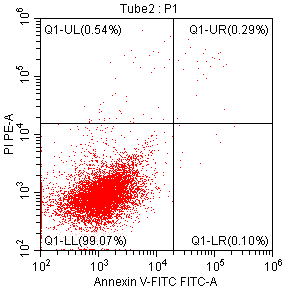

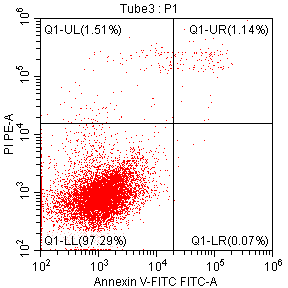

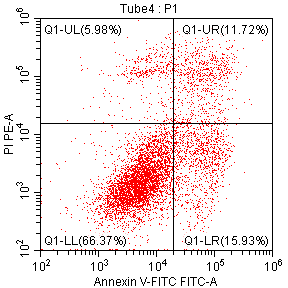

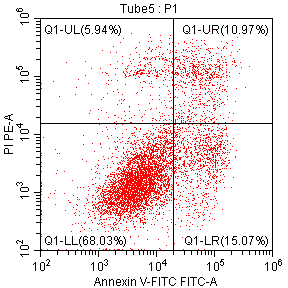

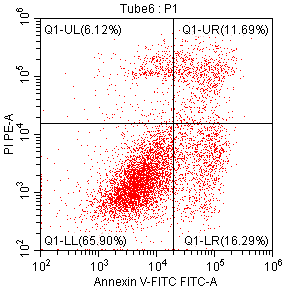

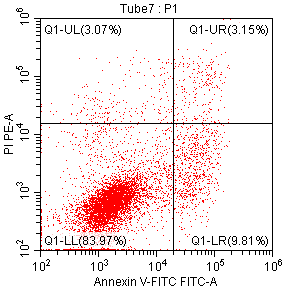

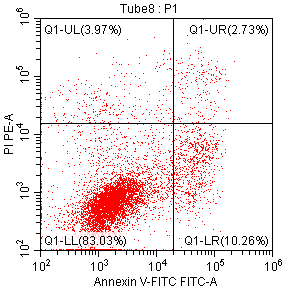

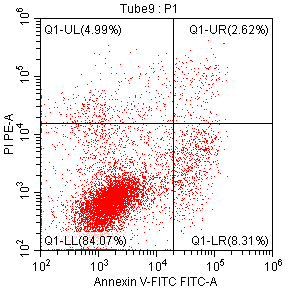

Supplement: Data S3 [file peerj-12-17664-s003.zip › raw data3/apoptosis/result.docx]

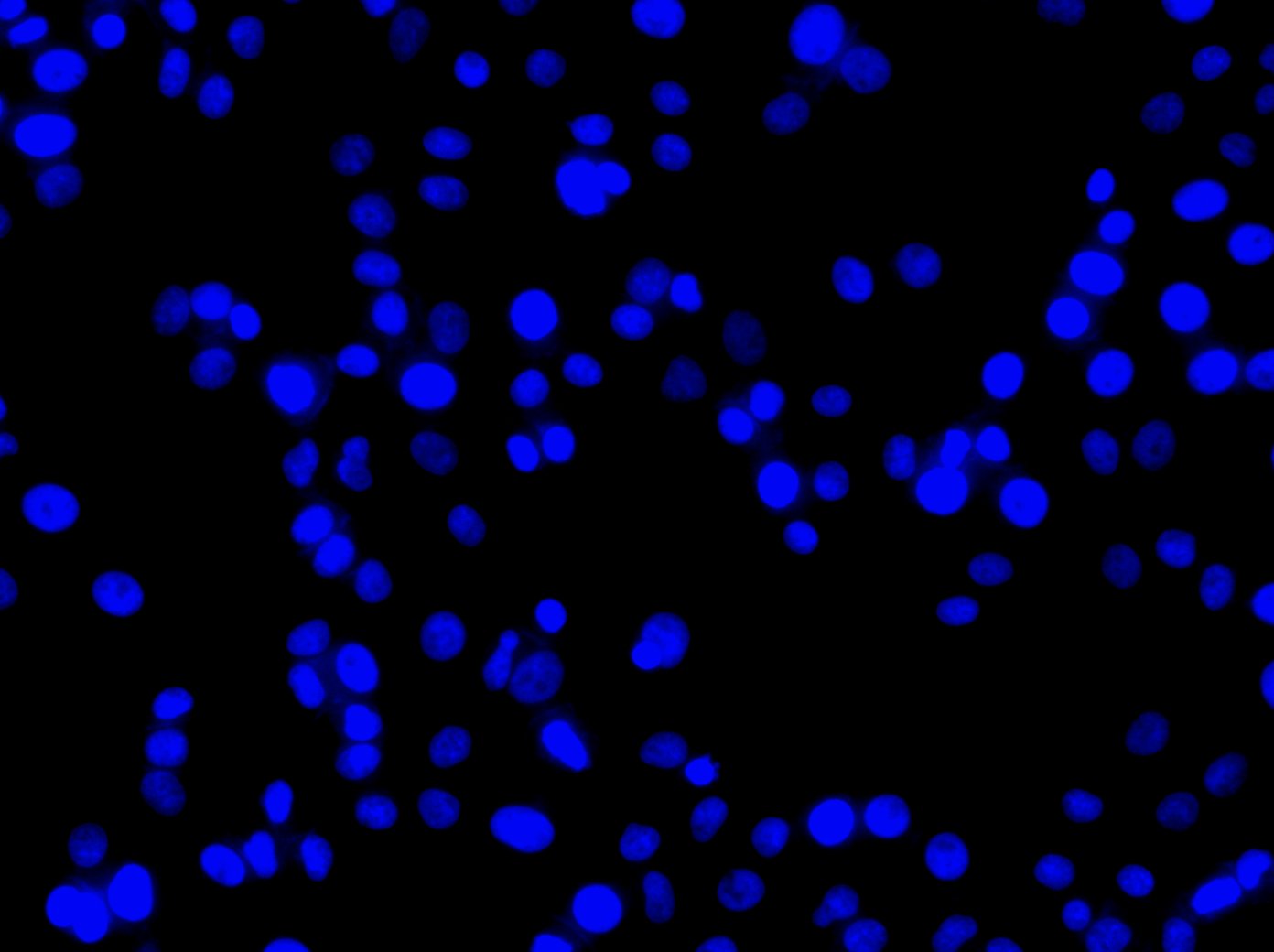

Supplement: Data S3 [file peerj-12-17664-s003.zip › raw data3/EDU/BV2_C8-D1A (1).jpg]

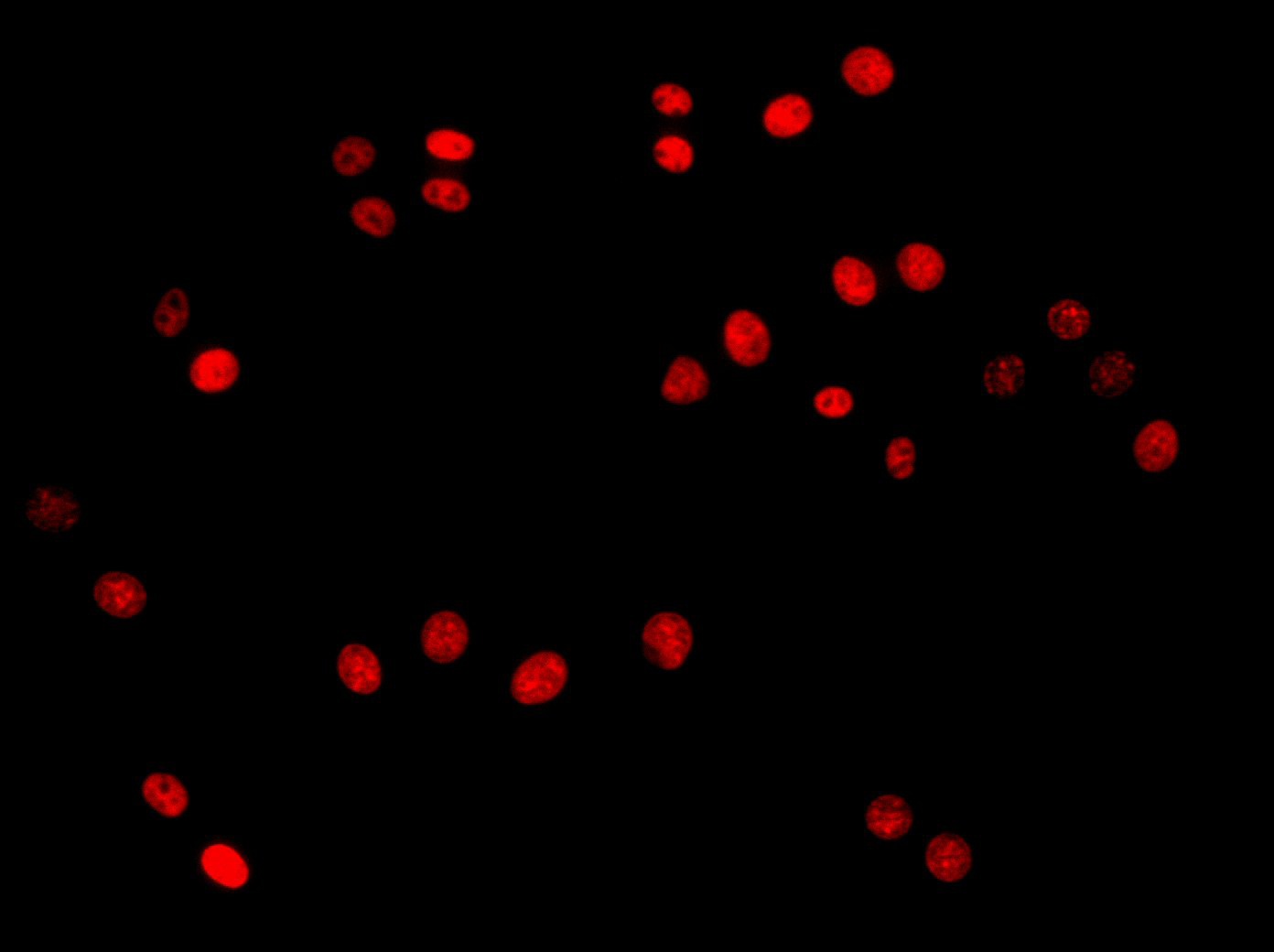

Supplement: Data S3 [file peerj-12-17664-s003.zip › raw data3/EDU/BV2_C8-D1A (2).jpg]

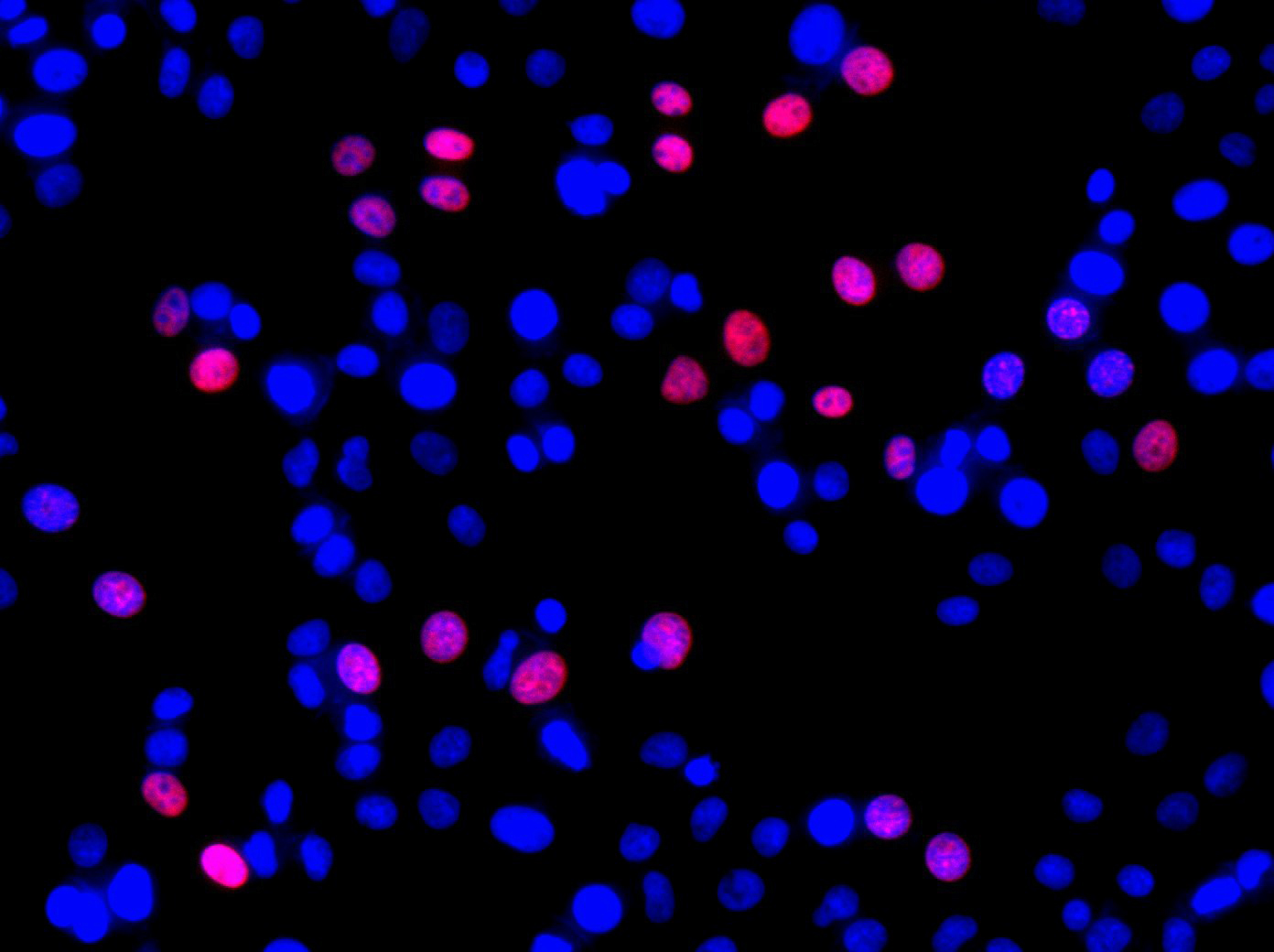

Supplement: Data S3 [file peerj-12-17664-s003.zip › raw data3/EDU/BV2_C8-D1A (3).jpg]

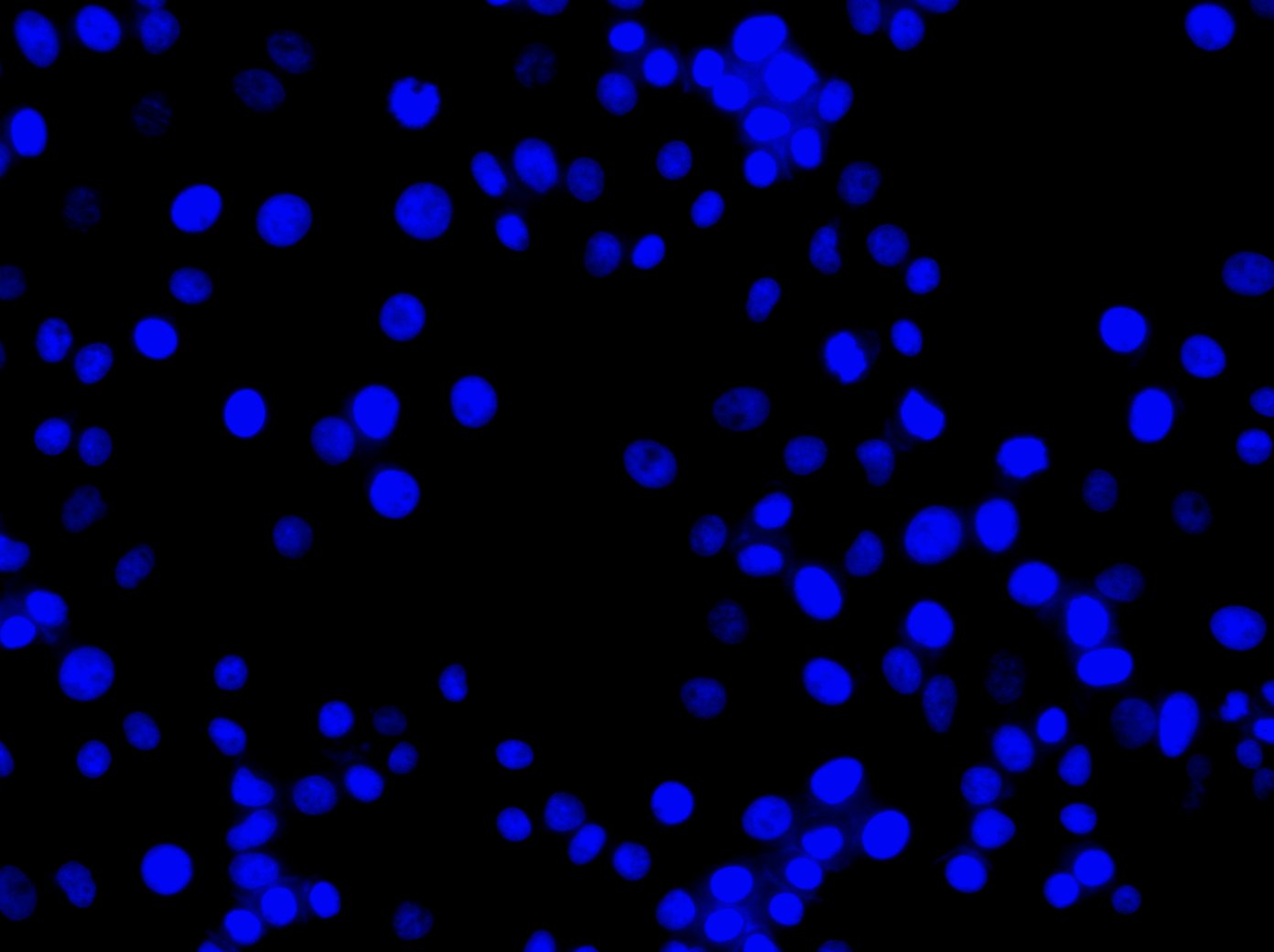

Supplement: Data S3 [file peerj-12-17664-s003.zip › raw data3/EDU/BV2_C8-D1A (4).jpg]

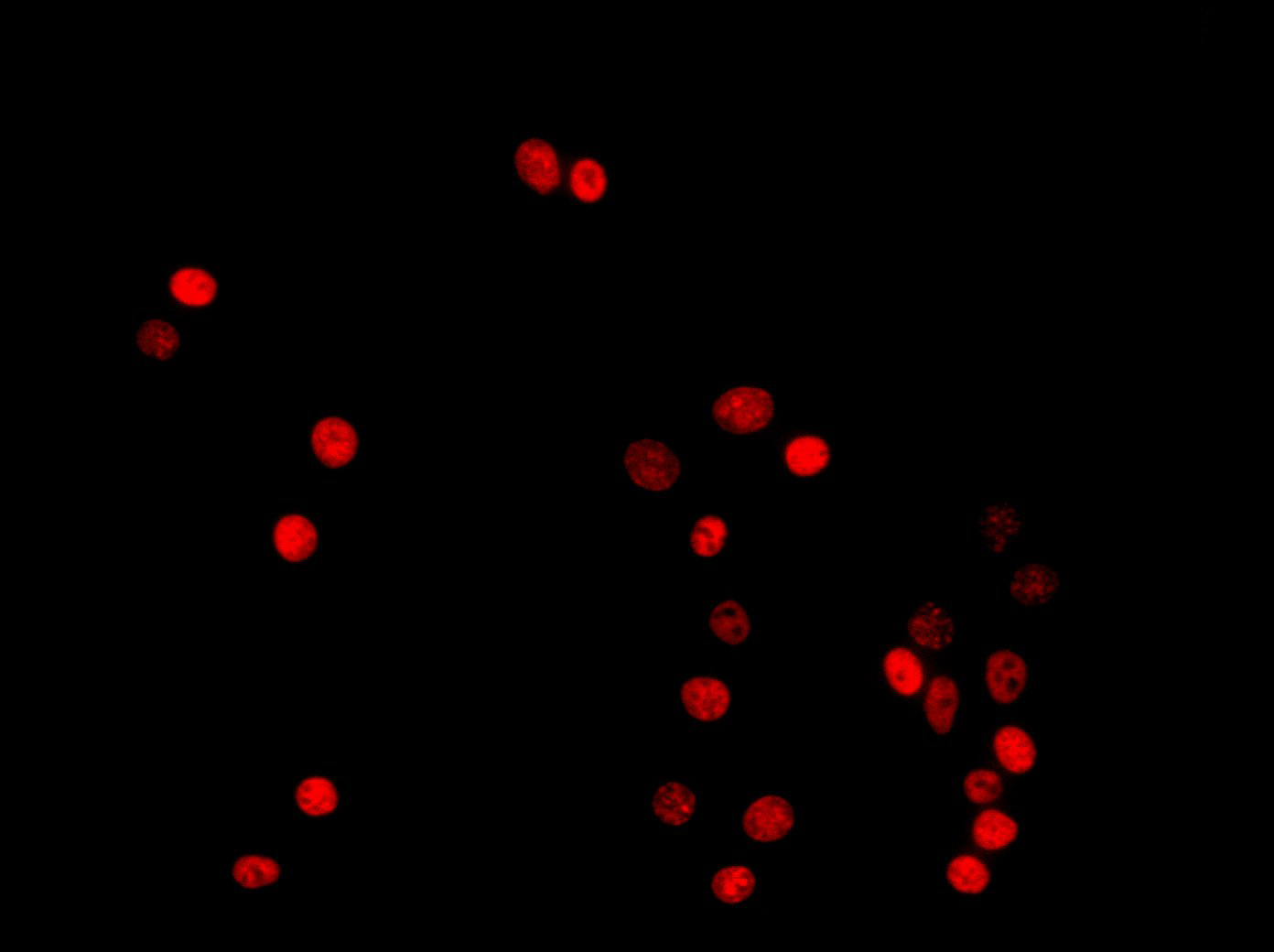

Supplement: Data S3 [file peerj-12-17664-s003.zip › raw data3/EDU/BV2_C8-D1A (5).jpg]

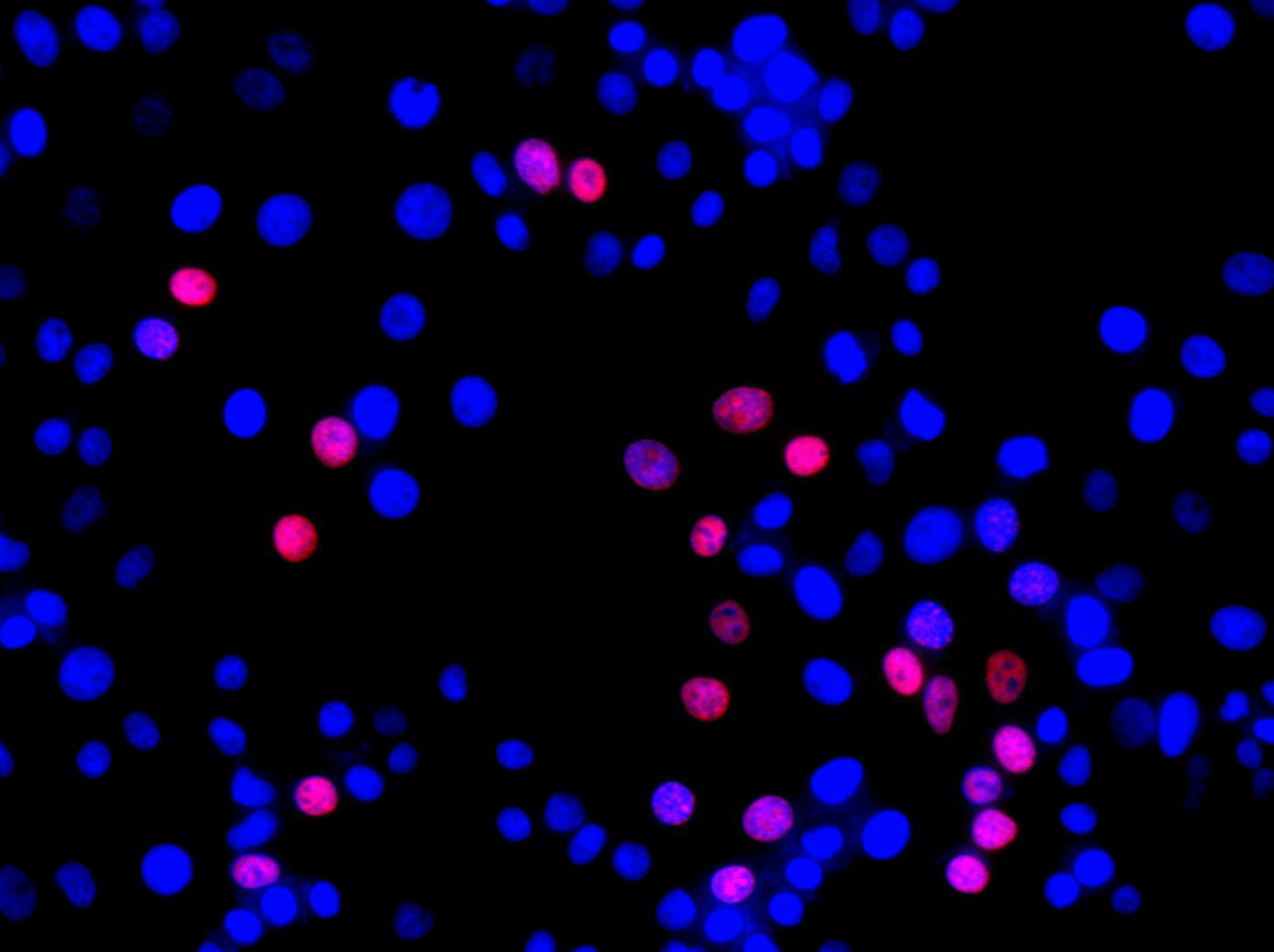

Supplement: Data S3 [file peerj-12-17664-s003.zip › raw data3/EDU/BV2_C8-D1A (6).jpg]

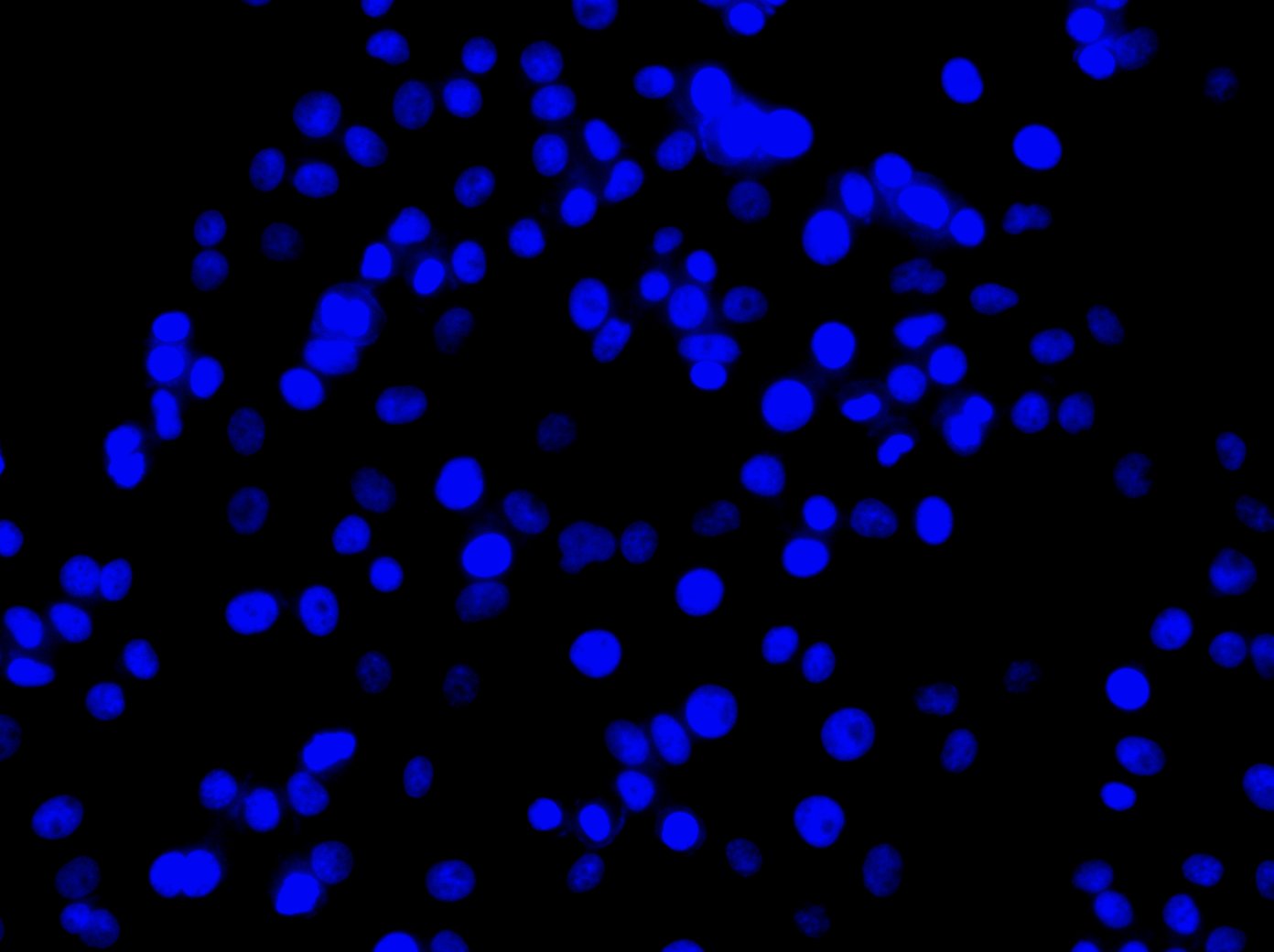

Supplement: Data S3 [file peerj-12-17664-s003.zip › raw data3/EDU/BV2_C8-D1A (7).jpg]
