# Supplementary figures and images for: Conditioned culture medium of bone marrow mesenchymal stem cells promotes phenotypic transformation of microglia by regulating mitochondrial autophagy (part 2 of 2)
Source: PeerJ. 2024 Jul 4;12:e17664. doi: 10.7717/peerj.17664 (PMC11227809; doi:10.7717/peerj.17664)

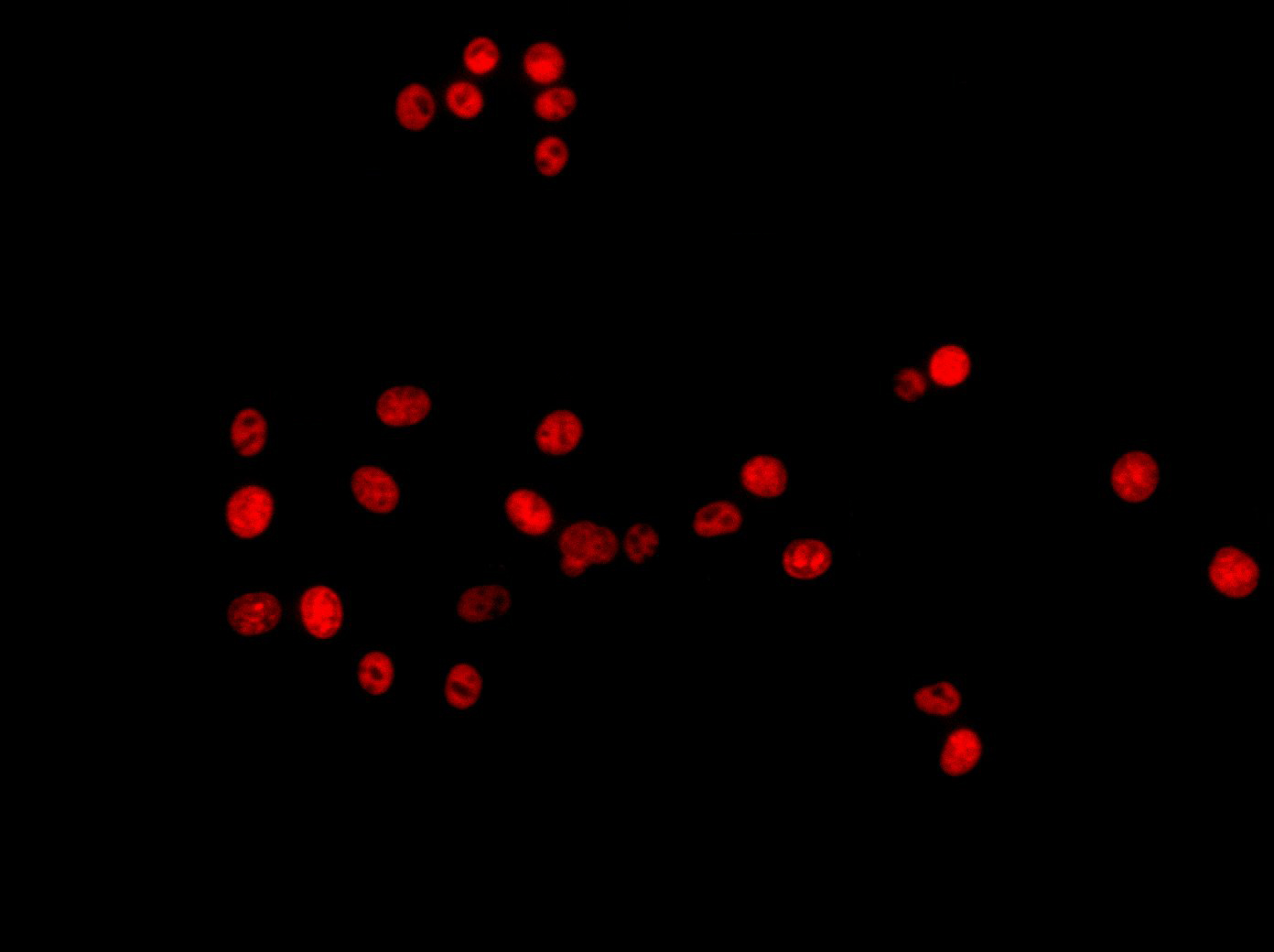

Supplement: Data S3 [file peerj-12-17664-s003.zip › raw data3/EDU/BV2_C8-D1A (8).jpg]

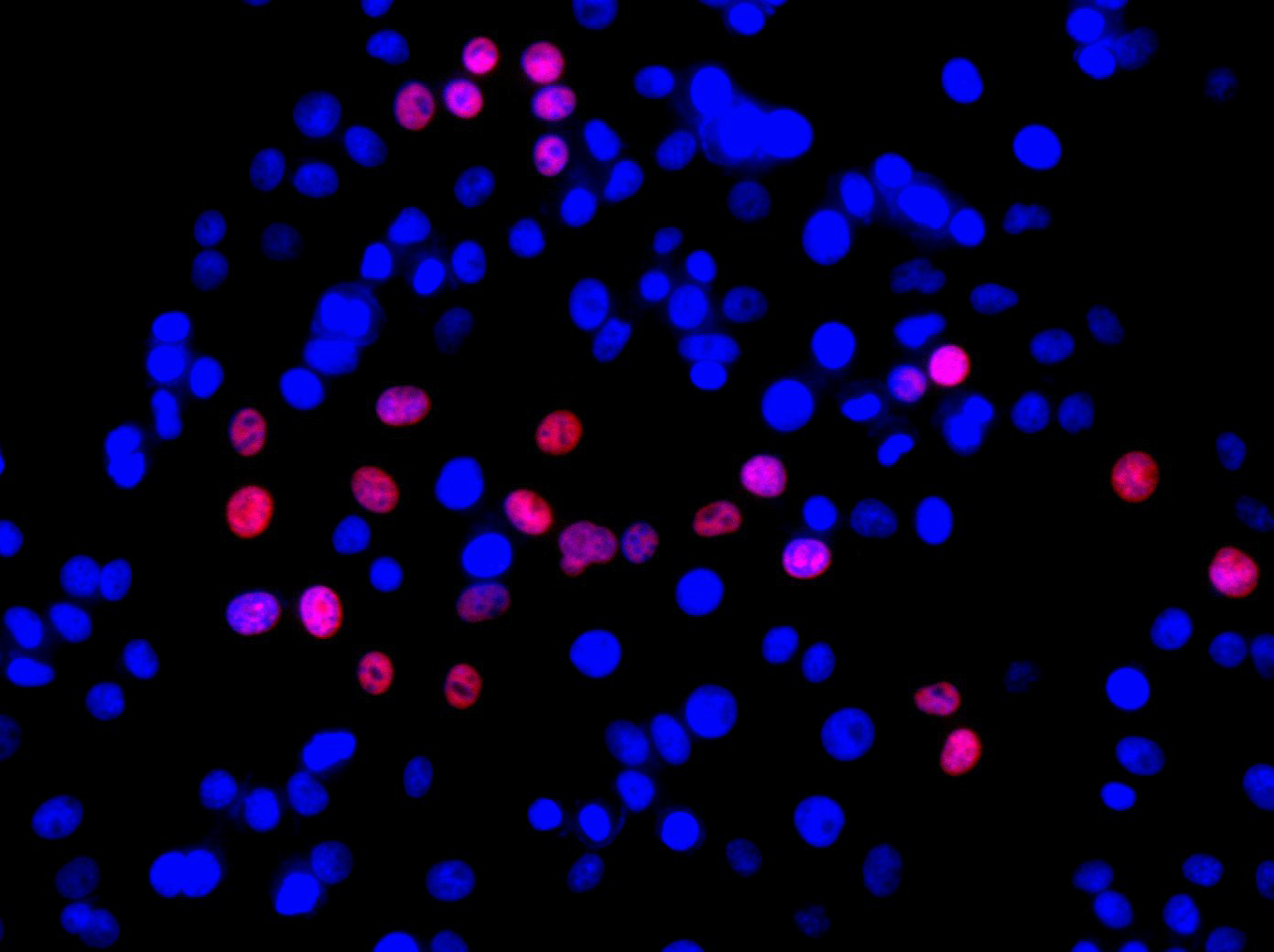

Supplement: Data S3 [file peerj-12-17664-s003.zip › raw data3/EDU/BV2_C8-D1A (9).jpg]

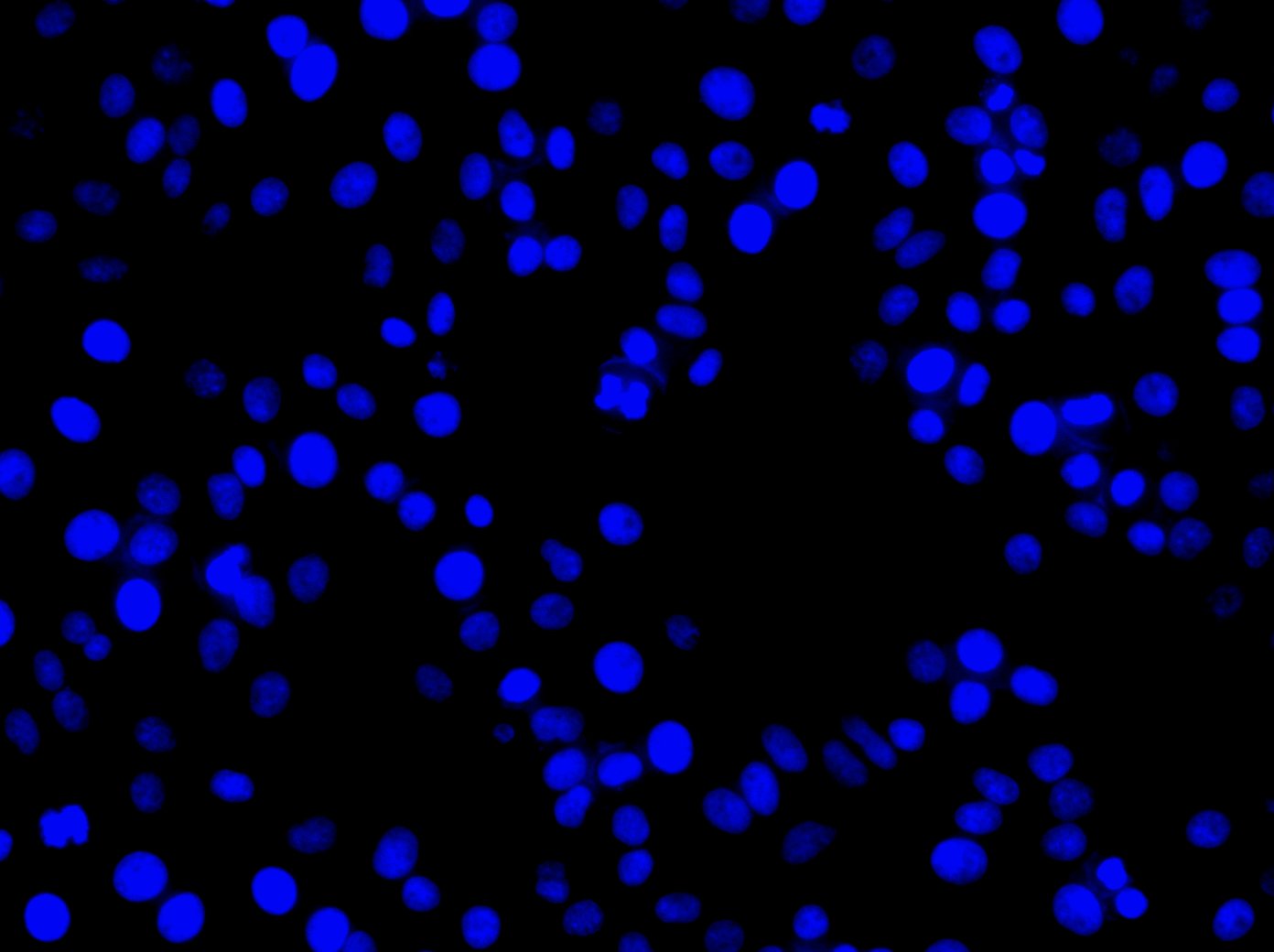

Supplement: Data S3 [file peerj-12-17664-s003.zip › raw data3/EDU/BV2+LPS_C8-D1A (1).jpg]

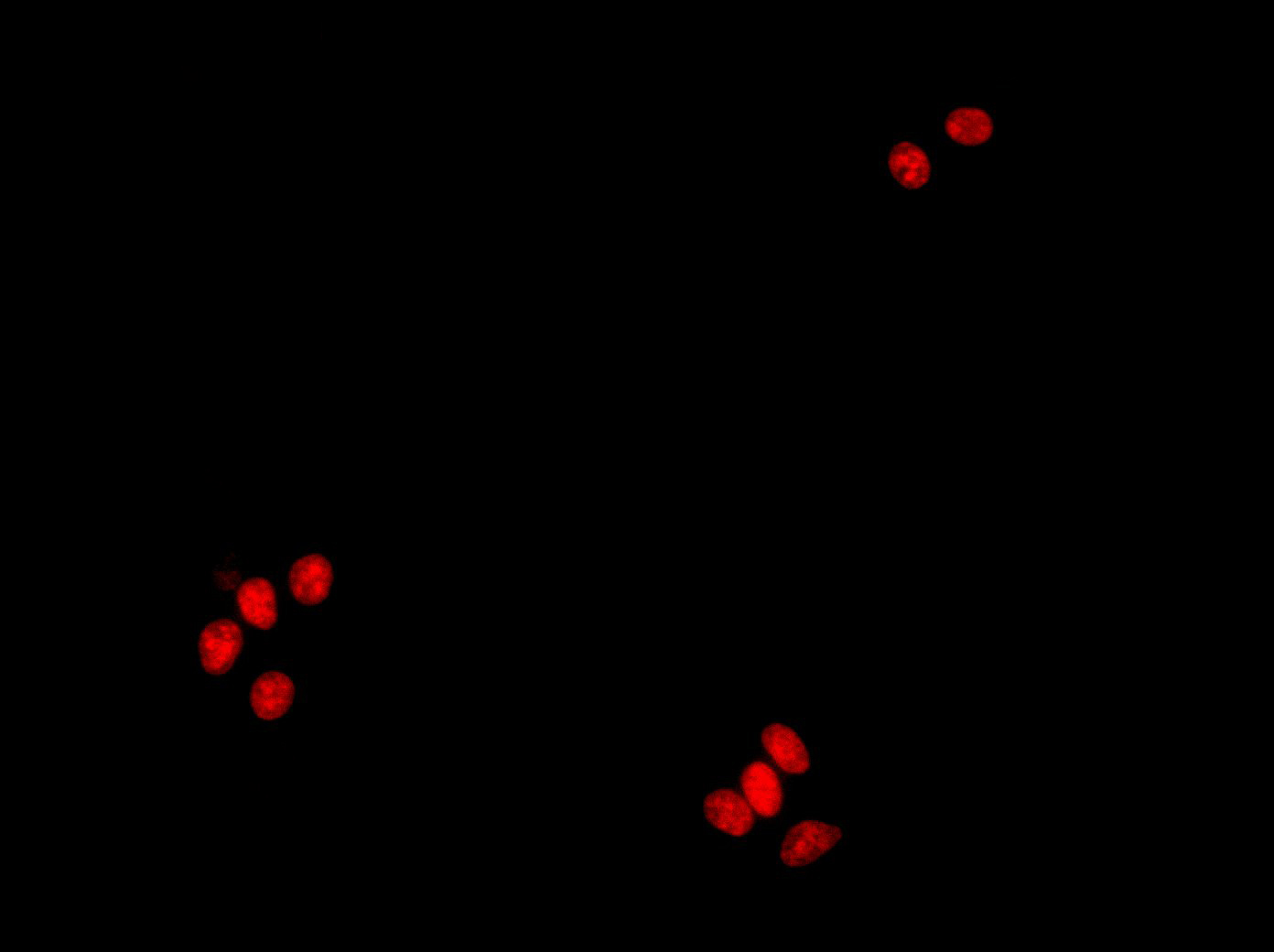

Supplement: Data S3 [file peerj-12-17664-s003.zip › raw data3/EDU/BV2+LPS_C8-D1A (2).jpg]

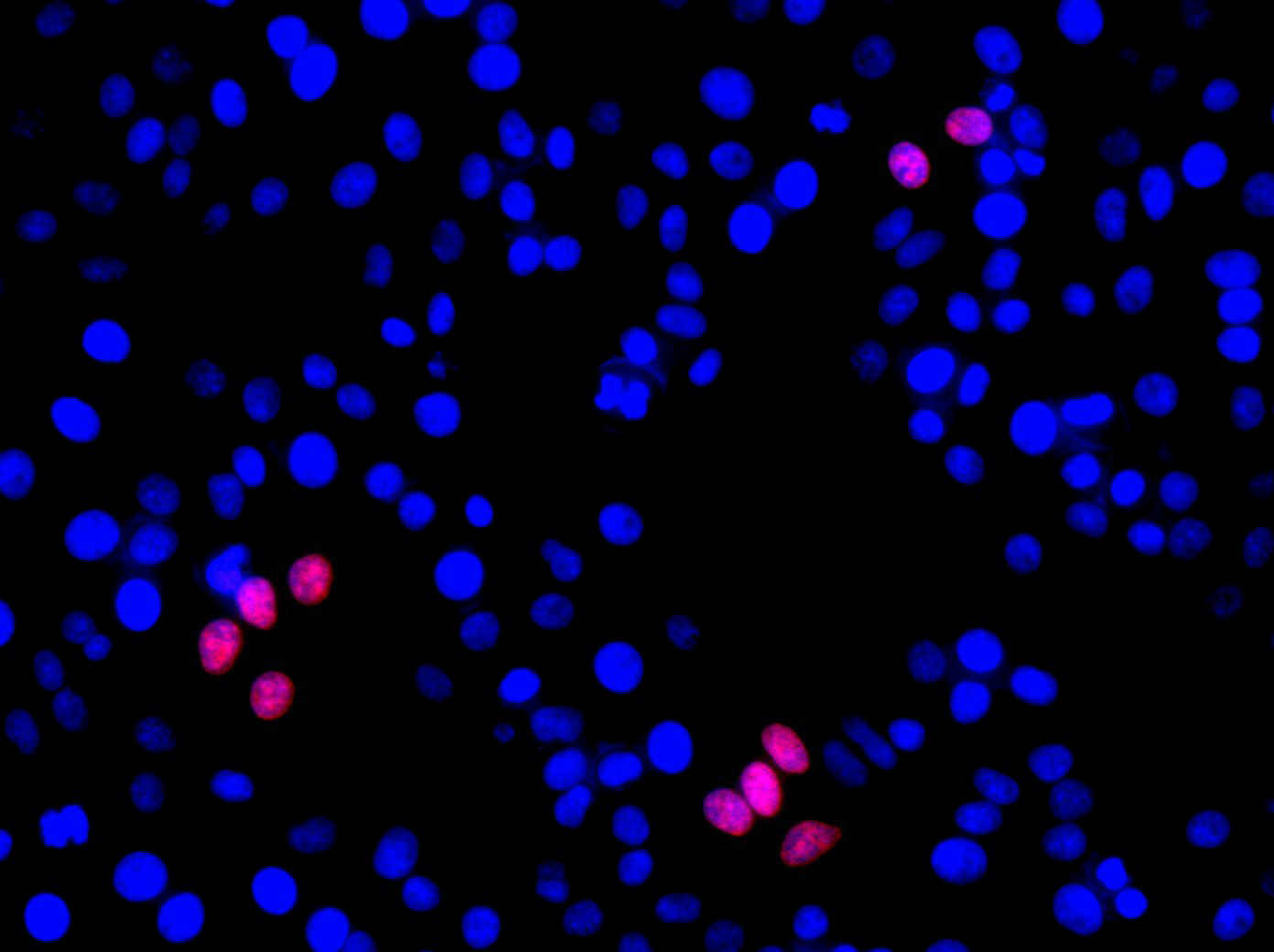

Supplement: Data S3 [file peerj-12-17664-s003.zip › raw data3/EDU/BV2+LPS_C8-D1A (3).jpg]

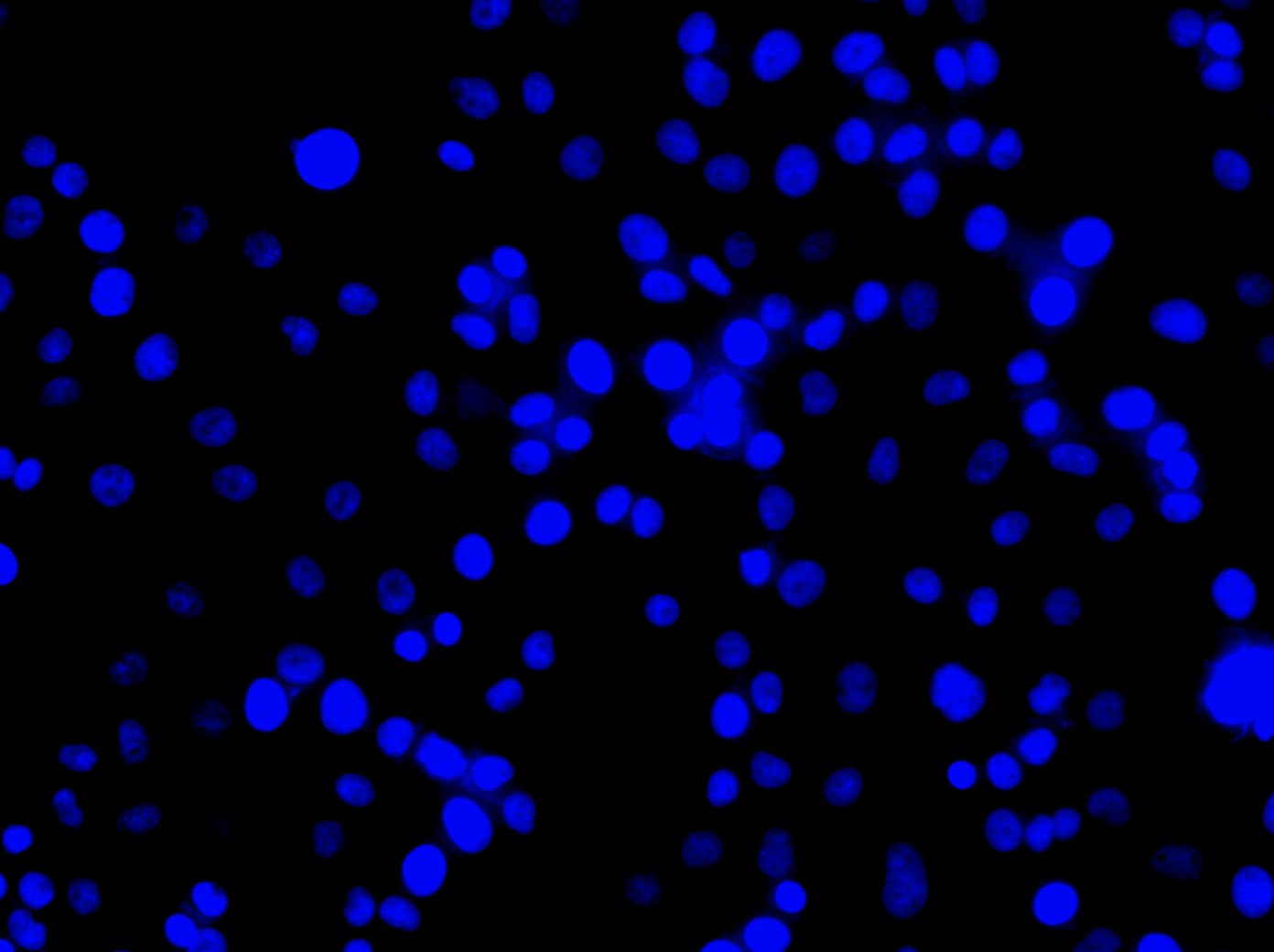

Supplement: Data S3 [file peerj-12-17664-s003.zip › raw data3/EDU/BV2+LPS_C8-D1A (4).jpg]

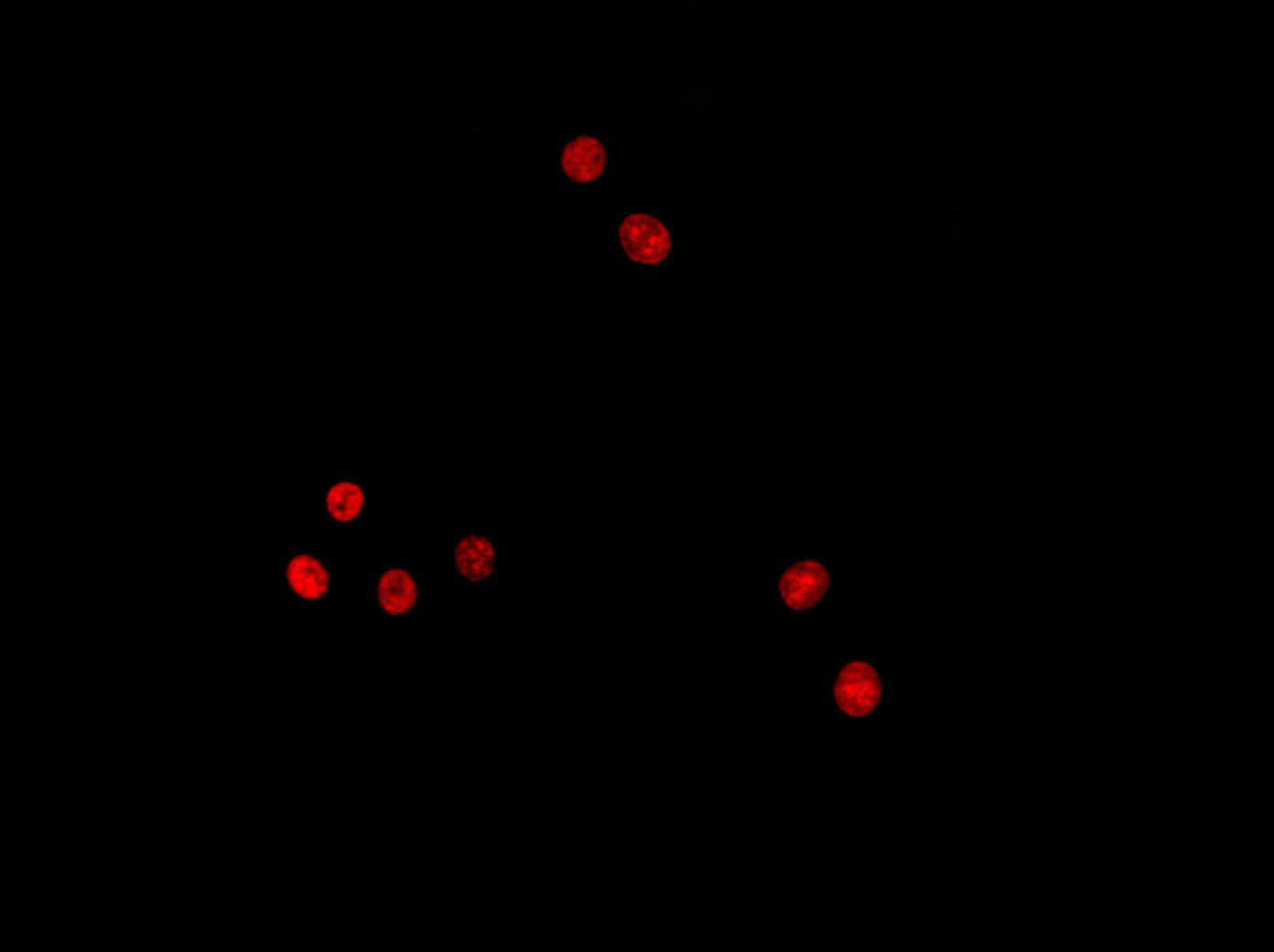

Supplement: Data S3 [file peerj-12-17664-s003.zip › raw data3/EDU/BV2+LPS_C8-D1A (5).jpg]

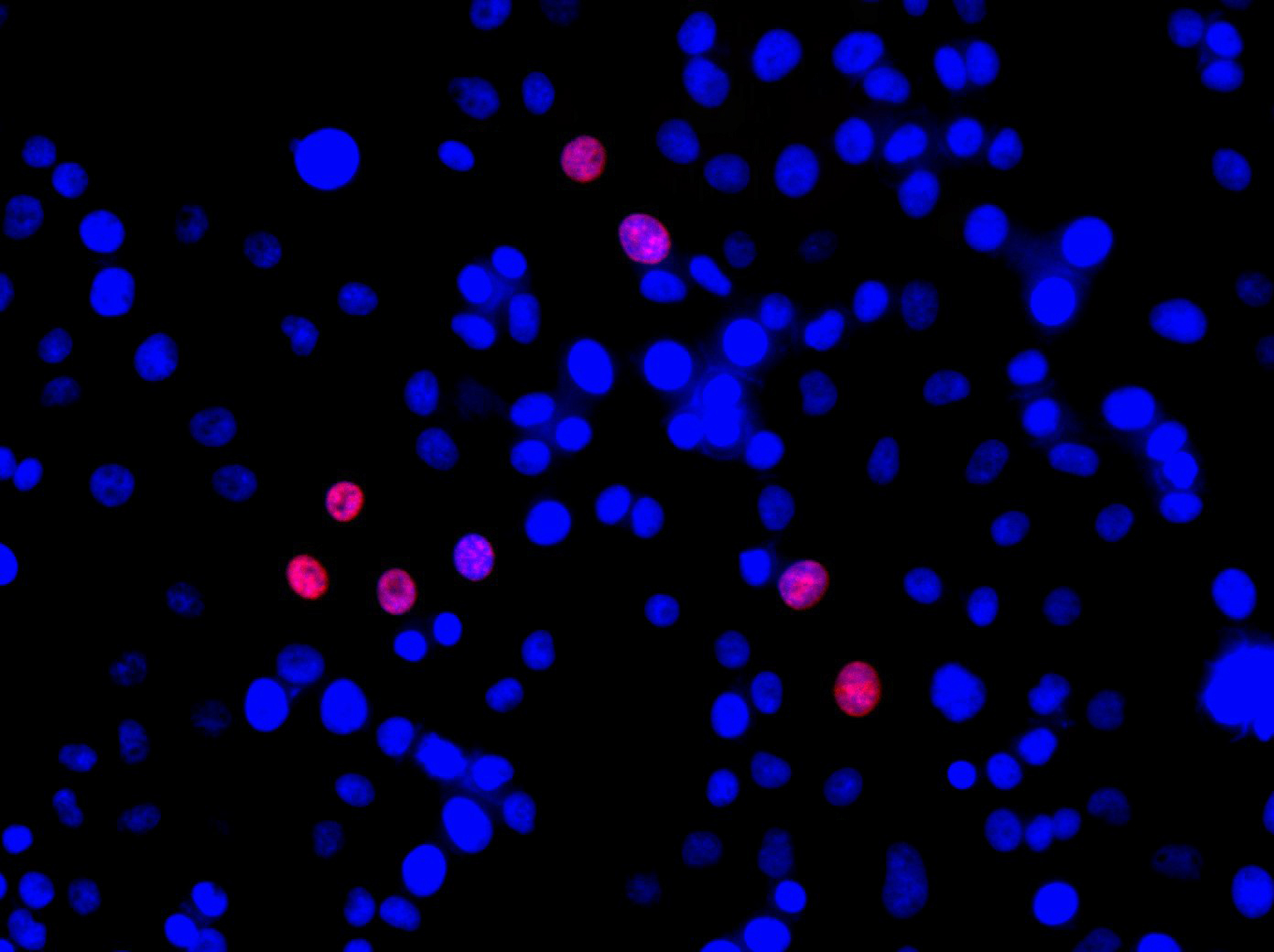

Supplement: Data S3 [file peerj-12-17664-s003.zip › raw data3/EDU/BV2+LPS_C8-D1A (6).jpg]

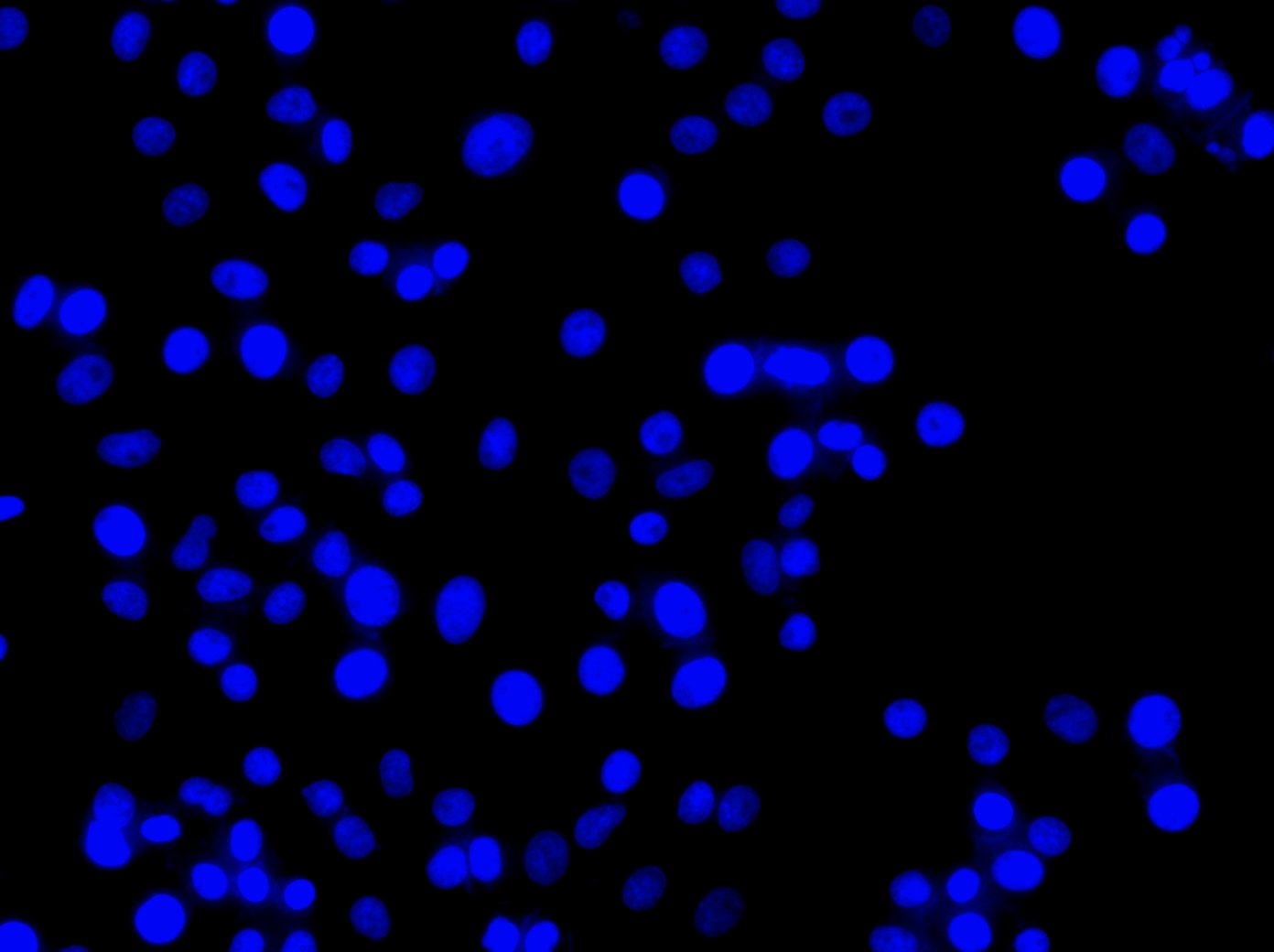

Supplement: Data S3 [file peerj-12-17664-s003.zip › raw data3/EDU/BV2+LPS_C8-D1A (7).jpg]

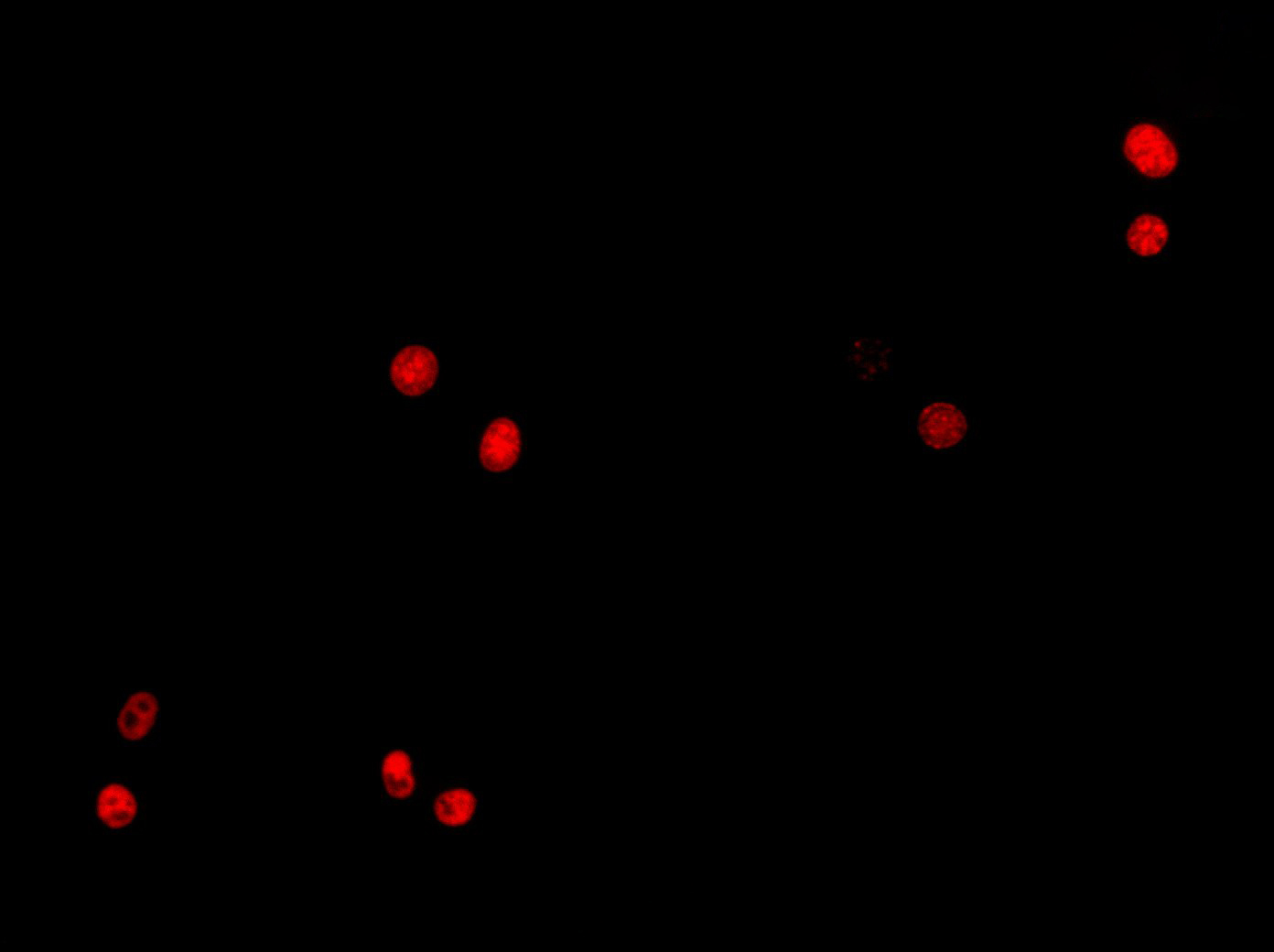

Supplement: Data S3 [file peerj-12-17664-s003.zip › raw data3/EDU/BV2+LPS_C8-D1A (8).jpg]

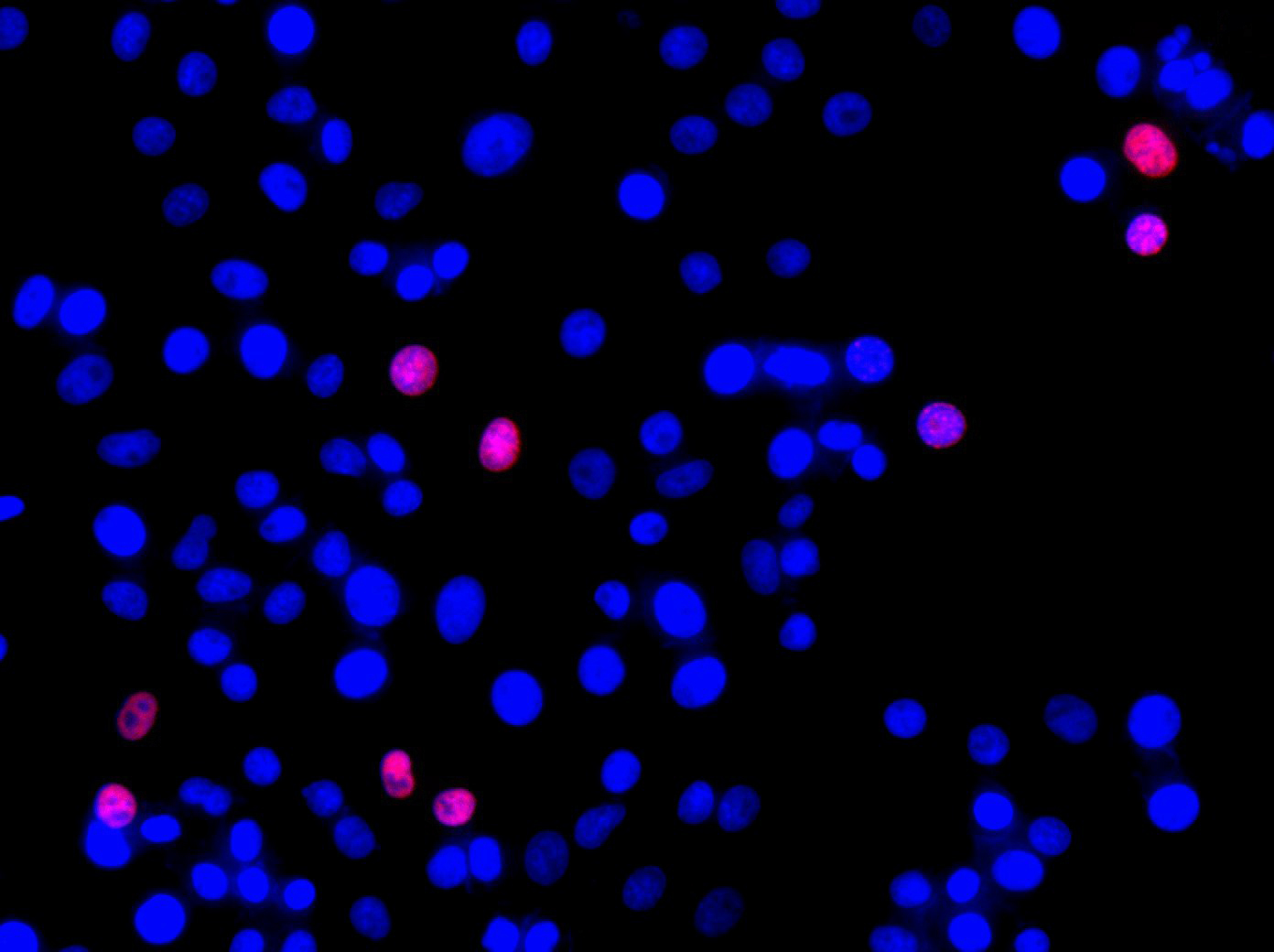

Supplement: Data S3 [file peerj-12-17664-s003.zip › raw data3/EDU/BV2+LPS_C8-D1A (9).jpg]

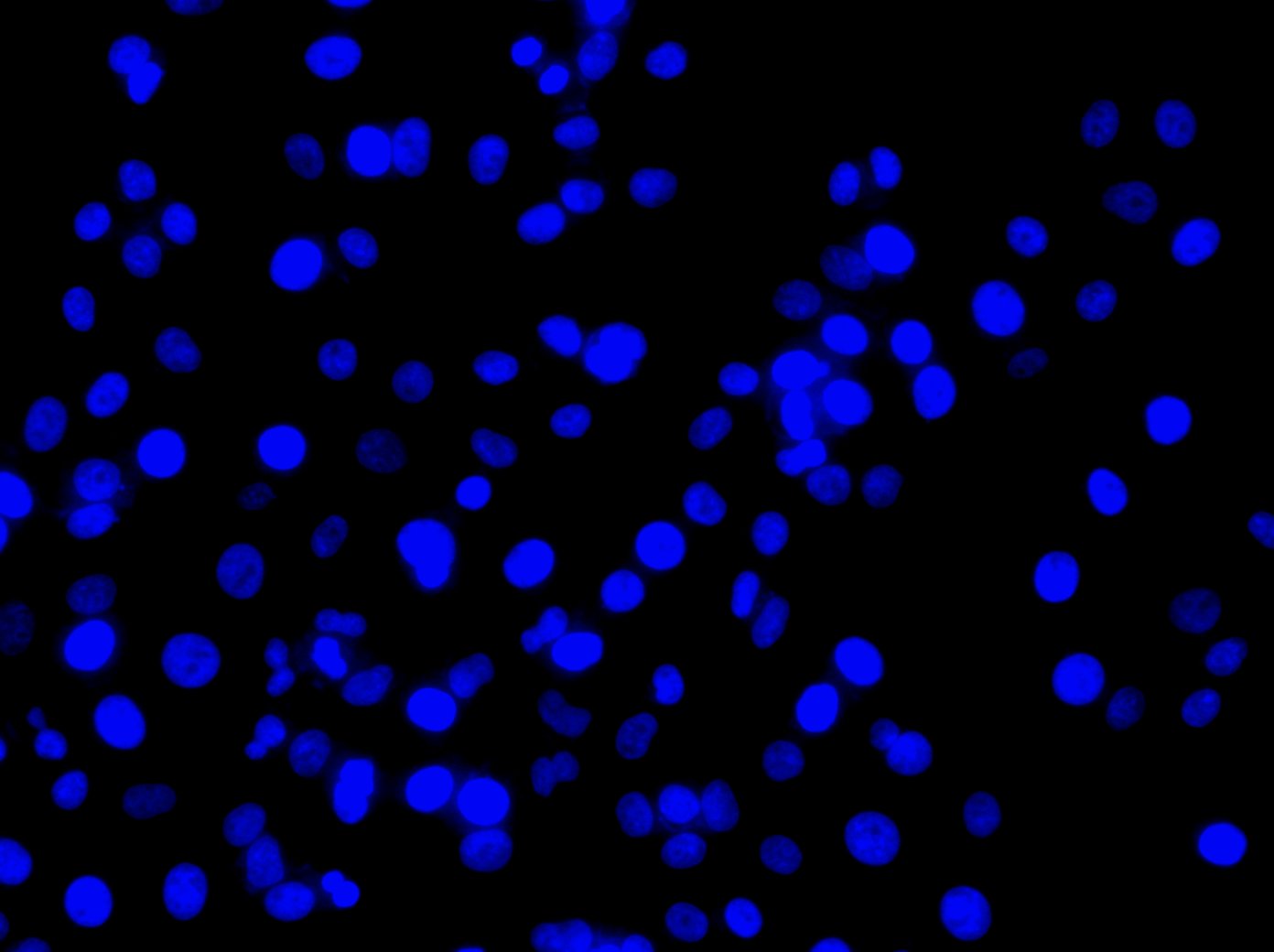

Supplement: Data S3 [file peerj-12-17664-s003.zip › raw data3/EDU/BV2+LPS+BMSC-CM_C8-D1A (1).jpg]

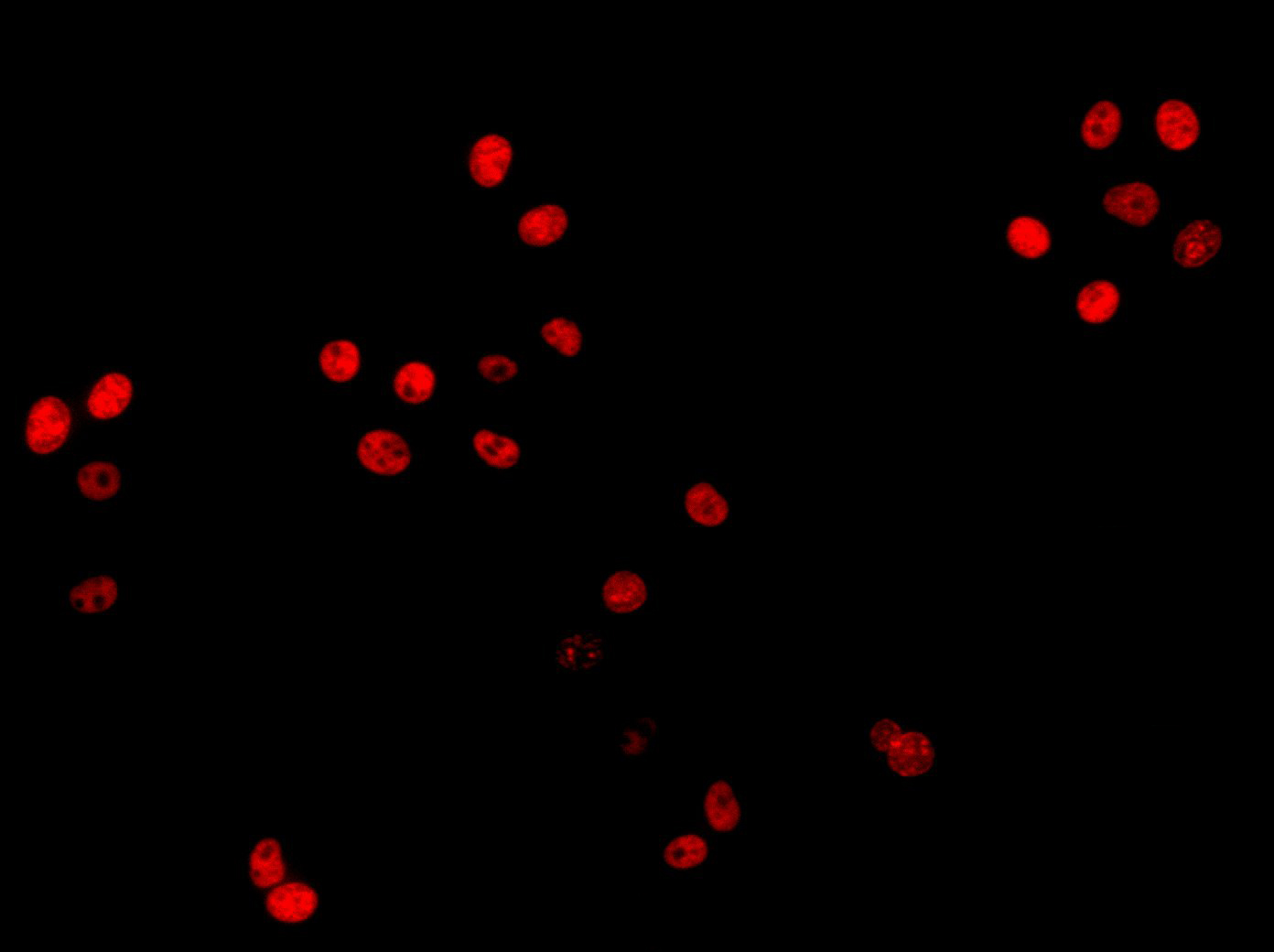

Supplement: Data S3 [file peerj-12-17664-s003.zip › raw data3/EDU/BV2+LPS+BMSC-CM_C8-D1A (2).jpg]

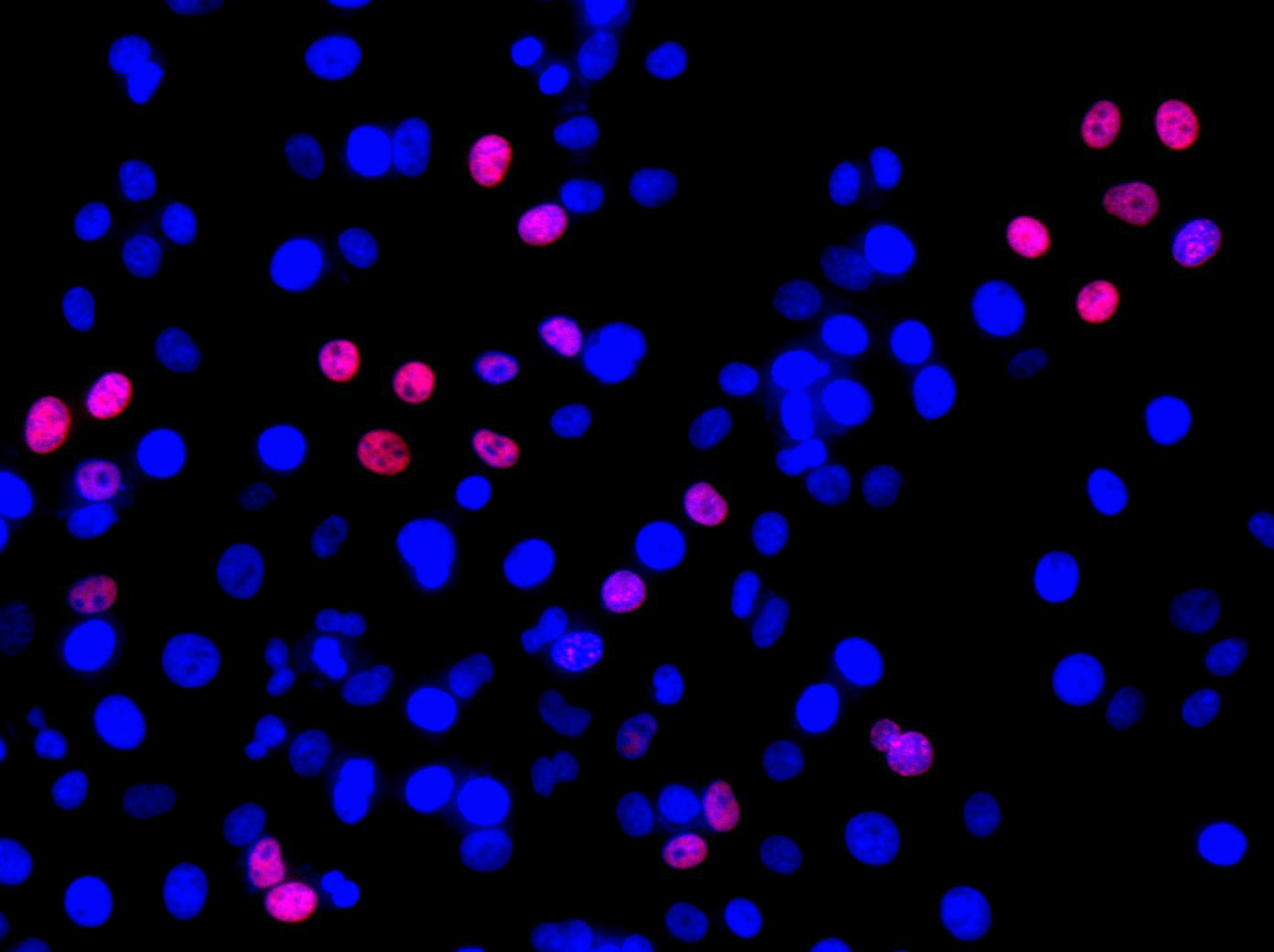

Supplement: Data S3 [file peerj-12-17664-s003.zip › raw data3/EDU/BV2+LPS+BMSC-CM_C8-D1A (3).jpg]

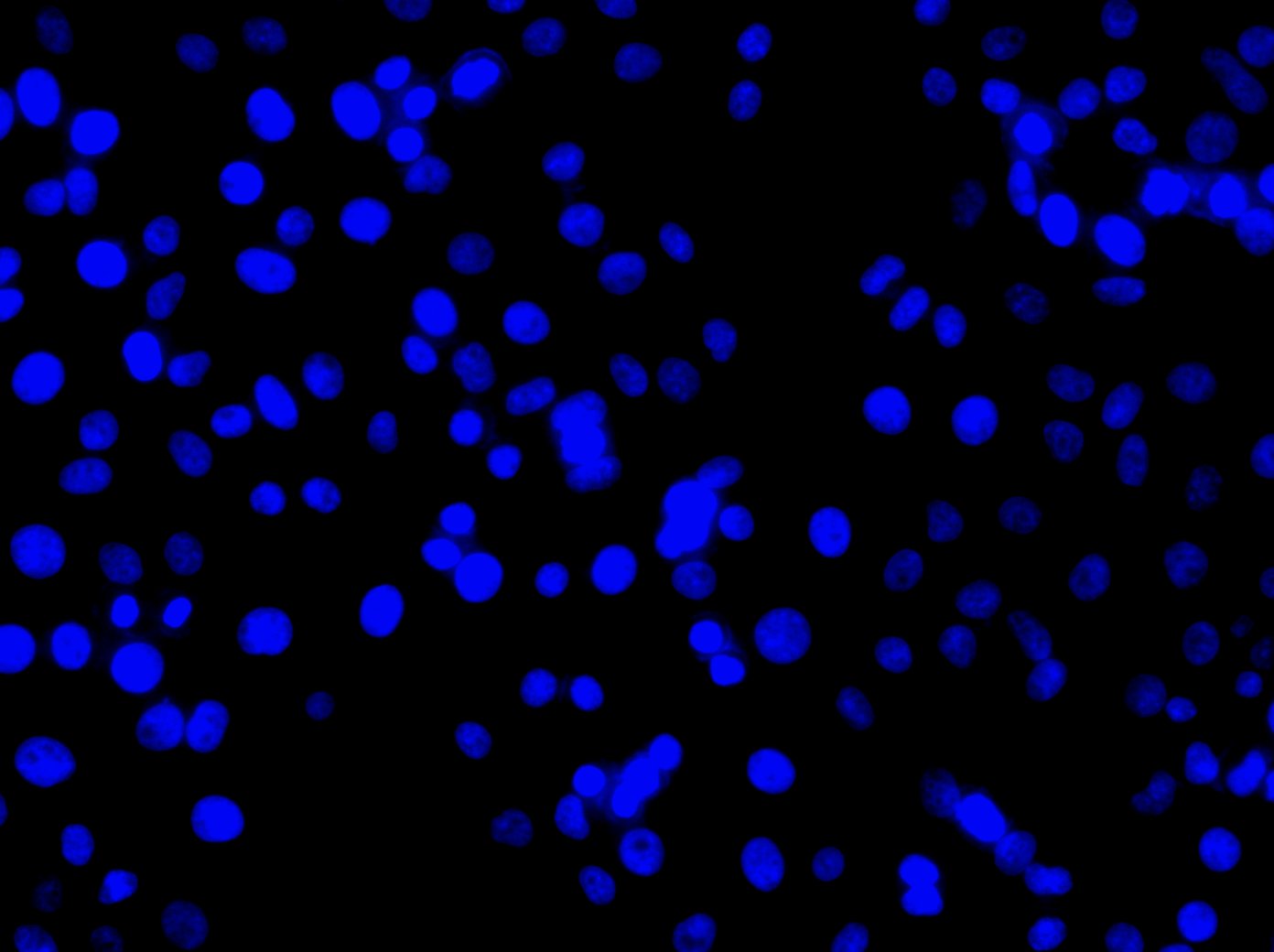

Supplement: Data S3 [file peerj-12-17664-s003.zip › raw data3/EDU/BV2+LPS+BMSC-CM_C8-D1A (4).jpg]

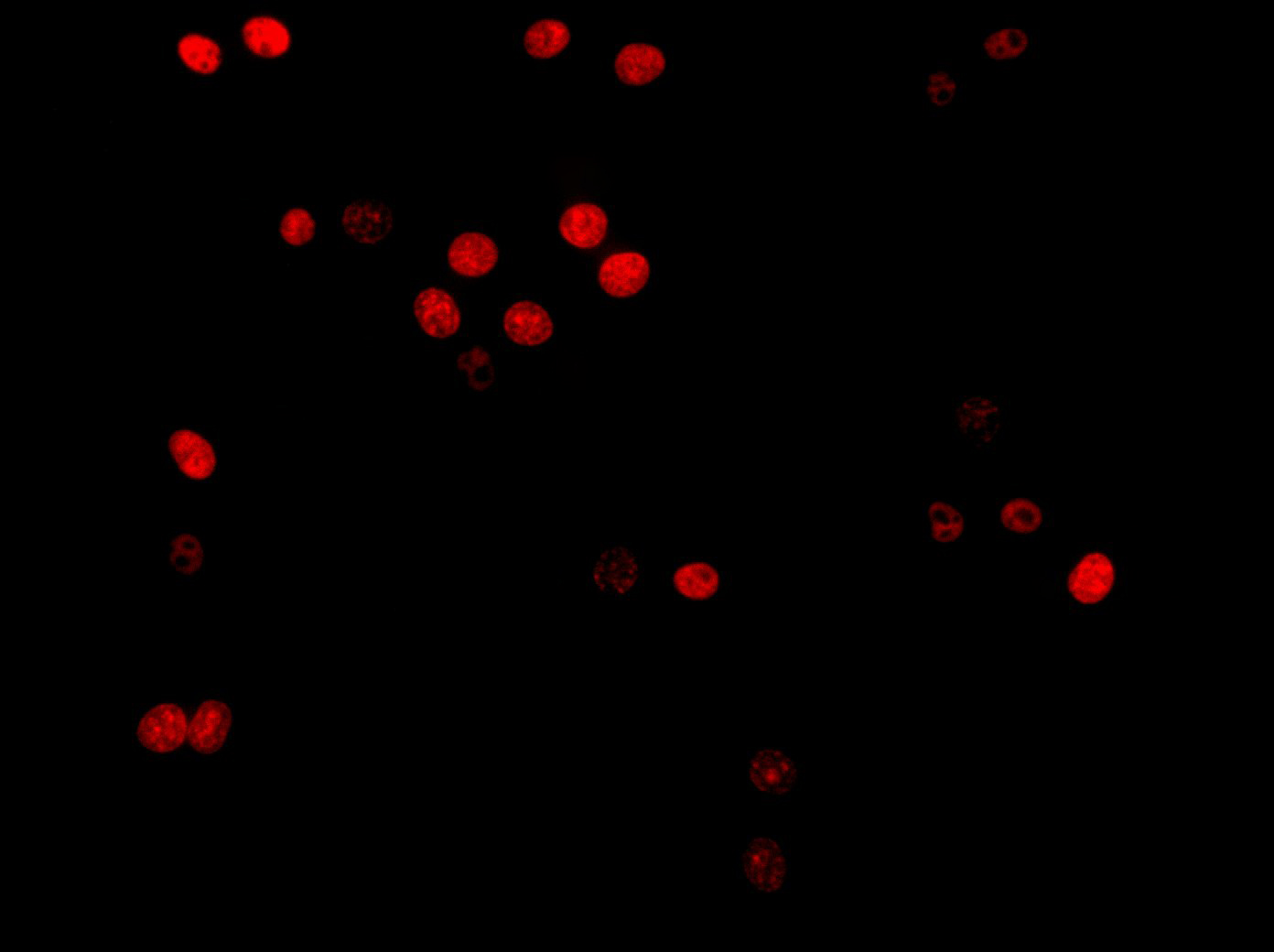

Supplement: Data S3 [file peerj-12-17664-s003.zip › raw data3/EDU/BV2+LPS+BMSC-CM_C8-D1A (5).jpg]

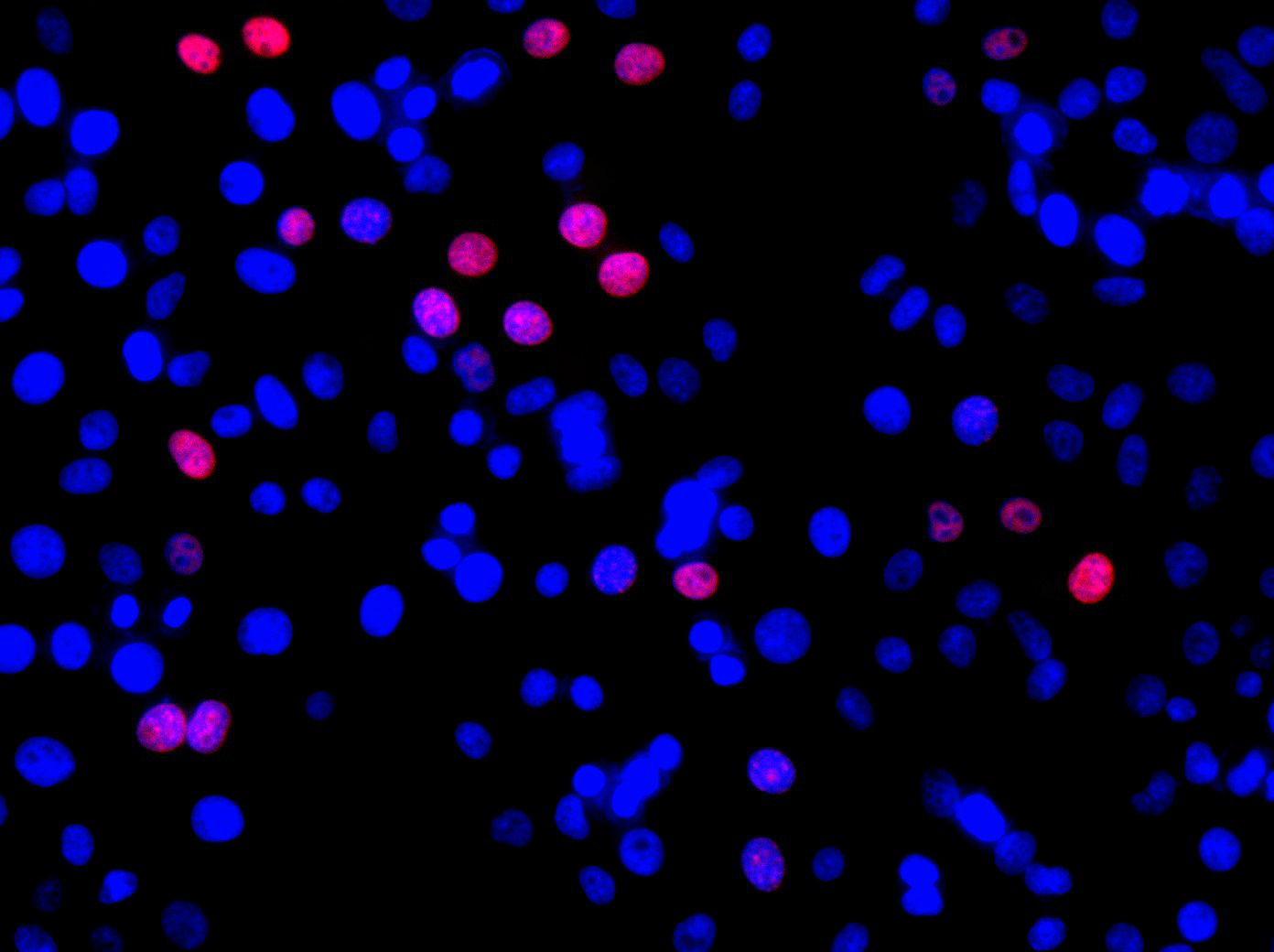

Supplement: Data S3 [file peerj-12-17664-s003.zip › raw data3/EDU/BV2+LPS+BMSC-CM_C8-D1A (6).jpg]

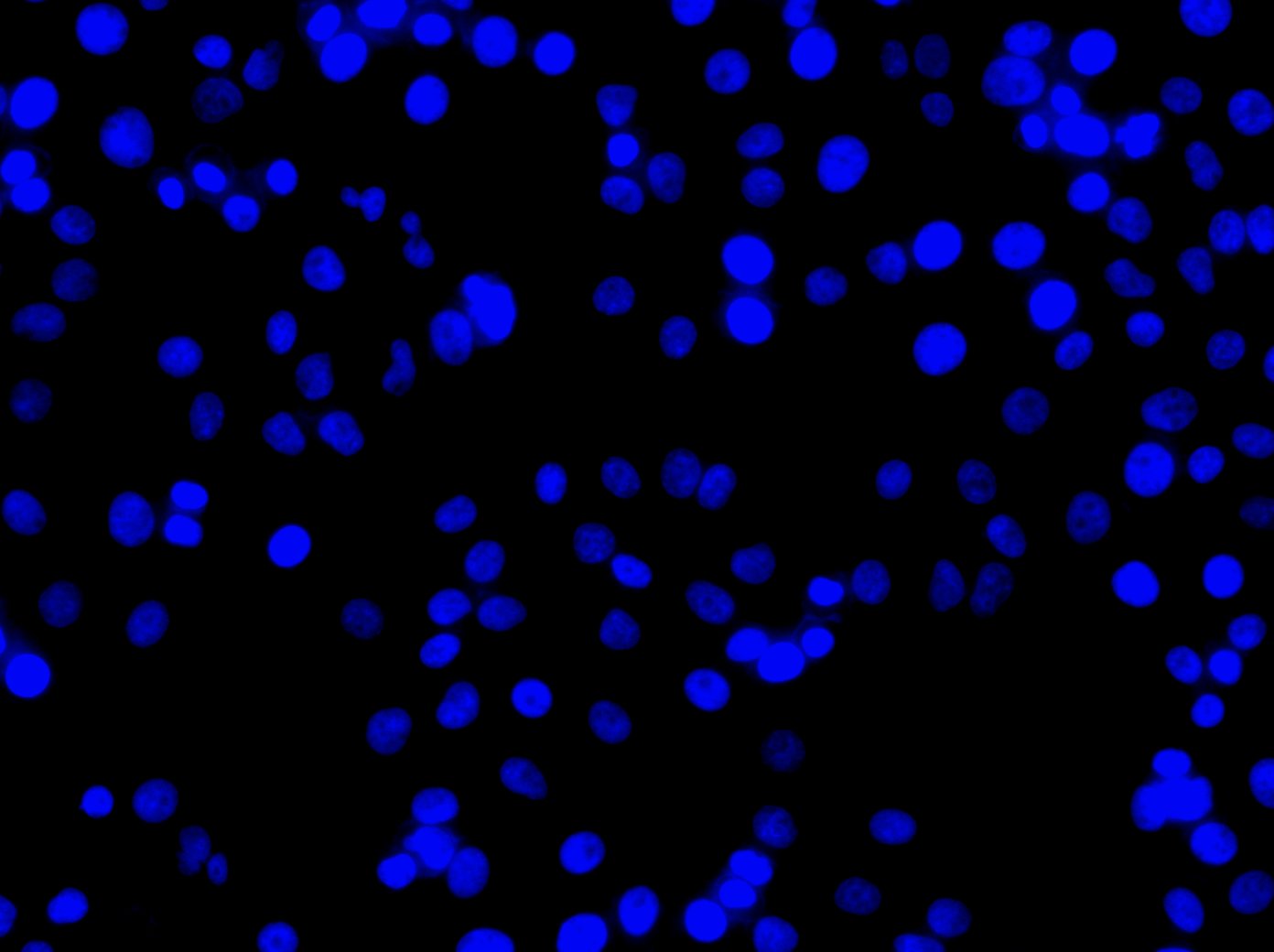

Supplement: Data S3 [file peerj-12-17664-s003.zip › raw data3/EDU/BV2+LPS+BMSC-CM_C8-D1A (7).jpg]

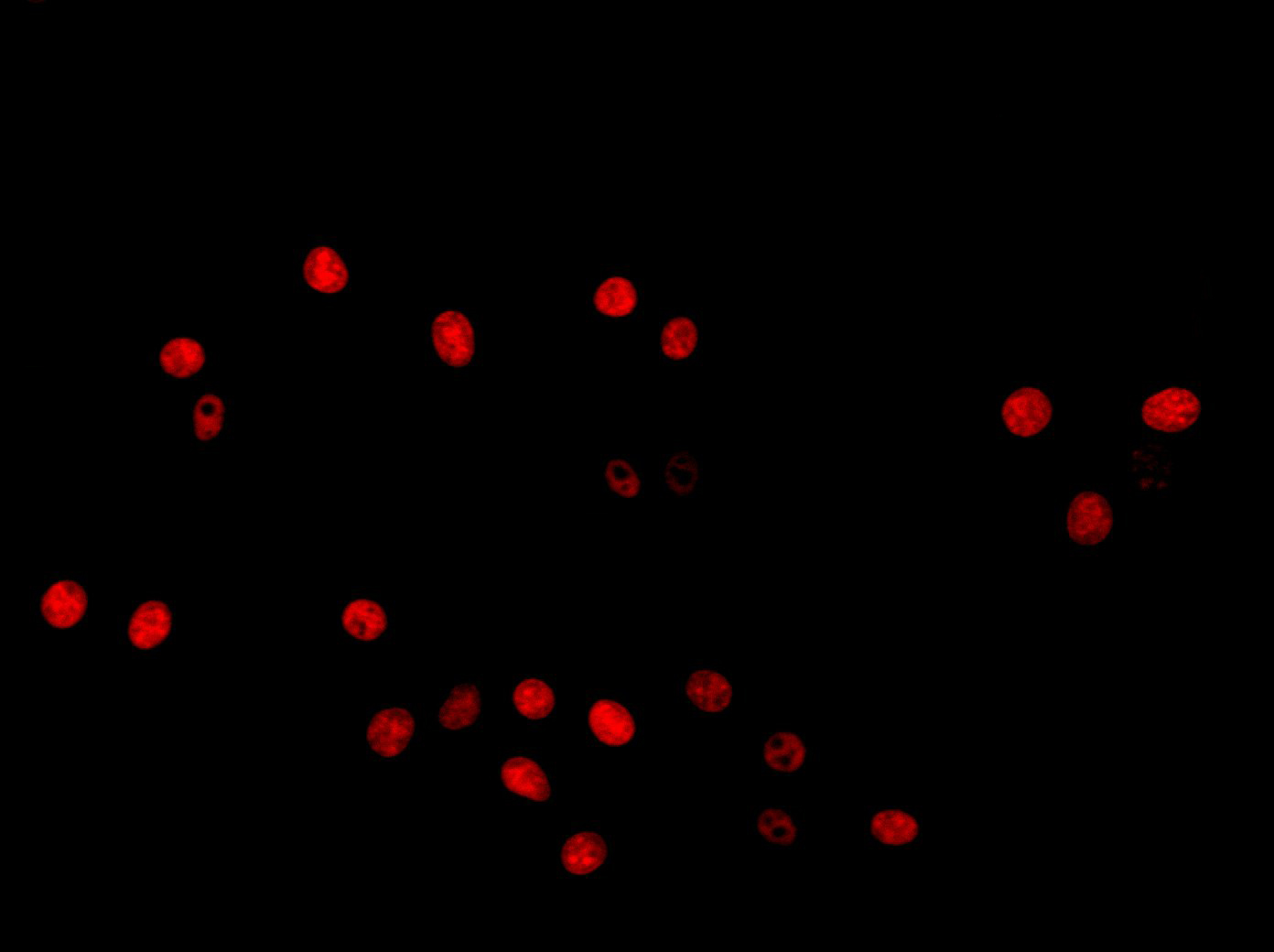

Supplement: Data S3 [file peerj-12-17664-s003.zip › raw data3/EDU/BV2+LPS+BMSC-CM_C8-D1A (8).jpg]

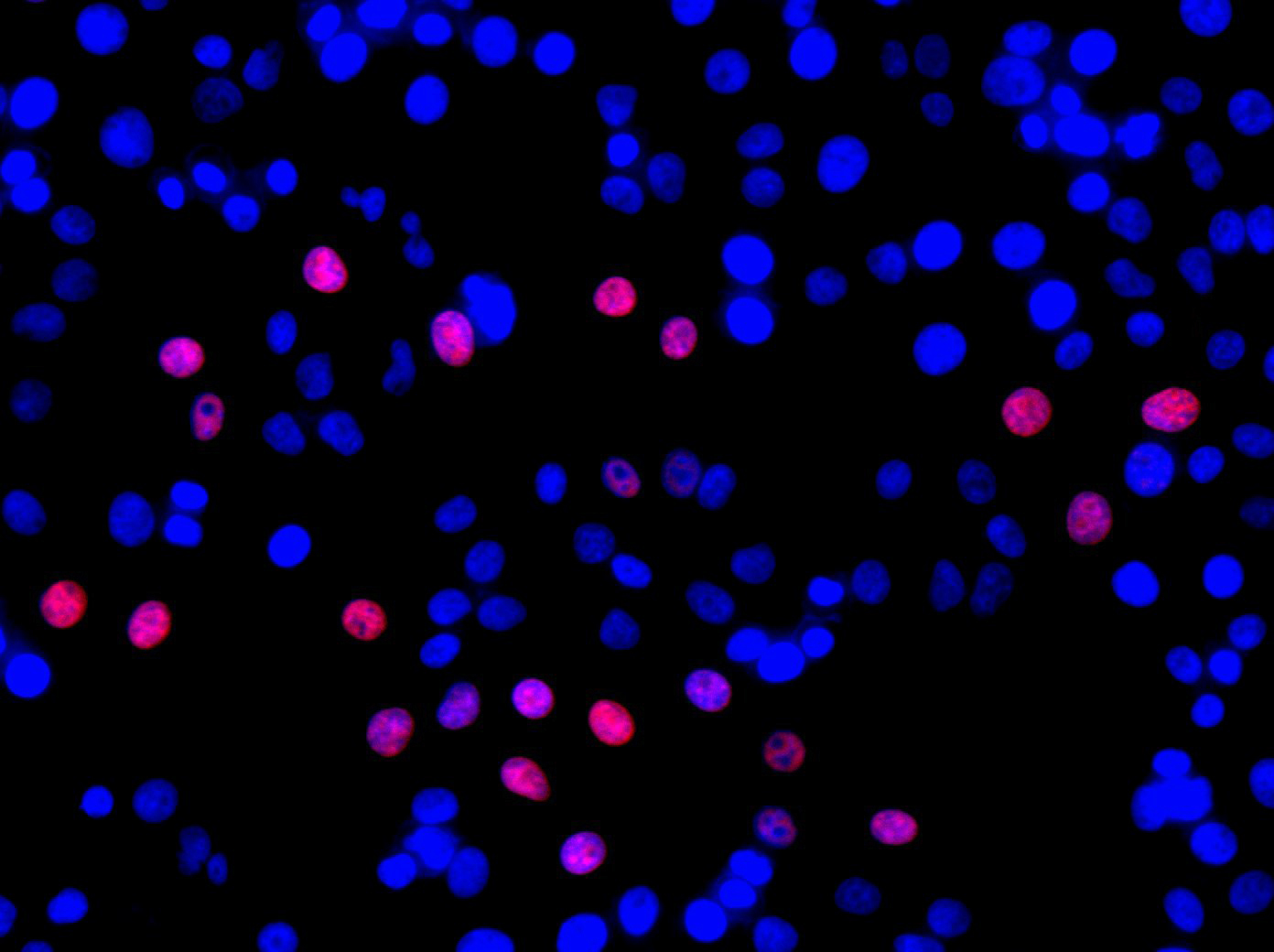

Supplement: Data S3 [file peerj-12-17664-s003.zip › raw data3/EDU/BV2+LPS+BMSC-CM_C8-D1A (9).jpg]

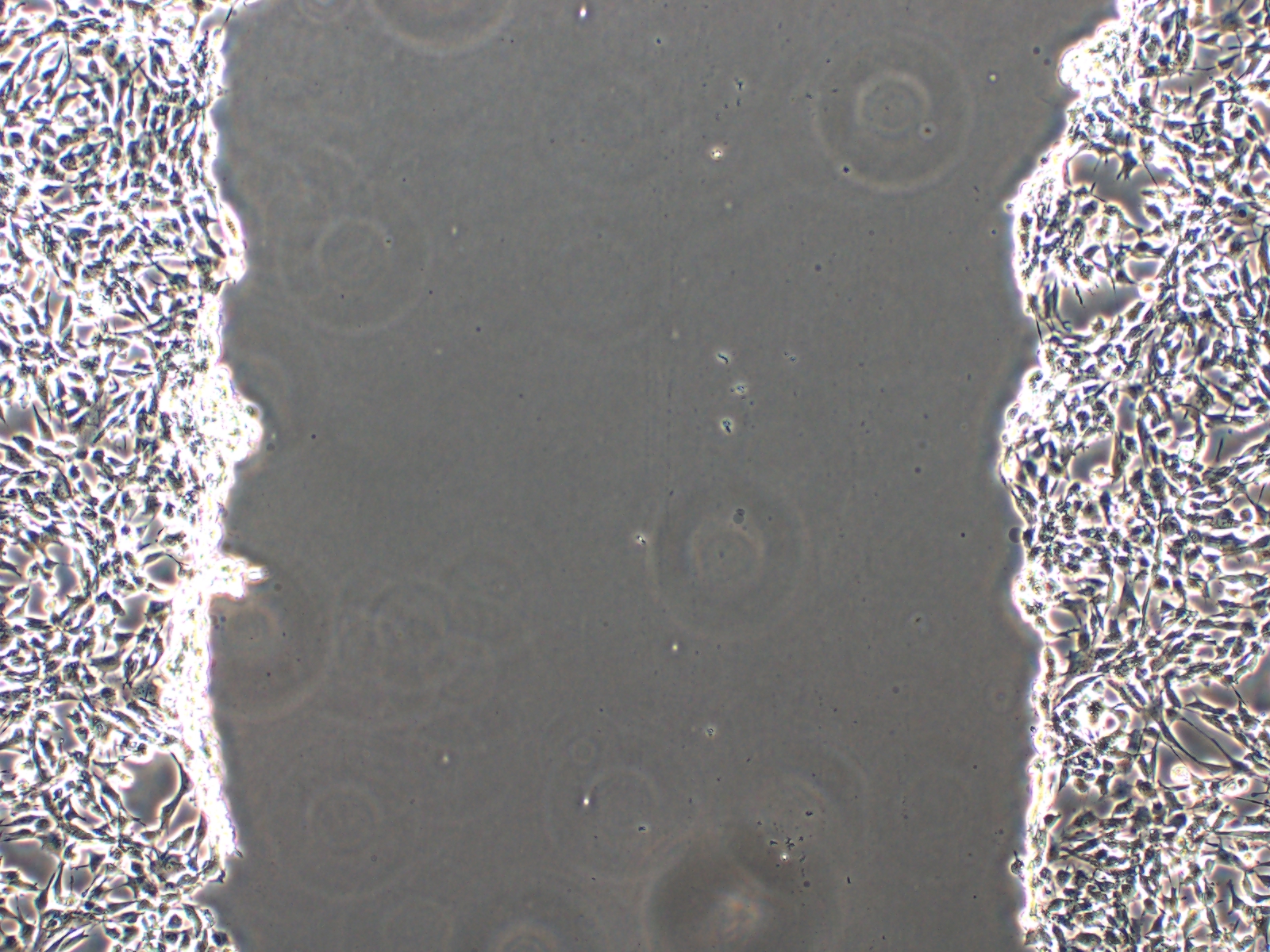

Supplement: Data S4 [file peerj-12-17664-s004.zip › raw data4/migration/0h/BV2_C8-D1A (1).jpg]

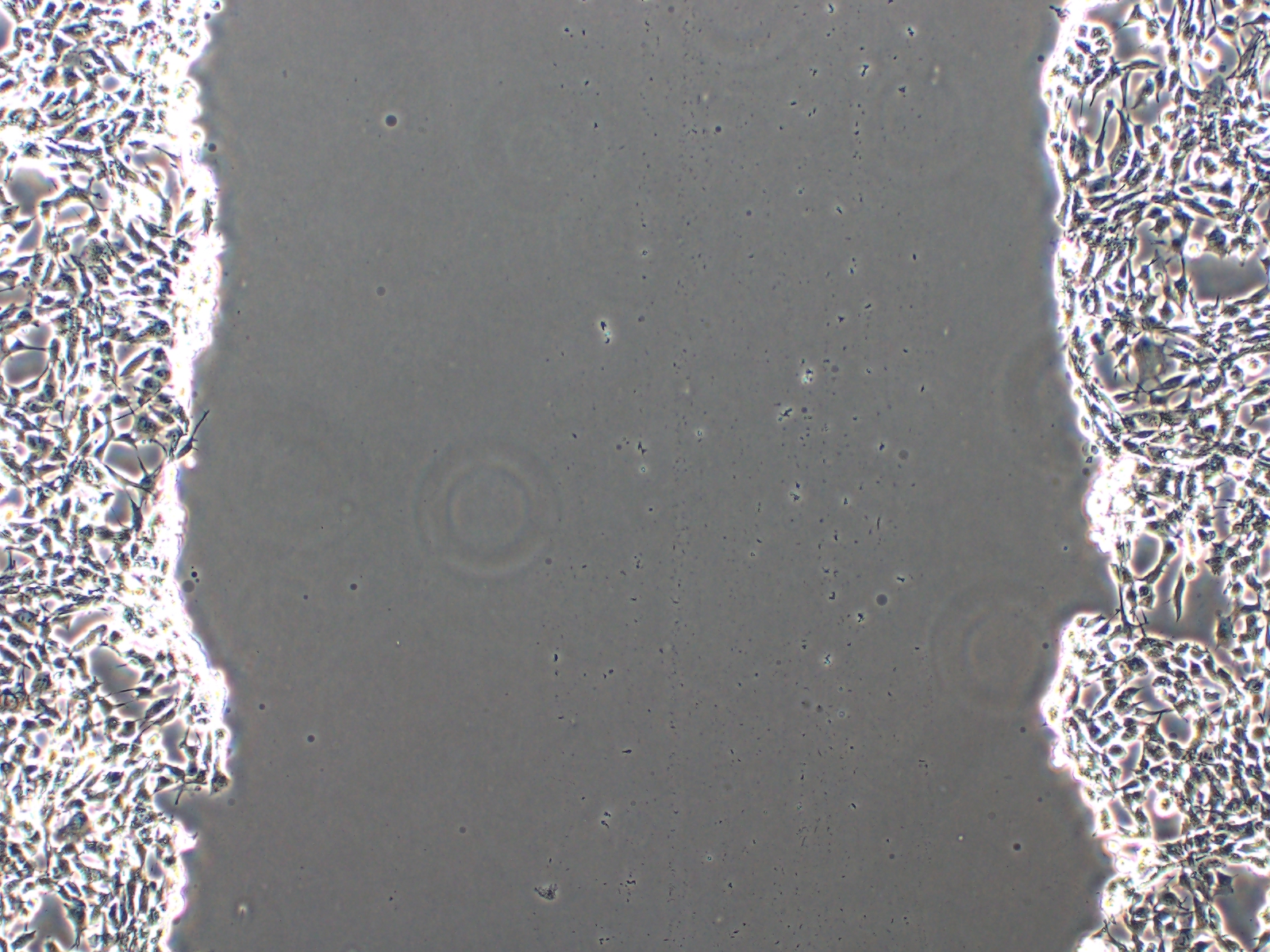

Supplement: Data S4 [file peerj-12-17664-s004.zip › raw data4/migration/0h/BV2_C8-D1A (2).jpg]

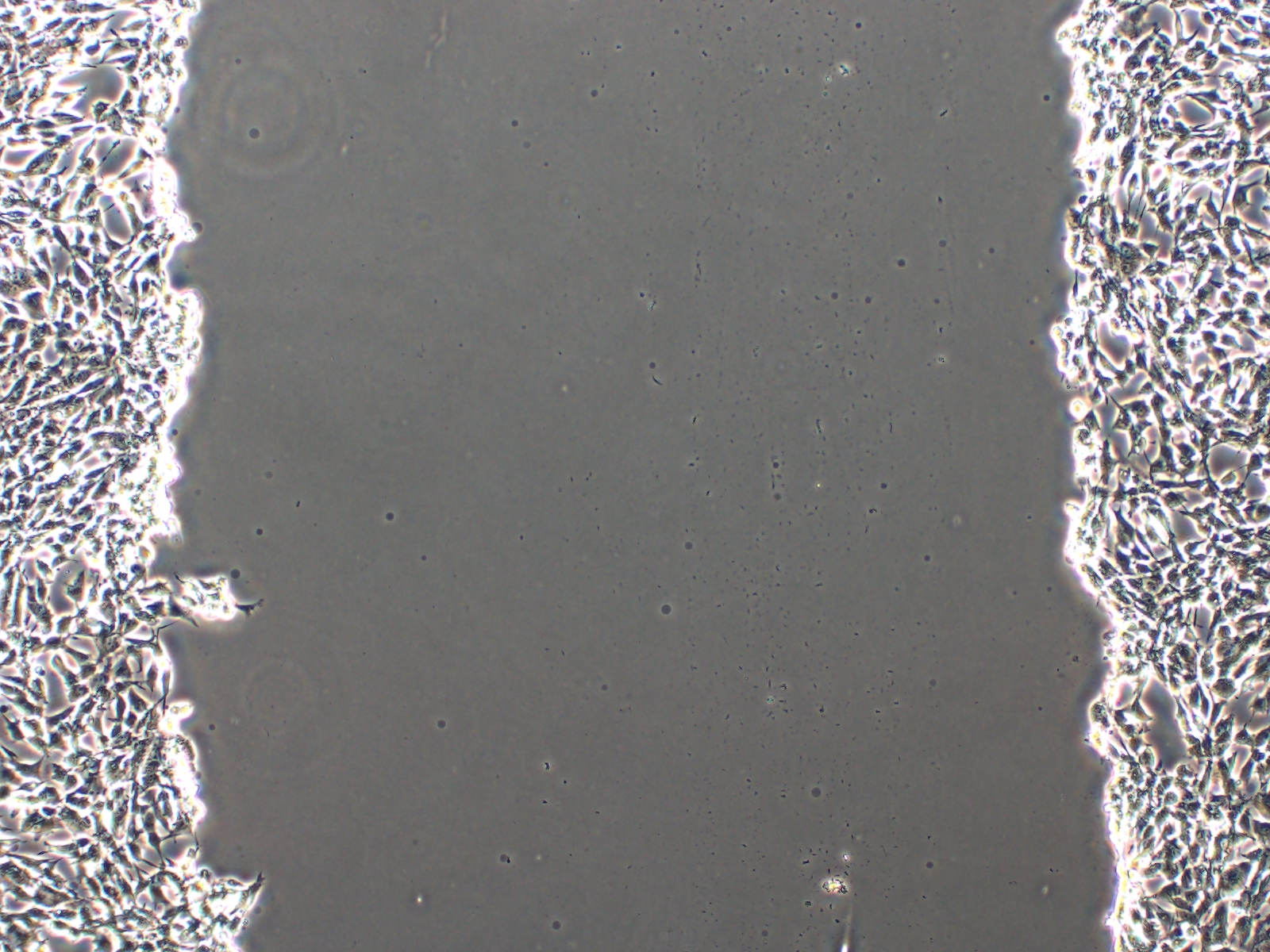

Supplement: Data S4 [file peerj-12-17664-s004.zip › raw data4/migration/0h/BV2_C8-D1A (3).jpg]

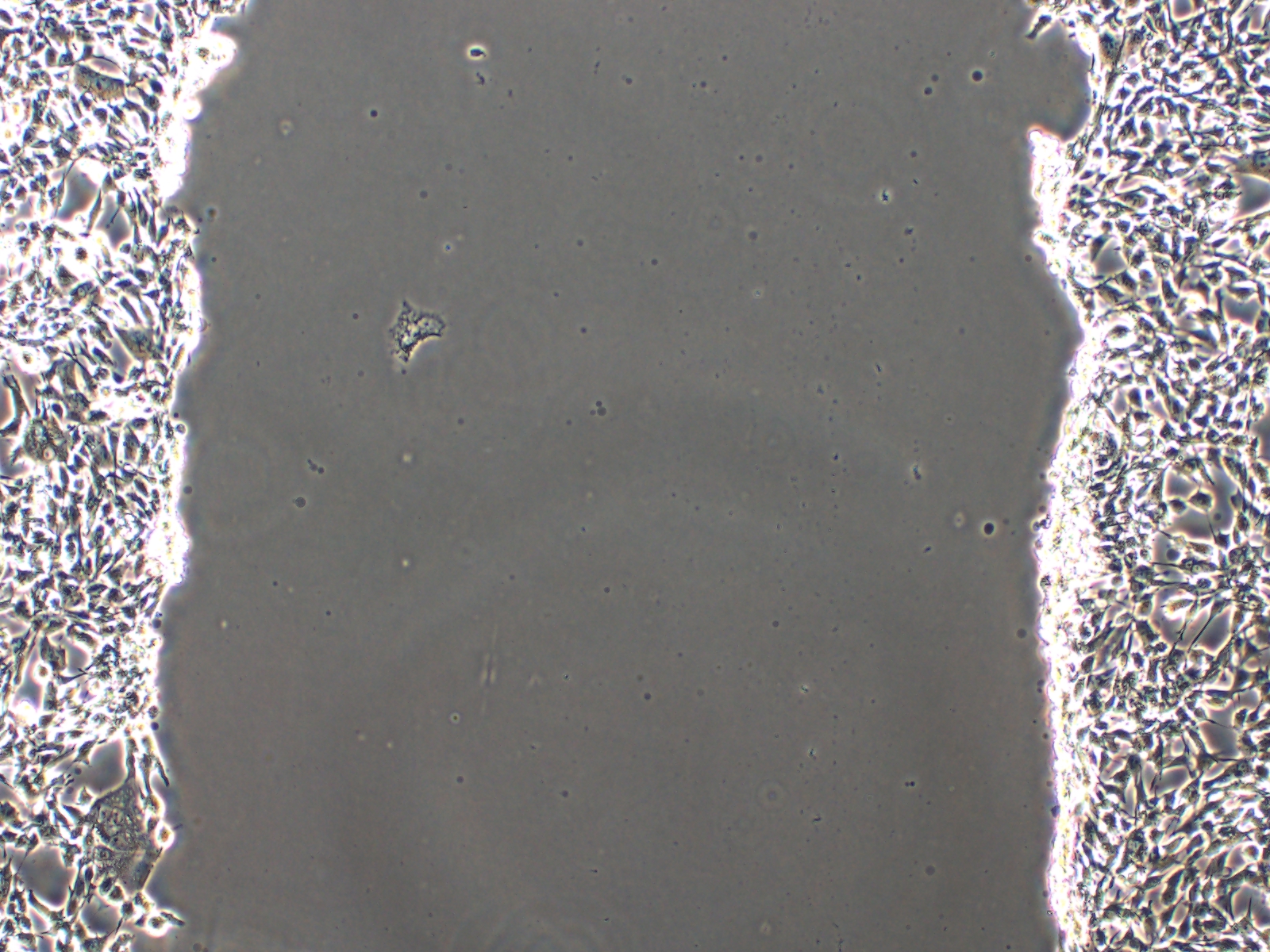

Supplement: Data S4 [file peerj-12-17664-s004.zip › raw data4/migration/0h/BV2+LPS_C8-D1A (1).jpg]

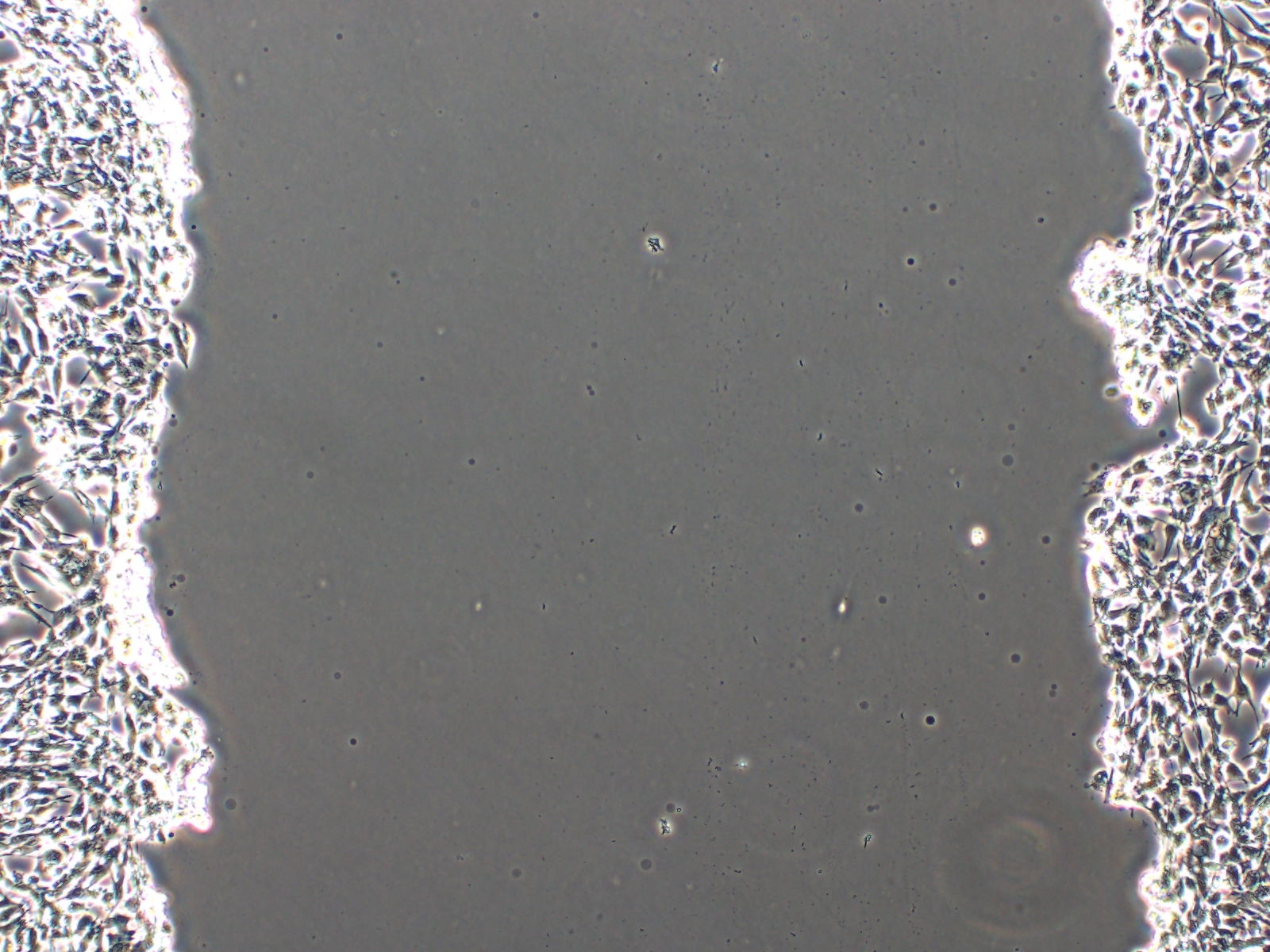

Supplement: Data S4 [file peerj-12-17664-s004.zip › raw data4/migration/0h/BV2+LPS_C8-D1A (2).jpg]

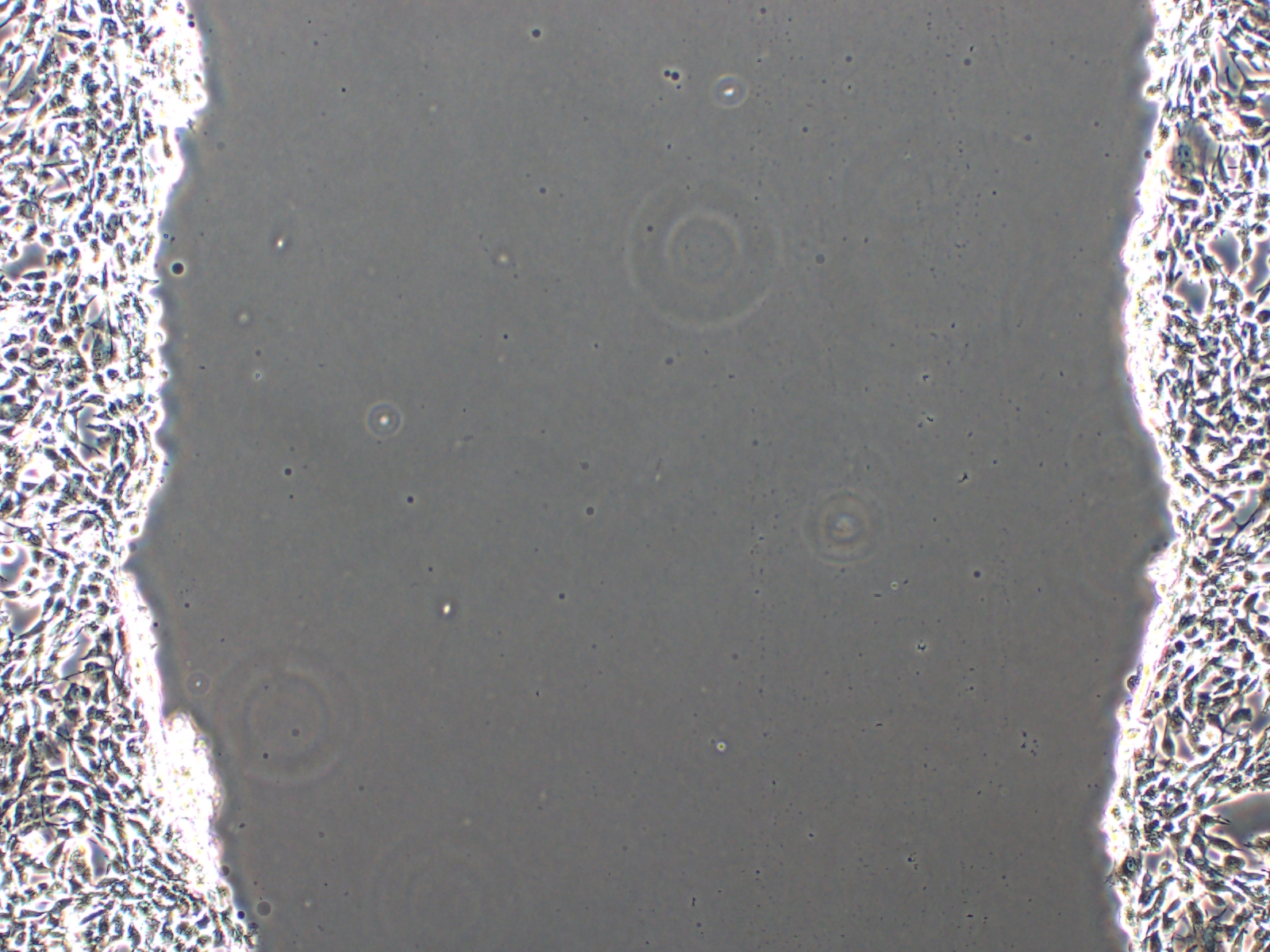

Supplement: Data S4 [file peerj-12-17664-s004.zip › raw data4/migration/0h/BV2+LPS_C8-D1A (3).jpg]

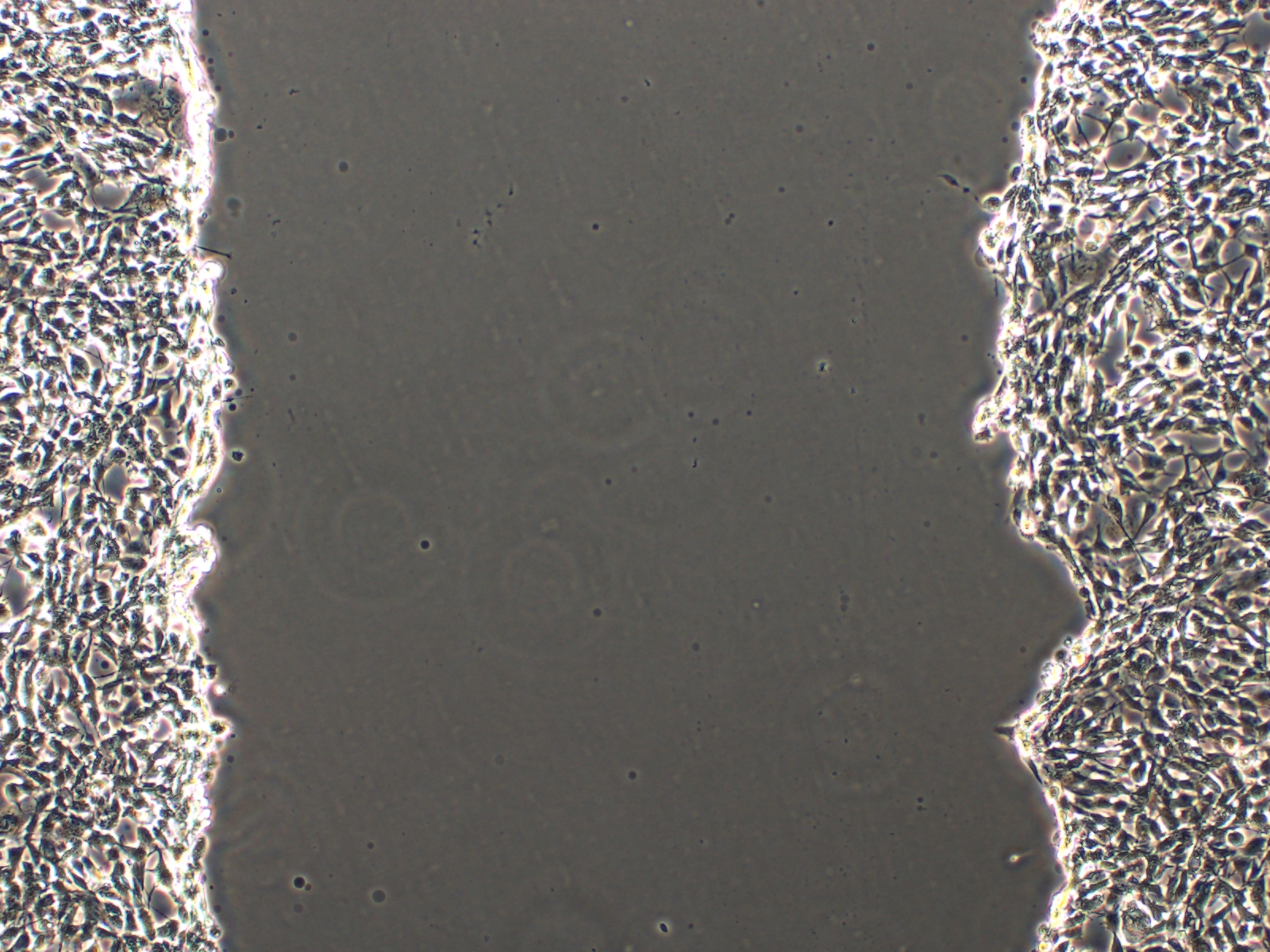

Supplement: Data S4 [file peerj-12-17664-s004.zip › raw data4/migration/0h/BV2+LPS+BMSC-CM_C8D1A (1).jpg]

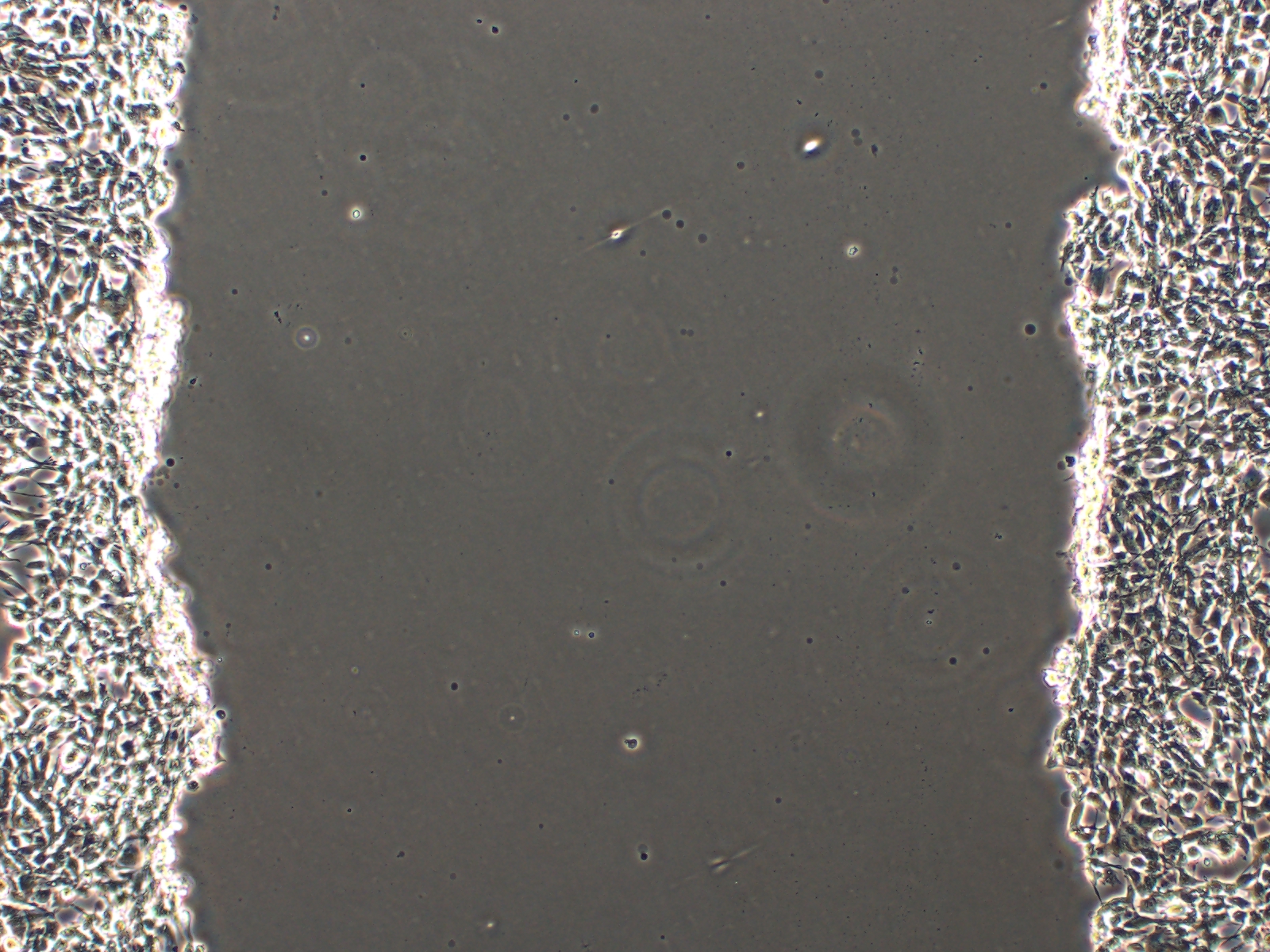

Supplement: Data S4 [file peerj-12-17664-s004.zip › raw data4/migration/0h/BV2+LPS+BMSC-CM_C8D1A (2).jpg]

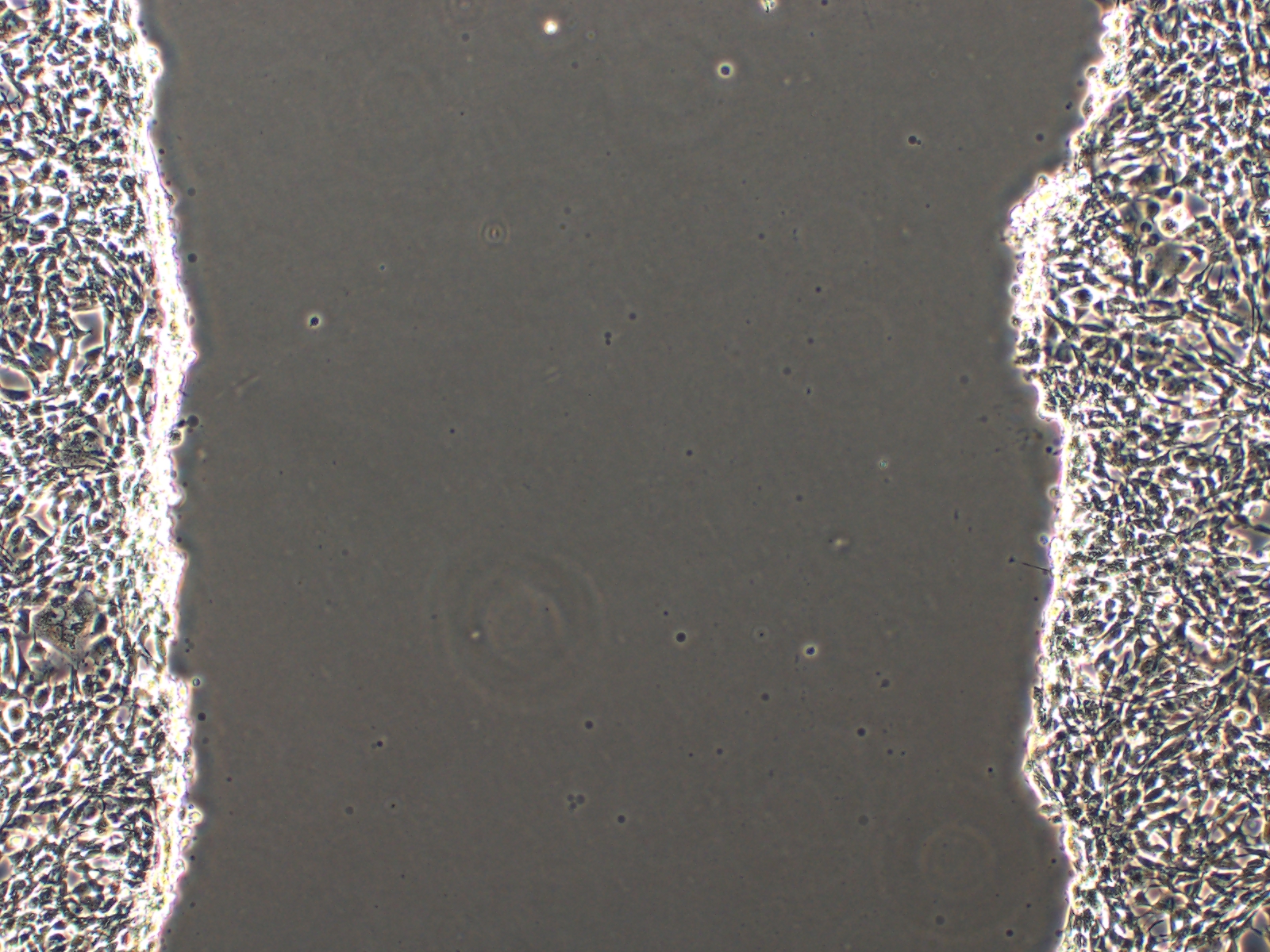

Supplement: Data S4 [file peerj-12-17664-s004.zip › raw data4/migration/0h/BV2+LPS+BMSC-CM_C8D1A (3).jpg]

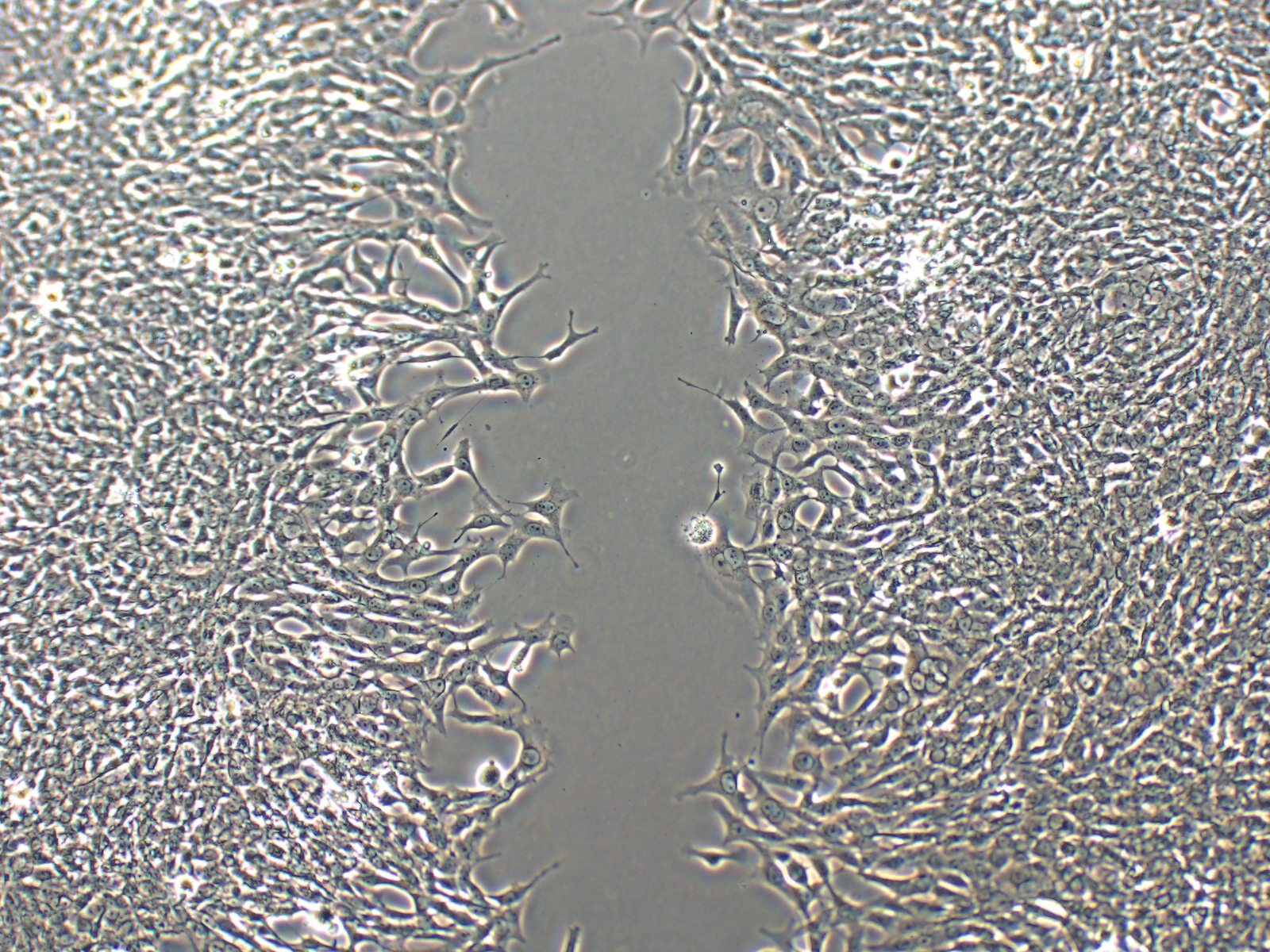

Supplement: Data S5 [file peerj-12-17664-s005.zip › raw data5/48h/BV2_C8-D1A (1).jpg]

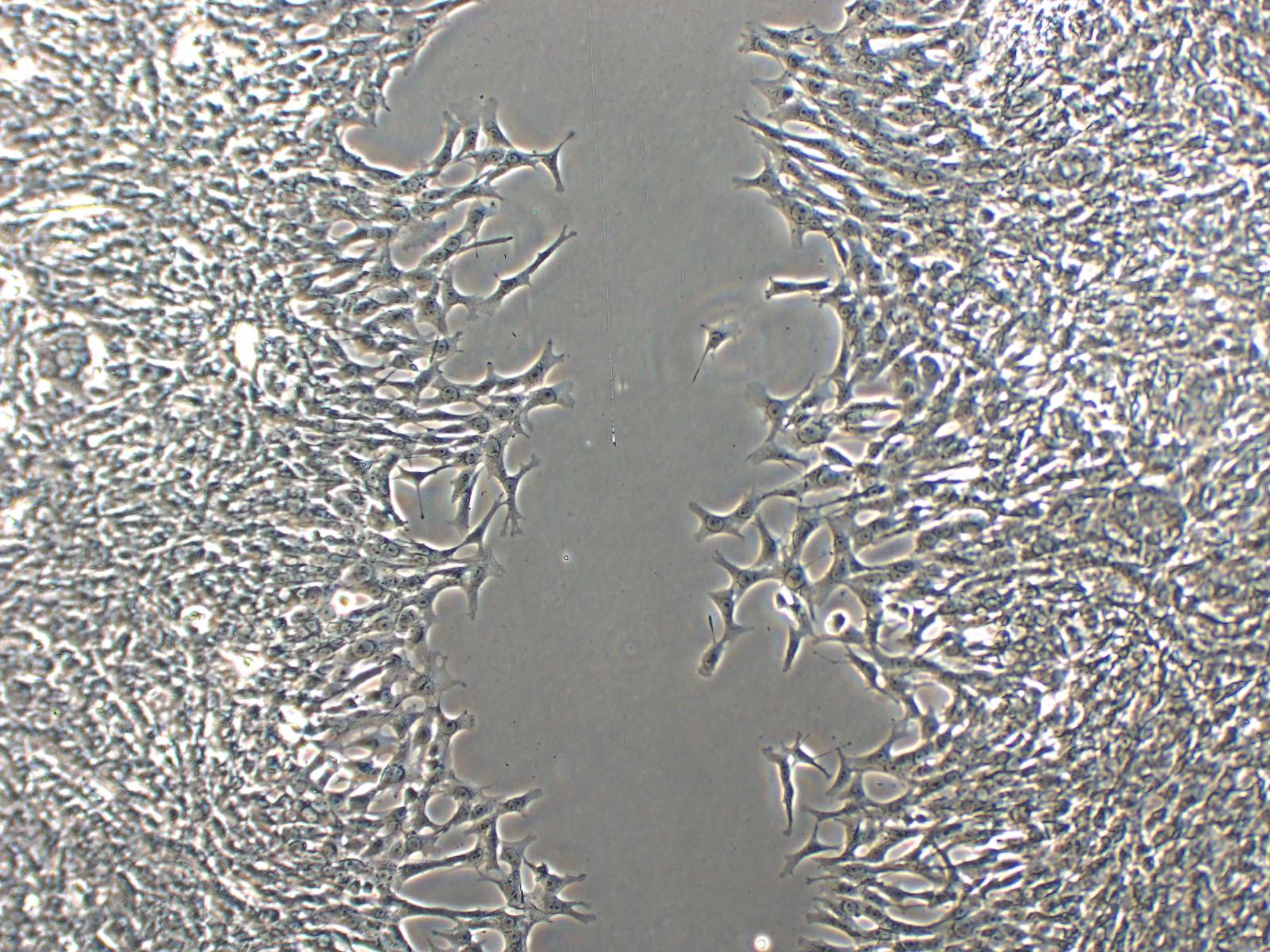

Supplement: Data S5 [file peerj-12-17664-s005.zip › raw data5/48h/BV2_C8-D1A (2).jpg]

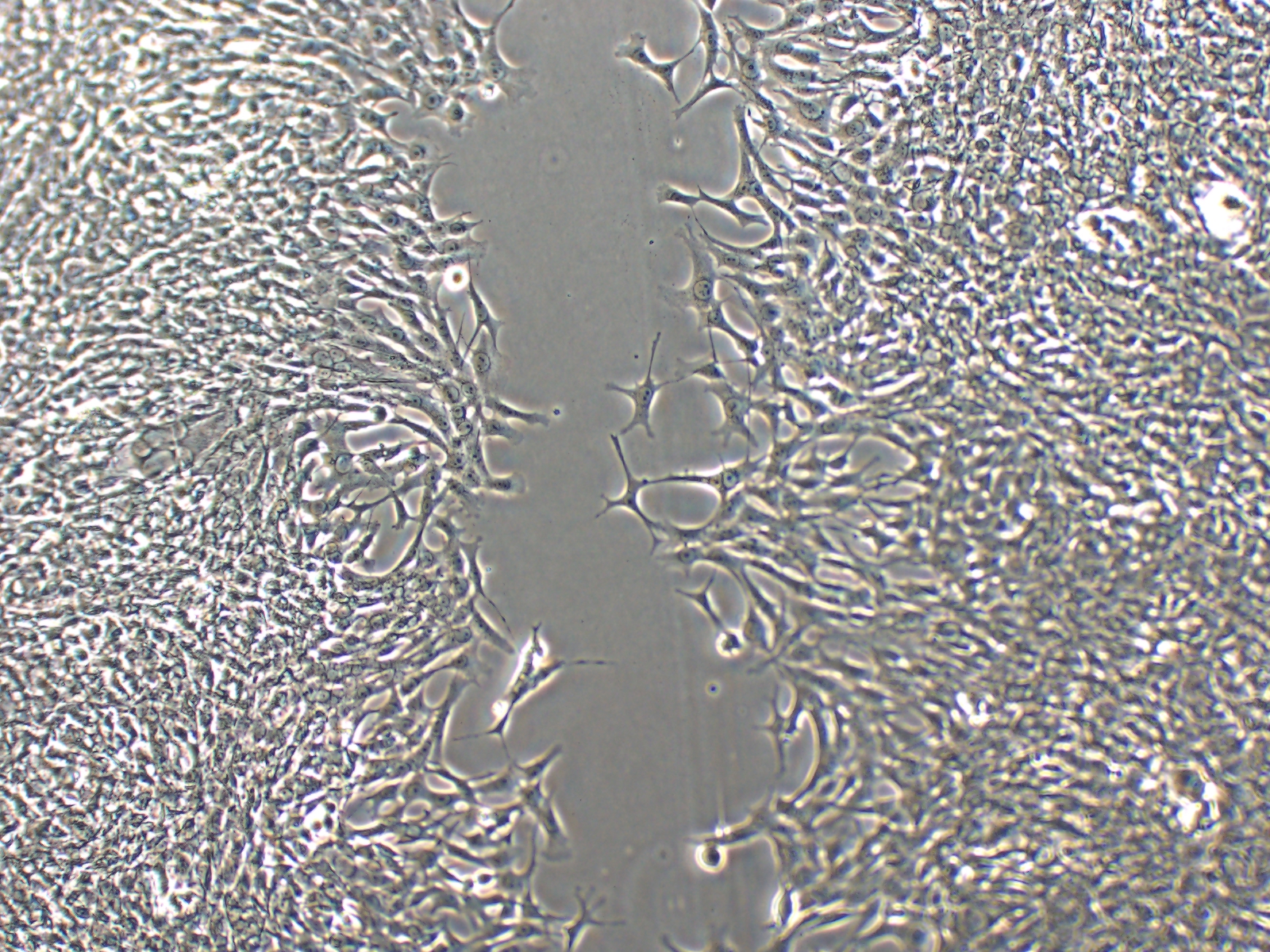

Supplement: Data S5 [file peerj-12-17664-s005.zip › raw data5/48h/BV2_C8-D1A (3).jpg]

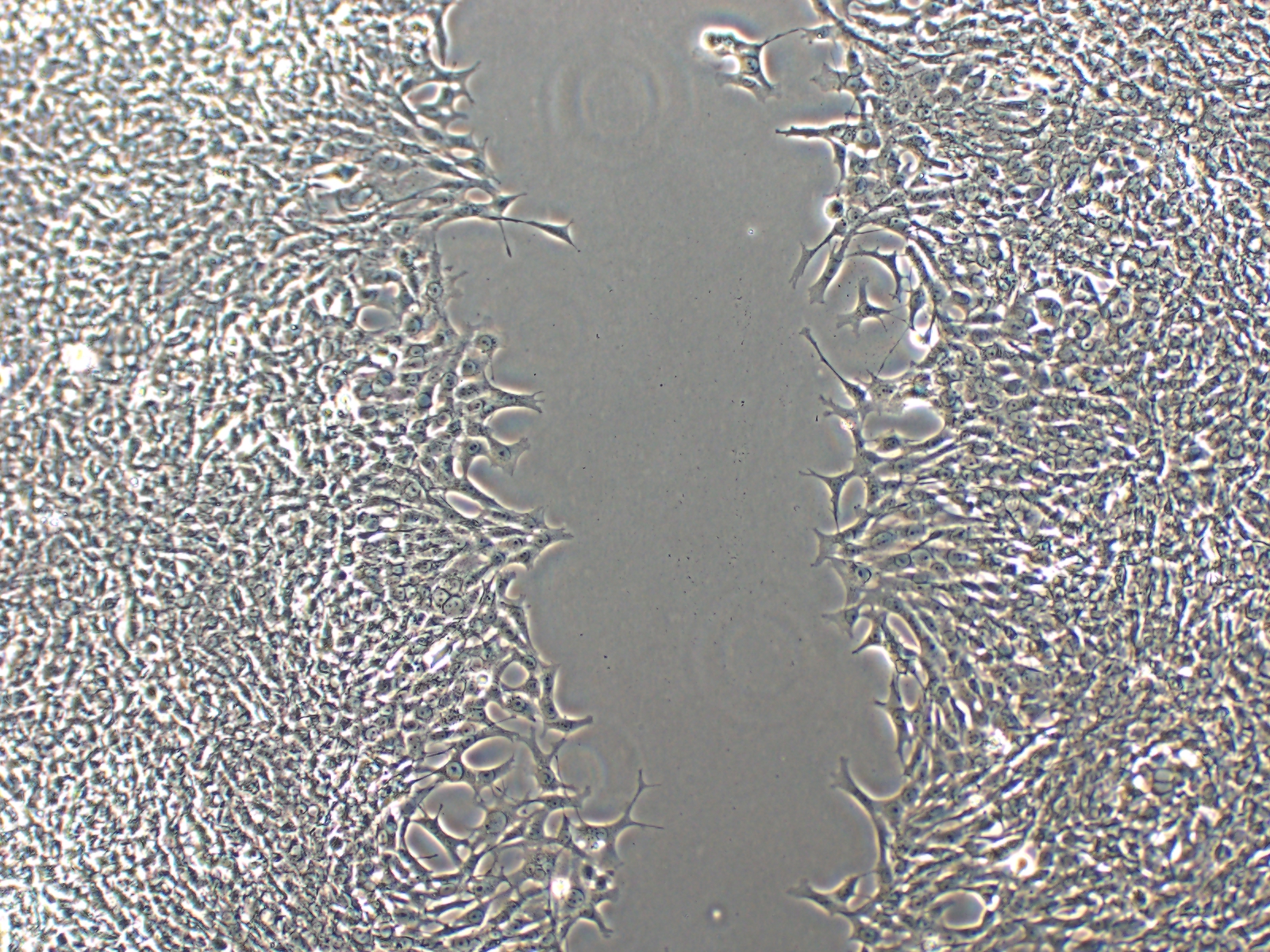

Supplement: Data S5 [file peerj-12-17664-s005.zip › raw data5/48h/BV2+LPS_C8-D1A (1).jpg]

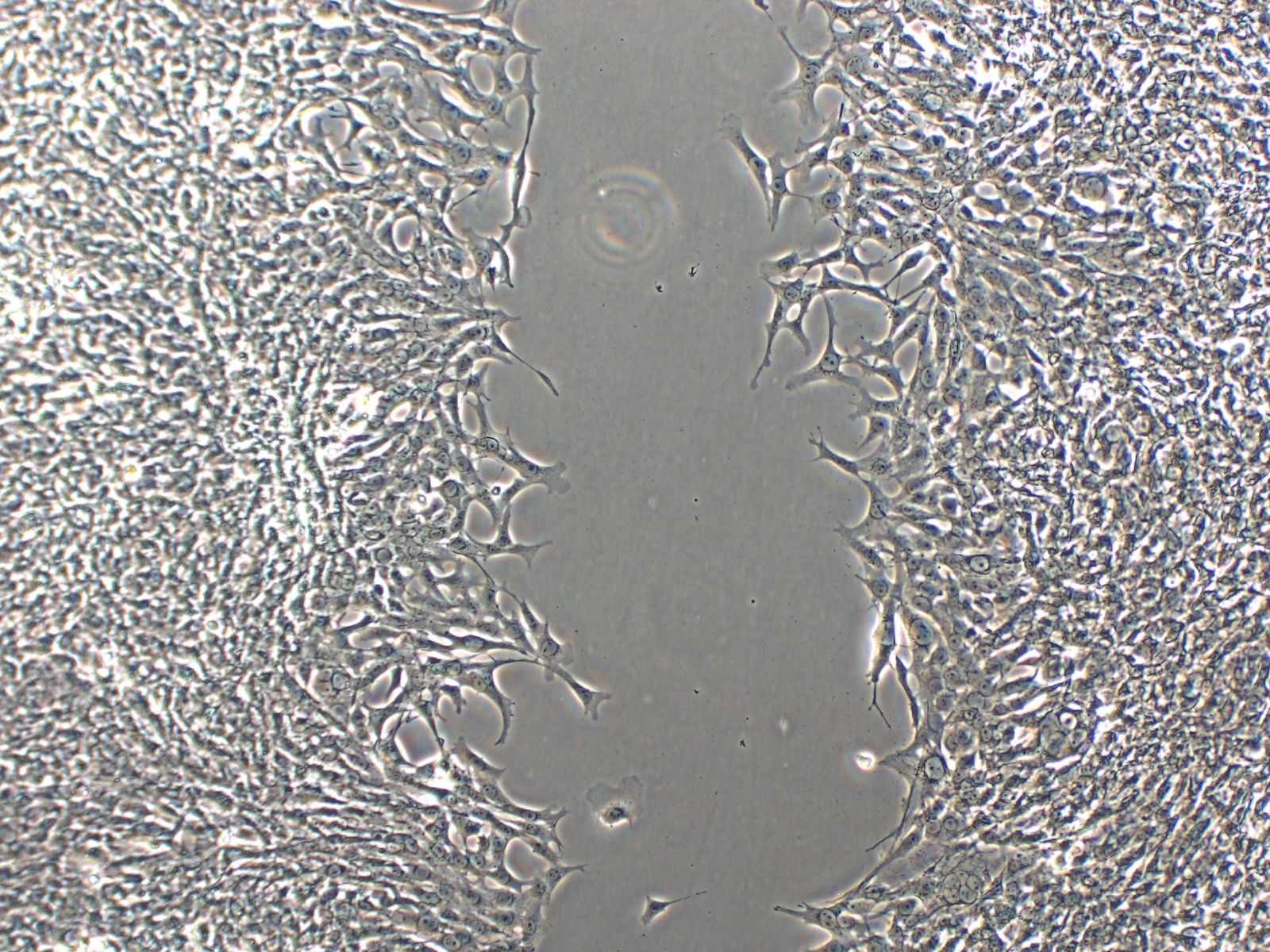

Supplement: Data S5 [file peerj-12-17664-s005.zip › raw data5/48h/BV2+LPS_C8-D1A (2).jpg]

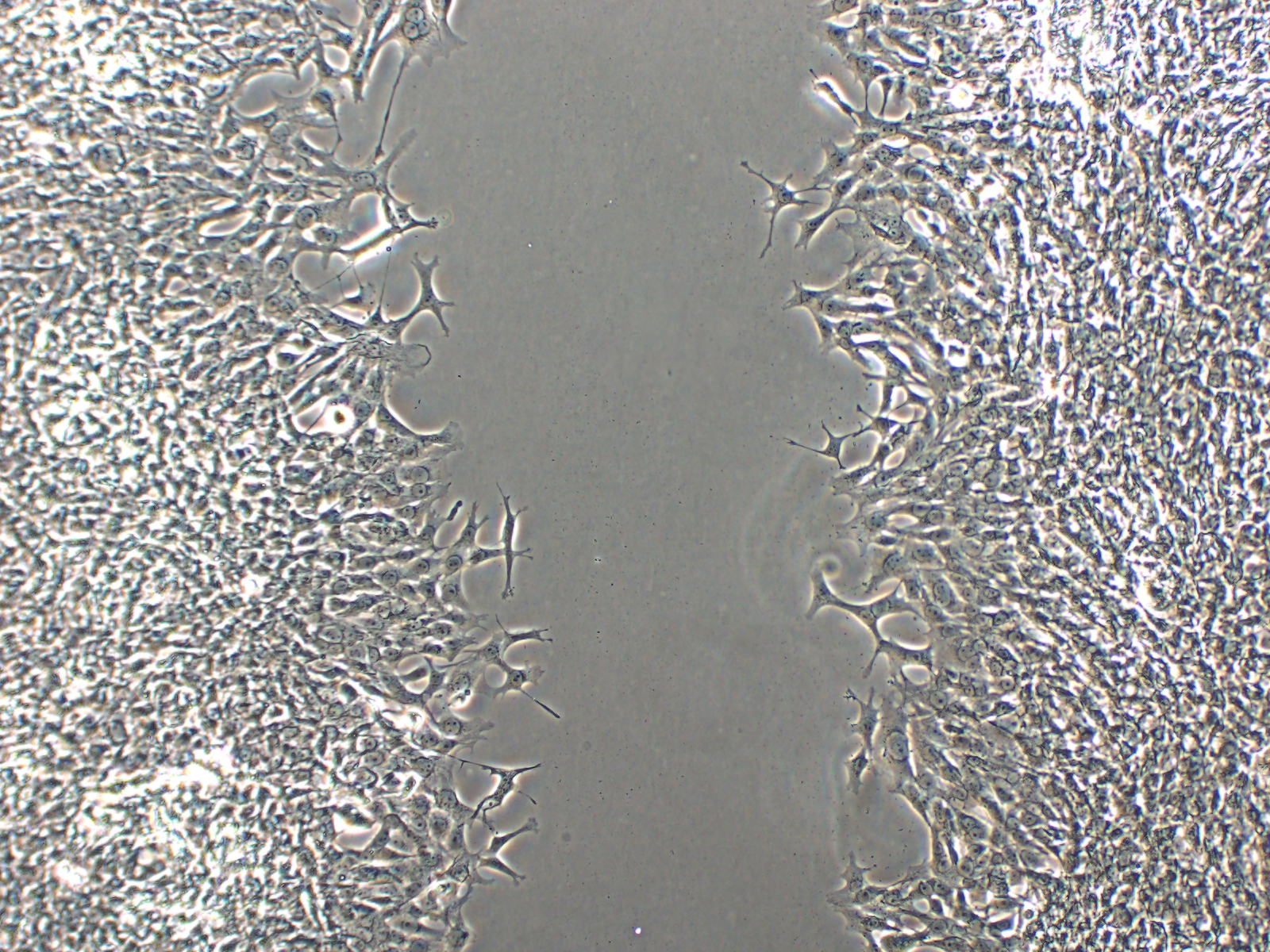

Supplement: Data S5 [file peerj-12-17664-s005.zip › raw data5/48h/BV2+LPS_C8-D1A (3).jpg]

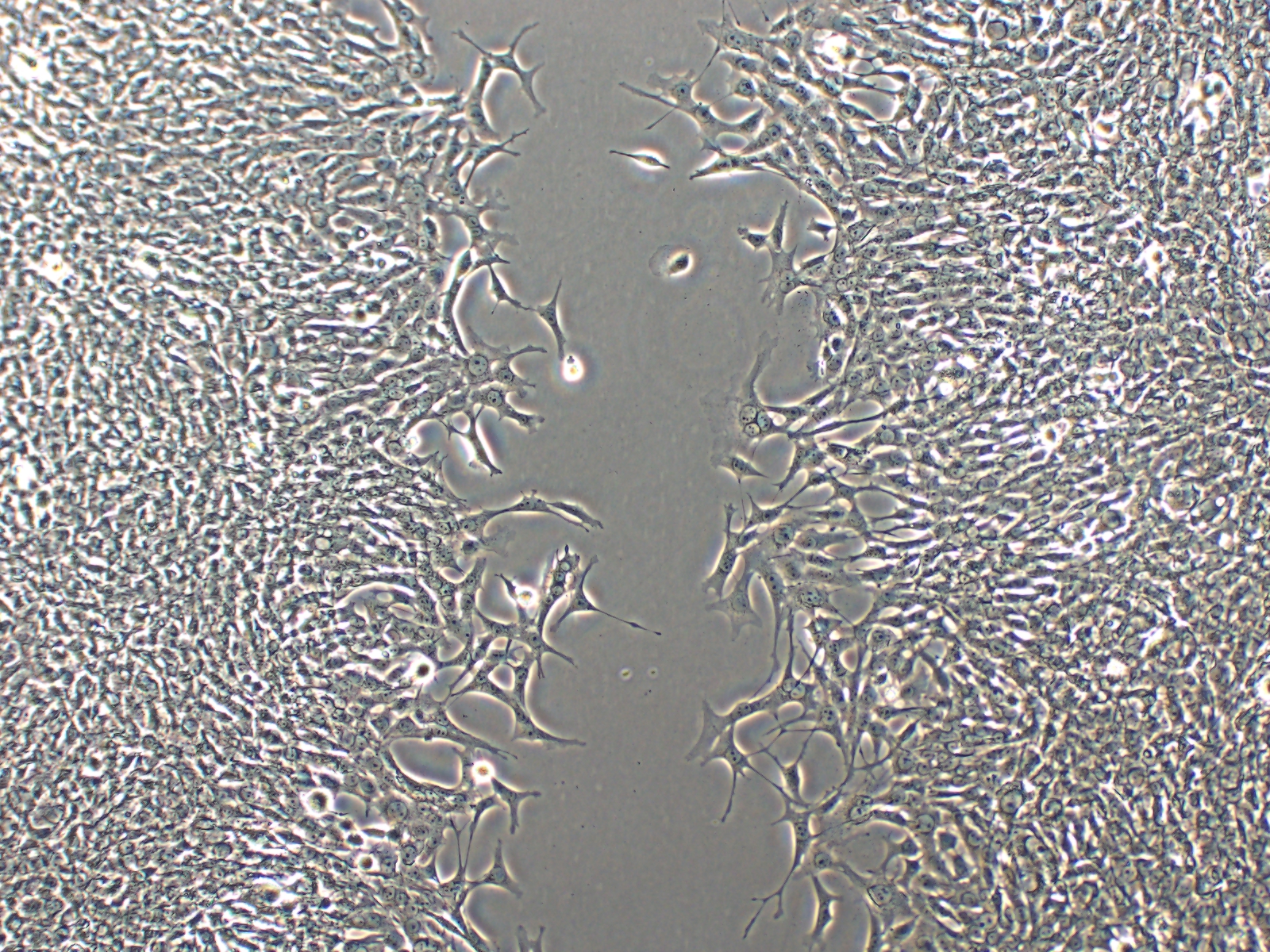

Supplement: Data S5 [file peerj-12-17664-s005.zip › raw data5/48h/BV2+LPS+BMSC-CM_C8D1A (1).jpg]

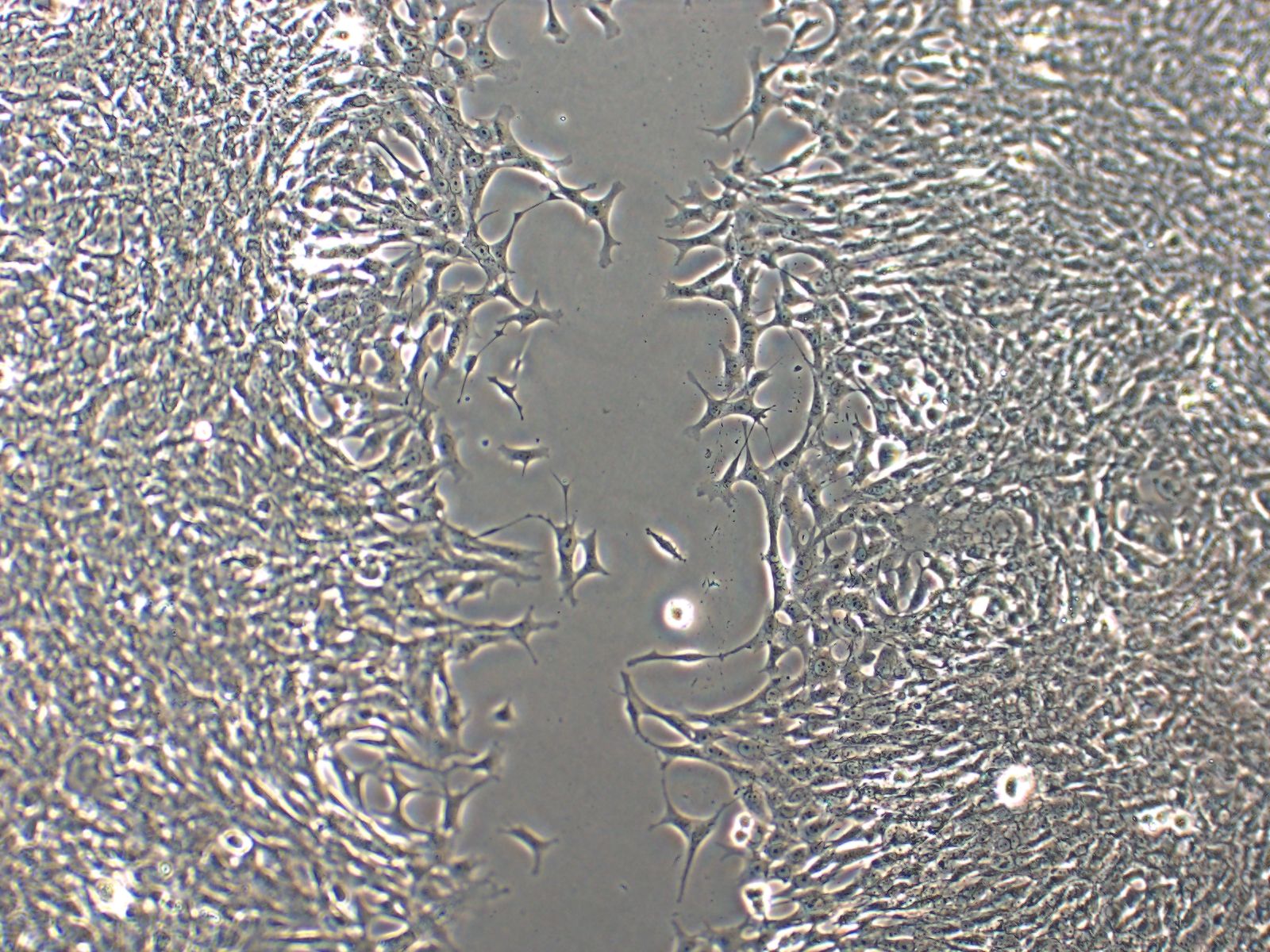

Supplement: Data S5 [file peerj-12-17664-s005.zip › raw data5/48h/BV2+LPS+BMSC-CM_C8D1A (2).jpg]

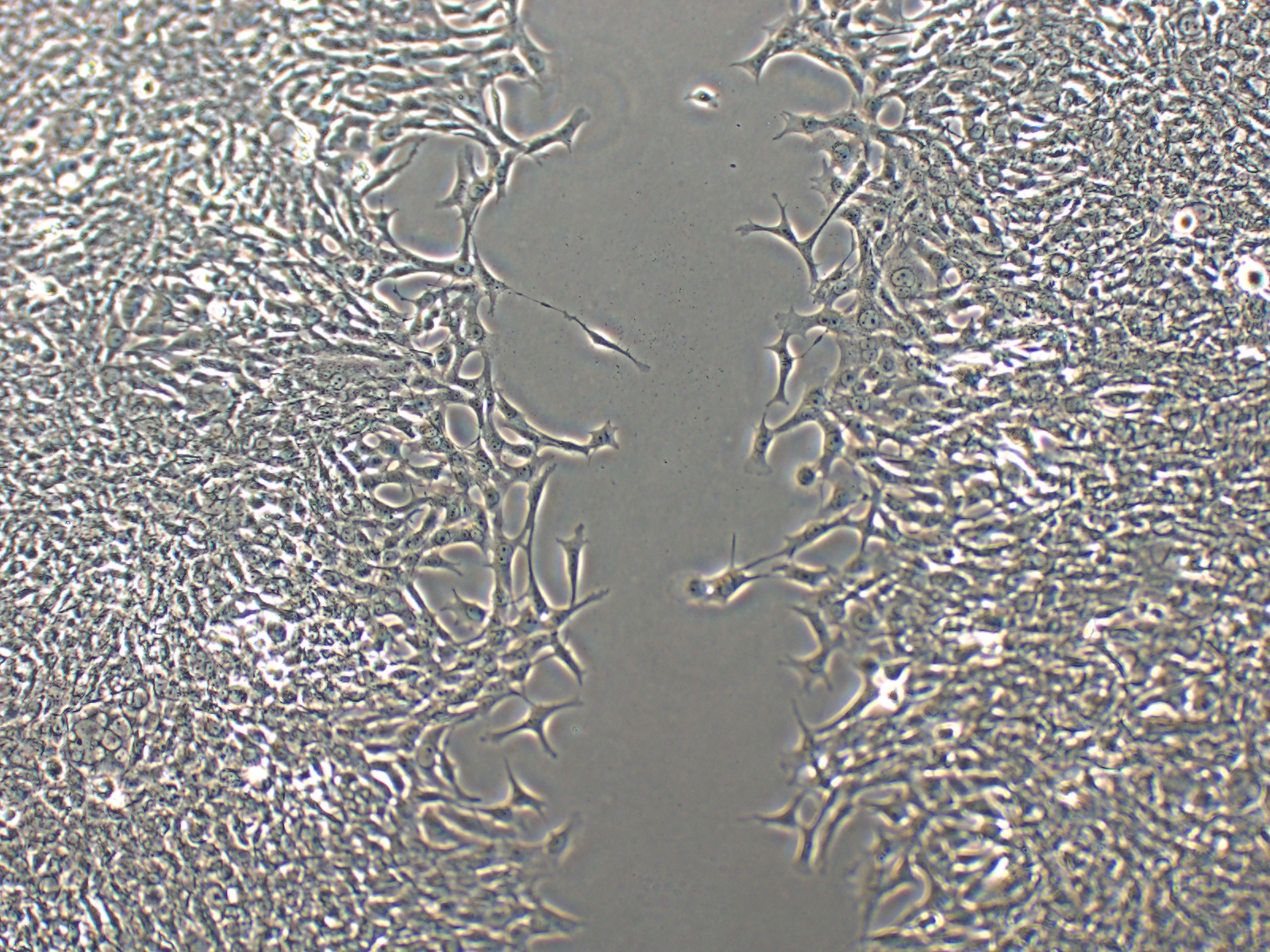

Supplement: Data S5 [file peerj-12-17664-s005.zip › raw data5/48h/BV2+LPS+BMSC-CM_C8D1A (3).jpg]
